# Effective Alkyl‐Alkyl Cross‐Coupling with an Iron‐Xantphos Catalyst: Mechanistic and Structural Insights

**DOI:** 10.1002/anie.202413566

**Published:** 2024-11-02

**Authors:** Magali Gimeno, Maria Camila Aguilera, Valerie E. Fleischauer, William W. Brennessel, Michael L. Neidig

**Affiliations:** ^1^ Inorganic Chemistry Laboratory University of Oxford South Parks Road Oxford OX1 3QR UK; ^2^ Department of Chemistry University of Rochester 120 Trustee Rd Rochester NY14627 USA

**Keywords:** Cross-Coupling, Homogeneous Catalysis, Iron, Ligand Effects, Mechanism

## Abstract

While iron‐catalyzed C(sp^2^)−C(sp^3^) cross‐couplings have been widely studied and developed in the last decade, alkyl‐alkyl cross‐coupling systems with iron remain underdeveloped despite the importance of C(sp^3^)−C(sp^3^) bonds in organic synthesis. A major challenge to the development of these reactions is the current lack of fundamental insight into ligand effects and organoiron intermediates that enable effective alkyl‐alkyl couplings. The current study addresses this longstanding limitation using a combination of ^57^Fe Mössbauer spectroscopy, SC‐XRD (single‐crystal X‐ray diffraction) and reactivity studies of alkyl‐alkyl coupling with iron‐Xantphos to define the in situ formed iron‐Xantphos intermediates in catalysis. Combined with detailed reactivity studies, the nature of the key mechanistic pathways in catalysis and ligands effects to achieve effective alkyl‐alkyl cross‐coupling over competing β‐H elimination pathways are probed. Overall, these foundational studies provide a platform for future bespoke ligand and pre‐catalyst design for alkyl‐alkyl cross‐coupling methods development with sustainable iron catalysis.

## Graphical abstract

In this study, a combination of ^57^Fe Mössbauer spectroscopy, single‐crystal X‐Ray diffraction, and reactivity studies are utilized to identify the key organoiron intermediates and reaction pathways, including ligand effects on iron speciation, that enable effective iron‐catalyzed alkyl‐alkyl cross‐couplings.

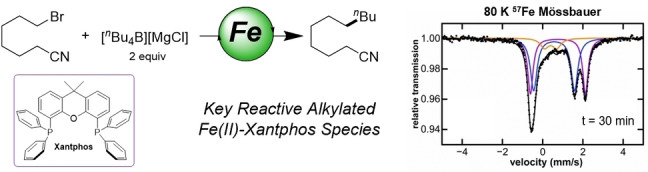

## Introduction

Cross‐coupling reactions provide an essential synthetic methodology for the construction of carbon‐carbon bonds in modern organic synthesis.[^1^, ^2^, ^3^, ^4^, ^5^] While these reactions have been historically dominated by the use of palladium‐based catalysts, the desire for more sustainable alternatives has motivated increasing interest in the development of iron‐catalyzed cross‐couplings.[^6^, ^7^, ^8^, ^9^, ^10^, ^11^, ^12^, ^13^, ^14^, ^15^, ^16^, ^17^, ^18^, ^19^, ^20^] Over the past two decades, work in this area has largely focused on the development of iron‐based methods for C(sp^2^)−C(sp^3^) bond formation in both two‐ and three‐component cross‐couplings, with complementary mechanistic studies defining the key organoiron intermediates effective in these transformations.[^21^, ^22^, ^23^, ^24^, ^25^, ^26^, ^27^, ^28^, ^29^] However, for iron cross‐couplings to be of broadest practical use in modern organic synthesis, further development of couplings beyond C(sp^2^)−C(sp^3^) are critical, though these remain significantly underdeveloped at present.

**Scheme 1:**
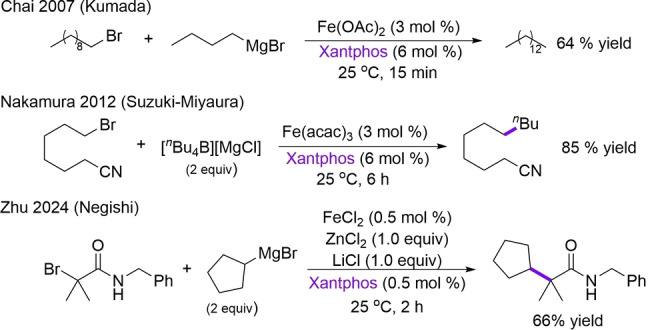
Representative examples of iron‐catalyzed alkyl‐alkyl cross‐coupling reactions using Xantphos as supporting ligand.

Of particular interest are C(sp^3^)−C(sp^3^) bonds which represent an essential scaffold for numerous biologically active molecules and their derivatives.[^30^, ^31^] Alkyl‐alkyl cross‐coupling reactions have been historically challenging with traditional palladium catalysts due to the prevalence of β‐H elimination from Pd‐alkyl intermediates involved in catalysis.[^31^, ^32^] By contrast, there is a growing body of work demonstrating that iron can be an effective catalyst for these transformations. Early examples include the work of Chai and Nakamura which demonstrated the effectiveness of iron with Xantphos ligand in both Kumada and Suzuki–Miyaura cross‐couplings of simple alkyl substrates (Scheme 1).[^33^, ^34^] More recent studies have expanded these reactions to include the formation of quaternary centers using iron‐bisphosphine catalysis including again Xantphos in a Negishi coupling (Scheme 1),^[35]^ as well as a related reductive cross‐coupling with iron‐Xantphos to yield a new C(sp^3^)−C(sp^3^) linkage.^[36]^ While Xantphos has historically been the ligand of choice for alkyl‐alkyl couplings with iron, recent studies have indicated that anilidoaldimine and diketiminate iron(II) pre‐catalysts can also be effective in Suzuki–Miyaura alkyl‐alkyl couplings.[^37^, ^38^]

While existing methods for alkyl‐alkyl cross‐coupling with iron demonstrate the synthetic potential of these reactions, challenges including limited substrate scopes and product yields combined with the common use of excess nucleophiles must be overcome in order to be competitive for practical synthetic use. Contributing to this challenge is a current lack of molecular‐level insight into the key organoiron intermediates, mechanistic pathways and ligand effects that enable effective cross‐coupling (and minimize competing β‐H elimination pathways). This limitation is in stark contrast to C(sp^2^)−C(sp^3^) cross‐couplings with iron where detailed mechanistic studies over the past decade have defined the critical role of organoiron(II) intermediates in productive catalysis and facilitated the development of new cross‐coupling methods including three‐component radical cascade couplings.^[27]^ Furthermore, a recent mechanistic proposal has suggested the potential role of iron(0) intermediates in alkyl‐alkyl cross‐couplings as the species responsible for initiation of the catalytic cycle,^[35]^ which would represent a significant deviation from the Fe(II)−Fe(III)−Fe(I) cycles in C(sp^2^)−C(sp^3^) couplings with iron‐bisphosphines.[^39^, ^40^]

It is clear that a molecular‐level understanding of the key organoiron intermediates, reaction pathways and redox cycles required for effective C(sp^3^)−C(sp^3^) couplings with iron is critical to advancing catalyst and method development for practical synthetic use in academia and industry. Towards this goal, the current study uses a combination of freeze‐trapped inorganic spectroscopy (^57^Fe Mössbauer and electron paramagnetic resonance (EPR)), inorganic synthesis and reaction studies to define the key iron intermediates and reaction pathways in iron‐catalyzed alkyl‐alkyl Suzuki–Miyaura cross‐coupling with iron‐Xantphos (Scheme 1).[^34^, ^39^, ^40^, ^41^] In contrast to recent proposals, these studies identify alkyl‐iron(II) intermediates as the key species leading to selective cross‐coupled product formation and an overall mechanism of catalysis consistent with that found in C(sp^2^)−C(sp^3^) couplings.[^25^, ^26^, ^27^, ^28^, ^29^] Further studies address the structural features of iron‐Xantphos that enable effective alkyl‐alkyl couplings as well as the potential for alternative bisphosphines ligands for these reactions catalyzed by iron. The findings on the evaluation of the ligand features demonstrate that the properties of the supporting ligand are crucial for a successful cross‐coupling reaction, representing a platform for bespoke ligand design and the development of new methods for iron catalyzed alkyl‐alkyl cross‐coupling.

## Results and Discussion

### Iron Speciation During Catalysis

**Figure 1:**
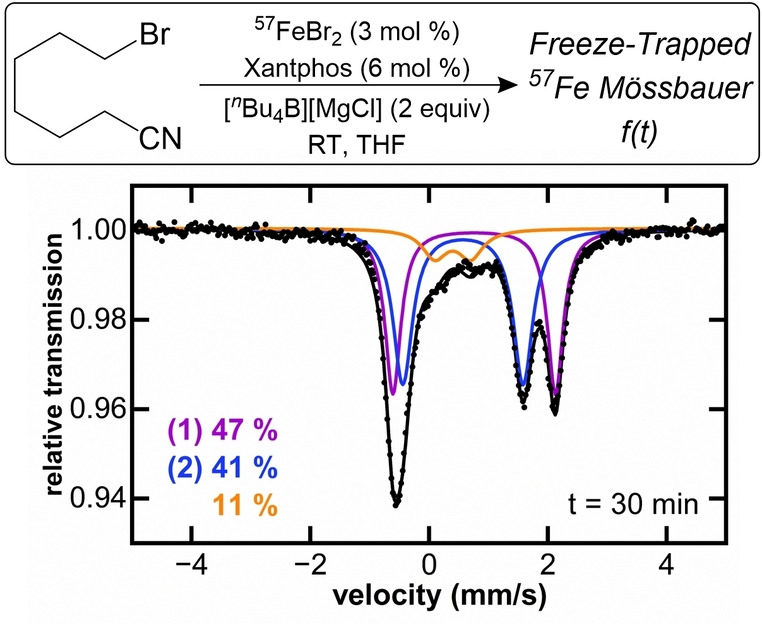
Freeze‐trapped 80 K ^57^Fe Mössbauer spectra of iron speciation during catalysis in THF. Mössbauer parameters for purple component (**1**) δ=0.78 mm/s |ΔE_Q_|=2.76 mm/s, blue component (**2**) δ=0.55 mm/s |ΔE_Q_|=2.05 mm/s and orange component δ=0.41 mm/s |ΔE_Q_|=0.64 mm/s.

To determine the key iron intermediates in iron‐Xantphos catalyzed Suzuki–Miyaura alkyl‐alkyl cross‐coupling, initial studies focused on the identification of the iron species formed in situ during catalysis. For all experiments, ^57^FeBr_2_ is used as the iron salt as it gives similar yields to Fe(acac)_3_ and removes potential complications of the mechanistic studies due to halogen exchange. Freeze‐trapped 80 K ^57^Fe Mössbauer spectroscopy of the catalytic reaction identifies three iron species formed in situ 30 minutes into the 6‐hour catalytic reaction (Figure 1). The observed Mössbauer parameters for species **1** (δ=0.78 mm/s |ΔE_Q_|=2.76 mm/s) and **2** (δ=0.55 mm/s |ΔE_Q_|=2.05 mm/s), combined with the absence of an EPR signal at 10 K, suggest that the majority of the iron intermediates (~90 % of iron in solution) present in situ during catalysis likely corresponds to high spin iron(II) complexes.[^39^, ^40^, ^42^] From previous mechanistic studies of iron‐bisphosphine catalyzed cross‐coupling systems, it is known that Fe(bisphosphine)X_2_ (X=Cl, Br or I) dihalide complexes are commonly observed as nontransmetalated resting states during catalysis.[^25^, ^26^, ^27^, ^28^] Comparing the Mössbauer parameters of these dihalide complexes with those of **1** (δ=0.78 mm/s |ΔE_Q_|=2.76 mm/s), we identified that the parameters are similar to the previously reported high spin (S=2) Fe(Xantphos)Cl_2_.^[42]^ In addition, independent synthesis, crystallization, and characterization further supported the assignment of **1** as Fe(Xantphos)Br_2_, with Mössbauer parameters of δ=0.74 mm/s |ΔE_Q_|=2.72 mm/s in the solid state (SI Figure S1, ^1^H NMR shown in Supporting Information Figure S2). This species corresponds to ~50 % of the total iron present in solution during catalysis (Figure 1). These dihalide complexes exhibit distorted tetrahedral structures with wider P−Fe−P bite angles (106° for **1‐Br** and 109° for **1‐Cl** analogue,^[42]^ Figure 3) than analogues with other bisphosphines (80–91°), as expected for Xantphos.[^42^, ^43^, ^44^]

An additional minor iron species (orange component fitted as δ=0.41 mm/s |ΔE_Q_|=0.64 mm/s) is also present, which grows in magnitude throughout the catalytic reaction (SI Figure S3). However, if catalysis is performed in 2‐MeTHF instead of THF, similar yields of cross‐coupled product are formed but this third species is not observed at analogous time points in catalysis and is only observed to form as a minor component after several hours of reaction (SI Figure S4). Combined with reactivity studies (vide infra), these observations are consistent with formation of an off‐cycle species in catalysis. Due to the reduced solubility of the catalyst in 2‐MeTHF, all subsequent studies were performed in THF.

Catalysis with 1 equivalent of Xantphos with respect to iron, rather than the 2 equivalents found to be optimal by Nakamura, negatively impacted catalytic performance, yielding ~65 % cross‐coupled product, ~10 % side product (heptanenitrile) and ~25 % unreactive electrophile (Table S1). Iron speciation under these conditions, evidenced by 80 K ^57^Fe Mössbauer spectroscopy (see Supporting Information Figure S5), showed a marked increase in the formation of the species with parameters δ=0.41 mm/s and |ΔE_Q_|=0.64 mm/s, which was accompanied by formation of a black suspension, indicative of catalyst decomposition. ^31^P NMR experiments under these conditions evidenced free Xantphos in solution, suggesting that the third species observed in catalysis likely forms following ligand dissociation. These observations alongside the fact that the reaction in 2‐MeTHF shows lower amounts of this off‐cycle species, suggests solvent coordination, contributing to phosphine dissociation in solution. These results indicate that the extra equivalent of Xantphos used in Nakamura's protocol aids to prevent catalyst decomposition through displacement of the bisphosphine.

### Identification and Characterization of Alkylated Iron(II)−Xantphos Intermediates

**Figure 2:**
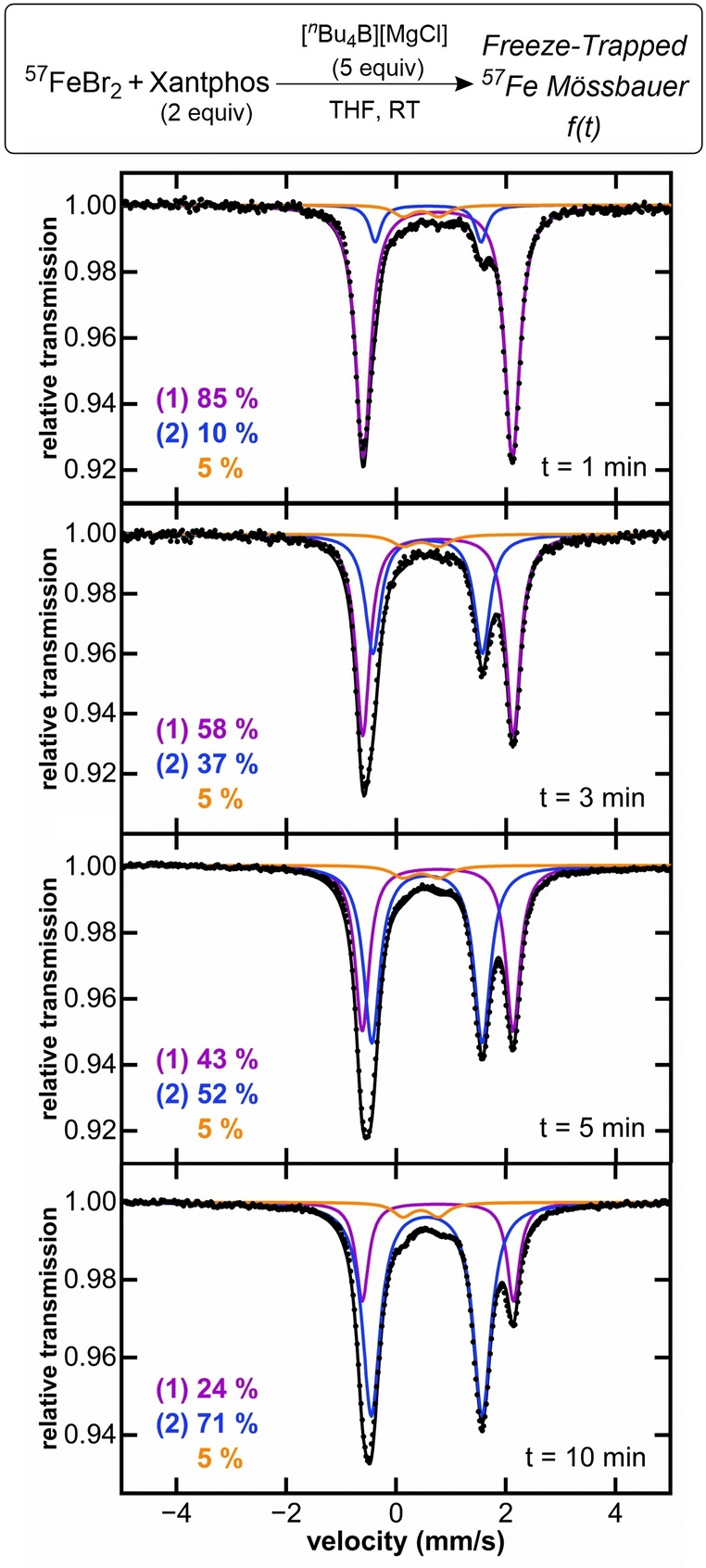
Freeze‐trapped 80 K ^57^Fe Mössbauer spectra of reaction of Fe(Xantphos)Br_2_ with ^*n*^butylborane. Mössbauer parameters for purple component δ=0.78 mm/s |ΔE_Q_|=2.76 mm/s (**1‐Br**), blue component δ=0.55 mm/s |ΔE_Q_|=2.05 mm/s (**2**), orange component δ=0.41 mm/s |ΔE_Q_|=0.64 mm/s.

To assign the iron species observed during catalysis and evaluate the possible organoiron intermediates that may be accessible in situ, the iron speciation upon reaction of **1** with alkyl nucleophiles was evaluated at catalytically relevant conditions (solvent, temperature and iron concentration). While **1** exhibits no reactivity towards electrophile, transmetalation with nucleophile could result in the formation of Xantphos‐iron(II)‐alkyl complexes. To evaluate this possibility, we performed freeze‐trapped 80 K ^57^Fe Mössbauer spectroscopy to determine the iron speciation in the reaction of Fe(Xantphos)Br_2_ with nucleophile (Figure 2). As transmetalation with stoichiometric amounts (1 equivalent with respect to iron) with ^*n*^butylborate is remarkably slow, 5 equivalents of nucleophile were utilized in order to access iron species in a reasonable time frame. After 3 minutes, ~40 % of **1** had transmetalated leading to the formation of a species with Mössbauer parameters δ=0.55 mm/s and |ΔE_Q_|=2.05 mm/s (Figure 2). These are identical to species **2** observed during catalysis, indicating that **2** can be accessed readily in situ from the direct reaction of **1** with nucleophile. Following its formation, **2** remained stable at room temperature for several minutes. Alkylation of **1** leads to a reduced isomer shift, as observed for **2**, due to the formation of a shorter, more covalent Fe−C bond compared to the more ionic Fe−X bond (X=Cl, Br).^[45]^ This is consistent with Mössbauer parameters of mono‐ and bistransmetalated species, shown in previous studies (Table 1).[^25^, ^26^, ^27^] Based on these observations, we hypothesized that **2** likely corresponds to the iron(II) monoalkylated Fe(Xantphos)(^*n*^Bu)Br species. Unfortunately, crystallization attempts of **2** were not successful, likely due to the flexibility of the *n*‐butyl chain. Thus, to further support this assignment, synthesis of alternative alkyl analogues that would be more synthetically accessible and could provide further support for the assignment was performed.

**Table 1:** ^57^Fe Mössbauer parameters for Fe(II)‐bisphosphine complexes in frozen THF solution.[^25^, ^26^, ^27^] SciOPP: 1,2‐Bis[bis[3,5‐di(t‐butyl)phenyl]phosphino]‐benzene; dcype: 1,2‐bis(dicyclohexylphosphino)ethane

Category	Complex	δ (mm/s)	|ΔE_Q_| (mm/s)
Dihalide	Fe(SciOPP)Br_2_	0.94	2.95
	Fe(SciOPP)Cl_2_	0.94	2.69
	Fe(dcype)Br_2_	0.72	3.21
Mono‐aryl	Fe(SciOPP)(Ph)Br	0.50	2.37
	Fe(SciOPP)(Mes)Br	0.52	2.12
	Fe(dcype)(3‐MeOC_6_H_4_)Br	0.51	2.49
Bis‐aryl	Fe(SciOPP)(Ph)_2_	0.33	1.50
	Fe(SciOPP)(Ph)_2_(THF)	0.32	3.13
	Fe(SciOPP)(Mes)_2_	0.28	3.67
	Fe(dcype)(3‐MeOC_6_H_4_)_2_	0.23	4.35

**Figure 3:**
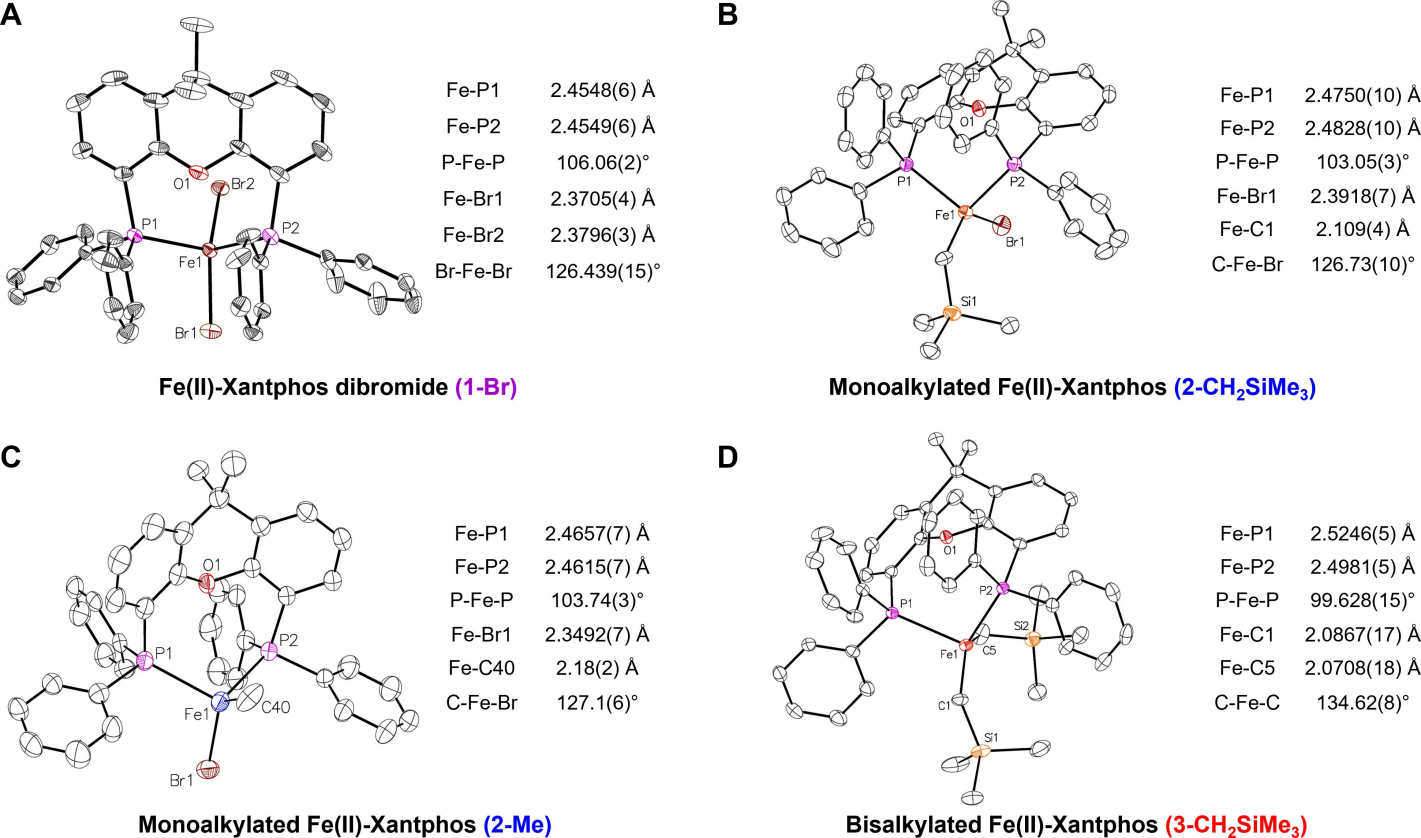
SC‐XRD of isolated crystalline material of iron(II)−Xantphos intermediates. A) Fe(II)−Xantphos dibromide (**1‐Br**). B) Monoalkylated Fe(II)−Xantphos (**2‐CH_2_SiMe_3_**). C) Monoalkylated Fe(II)−Xantphos (**2‐Me**). D) Bisalkylated Fe(II)−Xantphos (**3‐CH_2_SiMe_3_**). Thermal ellipsoids are shown at 50 % probability.^[58]^

The use of EtMgBr, MeMgBr and CH_2_SiMe_3_MgCl, led to the corresponding mono‐alkylated iron‐Xantphos species (Fe(Xantphos)(Et)Br, Fe(Xantphos)(Me)Br and Fe(Xantphos)(CH_2_SiMe_3_)Br respectively), with Mössbauer parameters shown in Table 2. The similarities in these parameters indicate the electronic and geometric structure around the iron center is analogous for all the formed mono‐alkylated iron‐Xantphos species. Single crystals suitable for SC‐XRD were successfully obtained for the (trimethylsilyl)methyl and methyl (**2‐CH_2_SiMe_3_** and **2‐Me** respectively, Figure 3) alkylated Xantphos complexes. The 80 K ^57^Fe Mössbauer parameters of the solid samples from isolated crystalline material are δ=0.59 mm/s |ΔE_Q_|=1.91 mm/s and δ=0.57 mm/s |ΔE_Q_|=1.96 mm/s, respectively (SI Figure S6), similar to the parameters observed for **2**. ^1^H NMR of **2‐CH_2_SiMe_3_** shown in Supporting Information Figure S7.

**Table 2:** 80 K ^57^Fe Mössbauer parameters for alkylated Fe(II)−Xantphos complexes frozen THF solution and solid samples.

Complex	sample	δ (mm/s)	|ΔE_Q_| (mm/s)
Fe(Xantphos)(^*n*^Bu)Br	frozen soln.	0.55	2.05
Fe(Xantphos)(Me)Br	frozen soln.	0.55	1.99
	solid	0.57	1.96
Fe(Xantphos)(Et)Br	frozen soln.	0.54	1.98
Fe(Xantphos)(CH_2_SiMe_3_)Br	frozen soln.	0.56	1.97
	solid	0.59	1.91
Fe(Xantphos)(CH_2_SiMe_3_)_2_	frozen soln.	0.38	1.11
	solid	0.44	1.03
Fe(Xantphos)(^*n*^Bu)_2_	frozen soln.	0.25	1.33

Both alkylated complexes exhibit similar structural parameters with a smaller P−Fe−P bite angle from that observed for **1‐Br** (106° vs 103°). Note that the effective magnetic moment of **2‐CH_2_SiMe_3_** in THF was determined to be 5.3(3) μ_B_ by Evans method NMR. Overall, these observations support the assignment of **2** as the high spin iron(II) species Fe(Xantphos)(^*n*^Bu)Br.

**Figure 4:**
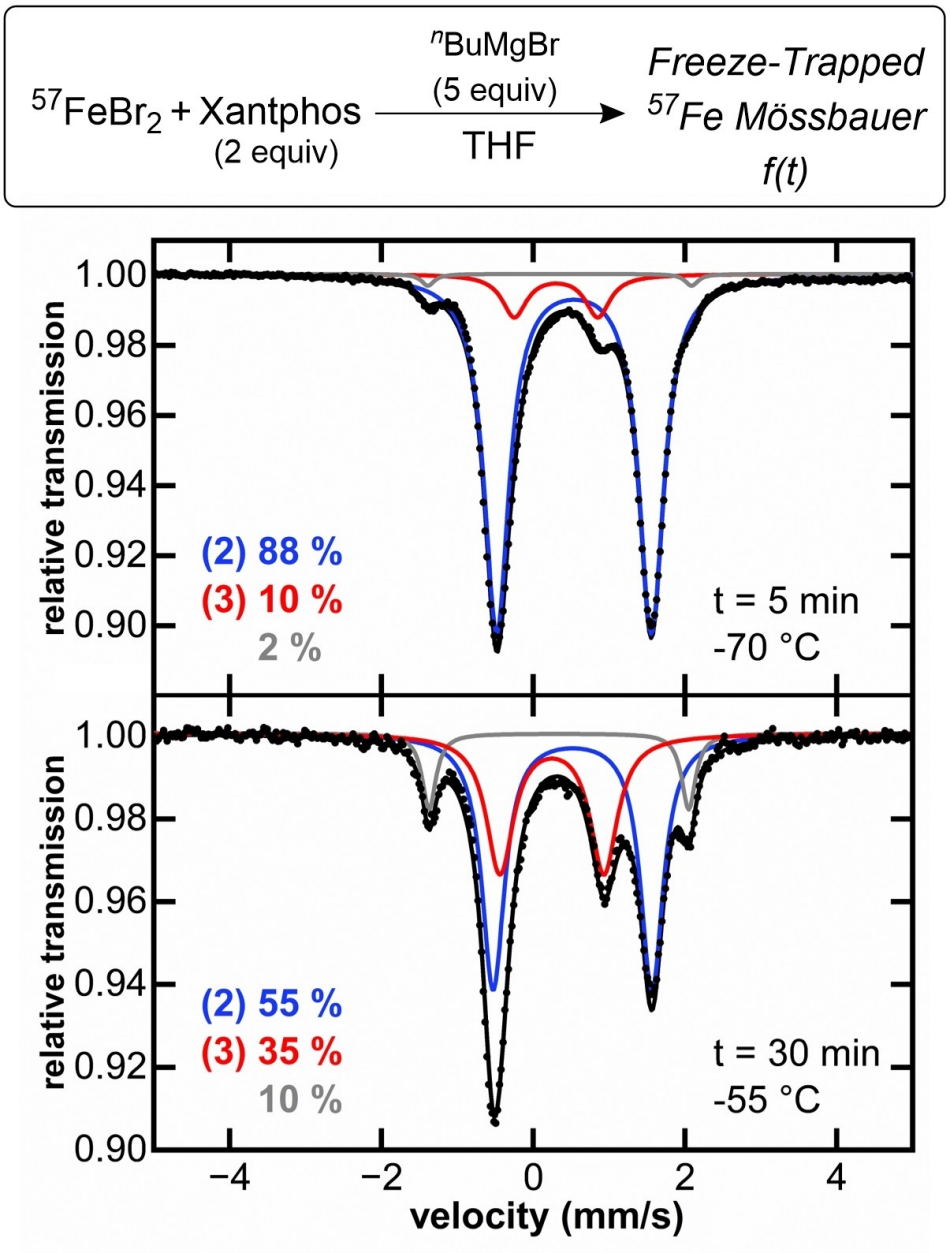
Freeze‐trapped 80 K ^57^Fe Mössbauer spectra of reaction of **1** with 5 equiv. of ^*n*^BuMgBr. The reaction time and temperature are indicated within each spectrum. Parameters for blue component δ=0.55 mm/s |ΔE_Q_|=2.05 mm/s (**2**), red component δ=0.25 mm/s |ΔE_Q_|=1.33 mm/s (**3**), grey component δ=0.34 mm/s |ΔE_Q_|=3.47 mm/s.

The ability of **2** to further transmetalate to form a bisalkylated species was also evaluated, as similar bistransmetalated species have been commonly observed with aryl nucleophiles.[^25^, ^26^, ^27^, ^28^] Due to the bulkiness and slower transmetalation rates of the borate nucleophile compared to their Grignard counterpart, the formation of monoalkylated species does not reach completion at room temperature (vide supra). However, the possibility of forming a bis‐alkylated complex with the borate nucleophile at RT, in amounts undetectable by Mössbauer spectroscopy, cannot be dismissed. This hypothesis was tested by addition of excess of ^*n*^BuMgBr to a solution of **1** at −70 °C. The reaction mixture showed color change to orange (indicating formation of **2**), which further evolved to dark red. This observation was accompanied by the formation of two new iron species, confirmed by freeze‐trapped 80 K ^57^Fe Mössbauer spectroscopy, with parameters δ=0.25 mm/s, |ΔE_Q_|=1.33 mm/s (**3**) and δ=0.34 mm/s, |ΔE_Q_|=3.47 mm/s (Figure 4). The observed decrease in the isomer shift (δ) for **3** compared to **2** is suggestive of further transmetalation, as previously observed for other iron(II)‐bisphosphines (Table 1),[^25^, ^26^, ^27^] consistent with formation of the iron (II) bis‐alkylated Xantphos species. The parameters of the species show as the grey component are consistent with the coordination of THF to **3** to form a THF adduct complex, as observed for Fe(II)−SciOPP bisphenyl complexes (Table 1).^[26]^ To further support the assignment of **3**, we proceeded to the isolation of a tetrahedral bisalkylated Xantphos analogue, Fe(Xantphos)(CH_2_SiMe_3_)_2_ (**3‐CH_2_SiMe_3_** Figure 3) with Mössbauer parameters δ=0.38 mm/s, |ΔE_Q_|=1.11 mm/s (Table 2), consistent with the species observed at low temperatures (**3**, δ=0.25 mm/s, |ΔE_Q_|=1.33 mm/s, Figure 4). Solid Mössbauer and ^1^H NMR of isolated crystals are shown in Supporting Information Figure S6 and Figure S8 respectively.

When left to run for longer time and warmed to room temperature, the reaction continued to convert into a new species with parameters similar to minor component during catalysis shown in orange, δ=0.41 mm/s |ΔE_Q_|=0.64 mm/s (SI Figure S9). This was accompanied by a color change from red to black, indicating the instability and further decomposition of the species observed in Figure 4. The evidence provided to this point, including speciation during catalysis with 1 and 2 equivalents of Xantphos (vide supra), suggests that the orange component is an off‐cycle species, potentially multinuclear iron species formed by dissociation of Xantphos from the iron center (vide supra).[^43^, ^44^, ^46^] The Mössbauer parameters of said species and its broad doublet at 80 K are consistent with previously observed multinuclear iron species,[^47^, ^48^, ^49^, ^50^, ^51^] indicating a pathway to the deactivation of the catalytically active species and the end of the reaction. The experimental data supporting this hypothesis is included in the Supporting Information (Figures S3 and S5).

**Figure 5:**
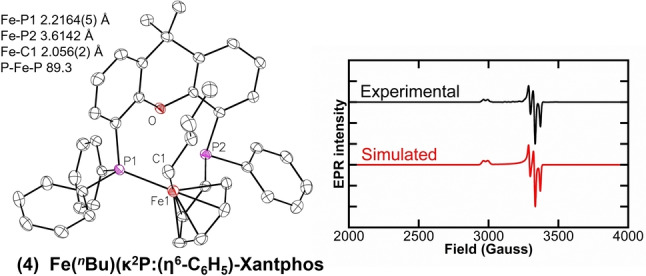
EPR characterization of **4**, Fe(I) *S*=1/2. Simulated g values of g_1_=2.25, g_2_=2.02and g_3_=1.99, and hyperfine coupling to the ^31^P from the Xantphos ligand with constant A_1_=111.4, A_2_=91.9 and A_3_=102.5 MHz. Thermal ellipsoids of **4** are shown at 50 % of probability.^[58]^ Fe−C (phenyl ring) bond length range 2.0853(19) – 2.136(2) Å.

Lastly, while attempting to crystallize the alkylated intermediates (mono‐ and bisalkylated), an additional reduced iron(I) species was formed at long incubation times: Fe(^*n*^Bu)(κ^2^P:(η^6^‐C_6_H_5_)‐Xantphos (**4**). This novel iron(I) complex exhibits an unusual structure where one of the phosphorus atoms is detached from the iron center, replaced by the neighboring phenyl moiety coordinated to the iron center in an η^6^ fashion (Figure 5). Intriguingly, this complex preserves the integrity of the alkyl ligand, suggesting that the reduction pathway may not involve an intermediate going through β‐H elimination as commonly expected for metal‐alkyl species.[^31^, ^32^] The Mössbauer parameters of isolated crystals of **4** are δ=0.47 mm/s |ΔE_Q_|=0.86 mm/s (SI Figure S10). In addition, EPR measurements indicate a *S*=1/2 signal with g values of g_1_=2.25, g_2_=2.02 and g_3_=1.99, and hyperfine coupling to the ^31^P from the Xantphos ligand with constant A_1_=111.4, A_2_=91.9 and A_3_=102.5 MHz (based on EPR simulation, Figure 5). Species **4** is not observed during catalysis and reactivity studies with isolated material showed no reactivity with electrophile, suggesting that the species corresponds to an off‐cycle thermodynamic product that forms at extended reaction times.

### Reactivity and Mechanism

**Figure 6:**
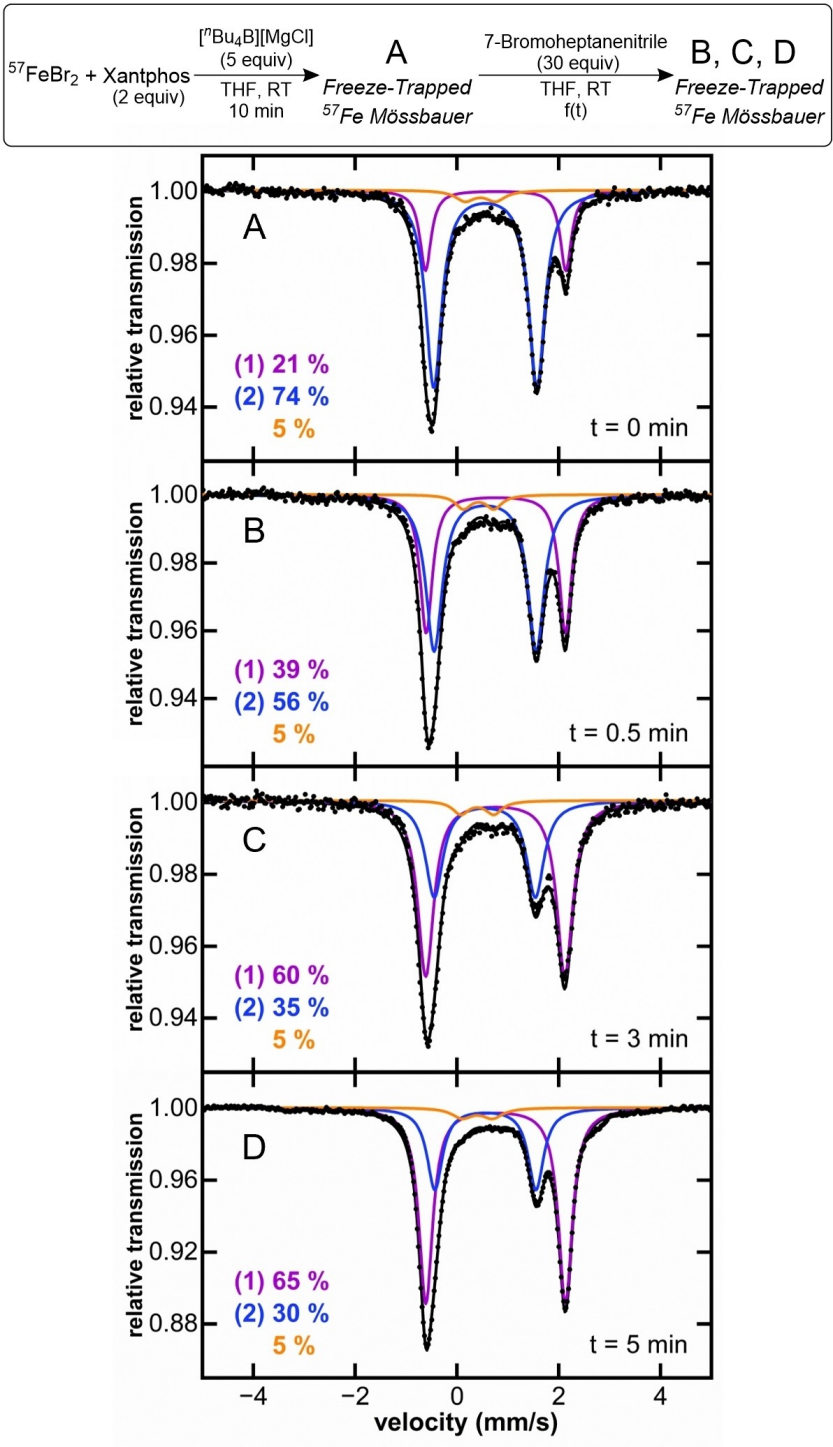
Freeze‐trapped 80 K ^57^Fe Mössbauer spectra of the in situ formed iron species upon reaction of ^57^FeBr_2_ and 2 equiv. of Xantphos, with 5 equiv. of [^*n*^Bu_4_B][MgCl] for 10 min (A) and following subsequent reaction with 7‐bromoheptanenitrile at different timepoints (B, C & D).

While a recent mechanistic proposal has suggested an alternative iron(0) pathway for iron alkyl‐alkyl cross‐couplings with bisphosphines such as Xantphos,^[35]^ it is notable that the iron distribution observed in the current study is similar to that previously observed in numerous types of cross‐couplings (i.e. with iron‐bisphosphines and aryl nucleophiles). Mono‐ and bisarylated Fe(II) species are accessible in those systems, and such species have been identified as key reactive intermediates for radical initiation.[^25^, ^26^, ^27^, ^28^, ^29^] Therefore, the reactivity of Fe(II) species in this system was tested, assessing the relative reactivity of **2** with 30 equivalents of 7‐bromoheptanenitrile at catalytically relevant conditions (10 mM concentration iron at room temperature). A control Mössbauer sample was taken 10 minutes after the addition of 5 equivalents of nucleophile (t=0 min), where **2** is maximally formed in situ. This was followed by the addition of the electrophile, and freeze‐trapped Mössbauer samples were taken at 30 seconds, 3 minutes, and 5 minutes (Figure 6). Consumption of 44 % of **2** after the addition of excess electrophile occurred at an observed rate of k_obs_=0.19(4) min^−1^, with the corresponding formation of 30 % cross‐coupled product with respect to iron, and 14 % of side products (heptanenitrile and 6‐heptenenitrile). These results are consistent with prior studies for two and three component iron‐bisphosphine catalyzed C(sp^2^)−C(sp^3^) couplings,[^25^, ^26^, ^27^, ^28^] where transmetalated iron(II) bisphosphine intermediates are responsible for product formation. The remaining question is then, what iron species initiates radical activation to start catalysis? Previous examples with SciOPP as ligand have shown that both mono‐ and bistransmetalated species can be effective for radical initiation.^[26]^ In this work, the observed reaction rate of **2** with electrophile (k_obs_=0.19(4) min^−1^) is faster than the average turnover during catalysis (approximately 0.1 min^−1^), arguing that, not only its consumption is directly related to product formation, demonstrating that it is a key intermediate, but also that the reactivity of this monoalkyl intermediate is fast enough to be responsible for radical initiation. Additionally, the fact that we observed **2** during catalysis (vide supra) is consistent with transmetalation of **1** to generate **2** being faster than the reactivity of **2** with electrophile. Unfortunately, the challenges associated with transmetalation with the bulky tetrabutylborate prevent in situ generation of pure bisalkyl (**3**) at catalytic conditions to perform relevant reactivity studies. Nevertheless, the participation of the bisalkylated Xantphos intermediate in catalysis cannot be discarded, as undetectable amounts of this species could be generated, initiating radical by its immediate reaction with electrophile.

**Scheme 2:**
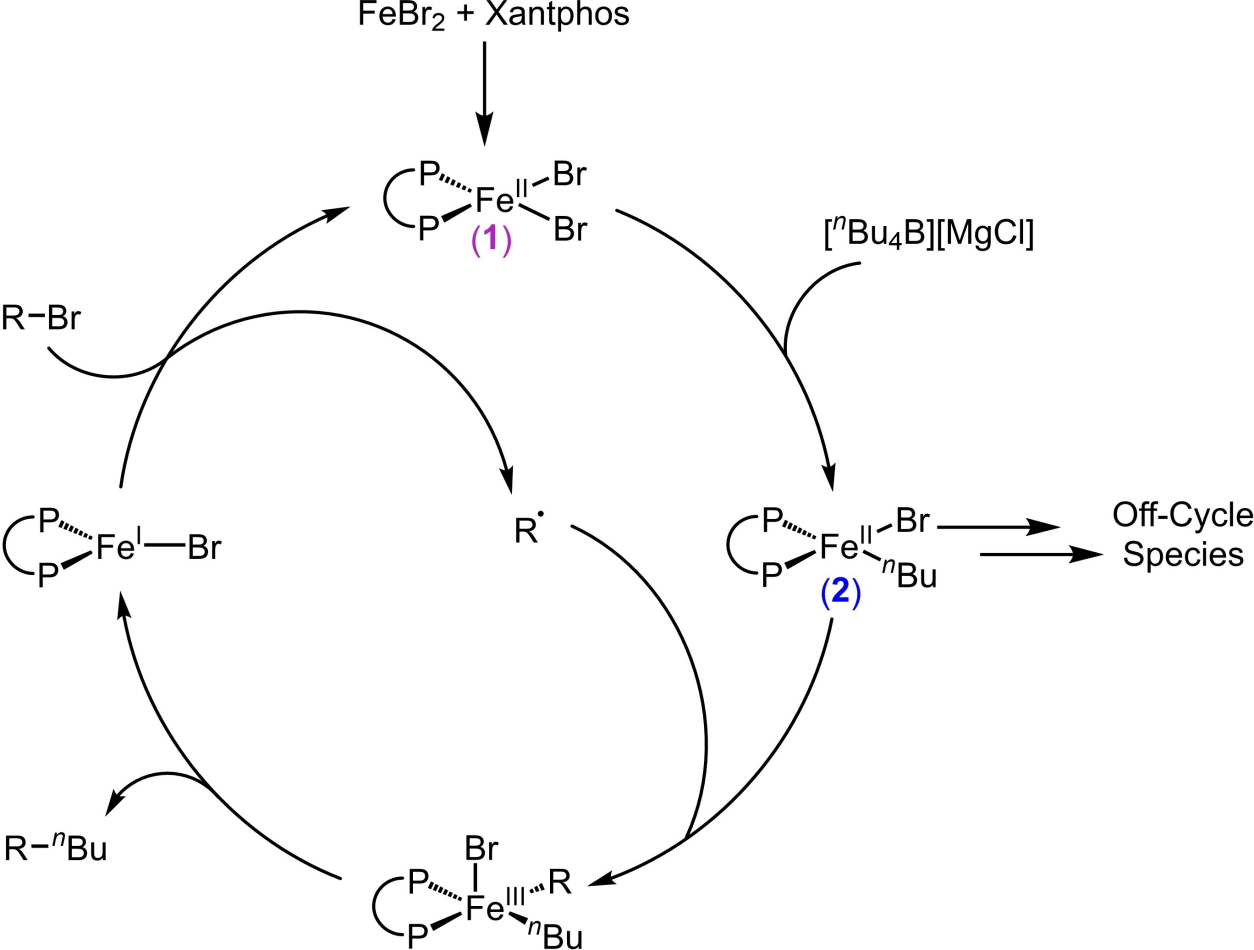
Proposed mechanistic cycle for Fe‐Xantphos catalyzed C(sp^3^)−C(sp^3^) cross‐couplings.

Regardless of how the first radical is initiated, the experimental evidence shown to this point argues that this alkyl‐alkyl system behaves very similarly to previous iron‐bisphosphine catalyzed C(sp^2^)−C(sp^3^) couplings,[^25^, ^26^, ^27^, ^28^] where radical recombines with monophenylated species, generating a five‐coordinate iron(III) intermediate which subsequently undergoes reductive elimination to generate cross‐coupled product and an iron(I) bisphosphine species. This iron(I) complex can then react with electrophile to generate additional organoradical and the precatalyst. Accordingly, we hypothesized that this iron‐Xantphos catalyzed alkyl‐alkyl cross‐coupling of Nakamura and co‐workers proceeds through an equivalent mechanism (Scheme 2) where organoiron(II) intermediates are central to selective cross‐coupled product formation.

Lastly, consumption of the orange component from catalysis during the reactivity studies was not observed, indicating that this species is unlikely to be involved in the catalytic cycle and further continue as an off‐cycle species.

### Identifying Ligand Features that Correlate to Effective Alkyl‐Alkyl Cross‐Coupling

With the reactive iron intermediates and reaction pathways established, we next turned to the core question of what enables Xantphos as an effective supportive ligand for alkyl‐alkyl cross‐coupling,[^33^, ^34^, ^35^, ^36^] and what are the key aspects of the supporting ligand required for effective coupling. A key distinguishing feature of Xantphos compared to other bisphosphines commonly employed in cross‐coupling is its large bite angle (>100° (vide supra) vs ~85°) and rigid backbone.^[42]^ While this feature might be anticipated to be a core predictor for effective catalysis, prior studies by Chai and co‐workers for a Kumada alkyl‐alkyl cross‐coupling found related large bite angle ligands such as DPEphos (Bis[(2‐diphenylphosphino)phenyl]ether), which possess a flexible backbone, are ineffective for catalysis.^[33]^ In light of these prior observations, our initial studies of ligand effects in alkyl‐alkyl cross‐couplings focused on why DPEphos is ineffective despite its similar bite angle to Xantphos as well as the origin of poor reactivity with simple bisphosphines such as dppe (1,2‐Bis(diphenylphosphino)ethane) and dpbz (1,2‐Bis(diphenylphosphino)benzene) which have been successfully employed in C(sp^2^)−C(sp^3^) cross‐couplings with iron.[^52^, ^53^, ^54^]

**Figure 7:**
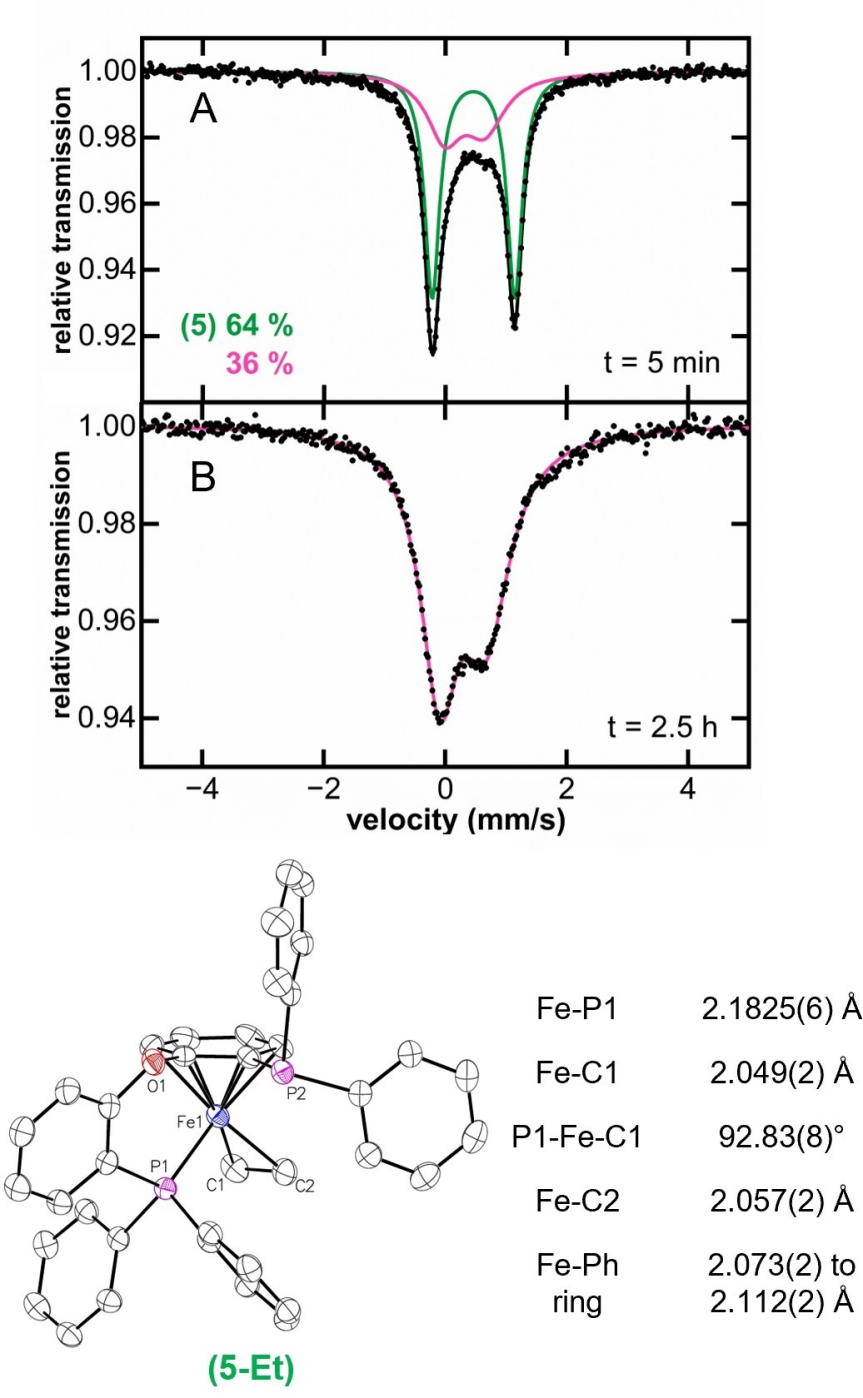
Freeze‐trapped 80 K ^57^Fe Mössbauer spectra of A) Reaction of FeBr_2_ with 60 equivalents *n*‐butylborane in the presence of 2 equivalents of DPEphos, Mössbauer parameters for green component (**5**) δ=0.46 mm/s |ΔE_Q_|=1.36 mm/s, Mössbauer parameters for pink component δ=0.28 mm/s |ΔE_Q_|=0.76 mm/s. B) Catalytic reaction solution performed using 2 equivalents of DPEphos at 2.5 h. Crystal structure of **5‐Et**, thermal ellipsoids are shown at 50 % probability.^[58]^

We started by evaluating DPEphos performance in the current catalytic system, where this ligand was also ineffective, giving only 6 % of cross‐coupled product, 60 % of recovered electrophile (7‐bromoheptanitrile) and 30 % of side products (6‐heptenenitrile and heptanenitrile, see Supporting Information Table S1). This result indicates that bite angle alone is insufficient to explain catalytic performance, and other features such as backbone rigidity might be equally critical (note that DPEphos lacks the backbone rigidity present for Xantphos). To determine the molecular‐level origin of the poor reactivity with DPEphos, we proceeded to investigate the generated iron intermediates upon reaction of FeBr_2_ with excess ^*n*^butylborane in the presence of DPEphos. After addition of nucleophile, a rapid color change from colorless to dark orange was observed, accompanied by the formation of two iron species after 5 min, a new iron species with δ=0.46 mm/s |ΔE_Q_|=1.36 mm/s (**5**) and a broad, asymmetric doublet that could be fit to Mössbauer parameters of δ=0.28 mm/s |ΔE_Q_|=0.76 mm/s (Figure 7A). While **5** could not be isolated, using EtMgBr as an alkyl nucleophile for this reaction enabled the isolation and SC‐XRD characterization of the diamagnetic species **5‐Et** (Figure 7). Note that this complex has a similar κ^2^P:(η^6^‐C_6_H_5_)‐bisphosphine interaction with an η^2^‐ethylene bound to Fe(0) compared to the η^1^‐bound alkyl to Fe(I) present in **4**, which is evidence of a β‐H elimination step prior to reduction. Furthermore, **5** can also be accessed by stoichiometric reaction of FeBr_2_ and DPEphos with ^*n*^BuMgBr at low temperature, where the formation of another iron species with parameters consistent with a monoalkylated intermediate (δ=0.55 mm/s and |ΔE_Q_|=2.00 mm/s) is observed (SI Figure S11). This suggests the initial formation of Fe(DPEphos)(^*n*^Bu)Br (**2‐DPEphos**) complex previous to β‐H elimination followed by reduction to yield **5**. Mössbauer parameters of the isolated crystalline material of **5‐Et** (δ=0.45 mm/s and |ΔE_Q_|=1.33 mm/s, Supporting Information Figure S12) are consistent with those observed for **5**, leading to the assignment of **5** as the butene analog of **5‐Et**.

Next, the iron speciation distribution during catalysis was determined. 80 K ^57^Fe Mössbauer revealed the same broad, asymmetric doublet observed in the stoichiometric experiments (δ=0.28 mm/s |ΔE_Q_|=0.76 mm/s, Figure 7B). No EPR active species are observed by 10 K EPR spectroscopy. ^31^P NMR of the reaction indicates the presence of free DPEphos in solution (SI Figure S13), consistent with dissociation of the ligand during catalysis. This conclusion is further supported by freeze‐trapped ^57^Fe Mössbauer of the catalytic reaction performed in absence of DPEphos (only FeBr_2_, electrophile and activated *n*‐butylborane), which results in the same iron distribution in situ (SI Figure S13). While the iron species formed during catalysis could not be isolated despite extensive efforts, the broadness and asymmetry features of the 80 K ^57^Fe Mössbauer spectrum combined with the magnetic hyperfine splitting observed in 5 K ^57^Fe Mössbauer measurements supports the formation of multinuclear iron species or clusters (SI Figure S14).[^47^, ^48^, ^49^, ^50^, ^51^] The formation of iron clusters suggests decomposition of the alkylated iron‐DPEphos intermediates, probably through facile β‐H elimination and subsequent reduction when using DPEphos instead of Xantphos.

We hypothesize that initial formation of the iron(0)‐DPEphos complex **5** at early stages of catalysis, eventually transforms into reduced iron clusters upon DPEphos dissociation. Overall, these observations highlight the drastic effect that the backbone rigidity has on avoiding β‐H elimination of the iron‐alkyl intermediates since, in sharp contrast to Xantphos, no iron‐DPEphos alkylated species can be observed at RT.

**Figure 8:**
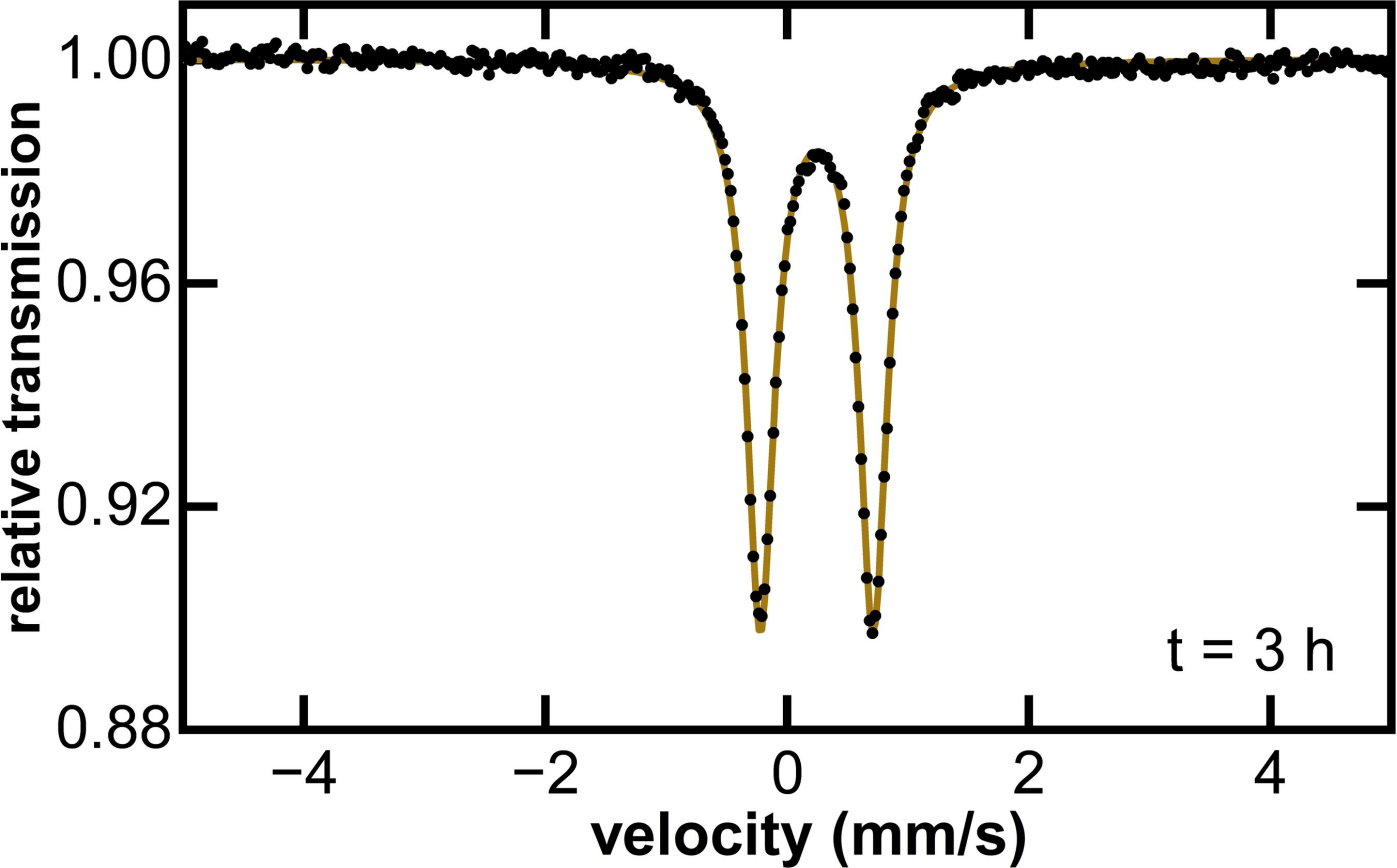
Freeze‐trapped 80 K ^57^Fe Mössbauer spectrum of the iron speciation during catalysis with dppe after 3 hours. Mössbauer parameters δ=0.25 mm/s |ΔE_Q_|=0.99 mm/s.

Beyond wide bite angle bisphosphines, it was also important to determine why bisphosphines with smaller bite angles such as dppe have not been effective in alkyl‐alkyl cross‐couplings despite their common use for C(sp^2^)−C(sp^3^) iron cross‐coupling methods. Is the ineffectiveness of dppe due to a rapid β‐H elimination and subsequent reduction to unproductive low‐valent iron complexes or clusters? Furthermore, can modifying the ligand electronics/rigidity upon moving to the related ligand dpbz lead to improved catalytic performance? To address these questions, we first evaluated the catalytic performance of dppe and dpbz in place of Xantphos for alkyl‐alkyl cross‐coupling of 7‐bromoheptanenitrile and activated tributylborane ([^*n*^Bu_4_B][MgCl]). In both cases, 99 % recovered electrophile was observed after the 6 hours catalytic reaction when using 2 equivalents of the corresponding ligand following the catalytic protocol reported. Note that these observations are consistent with the original paper by Nakamura where they reported poor yields (3 %) for dppe as supporting ligand.^[34]^ Interestingly, the iron speciation during catalysis with both dppe and dpbz shows the formation of a major species (see Figure 8 for dppe and Supporting Information Figure S15 for dpbz) with analogous Mössbauer parameters to one another (e.g. δ=0.25 mm/s |ΔE_Q_|=0.99 mm/s with dppe, consistent with a six‐coordinate low spin Fe(II)).[^27^, ^55^, ^56^] ^31^P NMR studies during catalysis with dppe (see Supporting Information Figure S16) exhibited a single signal at δ=84.21 ppm, consistent with diamagnetic iron species with two ligands bound in equivalent environments. Furthermore, it was not possible to generate this complex with either ligand in the absence of electrophile, suggesting that the ‐CN group from the electrophile (7‐bromoheptanenitrile) might act as a ligand to iron, generating a coordinatively saturated unreactive species. Overall, these results are consistent with the major iron species formed during catalysis being an Fe(II) S=0 complex with two bisphosphine ligands and two electrophile molecules coordinating the iron through the cyanide group (e.g. [Fe(dppe)_2_(R‐CN)_2_]^2+^). This assignment is further supported by previous literature studies of a *trans*‐[Fe(depe)_2_(NCMe)_2_][BF_4_]_2_ complex (depe: 1,2‐Bis(diethylphosphino)ethane) with 80 K ^57^Fe Mössbauer parameters δ=0.27 mm/s |ΔE_Q_|=1.02 mm/s,^[57]^ consistent with those observed for the major iron species in catalysis in this study with both dppe and dpbz.

Overall, the studies of dppe and dpbz reveal a surprising origin of their poor catalytic performance. It is not rapid β‐H elimination and reduction but, instead, the formation of coordinatively saturated, Fe(II) complexes with two bisphosphine ligands coordinated that appear to be largely unreactive in catalysis. Note that using 1 equivalent of dppe does not lead to cross‐coupled product, consistent with the formation of the 2 : 1 ligand:Fe adduct even when only 1 equivalent of ligand is used (50 % of the 2 : 1 adduct forms in catalysis and no free phosphine is observed by ^31^P NMR, see Supporting Information Figure S17). Analogous 2 : 1 complexes were also observed for three‐component cross‐couplings with depe,^[27]^ supporting this hypothesis. Furthermore, changing the electrophile to 1‐bromoheptane to remove the cyano functionality does not lead to improved catalytic performance, indicating once again that the formation of coordinatively saturated 2 : 1 ligand:Fe complexes is the reason for poor reactivity (see Supporting Information Figure S18). By contrast, when using Xantphos the results from catalysis and the iron speciation were, as expected, the same for 1‐bromoheptane as with the original electrophile (~75 % yield, see Supporting Information Figure S19 and Table S2).

Taking this new definition of the molecular‐level origin of poor alkyl‐alkyl cross‐couplings with bisphosphines into account, we hypothesized that the use of a simple bisphosphine with increased steric bulk that preferentially forms 1 : 1 ligand:Fe complexes (analogous to Xantphos) combined with a simple alkyl electrophile lacking a coordinating functional group might facilitate alkyl‐alkyl cross‐coupling.

To test this hypothesis, we selected 1‐bromoheptane as electrophile and dcype (1,2‐Bis(dicyclohexylsphino)ethane) as supporting ligand, the latter previously shown to prefer formation of 1 : 1 ligand:Fe complexes.^[27]^ Alkyl‐alkyl cross‐coupling of 1‐bromoheptane and activated tributylborane ([^*n*^Bu_4_B][MgCl]), using 1 equivalent of dcype as supporting ligand led to consumption of 70 % electrophile, yielding 50 % of cross‐coupled product (undecane) and 20 % of side products (15 % heptane and 5 % 1‐heptene, similar results were obtained with 2 equivalents of ligand, see Supporting Information Table S2). The Mössbauer spectrum during catalysis is shown in Supporting Information Figure S20, where it can be seen that majority of all the iron in solution is in the form of the dihalide complex **1‐dcype**,^[27]^ with the remaining iron species being a dcype equivalent of the mono‐alkyl complex (**2‐dcype**, inferred based on the Mössbauer parameters δ=0.64 mm/s |ΔE_Q_|=2.30 mm/s) and a reduced species with similar parameters to the orange component from the catalysis with Xantphos (δ=0.41 mm/s |ΔE_Q_|=0.68 mm/s). Consistent with our hypothesis, all the species observed by Mössbauer with both 1 and 2 equivalents of dcype are four coordinate 1 : 1 ligand:Fe complexes, which enable successful reactivity and formation of cross‐coupled product in contrast to unreactive 2 : 1 complexes.

In terms of future ligand design, these ligand studies demonstrate that a key aspect of ligand effectiveness for alkyl‐alkyl cross‐coupling is the formation of 1 : 1 ligand:iron complexes, promoted either by the presence of wide bite angle ligands such as Xantphos or small bite angle bisphosphines with sufficient steric bulk (ex. dcype) to disfavor the formation of coordinatively saturated 2 : 1 complexes. Additional properties such as reduced ligand rigidity (ex. DPEphos) can be detrimental as these can result in alternative reduction pathways that are unproductive for catalysis, leading to ligand dissociation. While beyond the scope of the current study, future investigations of a broader set of ligands (including beyond bisphosphines) to evaluate donor atom effects, electronic variations, etc. should continue to broaden our molecular‐level understanding of ligand design for alkyl‐alkyl cross‐coupling with iron.

## Conclusion

In the present study, a combination of inorganic spectroscopies (80 K ^57^Fe Mössbauer and 10 K EPR), isolation and identification of iron intermediates by SC‐XRD, and reaction studies have allowed us to shed light into the reaction mechanism of an Iron‐Xantphos catalyzed alkyl‐alkyl Suzuki–Miyaura cross‐coupling, and identify the key features in the supporting ligand for a successful reaction. In this way, it was demonstrated that the reactivity and mechanism of this Iron‐Xantphos alkyl‐alkyl cross‐coupling system is dominated by Fe(II) alkylated intermediates and behaves very similarly to other iron cross‐coupling systems with aryl nucleophiles.[^25^, ^26^, ^27^, ^28^, ^29^] Here, the reaction mechanism is proposed to go through reaction of radical with monoalkylated iron(II) (**2**), to generate cross‐coupled product. Combined with additional studies defining correlations between ligand structure, iron speciation and catalytic performance, these mechanistic insights will facilitate the rational development of new ligand and pre‐catalysts for iron catalyzed alkyl‐alkyl cross‐coupling.

## Conflict of Interests

The authors declare no conflict of interest.

1

## Supporting information

As a service to our authors and readers, this journal provides supporting information supplied by the authors. Such materials are peer reviewed and may be re‐organized for online delivery, but are not copy‐edited or typeset. Technical support issues arising from supporting information (other than missing files) should be addressed to the authors.

Supporting Information

Supporting Information

Supporting Information

Supporting Information

Supporting Information

Supporting Information

Supporting Information

## Data Availability

The data that support the findings of this study are available from the corresponding author upon reasonable request.
