# Supplementary material for: Effective Alkyl‐Alkyl Cross‐Coupling with an Iron‐Xantphos Catalyst: Mechanistic and Structural Insights
Source: Angew Chem Int Ed Engl. 2024 Nov 2;64(1):e202413566. doi: 10.1002/anie.202413566 (PMC11701360; doi:10.1002/anie.202413566)
Supplement: Supplementary file 1 — Supporting Information [file ANIE-64-e202413566-s004.pdf]

Angewandte Chemie
Eine Zeitschrift der Gesellschaft Deutscher Chemiker

Supporting Information

Effective Alkyl-Alkyl Cross-Coupling with an Iron-Xantphos Catalyst: Mechanistic and Structural Insights

*M. Gimeno, M. C. Aguilera, V. E. Fleischauer, W. W. Brennessel, M. L. Neidig**

Supporting Information for

Effective Alkyl-Alkyl Cross-Coupling with an Iron-Xantphos Catalyst: Mechanistic and Structural Insights

Magali Gimeno, [a][b] Maria Camila Aguilera, [b] Valerie E. Fleischauer, [b] William W. Brennessel, [b] and Michael L. Neidig*[a]

Table of Contents

1. Experimental Procedures S3
   1.1 General Considerations S3
   1.2 Mössbauer Spectroscopy S3
   1.3 Electron Paramagnetic Resonance Spectroscopy (EPR) S3
   1.4 Preparation of **1-Br** (Fe(Xantphos)Br_2) S3
   1.5 Preparation of **2-CH_2SiMe_3** (Fe(Xantphos)(CH_2SiMe_3)Br S3
   1.6 Preparation of **2-Me** (Fe(Xantphos)(Me)Br) S3
   1.7 Preparation of **3-CH_2SiMe_3** (Fe(Xantphos)(CH_2SiMe_3)$_2$). S4
   1.8 Preparation of **4** Fe^{n}Bu(κ^{2}P:(η^6-C_6H_5)-Xantphos). S4
   1.9 Preparation of **5-Et** S4
   1.10 Suzuki-Miyaura Iron-Catalyzed Cross-Coupling S4
   1.11 Reaction of in situ generated **1** with nucleophile for ^{57}Fe Mössbauer analysis**.** S4
   1.12 Reaction of in situ generated **2** with 7-bromoheptanenitrile**.** S5
   1.13 Synthesis of ^{n}BuMgBr 1M in THF S5
   1.14 Reaction of in situ generated **1** with nucleophile at low temperature S5
2. Supplementary Mössbauer, NMR and Reactivity data S6
3. X-Ray Diffraction Data S17
   3.1 Fe(II)-Xantphos dibromide **(1-Br)** S17
   3.2 Monoalkylated Fe(II)-Xantphos **(2-CH_2SiMe_3)** S35
   3.3 Monoalkylated Fe(II)-Xantphos **(2-Me)** S59
   3.4 Bisalkylated Fe(II)-Xantphos **(3-CH_2SiMe_3)** S91
   3.5 Fe^{n}Bu(κ^{2}P:(η^6-C_6H_5)-Xantphos **(4)** S111
   3.6 Fe(0)-DPEphos **(5-Et)** S131

1. Experimental Procedures

1.1 General Considerations. All chemical reagents and solvents were purchased from commercial sources. ^{n}BuMgBr was prepared from commercial ^{n}BuBr and magnesium powder. All air and moisture sensitive manipulations were carried out in an MBraun inert-atmosphere (N_2) dry box equipped with a direct liquid nitrogen inlet line. All anhydrous solvents were further dried using activated alumina/4Å molecular sieves and stored under N_2-atmosphere over 4Å molecular sieves. $^{57}FeBr_2$ was synthesized following literature procedures starting from ^{57}Fe metal (95% enriched) purchased from Isoflex. Low-temperature reactions (0 °C) were performed in the glovebox using a Huber ministat 230-cc-NR recirculating bath combined with a Syrris hot/cold plate fit with a PT100 thermocouple for direct measurement and control of the reaction temperature. Additional infrastructure for low-temperature reactions and manipulations (less than - 25 °C) was provided by dry gloveboxes equipped with cold wells. Appropriate solvent-dry ice or -liquid N_2 formulations were utilized to achieve temperatures as low as -80 °C. ^{31}P NMR spectra were recorded at 400 MHz on a Bruker DPX-400 spectrometer (University of Rochester) and on a Bruker AVIII HD 400 (University of Oxford). GC-MS measurements were done in an Agilent 5977B GC/MSD (University of Oxford). EA measurements were done by the Elemental Analysis Service at the London Metropolitan University.

1.2 Mössbauer Spectroscopy. All samples were prepared under nitrogen atmosphere in a glovebox equipped with a liquid nitrogen fill port to enable sample freezing at 77 K within the glovebox. Samples were loaded in Derlin Mössbauer sample cups, and subsequently frozen in liquid N_2. Zero field 80 K Mössbauer measurements were performed using a SeeCo. MS4 Mössbauer spectrometer integrated with a Janis SVT-400 T He/N_2 cryostat. Isomer shifts reported were determined relative to α-Fe at 298 K. In addition, reported ΔE_Q parameters for zero field correspond to absolute values. All Mössbauer spectra were fit using the program WMoss (SeeCo). Freezed trapped solution Mössbauer samples were prepared utilizing $^{57}FeBr_2$. Experiments were carried out under catalytically relevant conditions as reported for the method (Fe concentration, temperature, and solvent). Solid Mössbauer samples were prepared by isolating crystalline material obtained from crystallization procedures performed starting from $^{57}FeBr_2$.

1.3 Electron Paramagnetic Resonance Spectroscopy (EPR). Samples for EPR spectroscopy were prepared in an inert atmosphere glove box equipped with a liquid nitrogen fill port to enable sample freezing to 77 K within the glove box. EPR samples were prepared in 4 mm OD suprasil quartz EPR tubes from Wilmad Labglass. Samples for spin integration utilized high precision suprasil quartz tubes to allow for direct comparison of intensities between different samples. X-band EPR spectra were recorded on a Bruker EMXplus spectrometer equipped with a 4119HS cavity and an Oxford ESR-900 helium flow cryostat. The instrumental parameters employed for all samples were as follows: 0.0012 mW power; time constant 0.01 ms; modulation amplitude 1 G; 9.38 GHz; modulation frequency 100 kHz. Samples exhibiting S = 1/2 EPR spectra were spin integrated using an 10 mM $CuSO_4$ standard under non-saturating conditions. Identical instrumentation parameters were used for both the iron and standard samples. Reactions were performed at catalytic relevant conditions (10 mM of iron concentration).

1.4 Preparation of 1-Br (Fe(Xantphos)Br_2). Fe(Xantphos)Br_2 was prepared in an analogous way to **1-Cl**. To a round bottom flask was added $FeBr_2$ (0.155 g, 0.7 mmol) and Xantphos (0.415 g, 0.7 mmol) followed by THF (20 mL). The solids were stirred at 60 °C for 4 h followed by removal of solvents *in vacuo* at room temperature. The pale green residues were then washed with toluene and collected after filtration. Colorless crystals, corresponding with **1-Br** were formed from dissolution of the solids in a minimal volume of THF and slow evaporation at room temperature. Elemental Analysis: Expected C, 59.61 %; H, 4.65 %; Found C, 59.53 %; H, 4.05 %.

1.5 Preparation of 2-CH_2SiMe_3 (Fe(Xantphos)(CH_2SiMe_3)Br). A 20 mL scintillation vial was charged with 2.2 mg of $FeBr_2$, 5.7 mg of Xantphos and 1 mL of diethylether. A white slurry formed was stirred for 1h at RT. 1 equivalent of (trimethylsilyl)methyl magnesium chloride (1 M in diethyl ether) was added dropwise to the slurry at RT. Slurry slowly become a pale-yellow solution over the course of 15 min at RT. Subsequently, reaction mixture was placed at -80 °C. After 30 min, solution is filtered through celite (cold) and placed it back to RT. This mixture was transferred to a 4 mL vial and allowed to crystallize by vapor diffusion with pentane at -10 °C, resulting in colorless needles suitable for X-ray crystallography after 1 week.

1.6 Preparation of 2-Me (Fe(Xantphos)(Me)Br). A 20 mL scintillation vial was charged with 2.1 mg of $FeBr_2$, 5.5 mg of Xantphos and 1 mL of THF. The colorless solution was stirred for 10 minutes at room temperature, and subsequently placed at -50 °C. After 20 minutes, 1 equivalent of methylmagnesium bromide (1M in THF) was added dropwise to the solution. The solution turned yellow. After reacting for 15 minutes, 1 mL of THF was added and the

reaction mixture was filtered through celite (cold) and stored at -80 °C for a week. Then, magnesium salts were filtered through celite (cold) and the sample was stored again at -80 °C resulting in colorless needles suitable for X-ray crystallography after 1 week.

1.7 Preparation of 3-CH_2SiMe_3 (Fe(Xantphos)(CH_2SiMe_3)$_2$). A 20 mL scintillation vial was charged with 4.3 mg of $FeBr_2$, 10.4 mg of Xantphos and 1 mL of diethylether. A white slurry formed was stirred for 1h at RT. 2 equivalents of (trimethylsilyl)methyl magnesium (1 M in diethyl ether) chloride were added dropwise to the slurry at RT. Slurry slowly become a bright yellow solution over the course of 30 min at RT. This mixture was filtered through celite and transferred to a 4 mL vial and allowed to crystallize by vapor diffusion with pentane at -10 °C, resulting in yellow plates suitable for X-ray crystallography after 1 week.

1.8 Preparation of 4 $Fe^{n}Bu(\kappa^2P:(\eta^6\text{-}C_6H_5)$-Xantphos). To a 20 mL scintillation vial was added $FeBr_2$(Xantphos) (10 mg, 12.6 mmol) and THF (ca. 6 mL). The solution was stirred at 0 °C for 20 min until a fine slurry was present. To the cold slurry was added 25.6 µL (0.5 M, 12.6 mmol) of ^{n}BuMgBr in THF. After stirring for 20 min, cold pentane (-30 °C, ca. 2 mL) was added, and the solution was placed at -74 °C and stirred for an additional 20 min before filtering through a Celite plug. The solution was stored at -30 °C for 2 weeks after which orange single crystals suitable for X-ray diffraction were obtained.

1.9 Preparation of 5-Et. A 20 mL scintillation vial was charged with 3.5 mg of $FeBr_2$, 13 mg of DPEphos and 1 mL of THF. Colorless solution was formed and stirred for 30 min at RT. 2 equivalents of EtMgBr (1 M in THF) were added dropwise to the colorless solution at RT, observing a rapid color change to dark orange. THF was removed under vacuum, dark residues were redissolved in 3.5 mL of hexane yielding a bright orange solution with dark precipitates. Hexane solution was finally filtered through a celite plug. Orange needles of **5-Et** suitable for X-ray crystallography crystallized from hexane solution overnight at RT.

****Handling of temperature sensitive crystals of 2-CH_2SiMe_3, 2-Me, 3-CH_2SiMe_3 and 4 for X-ray diffraction analysis.** Manipulations were carried out inside of a N_2 purged glovebag (under constant positive N_2 pressure). 20 mL scintillation vials with crystalline material were stored in an aluminum pie-block containing dry ice. A specialized aluminum block with a hollow internal chamber, equipped with an inlet and outlet nozzle for passage of liquid N_2 was utilized to maintain integrity of crystalline samples. Microscope slides coated with SilOil were pre-cooled on the cold aluminum block, upon which an aliquot of cold sample solution was pipetted. Crystalline specimens for X-ray diffraction were examined microscopically. High quality single crystals were removed from the glass slide with a goniometer pin and transported to the diffractometer (100 K mounting temperature) while being held over a hand dewar containing liquid N_2.

1.10 Suzuki-Miyaura Iron-Catalyzed Cross-coupling. All catalytic reactions were performed following an adaptation of the procedure reported by Nakamura.[1] To a THF solution (1.0 mL) of $^{n}Bu_3B$ (218 mg, 1.2 mmol, 60 equivalents respect to Fe) in a 20 mL scintillation vial was added Grignard reagent ^{n}BuMgCl (1.1 mmol, 2M in THF) at 0 °C. After stirring for 30 min, the reaction mixture was allowed to warm to 25 °C. After stirring for 1 h, the solvent was removed in vacuo at 25 °C. The residual borate was dissolved in 0.5 mL THF and added dropwise to 20 mL scintillation vial charged with a solution of $FeBr_2$ (4.3 mg, 0.02 mmol), Xantphos (23.1 mg, 0.04 mmol), 7-bromoheptanenitrile (114.0 mg, 90.0 mL, 0.6 mmol) and dodecane (102.2 mg, 136.0 mL, 0.6 mmol) in 1.5 mL THF at RT. The coupling reaction was carried out at 25 °C for 6 h. A 1 mL aliquot of the reaction was quenched with 0.2 mL aqueous 1M HCl solution and the solution was taken to a final volume of 20 mL by adding THF and stirring for 5 minutes. The reaction mixture was filtered through a Florisil pad and then an aliquot of 0.4 mL was taken to a final volume of 1 mL. Product yields and recovery of 1-bromoheptanenitrile were determined by GC-MS analysis of the crude product using dodecane as an internal standard. The same procedure was followed when using the alternative bisphosphines DPEphos, dppe, dpbz and dcype, as well as when the electrophile 1-Bromoheptane was used.

1.11 Reaction of in situ generated ^{57}Fe(Xantphos)Br_2 with nucleophile ([$^{n}Bu_4B$][MgCl]) for ^{57}Fe Mössbauer analysis. As an example procedure, the reaction of Fe(Xantphos)Br_2 with 5.0 equivalents of [$^{n}Bu_4B$][MgCl], is described. To a THF solution (0.5 mL) of nBu$_3$B (18.2 mg, 0.1 mmol) in a 20 mL scintillation vial was added Grignard reagent ^{n}BuMgCl (0.09 mmol, 2M in THF) at 0 °C. After stirring for 30 min, the reaction mixture was allowed to warm to 25 °C. After stirring for 1 h, the solvent was removed in vacuo at 25 °C. The residual borate was dissolved in 0.5 mL THF and added dropwise to 20 mL scintillation vial charged with a solution of $^{57}FeBr_2$ (4.3 mg, 0.02 mmol) and Xantphos (23.1 mg, 0.04 mmol) in 1.5 mL THF at RT. The reaction was stirred for 30 s at RT after addition of Grignard reagent

before freeze-trapping a ca. 0.5 mL aliquot inside of a Delrin Mössbauer cup by submerging in liquid N_2. Samples at later time points were prepared in the same way. The same procedure was followed for the corresponding experiments with the alternative bisphosphines and the different equivalents of activated borate.

1.12 Reaction of in situ generated iron-Xantphos monoalkylated species with 7-bromoheptanenitrile for spectroscopic and reactivity studies. As an example of the general procedure employed, the reaction of Fe(Xantphos)Br_2 with 5.0 equivalents of [^{n}Bu_4B][MgCl], with 30 equivalents of 7-bromoheptanenitrile is described. In a a 20 mL scintillation vial, 5.0 equivalents of [^{n}Bu_4B][MgCl] were activated as per the procedure described in 1.11. In a separate 20 mL scintillation vial, ^{57}FeBr_2 (4.3 mg, 0.02 mmol) and Xantphos (23.1 mg, 0.04 mmol) were dissolved in 1.5 mL THF at RT. The residual borate was dissolved in 0.5 mL THF and added dropwise to the ^{57}Fe(Xantphos)Br_2 solution. The reaction was stirred at RT for 10 minutes and a control sample was taken by freeze-trapping a ca. 0.5 mL aliquot inside of a Delrin Mössbauer cup by submerging in liquid N_2, followed by addition of 7-Bromoheptanenitrile (114.0 mg, 90.0 mL, 0.6 mmol) and dodecane (102.2 mg, 136.0 mL, 0.6 mmol). After the addition of electrophile, freeze-trapped samples were taken at selected time points for Mössbauer analysis. Samples for GC-MS analysis were prepared as described in 1.10.

1.13 Synthesis of ^{n}BuMgBr 1M in THF. Inside an MBraun inert-atmosphere (N_2) dry box, Magnesium powder (1.0 g, 0.04 mol), one small I_2 crystal and 10 mL of THF were charged into an oven-dry 250 mL RB flask equipped with a Teflon stir bar, sealed with a rubber septum. The suspension was set to stir at room temperature until the I_2 dissolved, leading to a brown suspension. A 20 mL scintillation vial was charged with ^{n}BuBr (2.74 g, 2.15 mL, 0.02 mol) and 10 mL THF and the resulting solution was added dropwise to the magnesium suspension though the rubber septum. The reaction mixture was stirred for 3 hours at RT, followed by 3 hours at 60 °C. Thereafter, the reaction mixture was left to cool down to RT and was filtered through a 30 mL medium frit to discard the unreactive magnesium. The resulting ^{n}BuMgBr was titrated with salicylaldehyde phenylhydrazone and stored in the glovebox inside the -30 °C freezer.

1.14 Reaction of in situ generated ^{57}Fe(Xantphos)Br_2 with nucleophile (^{n}BuMgBr) at low temperature for ^{57}Fe Mössbauer analysis. As an example procedure, the reaction of Fe(Xantphos)Br_2 with 5.0 equivalents of ^{n}BuMgBr, is described. A 20 mL scintillation vial was charged with 4.3 mg of ^{57}FeBr_2, 2 equivalents of Xantphos (23.1 mg) and 2 mL of THF. The solution was stirred at RT for 20 minutes to form ^{57}Fe(Xantphos)Br_2 in situ. Next, the vial was placed at -70 °C for 10 minutes and 5 equivalents of ^{n}BuMgBr (100 μL, 0.1 mmol, 1M in THF) were added dropwise. The reaction was stirred for 5 minutes at -70 °C after addition of Grignard reagent before freeze-trapping a ca. 0.5 mL aliquot inside of a Delrin Mössbauer cup by submerging in liquid N_2. Subsequently, the reaction mixture was let to slowly warm up to room temperature, taking Mössbauer samples at selected temperatures and time points.

2. Supplementary Data

Table S1. Iron-catalyzed Suzuki-Miyaura cross-coupling of 7-bromoheptanenitrile with tetrabutylborane.[a]

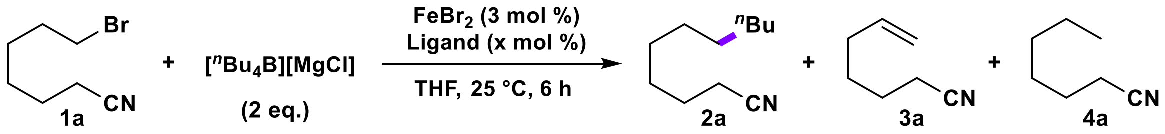

Entry	Ligand	Yield **2a**	Yield **3a**	Yield **4a**	Recovery **1a**
1	Xantphos (6 mol%)	80 %	2 %	12 %	5 %
2	Xantphos (3 mol%)	65 %	-	9 %	25 %
3	DPEphos (6 mol %)	6 %	7 %	21%	66 %
4	-	5 %	8 %	20 %	65 %
5	dppe (6 mol%)	-	-	-	99 %
6	dppe (3 mol%)	-	-	9 %	90 %
7	dpbz (6 mol%)	-	-	-	99 %
8	dpbz (3 mol%)	-	-	2 %	97 %
9	Xantphos (6 mol%) in 2Me-THF	75%	-	23 %	1%

[a] All yields determined by GC-MS analysis.

Table S2. Iron-catalyzed Suzuki-Miyaura cross-coupling of 1-bromoheptane with tetrabutylborane. [a]

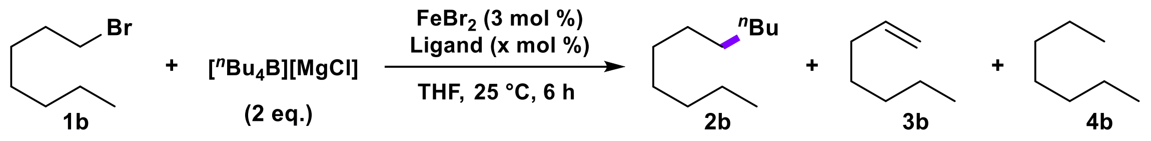

Entry	Ligand	Yield **2b**	Yield **3b**	Yield **4b**	Recovery **1b**
1	Xantphos (6 mol%)	75 %	2 %	16 %	6 %
2	dcype (6 mol%)	35 %	2 %	12 %	47 %
3	dcype (3 mol %)	49 %	4 %	14 %	27 %
4	dppe (6 mol%)	-	-	-	96 %
5	dppe (3 mol%)	-	4 %	20 %	75 %
6	dpbz (6 mol%)	-	-	-	92 %
7	dpbz (3 mol%)	-	6 %	17 %	70 %

[a] All yields determined by GC-MS analysis.

Table S3. ^{57}Fe Mössbauer parameters for Fe(II)-bisphosphine complexes as solid samples.[25–27] SciOPP: 1,2-Bis[bis[3,5-di(t-butyl)phenyl]phosphino]-benzene; dcype: 1,2-bis(dicyclohexylphosphino)ethane

Category	Complex	δ (mm/s)	$|\Delta E_Q|$ (mm/s)
Dihalide	$Fe(SciOPP)Cl_2$	0.73	2.54
	$Fe(dcype)Br_2$	0.5	3.13
Mono-aryl	Fe(SciOPP)(Mes)Br	0.52	1.97
	$Fe(dcype)(3\text{-}MeOC_6H_4)Br$	0.52	2.45
Bis-aryl	$Fe(SciOPP)(Mes)_2$	0.29	3.58
	$Fe(dcype)(3\text{-}MeOC_6H_4)_2$	0.23	4.22

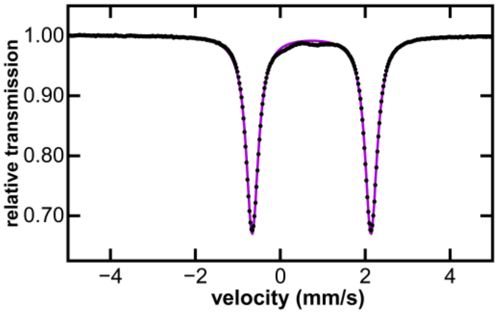

Figure S1. 80 K ^{57}Fe Mössbauer spectra of isolated crystals of $Fe(Xantphos)Br_2$ (**1-Br**), with Mössbauer parameters of δ = 0.74 mm/s $|\Delta E_Q|$ = 2.72 mm/s.

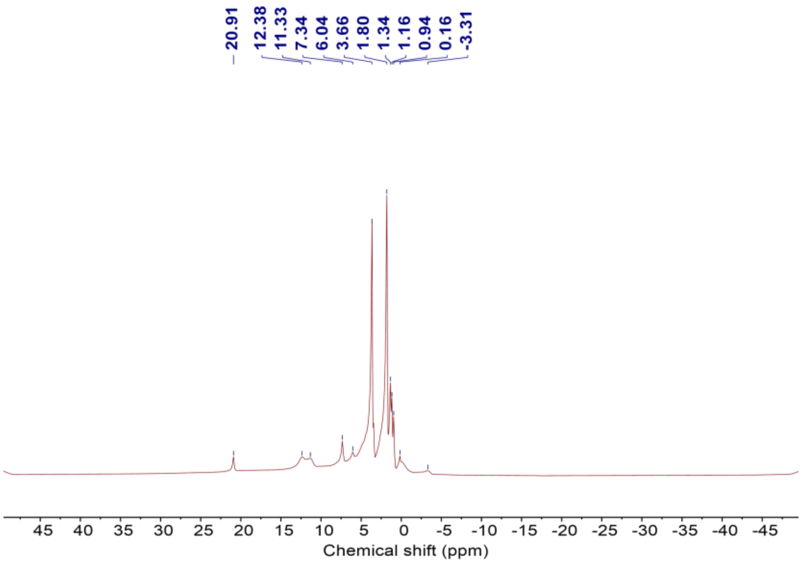

Figure S2. ^{1}H NMR spectrum of $Fe(Xantphos)Br_2$ (**1-Br**) in THF-d_8.

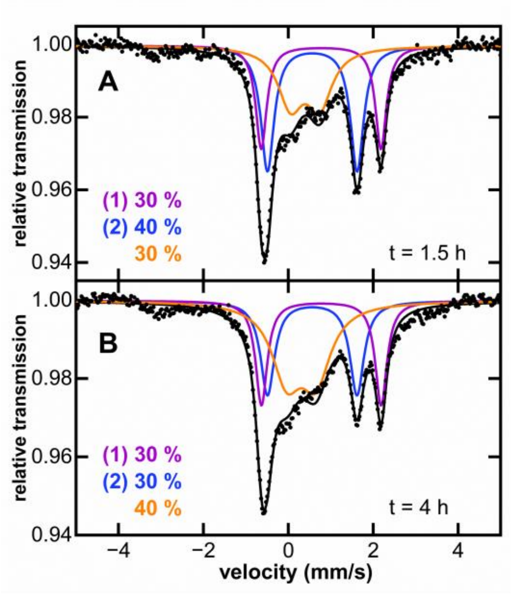

Figure S3. 80 K ^{57}Fe Mössbauer spectrum at 1.5 h and 4 h in catalysis, evidencing iron species that grows over time. Mössbauer parameters for purple component (**1**) δ = 0.78 mm/s $|\Delta E_Q|$ = 2.76 mm/s, blue component (**2**) δ = 0.55 mm/s $|\Delta E_Q|$ = 2.05 mm/s and orange component δ = 0.41 mm/s $|\Delta E_Q|$ = 0.64 mm/s

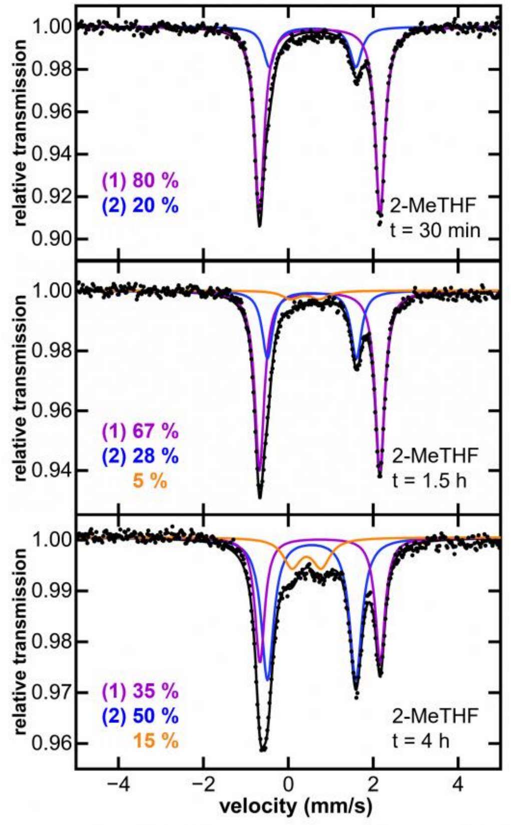

Figure S4. 80 K ^{57}Fe Mössbauer spectrum at 30 min, 1.5 h and 4 h in catalysis in 2-MeTHF. Mössbauer parameters for purple component (**1**) δ = 0.78 mm/s $|\Delta E_Q|$ = 2.76 mm/s, blue component (**2**) δ = 0.55 mm/s $|\Delta E_Q|$ = 2.05 mm/s and orange component δ = 0.41 mm/s $|\Delta E_Q|$ = 0.64 mm/s

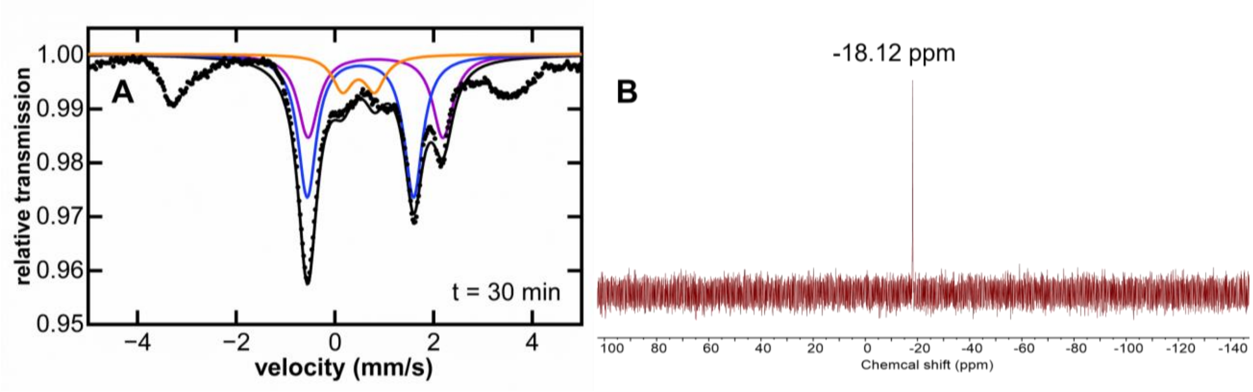

Figure S5. A) 80 K ^{57}Fe Mössbauer spectra at 30 minutes in catalysis in THF using only 1 equivalent of Xantphos. Evidence of 6-line pattern species which correlates to reduced Fe particles corresponding to observed black powder in solution. Mössbauer parameters for purple component (**1**) δ= 0.78 mm/s $|\Delta E_Q|$ = 2.76 mm/s, blue component (**2**) δ= 0.55 mm/s $|\Delta E_Q|$ = 2.05 mm/s and orange component δ= 0.41 mm/s $|\Delta E_Q|$ = 0.64 mm/s. B) ^{31}P NMR shows signal for free Xantphos in solution at -18.12 ppm.

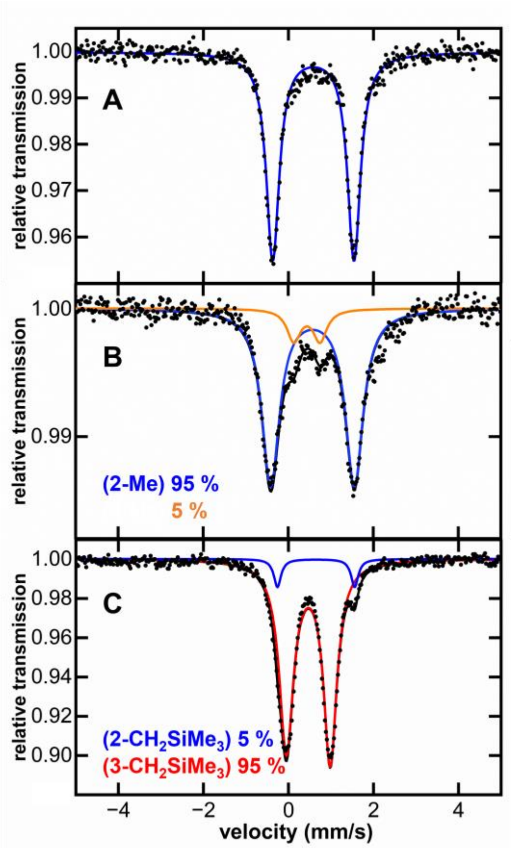

Figure S6. 80 K ^{57}Fe Mössbauer spectra of solid samples from isolated crystalline material. A) **2-CH$_2$SiMe$_3$** Mössbauer parameters δ = 0.59 mm/s and $|\Delta E_Q|$ = 1.91 mm/s (blue component). B) **2-Me** δ = 0.57 mm/s and $|\Delta E_Q|$ = 1.96 mm/s (blue component) and orange component δ= 0.41 mm/s $|\Delta E_Q|$ = 0.64 mm/s. C) **3-CH$_2$SiMe$_3$** δ= 0.44 mm/s $|\Delta E_Q|$ = 1.03 mm/s (red component).

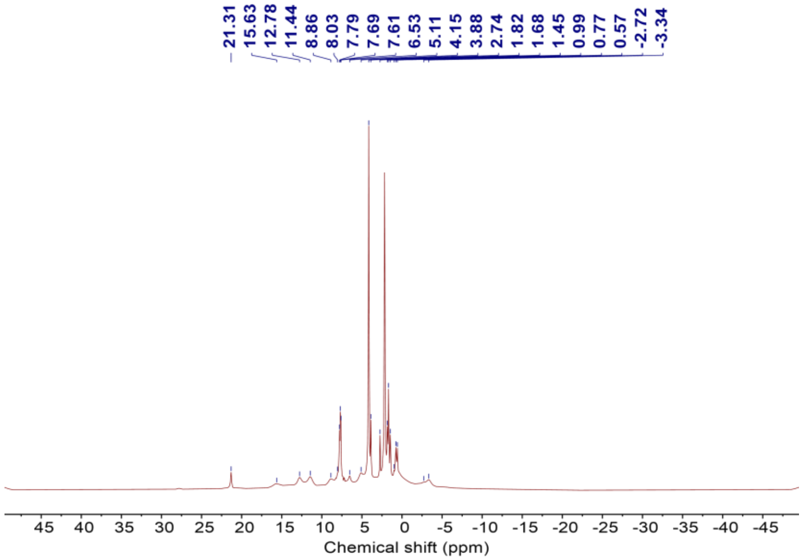

Figure S7. ^{1}H NMR spectrum of Fe(Xantphos)(CH_2SiMe_3)Br (**2-CH_2SiMe_3**) in THF-d_8.

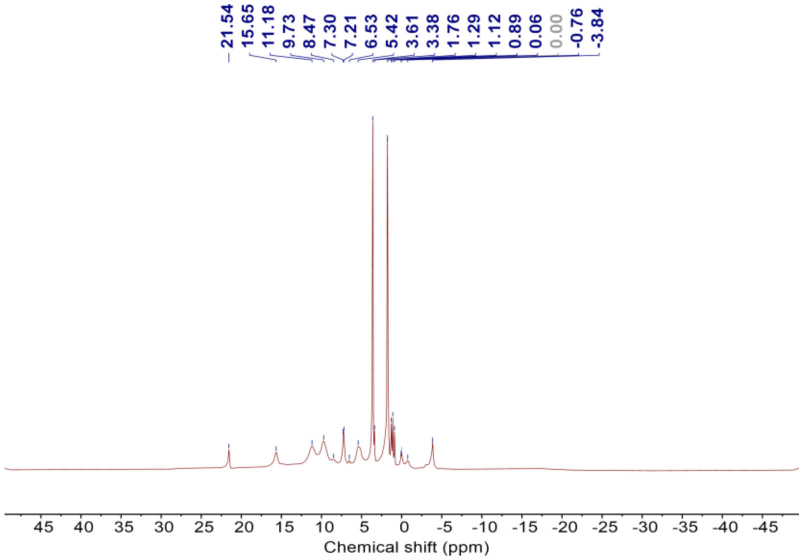

Figure S8. ^{1}H NMR spectrum of Fe(Xantphos)(CH_2SiMe_3)$_2$ (**3-CH_2SiMe_3**) in THF-d_8.

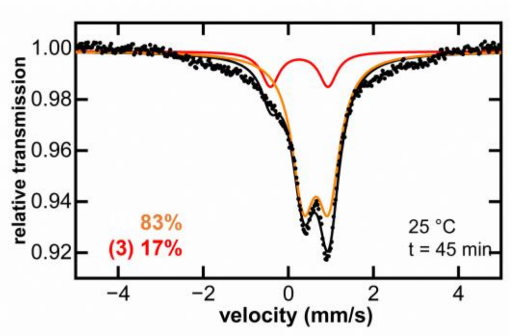

Figure S9. 80 K ^{57}Fe Mössbauer spectra of reaction of Fe(Xantphos)Br_2 with 5 equivalents of ^{n}BuMgBr at -70 °C when is let to warm up to room temperature. Mössbauer parameters for orange component δ = 0.41 mm/s $|\Delta E_Q|$ = 0.64 mm/s and red component (**3**) δ= 0.25 mm/s $|\Delta E_Q|$ = 1.33 mm/s.

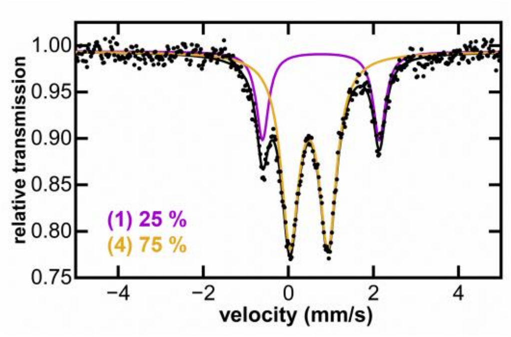

Figure S10. 80 K ^{57}Fe Mössbauer spectrum of isolated material of complex **4**. Mössbauer parameters of δ=0.47 mm/s $|\Delta E_Q|$ = 0.86 mm/s (gold component), purple component corresponds to complex **1-Br** that co-crystallizes with **4.** Reactivity experiments tracked by 10 K EPR and GC-MS showed no consumption of electrophile 7-bromoheptanenitrile and no reactivity of **4**.

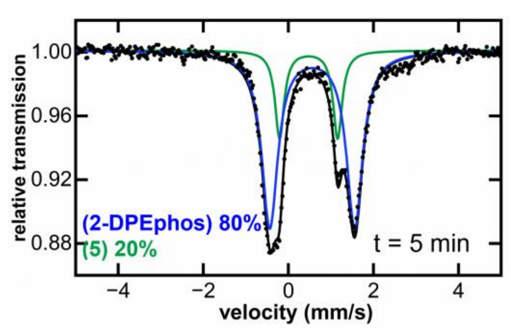

Figure S11. 80 K solid ^{57}Fe Mössbauer spectra of the reaction of $FeBr_2$ with 1 equivalent of ^{n}BuMgBr in the presence of 2 equivalents of DPEphos at -65 °C. Mössbauer parameters for blue component (**2-DPEphos**) δ =0.55 mm/s and $|\Delta E_Q|$ = 2.00 mm/s, parameters for green component (**5**) δ= 0.46 mm/s $|\Delta E_Q|$ = 1.36 mm/s.

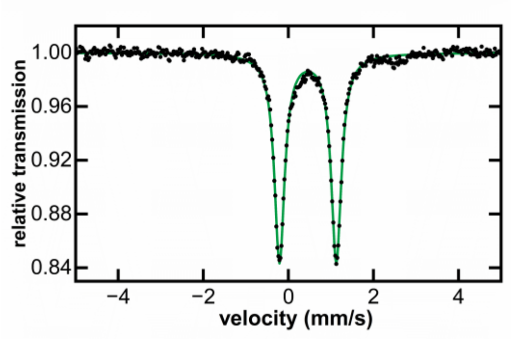

Figure S12. 80 K solid ^{57}Fe Mössbauer spectra of isolated crystalline material of complex **5-Et**. Mössbauer parameters δ= 0.45 mm/s $|\Delta E_Q|$ = 1.33 mm/s (green component).

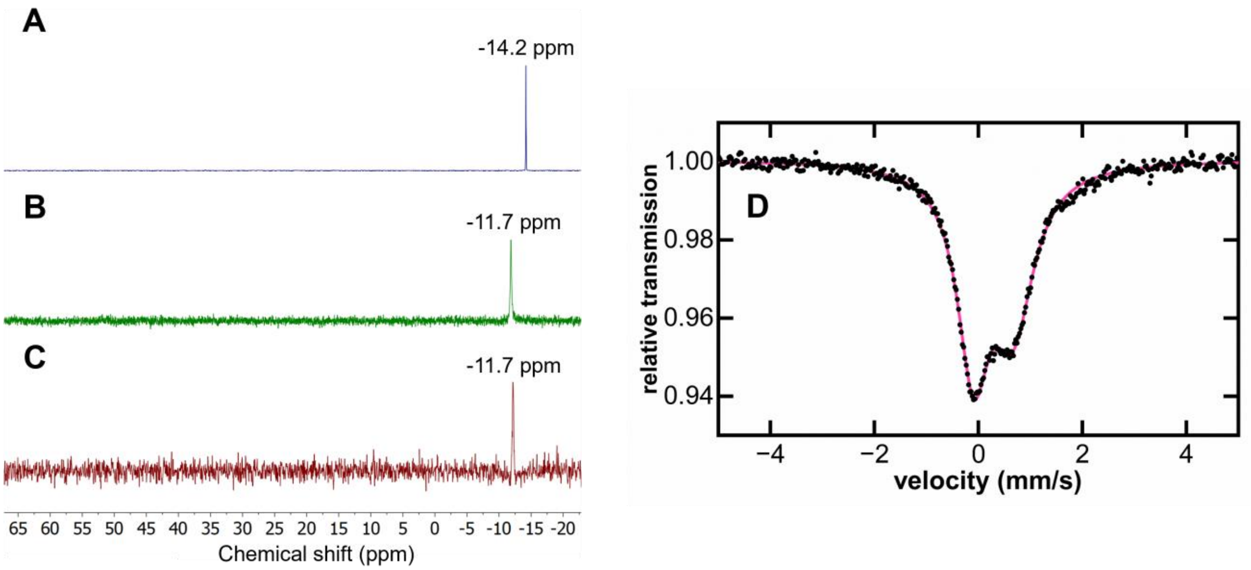

Figure S13. ^{31}P NMR analysis of (**A**) DPEphos in THF (**B**) catalytic reaction solution performed using 2 equivalents of DPEphos at 2.5 h (**C**) catalytic reaction solution performed using 1 equivalent of DPEphos at 2.5 h. (**D**) Freeze-trapped 80 K ^{57}Fe Mössbauer spectra of catalytic reaction solution at 2.5 h performed without any ligand, only employing $FeBr_2$, *n*-butylborane and 7-bromoheptanitrile in THF at RT. Mössbauer parameters for pink component δ = 0.28 mm/s $|\Delta E_Q|$ = 0.76 mm/s.

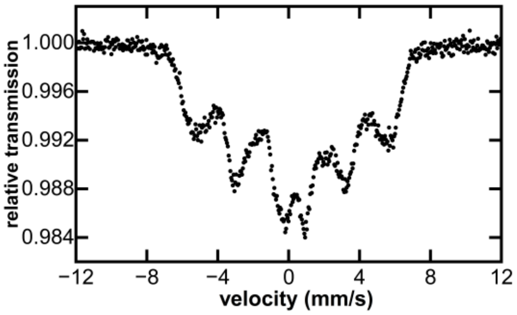

Figure S14. Freeze-trapped 5 K ^{57}Fe Mössbauer spectrum of the iron speciation during catalysis employing DPEphos at 2.5 h. This spectrum corresponds to the same solution sample of Figure 8B where we observed an asymmetric doublet at 80 K with Mössbauer parameters of δ = 0.28 mm/s |ΔE_Q| = 0.76 mm/s.

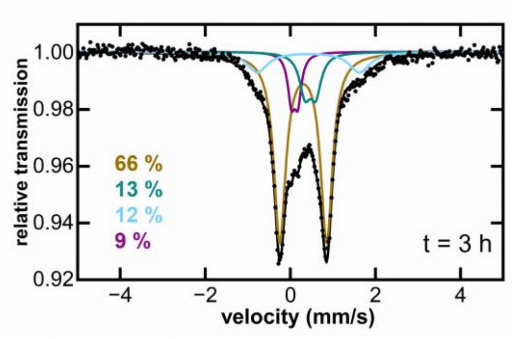

Figure S15. 80 K ^{57}Fe Mössbauer spectra of iron speciation during catalysis with 2 equivalents of dpbz after 3 hours. Mössbauer parameters δ= 0.30 mm/s |ΔE_Q| = 1.10 mm/s for dark yellow component, δ= 0.47 mm/s |ΔE_Q| = 0.25 mm/s for teal component, δ= 0.42 mm/s |ΔE_Q| = 2.40 mm/s for light blue component and δ= 0.10 mm/s |ΔE_Q| = 0.15 mm/s for dark purple component.

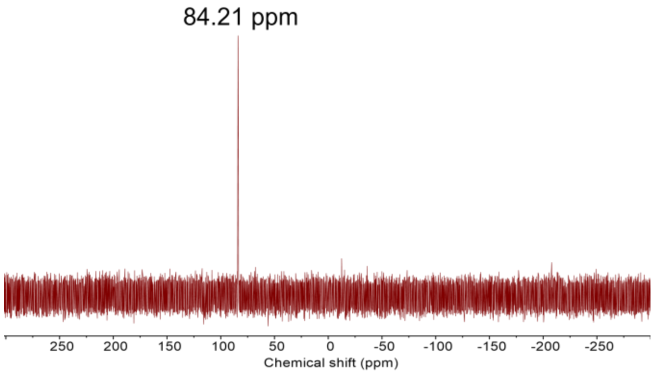

Figure S16. ^{31}P NMR analysis of catalysis with 2 equivalents of dppe after 3 hours.

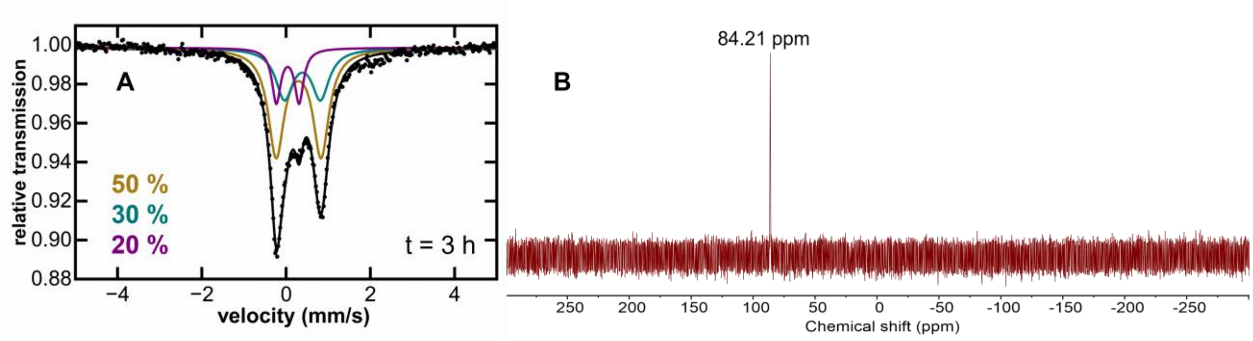

Figure S17. A) 80 K ^{57}Fe Mössbauer spectra of iron speciation during catalysis with 1 equivalent of dppe after 3 hours. Mössbauer parameters δ= 0.25 mm/s $|\Delta E_Q|$ = 0.99 mm/s for dark yellow component, δ= 0.38 mm/s $|\Delta E_Q|$ = 0.85 mm/s for teal component and δ= 0.03 mm/s $|\Delta E_Q|$ = 0.54 mm/s for dark purple component. B) ^{31}P NMR analysis of catalysis with 1 equivalent of dppe after 3 hours.

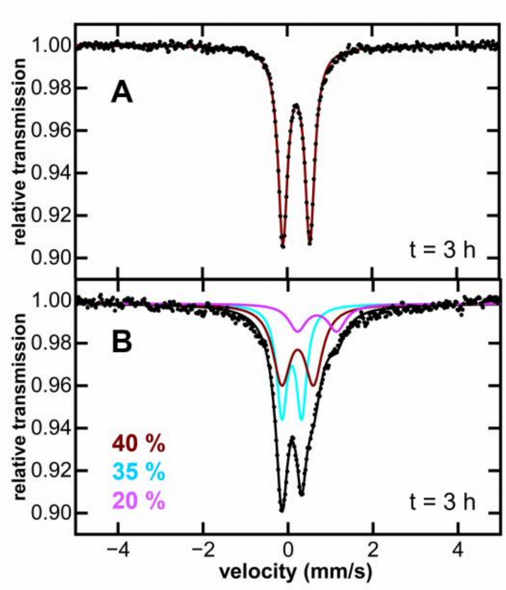

Figure S18. 80 K ^{57}Fe Mössbauer spectra of iron speciation during catalysis using 1-bromoheptane as electrophile with A) 2 equivalents of dppe and B) 1 equivalent of dppe after 3 hours. Mössbauer parameters δ= 0.21 mm/s $|\Delta E_Q|$ = 0.64 mm/s for maroon component, δ= 0.08 mm/s $|\Delta E_Q|$ = 0.46 mm/s for cyan component and δ= 0.68 mm/s $|\Delta E_Q|$ = 0.92 mm/s for pink component.

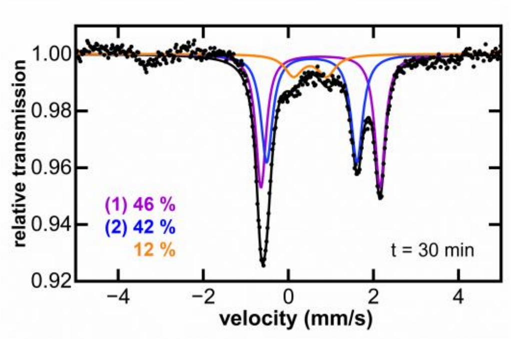

Figure S19. 80 K ^{57}Fe Mössbauer spectrum at 30 minutes in catalysis using 1-bromoheptane with 2 equivalents of Xantphos. Mössbauer parameters for purple component (**1**) δ= 0.78 mm/s $|\Delta E_Q|$ = 2.76 mm/s, blue component (**2**) δ= 0.55 mm/s $|\Delta E_Q|$ = 2.05 mm/s and orange component δ= 0.41 mm/s |ΔEQ| = 0.64 mm/s

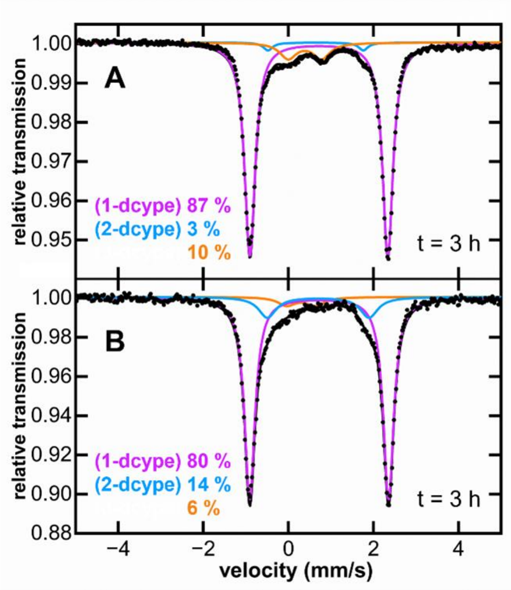

Figure S20. 80 K ^{57}Fe Mössbauer spectrum at 3 hours in catalysis using 1-bromoheptane with A) 2 equivalents of dcype and B) 1 equivalent of dcype. Mössbauer parameters for pink component (**1-dcype**) δ= 0.72 mm/s $|\Delta E_Q|$ = 3.21 mm/s, ligth blue component (**2-dcype**) δ= 0.64 mm/s $|\Delta E_Q|$ = 2.30 mm/s and orange component δ= 0.41 mm/s $|\Delta E_Q|$ = 0.68 mm/s

3. X-Ray Diffraction Data

3.1 Fe(II)-Xantphos dibromide (1-Br)

CRYSTAL STRUCTURE REPORT

C_{43} H_{40} Br_2 Fe O_2 P_2

or

(Xantphos)$FeBr_2$ · THF

Report prepared for:

V. Fleischauer, Prof. M. Neidig

December 28, 2017

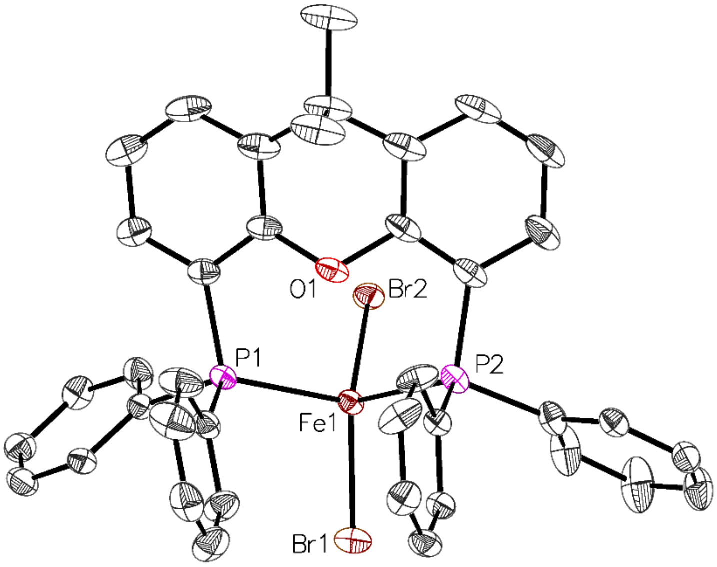

William W. Brennessel

X-ray Crystallographic Facility

Department of Chemistry, University of Rochester

120 Trustee Road

Rochester, NY 14627

Data collection

A crystal ($0.17 \times 0.14 \times 0.11$ mm^3) was placed onto a thin glass optical fiber or a nylon mesh and mounted on a XtaLab Synergy-S Dualflex diffractometer equipped with a HyPix-6000HE HPC area detector for data collection at 100.01(10) K. A preliminary set of cell constants and an orientation matrix were calculated from a small sampling of reflections.[1] A short pre-experiment was run, from which an optimal data collection strategy was determined. The full data collection was carried out using a PhotonJet (Cu) X-ray Source with frame times ranging from 0.18-0.72 seconds and a detector distance of 31.2 mm. Series of frames were collected in 0.50º steps in ω at different 2θ, κ, and ϕ settings. After the intensity data were corrected for absorption, the final cell constants were calculated from the xyz centroids of 19583 strong reflections from the actual data collection after integration.[1] See Table 1 for additional crystal and refinement information.

Structure solution and refinement

The structure was solved using XT[2] and refined using XL.[3] The space group *P*-1 was determined based on intensity statistics. Most or all non-hydrogen atoms were assigned from the solution. Full-matrix least squares / difference Fourier cycles were performed which located any remaining non-hydrogen atoms. All non-hydrogen atoms were refined with anisotropic displacement parameters. All hydrogen atoms were placed in ideal positions and refined as riding atoms with relative isotropic displacement parameters. The final full matrix least squares refinement converged to $R1 = 0.0305$ (F^2, $I > 2\sigma(I)$) and $wR2 = 0.0829$ (F^2, all data).

Structure description

The structure is the one suggested. The asymmetric unit contains one iron complex in a general position and one half each of two cocrystallized THF solvent molecules located at crystallographic inversion centers. The solvent molecules are modeled as disordered over the centers (0.50:0.50).

Structure manipulation and figure generation were performed using Olex2.[4] Unless noted otherwise all structural diagrams containing thermal displacement ellipsoids are drawn at the 50 % probability level.

Data collection, structure solution, and structure refinement were conducted at the X-ray Crystallographic Facility, B04 Hutchison Hall, Department of Chemistry, University of Rochester. All publications arising from this report MUST either 1) include William W. Brennessel as a coauthor or 2) acknowledge William W. Brennessel and the X-ray Crystallographic Facility of the Department of Chemistry at the University of Rochester.

[1] *CrysAlisPro*, version 171.39.35c; Rigaku Corporation: Oxford, UK, 2017.

[2] Sheldrick, G. M. *SHELXT*, version 2014/5; *Acta. Cryst.* **2015**, A*71*, 3-8.

[3] Sheldrick, G. M. *SHELXL*, version 2018/1; *Acta. Cryst.* **2015**, C*71*, 3-8.

[4] Dolomanov, O. V.; Bourhis, L. J.; Gildea, R. J.; Howard, J. A. K.; Puschmann, H. *Olex2*, version 1.2-ac3; *J. Appl. Cryst.* **2009**, *42*, 339-341.

Some equations of interest:

$$R_{int} = \Sigma\, |F_o^2 - \langle F_o^2 \rangle| \,/\, \Sigma |F_o^2|$$

$$R1 = \Sigma ||F_o| - |F_c|| \,/\, \Sigma |F_o|$$

$$wR2 = [\Sigma[w(F_o^2 - F_c^2)^2] \,/\, \Sigma\, [w(F_o^2)^2]]^{1/2}$$

where $w = 1 / [\sigma^2 (F_o^2) + (aP)^2 + bP]$ and

$$P = 1/3 \max (0, F_o^2) + 2/3\, F_c^2$$

$$\mathrm{GOF} = S = [\Sigma[w(F_o^2 - F_c^2)^2] \,/\, (m-n)]^{1/2}$$

where m = number of reflections and n = number of parameters

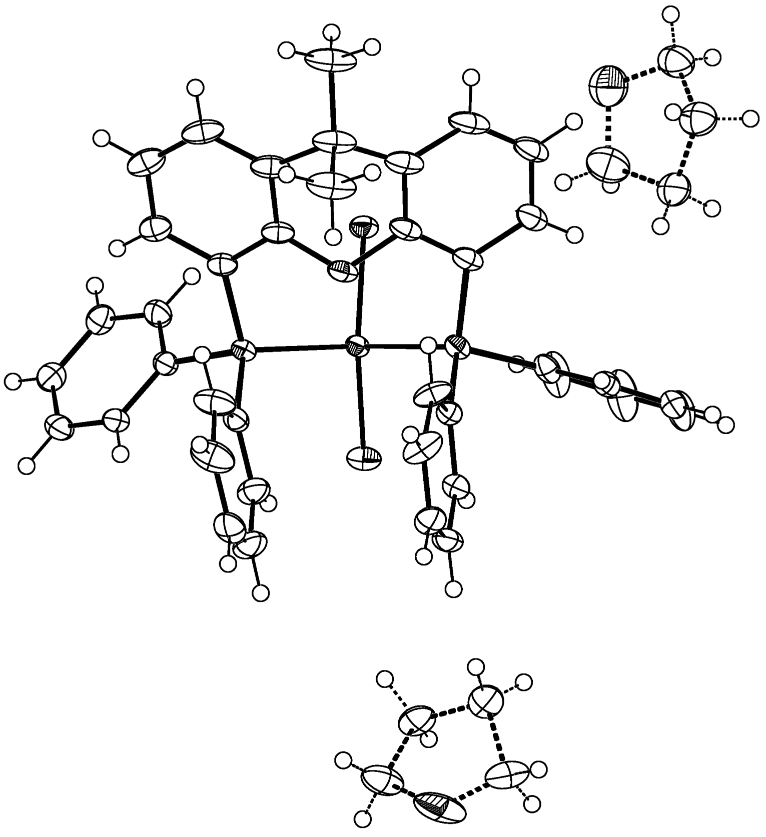

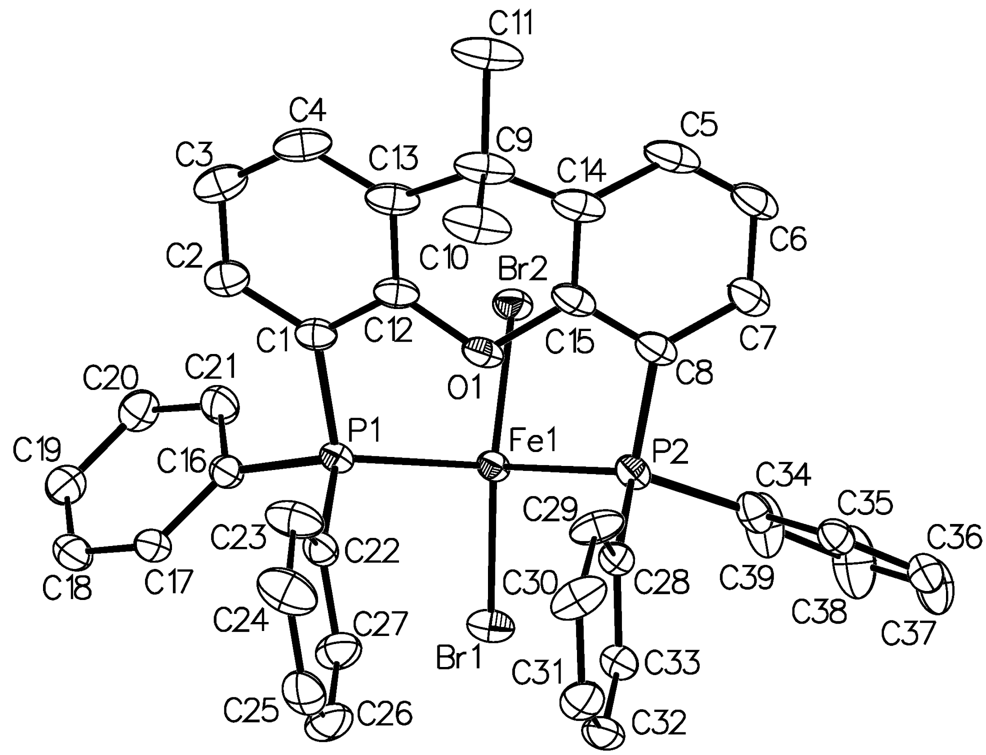
C11
C4
C5
C3
C13
C9
C14
C6
C2
C10
Br2
C1
C12
C15
C7
C8
O1
C20
C21
P1
Fe1
P2
C19
C16
C34
C35
C29
C36
C23
C22
C18
C17
C30
C28
C39
C38
C37
C24
C27
Br1
C33
C31
C25
C26
C32

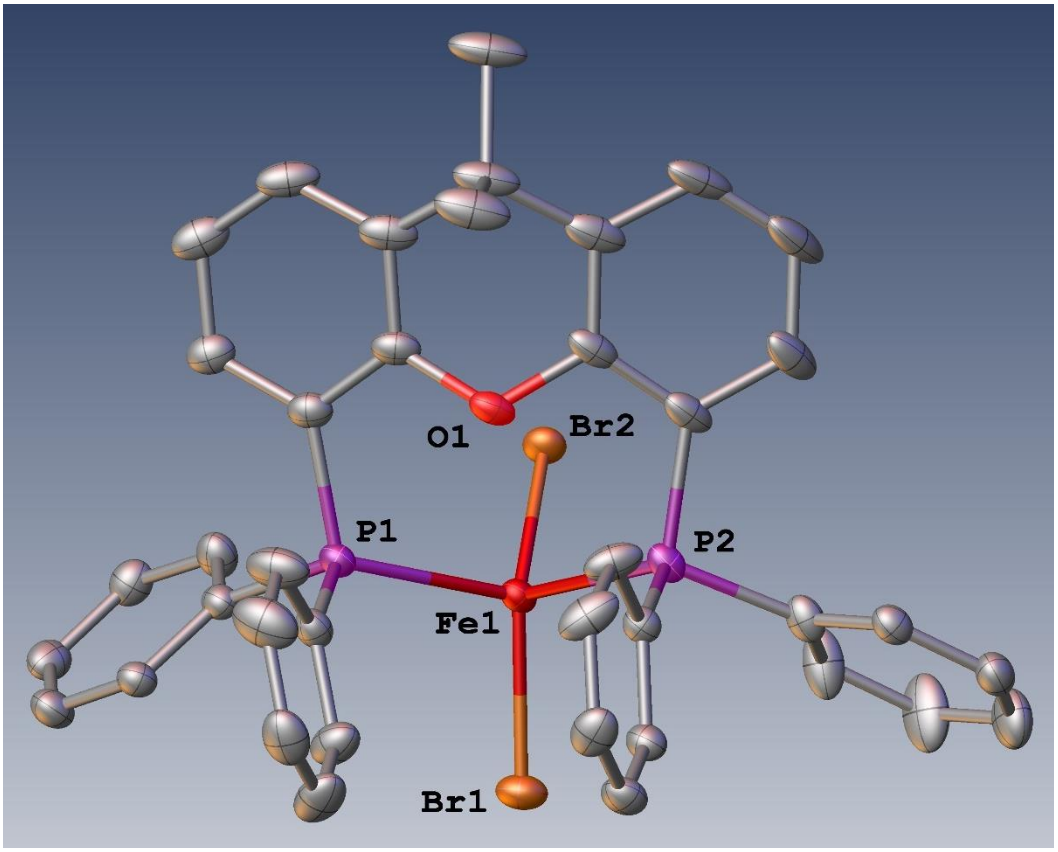
O1
Br2
P1
P2
Fe1
Br1

Table 1. Crystal data and structure refinement for neivf18.

Identification code	neivf18	
Empirical formula	C43 H40 Br2 Fe O2 P2	
Formula weight	866.36	
Temperature	100.01(10) K	
Wavelength	1.54184 Å	
Crystal system	triclinic	
Space group	*P*-1	
Unit cell dimensions	*a* = 9.63700(10) Å	α = 82.5320(10)°
	b = 10.6308(2) Å	β = 89.2270(10)°
	c = 19.5085(3) Å	γ = 74.1650(10)°
Volume	1905.97(5) $Å^3$	
Z	2	
Density (calculated)	1.510 Mg/m^3	
Absorption coefficient	6.701 mm^{-1}	
F(000)	880	
Crystal color, morphology	colourless, block	
Crystal size	0.17 x 0.14 x 0.11 mm^3	
Theta range for data collection	4.361 to 77.903°	
Index ranges	$-12 \le h \le 11$, $-13 \le k \le 13$, $-24 \le l \le 21$	
Reflections collected	29033	
Independent reflections	8003 [*R*(int) = 0.0302]	
Observed reflections	7696	
Completeness to theta = 74.504°	99.8%	
Absorption correction	Multi-scan	
Max. and min. transmission	1.00000 and 0.37354	
Refinement method	Full-matrix least-squares on F^2	
Data / restraints / parameters	8003 / 8 / 498	
Goodness-of-fit on F^2	1.082	
Final *R* indices [*I*>2sigma(*I*)]	*R*1 = 0.0305, *wR*2 = 0.0807	
R indices (all data)	*R*1 = 0.0333, *wR*2 = 0.0829	
Largest diff. peak and hole	0.392 and -0.693 e.$Å^{-3}$	

Table 2. Atomic coordinates (x 10^4) and equivalent isotropic displacement parameters ($Å^2$x 10^3) for neivf18. U_{eq} is defined as one third of the trace of the orthogonalized U_{ij} tensor.

	x	y	z	U_{eq}
Br1	7511(1)	2657(1)	1139(1)	30(1)
Br2	8021(1)	3983(1)	3054(1)	25(1)
Fe1	6654(1)	3402(1)	2200(1)	18(1)
P1	4554(1)	5307(1)	1902(1)	20(1)
P2	5716(1)	1669(1)	2835(1)	21(1)
O1	3831(2)	4051(2)	3189(1)	24(1)
C1	3828(2)	6155(2)	2639(1)	23(1)
C2	3543(2)	7506(2)	2655(1)	27(1)
C3	3021(2)	8040(3)	3255(1)	33(1)
C4	2776(2)	7240(3)	3836(1)	33(1)
C5	4427(3)	2733(3)	5015(1)	41(1)
C6	5379(4)	1529(3)	4956(1)	44(1)
C7	5789(3)	1136(3)	4314(1)	37(1)
C8	5239(2)	1994(2)	3715(1)	27(1)
C9	2699(3)	4925(3)	4426(1)	35(1)
C10	1214(3)	4711(3)	4254(1)	44(1)
C11	2600(3)	5454(4)	5125(1)	46(1)
C12	3564(2)	5384(2)	3233(1)	24(1)
C13	3032(2)	5886(3)	3840(1)	30(1)
C14	3842(3)	3617(3)	4431(1)	33(1)
C15	4310(2)	3219(2)	3796(1)	26(1)
C16	5069(2)	6577(2)	1319(1)	21(1)
C17	4425(2)	7097(2)	673(1)	22(1)
C18	4921(3)	8034(2)	256(1)	29(1)
C19	6045(2)	8472(2)	481(1)	28(1)
C20	6690(2)	7961(2)	1126(1)	28(1)
C21	6220(2)	7006(2)	1538(1)	29(1)
C22	2989(2)	5027(2)	1500(1)	23(1)
C23	1603(3)	5473(3)	1746(1)	40(1)
C24	449(3)	5188(3)	1441(1)	46(1)
C25	667(3)	4487(3)	882(1)	37(1)

C26	2036(3)	4060(3)	630(2)	40(1)
C27	3203(3)	4314(2)	940(1)	33(1)
C28	4099(2)	1418(2)	2465(1)	20(1)
C29	2741(2)	1793(2)	2745(1)	31(1)
C30	1546(2)	1647(3)	2410(2)	37(1)
C31	1700(2)	1102(2)	1800(1)	32(1)
C32	3056(2)	705(2)	1517(1)	28(1)
C33	4248(2)	878(2)	1844(1)	23(1)
C34	6947(2)	20(2)	2919(1)	25(1)
C35	6489(2)	-1093(2)	3154(1)	24(1)
C36	7426(3)	-2341(2)	3172(1)	29(1)
C37	8822(3)	-2500(3)	2953(2)	41(1)
C38	9289(3)	-1398(3)	2720(2)	51(1)
C39	8369(2)	-147(3)	2701(2)	41(1)
O2	-1053(5)	-738(6)	-214(2)	59(1)
C40	-118(6)	-1690(6)	267(3)	41(1)
C41	517(6)	-903(6)	698(4)	40(1)
C42	760(7)	174(7)	151(4)	38(1)
C43	-531(7)	411(9)	-344(3)	45(2)
O3	9990(8)	616(6)	5207(3)	71(2)
C44	9699(8)	169(9)	4569(4)	50(2)
C45	9279(6)	-1106(6)	4754(3)	40(1)
C46	8991(6)	-1120(6)	5524(3)	43(1)
C47	10016(7)	-405(7)	5756(4)	43(1)

Table 3. Bond lengths [Å] and angles [°] for neivf18.

Br(1)-Fe(1)	2.3705(4)	C(10)-H(10C)	0.9600
Br(2)-Fe(1)	2.3796(3)	C(11)-H(11A)	0.9600
Fe(1)-P(1)	2.4548(6)	C(11)-H(11B)	0.9600
Fe(1)-P(2)	2.4549(6)	C(11)-H(11C)	0.9600
P(1)-C(1)	1.827(2)	C(12)-C(13)	1.395(3)
P(1)-C(16)	1.820(2)	C(14)-C(15)	1.389(3)
P(1)-C(22)	1.821(2)	C(16)-C(17)	1.391(3)
P(2)-C(8)	1.823(2)	C(16)-C(21)	1.400(3)
P(2)-C(28)	1.824(2)	C(17)-H(17)	0.9300
P(2)-C(34)	1.817(2)	C(17)-C(18)	1.386(3)
O(1)-C(12)	1.384(3)	C(18)-H(18)	0.9300
O(1)-C(15)	1.386(3)	C(18)-C(19)	1.386(3)
C(1)-C(2)	1.391(3)	C(19)-H(19)	0.9300
C(1)-C(12)	1.392(3)	C(19)-C(20)	1.387(3)
C(2)-H(2)	0.9300	C(20)-H(20)	0.9300
C(2)-C(3)	1.395(3)	C(20)-C(21)	1.382(3)
C(3)-H(3)	0.9300	C(21)-H(21)	0.9300
C(3)-C(4)	1.384(4)	C(22)-C(23)	1.391(3)
C(4)-H(4)	0.9300	C(22)-C(27)	1.391(3)
C(4)-C(13)	1.391(4)	C(23)-H(23)	0.9300
C(5)-H(5)	0.9300	C(23)-C(24)	1.390(4)
C(5)-C(6)	1.374(5)	C(24)-H(24)	0.9300
C(5)-C(14)	1.397(4)	C(24)-C(25)	1.380(4)
C(6)-H(6)	0.9300	C(25)-H(25)	0.9300
C(6)-C(7)	1.390(4)	C(25)-C(26)	1.379(4)
C(7)-H(7)	0.9300	C(26)-H(26)	0.9300
C(7)-C(8)	1.399(3)	C(26)-C(27)	1.389(3)
C(8)-C(15)	1.392(3)	C(27)-H(27)	0.9300
C(9)-C(10)	1.557(3)	C(28)-C(29)	1.388(3)
C(9)-C(11)	1.532(3)	C(28)-C(33)	1.395(3)
C(9)-C(13)	1.524(3)	C(29)-H(29)	0.9300
C(9)-C(14)	1.519(4)	C(29)-C(30)	1.386(3)
C(10)-H(10A)	0.9600	C(30)-H(30)	0.9300
C(10)-H(10B)	0.9600	C(30)-C(31)	1.380(3)

C(31)-H(31)	0.9300	C(45)-C(46)	1.523(8)
C(31)-C(32)	1.389(3)	C(46)-H(46A)	0.9700
C(32)-H(32)	0.9300	C(46)-H(46B)	0.9700
C(32)-C(33)	1.388(3)	C(46)-C(47)	1.506(8)
C(33)-H(33)	0.9300	C(47)-H(47A)	0.9700
C(34)-C(35)	1.394(3)	C(47)-H(47B)	0.9700
C(34)-C(39)	1.400(3)	Br(1)-Fe(1)-Br(2)	126.439(15)
C(35)-H(35)	0.9300	Br(1)-Fe(1)-P(1)	106.094(17)
C(35)-C(36)	1.383(3)	Br(1)-Fe(1)-P(2)	106.619(18)
C(36)-H(36)	0.9300	Br(2)-Fe(1)-P(1)	106.507(17)
C(36)-C(37)	1.378(4)	Br(2)-Fe(1)-P(2)	103.648(17)
C(37)-H(37)	0.9300	P(1)-Fe(1)-P(2)	106.06(2)
C(37)-C(38)	1.388(4)	C(1)-P(1)-Fe(1)	113.62(7)
C(38)-H(38)	0.9300	C(16)-P(1)-Fe(1)	110.23(7)
C(38)-C(39)	1.379(4)	C(16)-P(1)-C(1)	103.27(10)
C(39)-H(39)	0.9300	C(16)-P(1)-C(22)	106.35(9)
O(2)-C(40)	1.413(7)	C(22)-P(1)-Fe(1)	117.80(7)
O(2)-C(43)	1.435(9)	C(22)-P(1)-C(1)	104.33(9)
C(40)-H(40A)	0.9700	C(8)-P(2)-Fe(1)	111.88(7)
C(40)-H(40B)	0.9700	C(8)-P(2)-C(28)	104.83(9)
C(40)-C(41)	1.503(7)	C(28)-P(2)-Fe(1)	116.08(7)
C(41)-H(41A)	0.9700	C(34)-P(2)-Fe(1)	115.09(7)
C(41)-H(41B)	0.9700	C(34)-P(2)-C(8)	105.67(10)
C(41)-C(42)	1.525(10)	C(34)-P(2)-C(28)	102.10(9)
C(42)-H(42A)	0.9700	C(12)-O(1)-C(15)	115.44(16)
C(42)-H(42B)	0.9700	C(2)-C(1)-P(1)	124.95(17)
C(42)-C(43)	1.531(8)	C(2)-C(1)-C(12)	117.69(19)
C(43)-H(43A)	0.9700	C(12)-C(1)-P(1)	117.35(16)
C(43)-H(43B)	0.9700	C(1)-C(2)-H(2)	120.1
O(3)-C(44)	1.444(8)	C(1)-C(2)-C(3)	119.9(2)
O(3)-C(47)	1.417(8)	C(3)-C(2)-H(2)	120.1
C(44)-H(44A)	0.9700	C(2)-C(3)-H(3)	119.7
C(44)-H(44B)	0.9700	C(4)-C(3)-C(2)	120.7(2)
C(44)-C(45)	1.516(10)	C(4)-C(3)-H(3)	119.7
C(45)-H(45A)	0.9700	C(3)-C(4)-H(4)	119.4
C(45)-H(45B)	0.9700	C(3)-C(4)-C(13)	121.3(2)

C(13)-C(4)-H(4)	119.4	C(12)-C(13)-C(9)	117.4(2)
C(6)-C(5)-H(5)	119.4	C(5)-C(14)-C(9)	126.1(2)
C(6)-C(5)-C(14)	121.2(2)	C(15)-C(14)-C(5)	116.1(3)
C(14)-C(5)-H(5)	119.4	C(15)-C(14)-C(9)	117.7(2)
C(5)-C(6)-H(6)	119.3	O(1)-C(15)-C(8)	115.41(18)
C(5)-C(6)-C(7)	121.4(2)	O(1)-C(15)-C(14)	120.3(2)
C(7)-C(6)-H(6)	119.3	C(14)-C(15)-C(8)	124.3(2)
C(6)-C(7)-H(7)	120.4	C(17)-C(16)-P(1)	124.00(16)
C(6)-C(7)-C(8)	119.3(3)	C(17)-C(16)-C(21)	119.22(19)
C(8)-C(7)-H(7)	120.4	C(21)-C(16)-P(1)	116.74(15)
C(7)-C(8)-P(2)	125.1(2)	C(16)-C(17)-H(17)	120.1
C(15)-C(8)-P(2)	117.26(15)	C(18)-C(17)-C(16)	119.87(19)
C(15)-C(8)-C(7)	117.5(2)	C(18)-C(17)-H(17)	120.1
C(11)-C(9)-C(10)	108.38(19)	C(17)-C(18)-H(18)	119.7
C(13)-C(9)-C(10)	107.6(2)	C(17)-C(18)-C(19)	120.6(2)
C(13)-C(9)-C(11)	112.4(2)	C(19)-C(18)-H(18)	119.7
C(14)-C(9)-C(10)	108.0(2)	C(18)-C(19)-H(19)	120.0
C(14)-C(9)-C(11)	112.7(2)	C(18)-C(19)-C(20)	119.9(2)
C(14)-C(9)-C(13)	107.61(18)	C(20)-C(19)-H(19)	120.0
C(9)-C(10)-H(10A)	109.5	C(19)-C(20)-H(20)	120.1
C(9)-C(10)-H(10B)	109.5	C(21)-C(20)-C(19)	119.8(2)
C(9)-C(10)-H(10C)	109.5	C(21)-C(20)-H(20)	120.1
H(10A)-C(10)-H(10B)	109.5	C(16)-C(21)-H(21)	119.7
H(10A)-C(10)-H(10C)	109.5	C(20)-C(21)-C(16)	120.6(2)
H(10B)-C(10)-H(10C)	109.5	C(20)-C(21)-H(21)	119.7
C(9)-C(11)-H(11A)	109.5	C(23)-C(22)-P(1)	122.20(17)
C(9)-C(11)-H(11B)	109.5	C(23)-C(22)-C(27)	119.4(2)
C(9)-C(11)-H(11C)	109.5	C(27)-C(22)-P(1)	118.40(17)
H(11A)-C(11)-H(11B)	109.5	C(22)-C(23)-H(23)	119.9
H(11A)-C(11)-H(11C)	109.5	C(24)-C(23)-C(22)	120.2(2)
H(11B)-C(11)-H(11C)	109.5	C(24)-C(23)-H(23)	119.9
O(1)-C(12)-C(1)	115.72(18)	C(23)-C(24)-H(24)	119.9
O(1)-C(12)-C(13)	120.3(2)	C(25)-C(24)-C(23)	120.2(3)
C(1)-C(12)-C(13)	123.9(2)	C(25)-C(24)-H(24)	119.9
C(4)-C(13)-C(9)	125.9(2)	C(24)-C(25)-H(25)	120.1
C(4)-C(13)-C(12)	116.5(2)	C(26)-C(25)-C(24)	119.9(2)

C(26)-C(25)-H(25)	120.1	C(38)-C(37)-H(37)	120.2
C(25)-C(26)-H(26)	119.7	C(37)-C(38)-H(38)	119.7
C(25)-C(26)-C(27)	120.5(2)	C(39)-C(38)-C(37)	120.7(2)
C(27)-C(26)-H(26)	119.7	C(39)-C(38)-H(38)	119.7
C(22)-C(27)-H(27)	120.1	C(34)-C(39)-H(39)	120.0
C(26)-C(27)-C(22)	119.9(2)	C(38)-C(39)-C(34)	119.9(2)
C(26)-C(27)-H(27)	120.1	C(38)-C(39)-H(39)	120.0
C(29)-C(28)-P(2)	123.85(16)	C(40)-O(2)-C(43)	109.5(5)
C(29)-C(28)-C(33)	119.02(18)	O(2)-C(40)-H(40A)	110.8
C(33)-C(28)-P(2)	117.04(15)	O(2)-C(40)-H(40B)	110.8
C(28)-C(29)-H(29)	119.7	O(2)-C(40)-C(41)	104.7(5)
C(30)-C(29)-C(28)	120.5(2)	H(40A)-C(40)-H(40B)	108.9
C(30)-C(29)-H(29)	119.7	C(41)-C(40)-H(40A)	110.8
C(29)-C(30)-H(30)	119.9	C(41)-C(40)-H(40B)	110.8
C(31)-C(30)-C(29)	120.3(2)	C(40)-C(41)-H(41A)	111.5
C(31)-C(30)-H(30)	119.9	C(40)-C(41)-H(41B)	111.5
C(30)-C(31)-H(31)	120.0	C(40)-C(41)-C(42)	101.4(5)
C(30)-C(31)-C(32)	119.9(2)	H(41A)-C(41)-H(41B)	109.3
C(32)-C(31)-H(31)	120.0	C(42)-C(41)-H(41A)	111.5
C(31)-C(32)-H(32)	120.1	C(42)-C(41)-H(41B)	111.5
C(33)-C(32)-C(31)	119.8(2)	C(41)-C(42)-H(42A)	111.3
C(33)-C(32)-H(32)	120.1	C(41)-C(42)-H(42B)	111.3
C(28)-C(33)-H(33)	119.8	C(41)-C(42)-C(43)	102.2(7)
C(32)-C(33)-C(28)	120.44(19)	H(42A)-C(42)-H(42B)	109.2
C(32)-C(33)-H(33)	119.8	C(43)-C(42)-H(42A)	111.3
C(35)-C(34)-P(2)	121.68(16)	C(43)-C(42)-H(42B)	111.3
C(35)-C(34)-C(39)	118.9(2)	O(2)-C(43)-C(42)	106.3(8)
C(39)-C(34)-P(2)	119.26(17)	O(2)-C(43)-H(43A)	110.5
C(34)-C(35)-H(35)	119.8	O(2)-C(43)-H(43B)	110.5
C(36)-C(35)-C(34)	120.4(2)	C(42)-C(43)-H(43A)	110.5
C(36)-C(35)-H(35)	119.8	C(42)-C(43)-H(43B)	110.5
C(35)-C(36)-H(36)	119.8	H(43A)-C(43)-H(43B)	108.7
C(37)-C(36)-C(35)	120.4(2)	C(47)-O(3)-C(44)	108.2(7)
C(37)-C(36)-H(36)	119.8	O(3)-C(44)-H(44A)	110.2
C(36)-C(37)-H(37)	120.2	O(3)-C(44)-H(44B)	110.2
C(36)-C(37)-C(38)	119.6(2)	O(3)-C(44)-C(45)	107.7(7)

H(44A)-C(44)-H(44B)	108.5	H(46A)-C(46)-H(46B)	109.2
C(45)-C(44)-H(44A)	110.2	C(47)-C(46)-C(45)	102.5(5)
C(45)-C(44)-H(44B)	110.2	C(47)-C(46)-H(46A)	111.3
C(44)-C(45)-H(45A)	111.2	C(47)-C(46)-H(46B)	111.3
C(44)-C(45)-H(45B)	111.2	O(3)-C(47)-C(46)	104.9(5)
C(44)-C(45)-C(46)	102.6(5)	O(3)-C(47)-H(47A)	110.8
H(45A)-C(45)-H(45B)	109.2	O(3)-C(47)-H(47B)	110.8
C(46)-C(45)-H(45A)	111.2	C(46)-C(47)-H(47A)	110.8
C(46)-C(45)-H(45B)	111.2	C(46)-C(47)-H(47B)	110.8
C(45)-C(46)-H(46A)	111.3	H(47A)-C(47)-H(47B)	108.9
C(45)-C(46)-H(46B)	111.3		

Table 4. Anisotropic displacement parameters ($Å^2 x 10^3$) for neivf18. The anisotropic displacement factor exponent takes the form: $-2\pi^2[h^2 a^{*2}U_{11} + ... + 2 h k a^* b^* U_{12}]$

	U_{11}	U_{22}	U_{33}	U_{23}	U_{13}	U_{12}
Br1	32(1)	40(1)	21(1)	-10(1)	2(1)	-11(1)
Br2	26(1)	28(1)	22(1)	-7(1)	-5(1)	-8(1)
Fe1	20(1)	21(1)	16(1)	-1(1)	-1(1)	-8(1)
P1	21(1)	22(1)	16(1)	-2(1)	-2(1)	-6(1)
P2	21(1)	25(1)	18(1)	1(1)	-2(1)	-12(1)
O1	29(1)	33(1)	15(1)	-4(1)	0(1)	-16(1)
C1	20(1)	30(1)	22(1)	-8(1)	-2(1)	-8(1)
C2	21(1)	33(1)	30(1)	-10(1)	-1(1)	-8(1)
C3	22(1)	40(1)	44(1)	-21(1)	4(1)	-13(1)
C4	24(1)	52(2)	34(1)	-25(1)	9(1)	-20(1)
C5	64(2)	63(2)	15(1)	-5(1)	3(1)	-50(2)
C6	79(2)	51(2)	17(1)	7(1)	-10(1)	-49(2)
C7	61(2)	40(1)	22(1)	7(1)	-10(1)	-37(1)
C8	37(1)	37(1)	16(1)	1(1)	-3(1)	-26(1)
C9	32(1)	64(2)	22(1)	-17(1)	9(1)	-32(1)
C10	37(1)	85(2)	28(1)	-19(1)	10(1)	-40(1)
C11	42(1)	90(2)	25(1)	-26(1)	12(1)	-39(2)
C12	21(1)	36(1)	21(1)	-10(1)	1(1)	-13(1)
C13	24(1)	51(1)	24(1)	-15(1)	3(1)	-20(1)
C14	39(1)	54(2)	19(1)	-7(1)	3(1)	-36(1)
C15	32(1)	40(1)	16(1)	0(1)	-2(1)	-25(1)
C16	22(1)	22(1)	19(1)	-3(1)	0(1)	-4(1)
C17	24(1)	25(1)	19(1)	-4(1)	-1(1)	-8(1)
C18	37(1)	31(1)	20(1)	-1(1)	-1(1)	-12(1)
C19	32(1)	28(1)	27(1)	-5(1)	8(1)	-11(1)
C20	24(1)	27(1)	36(1)	-6(1)	-1(1)	-8(1)
C21	30(1)	29(1)	28(1)	0(1)	-8(1)	-9(1)
C22	25(1)	25(1)	21(1)	0(1)	-4(1)	-9(1)
C23	28(1)	63(2)	29(1)	-16(1)	-4(1)	-11(1)
C24	29(1)	74(2)	36(1)	-7(1)	-6(1)	-16(1)
C25	34(1)	39(1)	39(1)	2(1)	-16(1)	-17(1)

C26	40(1)	34(1)	49(2)	-17(1)	-16(1)	-6(1)
C27	28(1)	33(1)	38(1)	-14(1)	-7(1)	-4(1)
C28	18(1)	23(1)	20(1)	-2(1)	-1(1)	-8(1)
C29	24(1)	40(1)	39(1)	-23(1)	9(1)	-17(1)
C30	19(1)	40(1)	58(2)	-27(1)	7(1)	-11(1)
C31	25(1)	32(1)	41(1)	-9(1)	-11(1)	-9(1)
C32	32(1)	34(1)	21(1)	-5(1)	-4(1)	-12(1)
C33	22(1)	27(1)	20(1)	-3(1)	0(1)	-6(1)
C34	19(1)	30(1)	26(1)	4(1)	-6(1)	-9(1)
C35	25(1)	28(1)	20(1)	0(1)	-1(1)	-12(1)
C36	36(1)	26(1)	25(1)	1(1)	-4(1)	-8(1)
C37	31(1)	35(1)	47(2)	7(1)	-6(1)	2(1)
C38	17(1)	49(2)	76(2)	18(2)	-3(1)	-1(1)
C39	19(1)	39(1)	60(2)	18(1)	-5(1)	-11(1)
O2	63(3)	106(4)	31(2)	-2(2)	-8(2)	-65(3)
C40	43(3)	58(3)	36(3)	-24(2)	15(2)	-31(3)
C41	27(2)	41(3)	52(4)	-10(3)	-11(3)	-8(2)
C42	33(3)	42(4)	48(4)	-15(3)	-5(3)	-19(3)
C43	46(4)	67(5)	31(4)	-4(3)	-4(3)	-33(4)
O3	110(5)	53(3)	56(3)	0(3)	-1(3)	-38(3)
C44	45(4)	68(5)	39(4)	-9(4)	6(3)	-19(3)
C45	33(3)	39(3)	43(3)	-12(2)	-2(2)	4(2)
C46	38(3)	48(3)	43(3)	-12(2)	2(2)	-8(2)
C47	38(3)	49(4)	38(4)	-10(3)	-5(3)	-1(3)

Table 5. Hydrogen coordinates (x 10^4) and isotropic displacement parameters ($Å^2$ x 10^3) for neivf18.

	x	y	z	U(eq)
H2	3701	8051	2267	33
H3	2835	8943	3264	40
H4	2435	7615	4232	40
H5	4167	2963	5452	49
H6	5757	964	5355	53
H7	6423	312	4284	45
H10A	992	4082	4607	67
H10B	1255	4386	3816	67
H10C	478	5534	4234	67
H11A	1845	6264	5100	70
H11B	3502	5610	5234	70
H11C	2390	4818	5478	70
H17	3662	6818	521	27
H18	4497	8372	-179	35
H19	6366	9107	200	34
H20	7435	8261	1281	34
H21	6673	6646	1964	35
H23	1448	5963	2115	47
H24	-473	5470	1613	55
H25	-107	4303	677	44
H26	2178	3600	250	48
H27	4126	4007	774	40
H29	2631	2146	3161	38
H30	637	1918	2598	44
H31	897	1000	1578	38
H32	3165	324	1110	34
H33	5151	633	1649	28
H35	5547	-995	3299	28
H36	7112	-3077	3333	35
H37	9447	-3341	2963	49

H38	10232	-1505	2574	62
H39	8693	585	2545	49
H40A	631	-2277	33	49
H40B	-648	-2209	552	49
H41A	1417	-1431	923	48
H41B	-149	-535	1044	48
H42A	1665	-124	-81	46
H42B	751	965	351	46
H43A	-1278	1192	-259	54
H43B	-238	532	-821	54
H44A	10550	21	4287	60
H44B	8920	828	4310	60
H45A	10057	-1862	4667	48
H45B	8422	-1098	4497	48
H46A	7997	-660	5604	51
H46B	9203	-2015	5760	51
H47A	9698	-43	6180	52
H47B	10981	-995	5831	52

Table 6. Torsion angles [°] for neivf18.

Fe1-P1-C1-C2	129.12(16)	C2-C1-C12-O1	178.59(17)
Fe1-P1-C1-C12	-49.70(17)	C2-C1-C12-C13	0.1(3)
Fe1-P1-C16-C17	123.56(16)	C2-C3-C4-C13	-0.5(3)
Fe1-P1-C16-C21	-54.21(18)	C3-C4-C13-C9	-175.1(2)
Fe1-P1-C22-C23	129.4(2)	C3-C4-C13-C12	0.9(3)
Fe1-P1-C22-C27	-48.9(2)	C5-C6-C7-C8	1.0(4)
Fe1-P2-C8-C7	-123.50(18)	C5-C14-C15-O1	-177.63(18)
Fe1-P2-C8-C15	53.08(17)	C5-C14-C15-C8	3.7(3)
Fe1-P2-C28-C29	-107.30(19)	C6-C5-C14-C9	175.0(2)
Fe1-P2-C28-C33	69.21(17)	C6-C5-C14-C15	-1.7(3)
Fe1-P2-C34-C35	-168.42(15)	C6-C7-C8-P2	177.38(18)
Fe1-P2-C34-C39	7.5(2)	C6-C7-C8-C15	0.8(3)
P1-C1-C2-C3	-178.42(16)	C7-C8-C15-O1	177.97(18)
P1-C1-C12-O1	-2.5(2)	C7-C8-C15-C14	-3.3(3)
P1-C1-C12-C13	179.00(16)	C8-P2-C28-C29	16.7(2)
P1-C16-C17-C18	-177.99(17)	C8-P2-C28-C33	-166.82(17)
P1-C16-C21-C20	179.58(17)	C8-P2-C34-C35	67.61(19)
P1-C22-C23-C24	-177.3(2)	C8-P2-C34-C39	-116.4(2)
P1-C22-C27-C26	178.8(2)	C9-C14-C15-O1	5.4(3)
P2-C8-C15-O1	1.1(2)	C9-C14-C15-C8	-173.32(19)
P2-C8-C15-C14	179.87(16)	C10-C9-C13-C4	96.8(3)
P2-C28-C29-C30	175.8(2)	C10-C9-C13-C12	-79.3(3)
P2-C28-C33-C32	-177.48(17)	C10-C9-C14-C5	-99.1(3)
P2-C34-C35-C36	176.00(16)	C10-C9-C14-C15	77.6(2)
P2-C34-C39-C38	-175.8(2)	C11-C9-C13-C4	-22.4(3)
O1-C12-C13-C4	-179.17(18)	C11-C9-C13-C12	161.5(2)
O1-C12-C13-C9	-2.8(3)	C11-C9-C14-C5	20.6(3)
C1-P1-C16-C17	-114.73(18)	C11-C9-C14-C15	-162.7(2)
C1-P1-C16-C21	67.50(18)	C12-O1-C15-C8	-148.54(18)
C1-P1-C22-C23	2.4(2)	C12-O1-C15-C14	32.7(3)
C1-P1-C22-C27	-175.95(18)	C12-C1-C2-C3	0.4(3)
C1-C2-C3-C4	-0.2(3)	C13-C9-C14-C5	145.0(2)
C1-C12-C13-C4	-0.7(3)	C13-C9-C14-C15	-38.3(2)
C1-C12-C13-C9	175.67(19)	C14-C5-C6-C7	-0.6(4)

C14-C9-C13-C4	-147.1(2)	C34-P2-C28-C29	126.7(2)
C14-C9-C13-C12	36.9(2)	C34-P2-C28-C33	-56.79(18)
C15-O1-C12-C1	147.46(18)	C34-C35-C36-C37	-0.5(3)
C15-O1-C12-C13	-34.0(3)	C35-C34-C39-C38	0.3(4)
C16-P1-C1-C2	9.7(2)	C35-C36-C37-C38	0.6(4)
C16-P1-C1-C12	-169.09(16)	C36-C37-C38-C39	-0.3(5)
C16-P1-C22-C23	-106.4(2)	C37-C38-C39-C34	-0.1(5)
C16-P1-C22-C27	75.3(2)	C39-C34-C35-C36	0.0(3)
C16-C17-C18-C19	-0.9(3)	O2-C40-C41-C42	-39.7(5)
C17-C16-C21-C20	1.7(3)	C40-O2-C43-C42	-5.2(7)
C17-C18-C19-C20	0.6(3)	C40-C41-C42-C43	35.2(6)
C18-C19-C20-C21	0.8(3)	C41-C42-C43-O2	-19.5(6)
C19-C20-C21-C16	-2.0(3)	C43-O2-C40-C41	28.5(6)
C21-C16-C17-C18	-0.3(3)	O3-C44-C45-C46	14.1(7)
C22-P1-C1-C2	-101.30(19)	C44-O3-C47-C46	-30.1(7)
C22-P1-C1-C12	79.88(17)	C44-C45-C46-C47	-31.0(6)
C22-P1-C16-C17	-5.2(2)	C45-C46-C47-O3	38.1(6)
C22-P1-C16-C21	177.02(17)	C47-O3-C44-C45	9.8(8)
C22-C23-C24-C25	-1.5(5)		
C23-C22-C27-C26	0.3(4)		
C23-C24-C25-C26	0.5(4)		
C24-C25-C26-C27	0.9(4)		
C25-C26-C27-C22	-1.4(4)		
C27-C22-C23-C24	1.1(4)		
C28-P2-C8-C7	109.89(19)		
C28-P2-C8-C15	-73.53(17)		
C28-P2-C34-C35	-41.8(2)		
C28-P2-C34-C39	134.2(2)		
C28-C29-C30-C31	1.3(4)		
C29-C28-C33-C32	-0.8(3)		
C29-C30-C31-C32	-0.4(4)		
C30-C31-C32-C33	-1.0(4)		
C31-C32-C33-C28	1.6(3)		
C33-C28-C29-C30	-0.7(4)		
C34-P2-C8-C7	2.5(2)		
C34-P2-C8-C15	179.05(16)		

3.2 Monoalkylated Fe(II)-Xantphos (2-CH_2SiMe_3)

CRYSTAL STRUCTURE REPORT

C_{55} H_{67} Br Fe O_4 P_2 Si

or

(Xantphos)FeBr(CH_2SiMe_3) · 3THF

Report prepared for:

M. C. Aguilera, Prof. M. Neidig

December 07, 2020

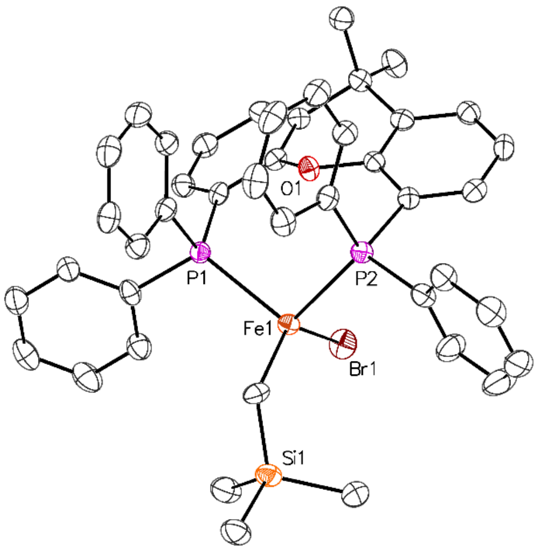

William W. Brennessel
X-ray Crystallographic Facility
Department of Chemistry, University of Rochester
120 Trustee Road
Rochester, NY 14627

Data collection

A crystal (0.336 x 0.084 x 0.024 mm^3) was placed onto a thin glass optical fiber or a nylon loop and mounted on a Rigaku XtaLAB Synergy-S Dualflex diffractometer equipped with a HyPix-6000HE HPC area detector for data collection at 100.00(10) K. A preliminary set of cell constants and an orientation matrix were calculated from a small sampling of reflections.[1] A short pre-experiment was run, from which an optimal data collection strategy was determined. The full data collection was carried out using a PhotonJet (Cu) X-ray source with frame times of 4.03 and 16.11 seconds and a detector distance of 31.2 mm. Series of frames were collected in 0.50º steps in ω at different 2θ, κ, and ϕ settings. After the intensity data were corrected for absorption, the final cell constants were calculated from the xyz centroids of 19042 strong reflections from the actual data collection after integration.[1] See Table 1 for additional crystal and refinement information.

Structure solution and refinement

The structure was solved using SHELXT[2] and refined using SHELXL.[3] The space group $P2_1/n$ was determined based on systematic absences. Most or all non-hydrogen atoms were assigned from the solution. Full-matrix least squares / difference Fourier cycles were performed which located any remaining non-hydrogen atoms. All non-hydrogen atoms were refined with anisotropic displacement parameters. All hydrogen atoms were placed in ideal positions and refined as riding atoms with relative isotropic displacement parameters. The final full matrix least squares refinement converged to $R1 = 0.0720$ (F^2, $I > 2\sigma(I)$) and $wR2 = 0.2136$ (F^2, all data).

Structure description

The structure is the one suggested. The asymmetric unit contains one iron complex and three cocrystallized THF solvent molecules in general positions. Two adjacent cocrystallized THF solvent molecules were modeled as disordered over two positions (0.74:0.26).

Structure manipulation and figure generation were performed using Olex2.[4] Unless noted otherwise all structural diagrams containing anisotropic displacement ellipsoids are drawn at the 50 % probability level.

Data collection, structure solution, and structure refinement were conducted at the X-ray Crystallographic Facility, B04 Hutchison Hall, Department of Chemistry, University of Rochester. The instrument was purchased with funding from NSF MRI program grant CHE-1725028. All publications arising from this report MUST either 1) include William W. Brennessel as a coauthor or 2) acknowledge William W. Brennessel and the X-ray Crystallographic Facility of the Department of Chemistry at the University of Rochester.

1 *CrysAlisPro*, version 171.40.84a; Rigaku Corporation: Oxford, UK, 2020.

2 Sheldrick, G. M. *SHELXT*, version 2018/2; *Acta. Crystallogr.* **2015**, *A71*, 3-8.

3 Sheldrick, G. M. *SHELXL*, version 2018/3; *Acta. Crystallogr.* **2015**, *C71*, 3-8.

4 Dolomanov, O. V.; Bourhis, L. J.; Gildea, R. J.; Howard, J. A. K.; Puschmann, H. *Olex2*, version 1.2-ac3; *J. Appl. Cryst.* **2009**, *42*, 339-341.

Some equations of interest:

$$R_{int} = \Sigma\, |F_o^2 - \langle F_o^2 \rangle| \,/\, \Sigma|F_o^2|$$

$$R1 = \Sigma||F_o|-|F_c|| \,/\, \Sigma|F_o|$$

$$wR2 = [\Sigma[w(F_o^2-F_c^2)^2] \,/\, \Sigma\, [w(F_o^2)^2]]^{1/2}$$

where $w = 1 / [\sigma^2 (F_o^2) + (aP)^2 + bP]$ and

$$P = 1/3 \max (0, F_o^2) + 2/3\, F_c^2$$

$$\text{GOF} = S = [\Sigma[w(F_o^2-F_c^2)^2] \,/\, (m-n)]^{1/2}$$

where m = number of reflections and n = number of parameters

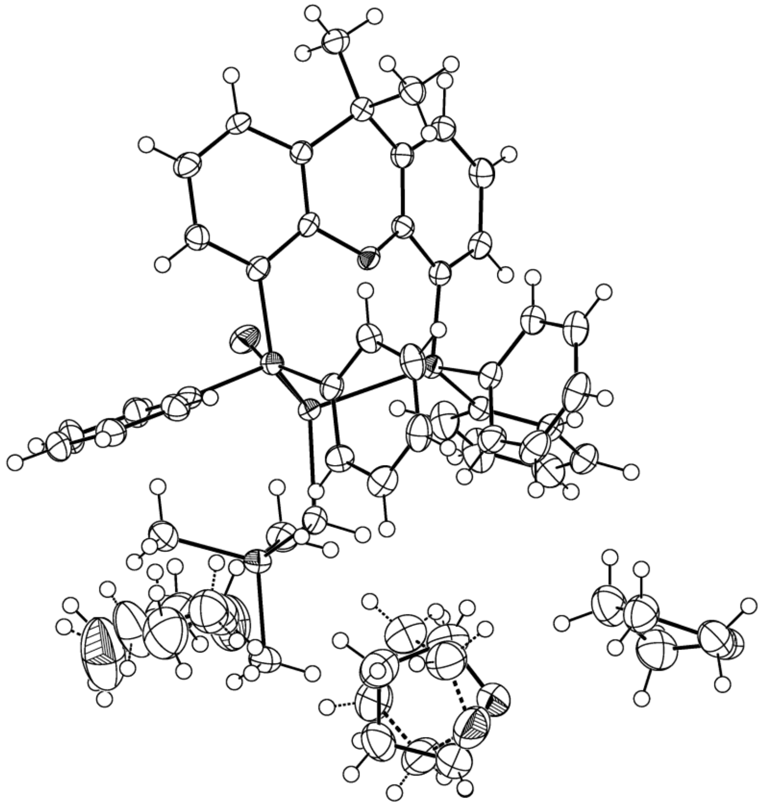

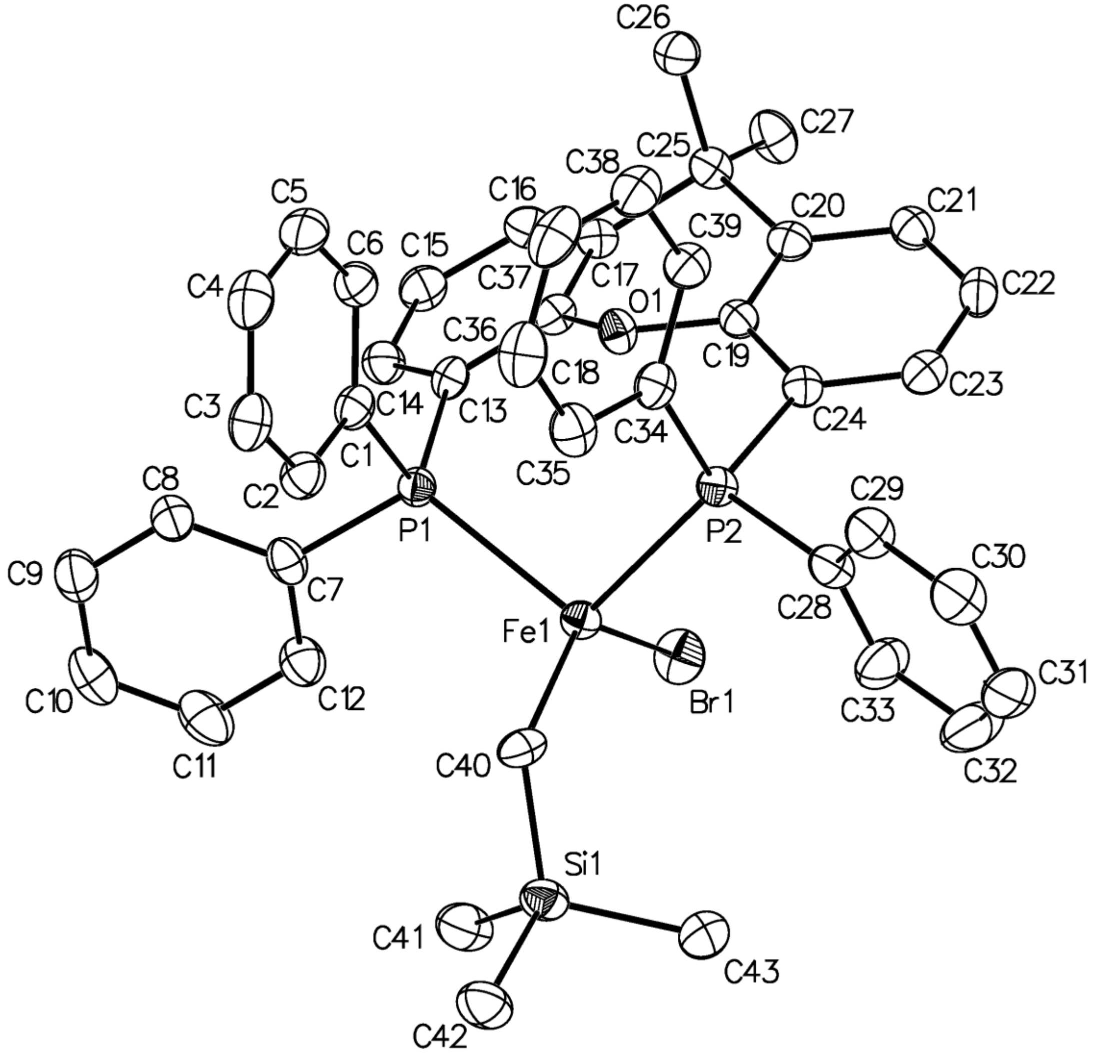
C26
C27
C38
C25
C16
C20
C21
C5
C39
C6
C15
C4
C37
C17
C22
O1
C36
C19
C18
C3
C14
C13
C23
C34
C24
C1
C35
C8
C2
C29
P1
P2
C30
C9
C7
C28
Fe1
C31
C12
Br1
C33
C10
C32
C11
C40
Si1
C41
C43
C42

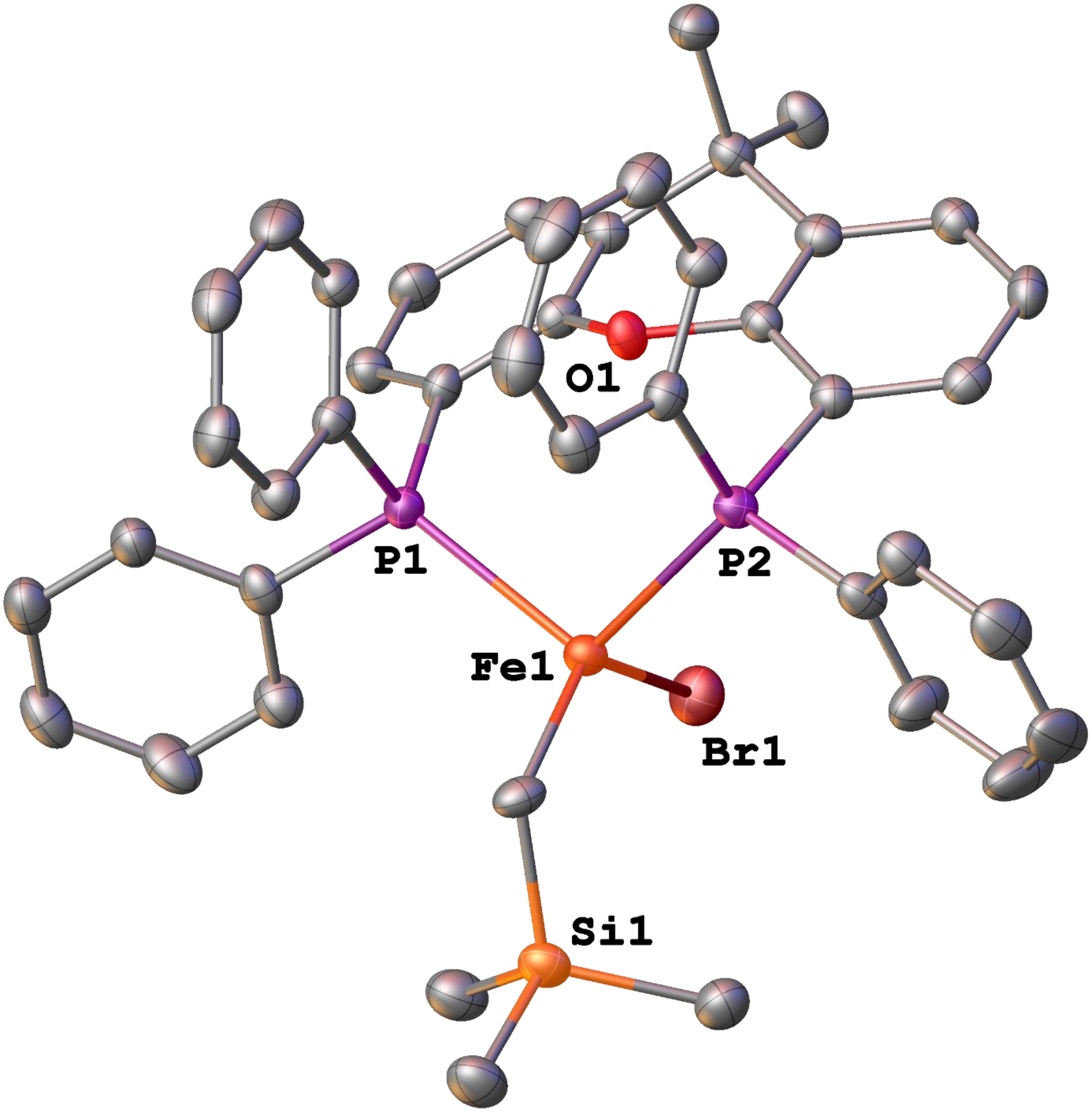
O1
P1
P2
Fe1
Br1
Si1

Table 1. Crystal data and structure refinement for neimca14.

Identification code	neimca14	
Empirical formula	C55 H67 Br Fe O4 P2 Si	
Formula weight	1017.87	
Temperature	100.00(10) K	
Wavelength	1.54184 Å	
Crystal system	monoclinic	
Space group	$P2_1/n$	
Unit cell dimensions	*a* = 10.6670(3) Å	α = 90°
	b = 28.3462(8) Å	β = 101.992(3)°
	c = 17.3132(5) Å	γ = 90°
Volume	5120.8(3) $Å^3$	
Z	4	
Density (calculated)	1.320 Mg/m^3	
Absorption coefficient	4.432 mm^{-1}	
F(000)	2136	
Crystal color, morphology	colourless, needle	
Crystal size	0.336 x 0.084 x 0.024 mm^3	
Theta range for data collection	3.040 to 77.876°	
Index ranges	$-13 \le h \le 9$, $-33 \le k \le 35$, $-21 \le l \le 21$	
Reflections collected	41829	
Independent reflections	10527 [*R*(int) = 0.0863]	
Observed reflections	9201	
Completeness to theta = 74.504°	98.5%	
Absorption correction	Multi-scan	
Max. and min. transmission	1.00000 and 0.46093	
Refinement method	Full-matrix least-squares on F^2	
Data / restraints / parameters	10527 / 268 / 673	
Goodness-of-fit on F^2	1.032	
Final *R* indices [*I*>2sigma(*I*)]	*R*1 = 0.0720, *wR*2 = 0.2063	
R indices (all data)	*R*1 = 0.0791, *wR*2 = 0.2136	
Largest diff. peak and hole	1.505 and -1.422 e.$Å^{-3}$	

Table 2. Atomic coordinates (x 10^4) and equivalent isotropic displacement parameters ($Å^2$x 10^3) for neimca14. U_{eq} is defined as one third of the trace of the orthogonalized U_{ij} tensor.

	x	y	z	U_{eq}
Br1	5492(1)	3838(1)	1605(1)	31(1)
Fe1	3591(1)	3809(1)	2131(1)	17(1)
P1	2064(1)	4305(1)	1203(1)	18(1)
P2	4106(1)	4304(1)	3338(1)	18(1)
Si1	3427(1)	2653(1)	2181(1)	25(1)
O1	3689(2)	4992(1)	2140(1)	19(1)
C1	716(3)	4501(1)	1611(2)	21(1)
C2	-85(4)	4149(1)	1806(2)	26(1)
C3	-1155(4)	4266(2)	2101(2)	31(1)
C4	-1429(4)	4734(2)	2216(2)	34(1)
C5	-628(4)	5085(2)	2042(3)	32(1)
C6	445(4)	4973(1)	1737(2)	25(1)
C7	1264(4)	4008(1)	300(2)	22(1)
C8	9(4)	4118(1)	-86(2)	26(1)
C9	-549(4)	3893(2)	-778(3)	31(1)
C10	134(4)	3559(2)	-1107(2)	34(1)
C11	1369(5)	3452(2)	-732(3)	36(1)
C12	1929(4)	3674(2)	-31(2)	28(1)
C13	2739(3)	4827(1)	821(2)	18(1)
C14	2581(4)	4934(1)	21(2)	23(1)
C15	3256(4)	5306(1)	-224(2)	25(1)
C16	4083(4)	5576(1)	323(2)	22(1)
C17	4257(3)	5485(1)	1135(2)	18(1)
C18	3577(3)	5108(1)	1354(2)	18(1)
C19	4849(3)	5093(1)	2630(2)	18(1)
C20	5575(3)	5474(1)	2492(2)	20(1)
C21	6683(4)	5568(1)	3059(2)	22(1)
C22	7034(4)	5285(1)	3722(2)	25(1)
C23	6304(4)	4896(1)	3836(2)	22(1)
C24	5191(3)	4794(1)	3284(2)	19(1)
C25	5090(4)	5788(1)	1775(2)	21(1)

C26	4234(4)	6174(1)	2023(2)	27(1)
C27	6194(4)	6018(2)	1471(2)	29(1)
C28	4943(4)	3977(1)	4201(2)	24(1)
C29	4699(4)	4042(2)	4952(2)	28(1)
C30	5380(5)	3786(2)	5590(3)	37(1)
C31	6302(6)	3470(2)	5474(3)	45(1)
C32	6573(6)	3408(2)	4731(3)	51(1)
C33	5870(5)	3657(2)	4094(3)	40(1)
C34	2791(4)	4592(1)	3680(2)	22(1)
C35	1701(4)	4330(2)	3711(2)	30(1)
C36	681(4)	4543(2)	3958(3)	36(1)
C37	745(4)	5013(2)	4173(3)	36(1)
C38	1816(4)	5277(2)	4149(2)	31(1)
C39	2849(4)	5070(1)	3905(2)	25(1)
C40	2671(4)	3198(1)	2433(2)	24(1)
C41	3415(5)	2620(2)	1100(3)	36(1)
C42	2496(5)	2136(2)	2452(3)	34(1)
C43	5127(4)	2596(2)	2729(3)	32(1)
O2	1498(4)	3458(1)	6925(2)	48(1)
C44	332(5)	3665(2)	6538(4)	49(1)
C45	318(6)	3677(2)	5664(3)	50(1)
C46	1575(6)	3448(2)	5590(4)	55(1)
C47	1946(6)	3182(2)	6348(4)	58(2)
O3	-685(7)	2903(2)	4261(3)	61(2)
C48	-1189(11)	2469(3)	3992(5)	55(2)
C49	-1025(9)	2405(3)	3168(5)	53(2)
C50	-977(10)	2913(3)	2881(5)	62(2)
C51	-344(12)	3156(4)	3626(6)	76(3)
O3'	-1680(20)	2820(6)	3915(11)	69(4)
C48'	-1110(30)	2414(7)	3650(20)	62(5)
C49'	-290(30)	2560(8)	3112(15)	61(5)
C50'	-250(20)	3092(8)	3174(19)	68(5)
C51'	-1450(20)	3200(7)	3434(15)	62(5)
O4	-2645(9)	2668(5)	-420(7)	131(4)
C52	-1347(18)	2552(9)	-152(13)	68(3)
C53	-850(11)	2819(4)	592(7)	68(2)

C54	-1976(18)	3097(8)	747(10)	71(3)
C55	-2998(13)	3017(5)	55(7)	88(3)
O4'	-1120(20)	2918(8)	1105(12)	86(5)
C52'	-470(30)	2678(15)	590(20)	80(7)
C53'	-1430(60)	2520(30)	-110(30)	72(8)
C54'	-2480(30)	2884(14)	-170(20)	87(6)
C55'	-2190(50)	3130(20)	600(30)	67(6)

Table 3. Bond lengths [Å] and angles [°] for neimca14.

Br(1)-Fe(1)	2.3918(7)	C(10)-C(11)	1.376(7)
Fe(1)-P(1)	2.4750(10)	C(11)-H(11)	0.9300
Fe(1)-P(2)	2.4828(10)	C(11)-C(12)	1.388(6)
Fe(1)-C(40)	2.109(4)	C(12)-H(12)	0.9300
P(1)-C(1)	1.817(4)	C(13)-C(14)	1.393(5)
P(1)-C(7)	1.823(4)	C(13)-C(18)	1.395(5)
P(1)-C(13)	1.828(4)	C(14)-H(14)	0.9300
P(2)-C(24)	1.825(4)	C(14)-C(15)	1.393(5)
P(2)-C(28)	1.826(4)	C(15)-H(15)	0.9300
P(2)-C(34)	1.825(4)	C(15)-C(16)	1.383(5)
Si(1)-C(40)	1.836(4)	C(16)-H(16)	0.9300
Si(1)-C(41)	1.870(5)	C(16)-C(17)	1.402(5)
Si(1)-C(42)	1.884(4)	C(17)-C(18)	1.387(5)
Si(1)-C(43)	1.871(5)	C(17)-C(25)	1.532(5)
O(1)-C(18)	1.381(4)	C(19)-C(20)	1.378(5)
O(1)-C(19)	1.377(4)	C(19)-C(24)	1.400(5)
C(1)-C(2)	1.399(5)	C(20)-C(21)	1.397(5)
C(1)-C(6)	1.396(5)	C(20)-C(25)	1.527(5)
C(2)-H(2)	0.9300	C(21)-H(21)	0.9300
C(2)-C(3)	1.384(6)	C(21)-C(22)	1.387(5)
C(3)-H(3)	0.9300	C(22)-H(22)	0.9300
C(3)-C(4)	1.381(7)	C(22)-C(23)	1.387(6)
C(4)-H(4)	0.9300	C(23)-H(23)	0.9300
C(4)-C(5)	1.384(7)	C(23)-C(24)	1.391(5)
C(5)-H(5)	0.9300	C(25)-C(26)	1.542(5)
C(5)-C(6)	1.394(6)	C(25)-C(27)	1.533(5)
C(6)-H(6)	0.9300	C(26)-H(26A)	0.9600
C(7)-C(8)	1.401(5)	C(26)-H(26B)	0.9600
C(7)-C(12)	1.378(6)	C(26)-H(26C)	0.9600
C(8)-H(8)	0.9300	C(27)-H(27A)	0.9600
C(8)-C(9)	1.379(6)	C(27)-H(27B)	0.9600
C(9)-H(9)	0.9300	C(27)-H(27C)	0.9600
C(9)-C(10)	1.387(7)	C(28)-C(29)	1.391(5)
C(10)-H(10)	0.9300	C(28)-C(33)	1.382(6)

C(29)-H(29)	0.9300	C(45)-H(45A)	0.9700
C(29)-C(30)	1.392(6)	C(45)-H(45B)	0.9700
C(30)-H(30)	0.9300	C(45)-C(46)	1.519(8)
C(30)-C(31)	1.376(8)	C(46)-H(46A)	0.9700
C(31)-H(31)	0.9300	C(46)-H(46B)	0.9700
C(31)-C(32)	1.387(8)	C(46)-C(47)	1.494(9)
C(32)-H(32)	0.9300	C(47)-H(47A)	0.9700
C(32)-C(33)	1.391(6)	C(47)-H(47B)	0.9700
C(33)-H(33)	0.9300	O(3)-C(48)	1.384(9)
C(34)-C(35)	1.390(6)	O(3)-C(51)	1.423(9)
C(34)-C(39)	1.406(5)	C(48)-H(48A)	0.9700
C(35)-H(35)	0.9300	C(48)-H(48B)	0.9700
C(35)-C(36)	1.388(6)	C(48)-C(49)	1.484(10)
C(36)-H(36)	0.9300	C(49)-H(49A)	0.9700
C(36)-C(37)	1.379(7)	C(49)-H(49B)	0.9700
C(37)-H(37)	0.9300	C(49)-C(50)	1.525(12)
C(37)-C(38)	1.375(7)	C(50)-H(50A)	0.9700
C(38)-H(38)	0.9300	C(50)-H(50B)	0.9700
C(38)-C(39)	1.391(6)	C(50)-C(51)	1.494(11)
C(39)-H(39)	0.9300	C(51)-H(51A)	0.9700
C(40)-H(40A)	0.9700	C(51)-H(51B)	0.9700
C(40)-H(40B)	0.9700	O(3')-C(48')	1.419(16)
C(41)-H(41A)	0.9600	O(3')-C(51')	1.413(15)
C(41)-H(41B)	0.9600	C(48')-H(48C)	0.9700
C(41)-H(41C)	0.9600	C(48')-H(48D)	0.9700
C(42)-H(42A)	0.9600	C(48')-C(49')	1.465(17)
C(42)-H(42B)	0.9600	C(49')-H(49C)	0.9700
C(42)-H(42C)	0.9600	C(49')-H(49D)	0.9700
C(43)-H(43A)	0.9600	C(49')-C(50')	1.51(2)
C(43)-H(43B)	0.9600	C(50')-H(50C)	0.9700
C(43)-H(43C)	0.9600	C(50')-H(50D)	0.9700
O(2)-C(44)	1.411(7)	C(50')-C(51')	1.474(17)
O(2)-C(47)	1.428(7)	C(51')-H(51C)	0.9700
C(44)-H(44A)	0.9700	C(51')-H(51D)	0.9700
C(44)-H(44B)	0.9700	O(4)-C(52)	1.404(15)
C(44)-C(45)	1.509(8)	O(4)-C(55)	1.386(13)

C(52)-H(52A)	0.9700	C(24)-P(2)-Fe(1)	114.80(11)
C(52)-H(52B)	0.9700	C(24)-P(2)-C(28)	102.78(17)
C(52)-C(53)	1.493(12)	C(24)-P(2)-C(34)	102.36(17)
C(53)-H(53A)	0.9700	C(28)-P(2)-Fe(1)	112.77(13)
C(53)-H(53B)	0.9700	C(34)-P(2)-Fe(1)	118.41(12)
C(53)-C(54)	1.51(2)	C(34)-P(2)-C(28)	103.87(17)
C(54)-H(54A)	0.9700	C(40)-Si(1)-C(41)	111.58(19)
C(54)-H(54B)	0.9700	C(40)-Si(1)-C(42)	108.4(2)
C(54)-C(55)	1.460(13)	C(40)-Si(1)-C(43)	112.30(19)
C(55)-H(55A)	0.9700	C(41)-Si(1)-C(42)	108.4(2)
C(55)-H(55B)	0.9700	C(41)-Si(1)-C(43)	107.9(2)
O(4')-C(52')	1.418(19)	C(43)-Si(1)-C(42)	108.2(2)
O(4')-C(55')	1.421(19)	C(19)-O(1)-C(18)	116.1(3)
C(52')-H(52C)	0.9700	C(2)-C(1)-P(1)	116.7(3)
C(52')-H(52D)	0.9700	C(6)-C(1)-P(1)	124.0(3)
C(52')-C(53')	1.486(18)	C(6)-C(1)-C(2)	119.3(4)
C(53')-H(53C)	0.9700	C(1)-C(2)-H(2)	119.7
C(53')-H(53D)	0.9700	C(3)-C(2)-C(1)	120.6(4)
C(53')-C(54')	1.52(3)	C(3)-C(2)-H(2)	119.7
C(54')-H(54C)	0.9700	C(2)-C(3)-H(3)	120.0
C(54')-H(54D)	0.9700	C(4)-C(3)-C(2)	119.9(4)
C(54')-C(55')	1.475(18)	C(4)-C(3)-H(3)	120.0
C(55')-H(55C)	0.9700	C(3)-C(4)-H(4)	120.0
C(55')-H(55D)	0.9700	C(3)-C(4)-C(5)	120.0(4)
Br(1)-Fe(1)-P(1)	103.20(3)	C(5)-C(4)-H(4)	120.0
Br(1)-Fe(1)-P(2)	104.12(3)	C(4)-C(5)-H(5)	119.6
P(1)-Fe(1)-P(2)	103.05(3)	C(4)-C(5)-C(6)	120.7(4)
C(40)-Fe(1)-Br(1)	126.73(10)	C(6)-C(5)-H(5)	119.6
C(40)-Fe(1)-P(1)	110.55(11)	C(1)-C(6)-H(6)	120.3
C(40)-Fe(1)-P(2)	106.78(11)	C(5)-C(6)-C(1)	119.4(4)
C(1)-P(1)-Fe(1)	112.83(12)	C(5)-C(6)-H(6)	120.3
C(1)-P(1)-C(7)	101.94(17)	C(8)-C(7)-P(1)	122.4(3)
C(1)-P(1)-C(13)	107.24(17)	C(12)-C(7)-P(1)	118.9(3)
C(7)-P(1)-Fe(1)	114.95(13)	C(12)-C(7)-C(8)	118.6(4)
C(7)-P(1)-C(13)	102.31(16)	C(7)-C(8)-H(8)	119.7
C(13)-P(1)-Fe(1)	116.10(11)	C(9)-C(8)-C(7)	120.5(4)

C(9)-C(8)-H(8)	119.7	C(21)-C(20)-C(25)	123.7(3)
C(8)-C(9)-H(9)	119.9	C(20)-C(21)-H(21)	119.5
C(8)-C(9)-C(10)	120.3(4)	C(22)-C(21)-C(20)	121.0(3)
C(10)-C(9)-H(9)	119.9	C(22)-C(21)-H(21)	119.5
C(9)-C(10)-H(10)	120.3	C(21)-C(22)-H(22)	119.5
C(11)-C(10)-C(9)	119.4(4)	C(23)-C(22)-C(21)	120.9(3)
C(11)-C(10)-H(10)	120.3	C(23)-C(22)-H(22)	119.5
C(10)-C(11)-H(11)	119.7	C(22)-C(23)-H(23)	120.3
C(10)-C(11)-C(12)	120.5(4)	C(22)-C(23)-C(24)	119.4(3)
C(12)-C(11)-H(11)	119.7	C(24)-C(23)-H(23)	120.3
C(7)-C(12)-C(11)	120.7(4)	C(19)-C(24)-P(2)	116.2(3)
C(7)-C(12)-H(12)	119.6	C(23)-C(24)-P(2)	125.6(3)
C(11)-C(12)-H(12)	119.6	C(23)-C(24)-C(19)	118.2(3)
C(14)-C(13)-P(1)	124.1(3)	C(17)-C(25)-C(26)	108.2(3)
C(14)-C(13)-C(18)	117.5(3)	C(17)-C(25)-C(27)	111.2(3)
C(18)-C(13)-P(1)	118.0(3)	C(20)-C(25)-C(17)	108.0(3)
C(13)-C(14)-H(14)	119.8	C(20)-C(25)-C(26)	107.9(3)
C(13)-C(14)-C(15)	120.3(3)	C(20)-C(25)-C(27)	111.8(3)
C(15)-C(14)-H(14)	119.8	C(27)-C(25)-C(26)	109.6(3)
C(14)-C(15)-H(15)	119.8	C(25)-C(26)-H(26A)	109.5
C(16)-C(15)-C(14)	120.4(3)	C(25)-C(26)-H(26B)	109.5
C(16)-C(15)-H(15)	119.8	C(25)-C(26)-H(26C)	109.5
C(15)-C(16)-H(16)	119.4	H(26A)-C(26)-H(26B)	109.5
C(15)-C(16)-C(17)	121.2(3)	H(26A)-C(26)-H(26C)	109.5
C(17)-C(16)-H(16)	119.4	H(26B)-C(26)-H(26C)	109.5
C(16)-C(17)-C(25)	124.0(3)	C(25)-C(27)-H(27A)	109.5
C(18)-C(17)-C(16)	116.6(3)	C(25)-C(27)-H(27B)	109.5
C(18)-C(17)-C(25)	119.4(3)	C(25)-C(27)-H(27C)	109.5
O(1)-C(18)-C(13)	115.4(3)	H(27A)-C(27)-H(27B)	109.5
O(1)-C(18)-C(17)	120.7(3)	H(27A)-C(27)-H(27C)	109.5
C(17)-C(18)-C(13)	124.0(3)	H(27B)-C(27)-H(27C)	109.5
O(1)-C(19)-C(20)	121.4(3)	C(29)-C(28)-P(2)	122.9(3)
O(1)-C(19)-C(24)	114.9(3)	C(33)-C(28)-P(2)	117.7(3)
C(20)-C(19)-C(24)	123.6(3)	C(33)-C(28)-C(29)	119.3(4)
C(19)-C(20)-C(21)	116.8(3)	C(28)-C(29)-H(29)	119.8
C(19)-C(20)-C(25)	119.3(3)	C(28)-C(29)-C(30)	120.3(4)

C(30)-C(29)-H(29)	119.8	H(40A)-C(40)-H(40B)	107.8
C(29)-C(30)-H(30)	120.2	Si(1)-C(41)-H(41A)	109.5
C(31)-C(30)-C(29)	119.6(4)	Si(1)-C(41)-H(41B)	109.5
C(31)-C(30)-H(30)	120.2	Si(1)-C(41)-H(41C)	109.5
C(30)-C(31)-H(31)	119.6	H(41A)-C(41)-H(41B)	109.5
C(30)-C(31)-C(32)	120.8(4)	H(41A)-C(41)-H(41C)	109.5
C(32)-C(31)-H(31)	119.6	H(41B)-C(41)-H(41C)	109.5
C(31)-C(32)-H(32)	120.4	Si(1)-C(42)-H(42A)	109.5
C(31)-C(32)-C(33)	119.2(5)	Si(1)-C(42)-H(42B)	109.5
C(33)-C(32)-H(32)	120.4	Si(1)-C(42)-H(42C)	109.5
C(28)-C(33)-C(32)	120.7(5)	H(42A)-C(42)-H(42B)	109.5
C(28)-C(33)-H(33)	119.6	H(42A)-C(42)-H(42C)	109.5
C(32)-C(33)-H(33)	119.6	H(42B)-C(42)-H(42C)	109.5
C(35)-C(34)-P(2)	118.6(3)	Si(1)-C(43)-H(43A)	109.5
C(35)-C(34)-C(39)	119.5(4)	Si(1)-C(43)-H(43B)	109.5
C(39)-C(34)-P(2)	121.9(3)	Si(1)-C(43)-H(43C)	109.5
C(34)-C(35)-H(35)	120.2	H(43A)-C(43)-H(43B)	109.5
C(36)-C(35)-C(34)	119.6(4)	H(43A)-C(43)-H(43C)	109.5
C(36)-C(35)-H(35)	120.2	H(43B)-C(43)-H(43C)	109.5
C(35)-C(36)-H(36)	119.8	C(44)-O(2)-C(47)	106.5(4)
C(37)-C(36)-C(35)	120.4(4)	O(2)-C(44)-H(44A)	110.1
C(37)-C(36)-H(36)	119.8	O(2)-C(44)-H(44B)	110.1
C(36)-C(37)-H(37)	119.6	O(2)-C(44)-C(45)	108.1(4)
C(38)-C(37)-C(36)	120.9(4)	H(44A)-C(44)-H(44B)	108.4
C(38)-C(37)-H(37)	119.6	C(45)-C(44)-H(44A)	110.1
C(37)-C(38)-H(38)	120.2	C(45)-C(44)-H(44B)	110.1
C(37)-C(38)-C(39)	119.6(4)	C(44)-C(45)-H(45A)	110.9
C(39)-C(38)-H(38)	120.2	C(44)-C(45)-H(45B)	110.9
C(34)-C(39)-H(39)	120.0	C(44)-C(45)-C(46)	104.5(5)
C(38)-C(39)-C(34)	120.0(4)	H(45A)-C(45)-H(45B)	108.9
C(38)-C(39)-H(39)	120.0	C(46)-C(45)-H(45A)	110.9
Fe(1)-C(40)-H(40A)	109.1	C(46)-C(45)-H(45B)	110.9
Fe(1)-C(40)-H(40B)	109.1	C(45)-C(46)-H(46A)	111.2
Si(1)-C(40)-Fe(1)	112.47(19)	C(45)-C(46)-H(46B)	111.2
Si(1)-C(40)-H(40A)	109.1	H(46A)-C(46)-H(46B)	109.2
Si(1)-C(40)-H(40B)	109.1	C(47)-C(46)-C(45)	102.6(5)

C(47)-C(46)-H(46A)	111.2	O(3')-C(48')-C(49')	109.0(13)
C(47)-C(46)-H(46B)	111.2	H(48C)-C(48')-H(48D)	108.3
O(2)-C(47)-C(46)	105.8(5)	C(49')-C(48')-H(48C)	109.9
O(2)-C(47)-H(47A)	110.6	C(49')-C(48')-H(48D)	109.9
O(2)-C(47)-H(47B)	110.6	C(48')-C(49')-H(49C)	110.8
C(46)-C(47)-H(47A)	110.6	C(48')-C(49')-H(49D)	110.8
C(46)-C(47)-H(47B)	110.6	C(48')-C(49')-C(50')	104.5(12)
H(47A)-C(47)-H(47B)	108.7	H(49C)-C(49')-H(49D)	108.9
C(48)-O(3)-C(51)	109.4(7)	C(50')-C(49')-H(49C)	110.8
O(3)-C(48)-H(48A)	110.0	C(50')-C(49')-H(49D)	110.8
O(3)-C(48)-H(48B)	110.0	C(49')-C(50')-H(50C)	111.3
O(3)-C(48)-C(49)	108.5(7)	C(49')-C(50')-H(50D)	111.3
H(48A)-C(48)-H(48B)	108.4	H(50C)-C(50')-H(50D)	109.2
C(49)-C(48)-H(48A)	110.0	C(51')-C(50')-C(49')	102.4(13)
C(49)-C(48)-H(48B)	110.0	C(51')-C(50')-H(50C)	111.3
C(48)-C(49)-H(49A)	111.3	C(51')-C(50')-H(50D)	111.3
C(48)-C(49)-H(49B)	111.3	O(3')-C(51')-C(50')	106.7(14)
C(48)-C(49)-C(50)	102.5(6)	O(3')-C(51')-H(51C)	110.4
H(49A)-C(49)-H(49B)	109.2	O(3')-C(51')-H(51D)	110.4
C(50)-C(49)-H(49A)	111.3	C(50')-C(51')-H(51C)	110.4
C(50)-C(49)-H(49B)	111.3	C(50')-C(51')-H(51D)	110.4
C(49)-C(50)-H(50A)	111.5	H(51C)-C(51')-H(51D)	108.6
C(49)-C(50)-H(50B)	111.5	C(55)-O(4)-C(52)	110.0(12)
H(50A)-C(50)-H(50B)	109.3	O(4)-C(52)-H(52A)	110.0
C(51)-C(50)-C(49)	101.2(7)	O(4)-C(52)-H(52B)	110.0
C(51)-C(50)-H(50A)	111.5	O(4)-C(52)-C(53)	108.4(12)
C(51)-C(50)-H(50B)	111.5	H(52A)-C(52)-H(52B)	108.4
O(3)-C(51)-C(50)	107.0(7)	C(53)-C(52)-H(52A)	110.0
O(3)-C(51)-H(51A)	110.3	C(53)-C(52)-H(52B)	110.0
O(3)-C(51)-H(51B)	110.3	C(52)-C(53)-H(53A)	110.7
C(50)-C(51)-H(51A)	110.3	C(52)-C(53)-H(53B)	110.7
C(50)-C(51)-H(51B)	110.3	C(52)-C(53)-C(54)	105.3(10)
H(51A)-C(51)-H(51B)	108.6	H(53A)-C(53)-H(53B)	108.8
C(51')-O(3')-C(48')	107.0(14)	C(54)-C(53)-H(53A)	110.7
O(3')-C(48')-H(48C)	109.9	C(54)-C(53)-H(53B)	110.7
O(3')-C(48')-H(48D)	109.9	C(53)-C(54)-H(54A)	110.7

C(53)-C(54)-H(54B)	110.7	C(52')-C(53')-H(53C)	111.2
H(54A)-C(54)-H(54B)	108.8	C(52')-C(53')-H(53D)	111.2
C(55)-C(54)-C(53)	105.1(11)	C(52')-C(53')-C(54')	102.7(17)
C(55)-C(54)-H(54A)	110.7	H(53C)-C(53')-H(53D)	109.1
C(55)-C(54)-H(54B)	110.7	C(54')-C(53')-H(53C)	111.2
O(4)-C(55)-C(54)	110.4(13)	C(54')-C(53')-H(53D)	111.2
O(4)-C(55)-H(55A)	109.6	C(53')-C(54')-H(54C)	110.8
O(4)-C(55)-H(55B)	109.6	C(53')-C(54')-H(54D)	110.8
C(54)-C(55)-H(55A)	109.6	H(54C)-C(54')-H(54D)	108.9
C(54)-C(55)-H(55B)	109.6	C(55')-C(54')-C(53')	104.5(15)
H(55A)-C(55)-H(55B)	108.1	C(55')-C(54')-H(54C)	110.8
C(52')-O(4')-C(55')	105(2)	C(55')-C(54')-H(54D)	110.8
O(4')-C(52')-H(52C)	110.0	O(4')-C(55')-C(54')	110(2)
O(4')-C(52')-H(52D)	110.0	O(4')-C(55')-H(55C)	109.6
O(4')-C(52')-C(53')	108(2)	O(4')-C(55')-H(55D)	109.6
H(52C)-C(52')-H(52D)	108.4	C(54')-C(55')-H(55C)	109.6
C(53')-C(52')-H(52C)	110.0	C(54')-C(55')-H(55D)	109.6
C(53')-C(52')-H(52D)	110.0	H(55C)-C(55')-H(55D)	108.1

Table 4. Anisotropic displacement parameters ($Å^2$x 10^3) for neimca14. The anisotropic displacement factor exponent takes the form: $-2\pi^2[h^2 a^{*2}U_{11} + ... + 2 h k a^* b^* U_{12}]$

	U_{11}	U_{22}	U_{33}	U_{23}	U_{13}	U_{12}
Br1	22(1)	39(1)	34(1)	4(1)	11(1)	-2(1)
Fe1	15(1)	18(1)	18(1)	1(1)	4(1)	-1(1)
P1	13(1)	20(1)	19(1)	1(1)	2(1)	0(1)
P2	18(1)	20(1)	16(1)	1(1)	3(1)	0(1)
Si1	26(1)	21(1)	28(1)	2(1)	6(1)	-3(1)
O1	16(1)	25(1)	15(1)	2(1)	2(1)	-4(1)
C1	16(2)	27(2)	19(2)	2(1)	2(1)	2(1)
C2	19(2)	29(2)	31(2)	1(2)	6(2)	0(2)
C3	17(2)	45(2)	30(2)	3(2)	3(2)	0(2)
C4	22(2)	52(3)	26(2)	-6(2)	5(2)	7(2)
C5	27(2)	36(2)	32(2)	-9(2)	3(2)	10(2)
C6	21(2)	28(2)	26(2)	-3(1)	1(1)	2(2)
C7	18(2)	25(2)	22(2)	1(1)	3(1)	-8(1)
C8	20(2)	26(2)	28(2)	0(2)	-1(1)	-2(2)
C9	26(2)	34(2)	29(2)	2(2)	-4(2)	-7(2)
C10	35(2)	41(2)	26(2)	-8(2)	4(2)	-13(2)
C11	35(2)	42(2)	34(2)	-15(2)	12(2)	-7(2)
C12	22(2)	33(2)	29(2)	-5(2)	5(2)	-2(2)
C13	13(2)	23(2)	18(2)	3(1)	2(1)	1(1)
C14	22(2)	24(2)	22(2)	0(1)	2(1)	1(2)
C15	26(2)	26(2)	24(2)	6(1)	3(1)	1(2)
C16	25(2)	21(2)	21(2)	5(1)	8(1)	-2(2)
C17	16(2)	20(2)	19(2)	0(1)	4(1)	1(1)
C18	16(2)	22(2)	15(2)	3(1)	5(1)	2(1)
C19	13(2)	20(2)	21(2)	-2(1)	4(1)	-1(1)
C20	17(2)	22(2)	23(2)	-2(1)	7(1)	2(1)
C21	17(2)	24(2)	25(2)	-2(1)	5(1)	-2(1)
C22	15(2)	34(2)	24(2)	-2(2)	-1(1)	1(2)
C23	19(2)	27(2)	21(2)	1(1)	5(1)	4(1)
C24	15(2)	23(2)	18(2)	-1(1)	4(1)	0(1)
C25	20(2)	22(2)	20(2)	0(1)	5(1)	-2(1)

C26	27(2)	25(2)	27(2)	-1(1)	2(2)	3(2)
C27	27(2)	33(2)	26(2)	2(2)	5(2)	-11(2)
C28	29(2)	22(2)	19(2)	0(1)	3(1)	-4(2)
C29	31(2)	31(2)	22(2)	4(2)	5(2)	-2(2)
C30	49(3)	38(2)	23(2)	7(2)	2(2)	-5(2)
C31	63(3)	32(2)	30(2)	8(2)	-12(2)	5(2)
C32	66(4)	44(3)	38(3)	0(2)	-1(2)	29(3)
C33	48(3)	39(2)	27(2)	2(2)	-2(2)	16(2)
C34	18(2)	29(2)	19(2)	1(1)	4(1)	2(2)
C35	25(2)	36(2)	27(2)	3(2)	3(2)	-5(2)
C36	21(2)	56(3)	32(2)	5(2)	7(2)	-3(2)
C37	25(2)	57(3)	28(2)	3(2)	10(2)	16(2)
C38	29(2)	40(2)	25(2)	-3(2)	6(2)	10(2)
C39	22(2)	29(2)	25(2)	-1(1)	6(1)	2(2)
C40	25(2)	23(2)	24(2)	1(1)	7(1)	10(2)
C41	43(3)	30(2)	34(2)	-2(2)	5(2)	-2(2)
C42	39(2)	26(2)	37(2)	3(2)	6(2)	-6(2)
C43	32(2)	29(2)	35(2)	4(2)	3(2)	3(2)
O2	48(2)	42(2)	51(2)	3(2)	7(2)	-8(2)
C44	36(3)	51(3)	62(3)	-17(3)	14(2)	-5(2)
C45	49(3)	51(3)	50(3)	1(2)	14(2)	6(3)
C46	53(3)	54(3)	60(4)	-13(3)	15(3)	3(3)
C47	56(4)	50(3)	68(4)	1(3)	14(3)	10(3)
O3	79(4)	59(3)	53(3)	-17(2)	30(3)	-30(3)
C48	70(5)	44(4)	55(5)	-3(3)	21(4)	-5(4)
C49	49(5)	51(4)	63(5)	-20(3)	22(4)	-5(3)
C50	63(5)	73(5)	53(4)	2(4)	18(4)	-13(4)
C51	92(7)	75(5)	65(5)	-3(4)	29(5)	-39(5)
O3'	88(10)	66(8)	65(8)	3(7)	44(7)	4(8)
C48'	80(11)	53(8)	64(12)	10(9)	38(10)	4(8)
C49'	54(11)	72(9)	62(11)	-7(9)	29(9)	-1(9)
C50'	70(10)	73(9)	66(11)	-2(9)	26(9)	-18(8)
C51'	72(11)	56(8)	64(11)	5(8)	24(9)	1(9)
O4	73(5)	213(11)	107(7)	-73(7)	13(4)	-9(6)
C52	75(6)	69(6)	71(6)	-5(5)	37(5)	-22(5)
C53	79(6)	70(7)	59(5)	5(4)	27(5)	0(5)

C54	61(7)	74(7)	79(7)	-17(7)	16(5)	-15(5)
C55	93(7)	97(8)	66(6)	3(5)	-1(5)	4(6)
O4'	80(10)	96(11)	78(9)	-16(8)	10(7)	20(9)
C52'	90(13)	76(16)	74(11)	2(11)	17(9)	22(11)
C53'	79(13)	81(15)	68(12)	-5(11)	41(11)	-5(11)
C54'	58(11)	108(13)	91(11)	-53(10)	8(9)	-8(10)
C55'	63(12)	73(11)	68(11)	-14(9)	16(9)	-5(9)

Table 5. Hydrogen coordinates (x 10^4) and isotropic displacement parameters (Å^2 x 10^3) for neimca14.

	x	y	z	U(eq)
H2	103	3834	1737	31
H3	-1688	4031	2223	37
H4	-2152	4814	2409	40
H5	-808	5399	2130	39
H6	976	5211	1618	30
H8	-451	4346	127	31
H9	-1387	3965	-1025	37
H10	-239	3410	-1577	41
H11	1831	3228	-951	44
H12	2761	3596	219	34
H14	2022	4756	-351	27
H15	3150	5374	-760	30
H16	4532	5822	150	26
H21	7193	5824	2992	27
H22	7770	5356	4096	30
H23	6556	4705	4277	26
H26A	4739	6368	2426	40
H26B	3554	6029	2224	40
H26C	3878	6365	1573	40
H27A	5853	6226	1041	43
H27B	6700	5778	1292	43
H27C	6721	6194	1890	43
H29	4077	4256	5029	34
H30	5212	3829	6091	45
H31	6750	3296	5899	54
H32	7217	3201	4659	61
H33	6026	3609	3591	48
H35	1655	4013	3566	35
H36	-49	4369	3980	43
H37	54	5152	4335	43

H38	1849	5594	4295	37
H39	3578	5246	3890	30
H40A	2691	3201	2995	29
H40B	1780	3202	2158	29
H41A	2556	2663	805	54
H41B	3725	2316	979	54
H41C	3957	2862	963	54
H42A	2510	2143	3008	52
H42B	2878	1847	2323	52
H42C	1625	2153	2163	52
H43A	5626	2849	2581	49
H43B	5470	2300	2599	49
H43C	5158	2609	3286	49
H44A	261	3982	6733	59
H44B	-385	3481	6636	59
H45A	271	3999	5471	59
H45B	-406	3501	5370	59
H46A	1457	3237	5140	66
H46B	2213	3683	5540	66
H47A	1551	2872	6302	69
H47B	2869	3144	6492	69
H48A	-2091	2455	4010	66
H48B	-747	2219	4324	66
H49A	-1742	2235	2854	64
H49B	-237	2238	3152	64
H50A	-472	2938	2478	74
H50B	-1829	3037	2679	74
H51A	579	3156	3677	91
H51B	-635	3480	3624	91
H48C	-1779	2204	3377	74
H48D	-611	2246	4097	74
H49C	-650	2461	2576	73
H49D	561	2427	3274	73
H50C	494	3197	3558	82
H50D	-237	3236	2667	82
H51C	-1361	3493	3731	75

H51D	-2151	3233	2982	75
H52A	-864	2636	-549	82
H52B	-1257	2216	-55	82
H53A	-158	3028	529	81
H53B	-535	2604	1025	81
H54A	-2229	2987	1222	85
H54B	-1767	3430	806	85
H55A	-3774	2923	223	106
H55B	-3175	3307	-244	106
H52C	-6	2410	854	96
H52D	146	2888	422	96
H53C	-1745	2205	-29	87
H53D	-1074	2516	-584	87
H54C	-2464	3102	-598	104
H54D	-3317	2735	-255	104
H55C	-2012	3459	519	81
H55D	-2933	3113	843	81

Table 6. Torsion angles [°] for neimca14.

Fe1-P1-C1-C2	-62.2(3)	C3-C4-C5-C6	1.3(6)
Fe1-P1-C1-C6	117.8(3)	C4-C5-C6-C1	-0.4(6)
Fe1-P1-C7-C8	147.4(3)	C6-C1-C2-C3	1.7(6)
Fe1-P1-C7-C12	-34.8(3)	C7-P1-C1-C2	61.6(3)
Fe1-P1-C13-C14	127.2(3)	C7-P1-C1-C6	-118.4(3)
Fe1-P1-C13-C18	-45.5(3)	C7-P1-C13-C14	1.2(4)
Fe1-P2-C24-C19	52.2(3)	C7-P1-C13-C18	-171.5(3)
Fe1-P2-C24-C23	-128.5(3)	C7-C8-C9-C10	-1.0(6)
Fe1-P2-C28-C29	-140.8(3)	C8-C7-C12-C11	0.2(6)
Fe1-P2-C28-C33	40.1(4)	C8-C9-C10-C11	0.7(7)
Fe1-P2-C34-C35	45.8(3)	C9-C10-C11-C12	0.1(7)
Fe1-P2-C34-C39	-133.6(3)	C10-C11-C12-C7	-0.5(7)
P1-C1-C2-C3	-178.3(3)	C12-C7-C8-C9	0.6(6)
P1-C1-C6-C5	178.9(3)	C13-P1-C1-C2	168.7(3)
P1-C7-C8-C9	178.4(3)	C13-P1-C1-C6	-11.3(4)
P1-C7-C12-C11	-177.7(3)	C13-P1-C7-C8	-85.9(3)
P1-C13-C14-C15	-172.0(3)	C13-P1-C7-C12	91.9(3)
P1-C13-C18-O1	-7.6(4)	C13-C14-C15-C16	-0.4(6)
P1-C13-C18-C17	173.0(3)	C14-C13-C18-O1	179.3(3)
P2-C28-C29-C30	-179.2(3)	C14-C13-C18-C17	-0.1(5)
P2-C28-C33-C32	177.9(4)	C14-C15-C16-C17	-0.5(6)
P2-C34-C35-C36	-179.1(3)	C15-C16-C17-C18	1.0(5)
P2-C34-C39-C38	178.9(3)	C15-C16-C17-C25	-175.9(3)
O1-C19-C20-C21	174.9(3)	C16-C17-C18-O1	179.9(3)
O1-C19-C20-C25	-0.7(5)	C16-C17-C18-C13	-0.7(5)
O1-C19-C24-P2	4.0(4)	C16-C17-C25-C20	-152.6(3)
O1-C19-C24-C23	-175.4(3)	C16-C17-C25-C26	90.8(4)
C1-P1-C7-C8	25.0(4)	C16-C17-C25-C27	-29.6(5)
C1-P1-C7-C12	-157.2(3)	C18-O1-C19-C20	31.9(5)
C1-P1-C13-C14	-105.6(3)	C18-O1-C19-C24	-150.8(3)
C1-P1-C13-C18	81.7(3)	C18-C13-C14-C15	0.7(5)
C1-C2-C3-C4	-0.9(6)	C18-C17-C25-C20	30.5(4)
C2-C1-C6-C5	-1.0(6)	C18-C17-C25-C26	-86.1(4)
C2-C3-C4-C5	-0.6(6)	C18-C17-C25-C27	153.6(3)

C19-O1-C18-C13	150.8(3)	C34-P2-C28-C29	-11.4(4)
C19-O1-C18-C17	-29.7(5)	C34-P2-C28-C33	169.6(4)
C19-C20-C21-C22	0.8(5)	C34-C35-C36-C37	0.1(6)
C19-C20-C25-C17	-28.7(4)	C35-C34-C39-C38	-0.5(6)
C19-C20-C25-C26	88.0(4)	C35-C36-C37-C38	-0.3(7)
C19-C20-C25-C27	-151.4(3)	C36-C37-C38-C39	0.1(6)
C20-C19-C24-P2	-178.7(3)	C37-C38-C39-C34	0.3(6)
C20-C19-C24-C23	1.9(5)	C39-C34-C35-C36	0.3(6)
C20-C21-C22-C23	0.8(6)	C41-Si1-C40-Fe1	-59.2(3)
C21-C20-C25-C17	156.0(3)	C42-Si1-C40-Fe1	-178.5(2)
C21-C20-C25-C26	-87.2(4)	C43-Si1-C40-Fe1	62.1(2)
C21-C20-C25-C27	33.4(5)	O2-C44-C45-C46	1.2(6)
C21-C22-C23-C24	-1.2(6)	C44-O2-C47-C46	35.5(6)
C22-C23-C24-P2	-179.4(3)	C44-C45-C46-C47	19.2(6)
C22-C23-C24-C19	-0.2(5)	C45-C46-C47-O2	-33.5(6)
C24-P2-C28-C29	95.0(4)	C47-O2-C44-C45	-22.6(6)
C24-P2-C28-C33	-84.0(4)	O3-C48-C49-C50	-25.7(10)
C24-P2-C34-C35	173.2(3)	C48-O3-C51-C50	14.9(13)
C24-P2-C34-C39	-6.2(3)	C48-C49-C50-C51	32.8(10)
C24-C19-C20-C21	-2.2(5)	C49-C50-C51-O3	-29.7(11)
C24-C19-C20-C25	-177.8(3)	C51-O3-C48-C49	7.3(12)
C25-C17-C18-O1	-3.0(5)	O3'-C48'-C49'-C50'	8(4)
C25-C17-C18-C13	176.4(3)	C48'-O3'-C51'-C50'	-29(3)
C25-C20-C21-C22	176.2(3)	C48'-C49'-C50'-C51'	-24(3)
C28-P2-C24-C19	175.1(3)	C49'-C50'-C51'-O3'	33(3)
C28-P2-C24-C23	-5.7(4)	C51'-O3'-C48'-C49'	12(4)
C28-P2-C34-C35	-80.1(3)	O4-C52-C53-C54	1(3)
C28-P2-C34-C39	100.5(3)	C52-O4-C55-C54	-9(2)
C28-C29-C30-C31	0.3(7)	C52-C53-C54-C55	-6(2)
C29-C28-C33-C32	-1.2(7)	C53-C54-C55-O4	9(2)
C29-C30-C31-C32	0.8(8)	C55-O4-C52-C53	5(3)
C30-C31-C32-C33	-2.1(9)	O4'-C52'-C53'-C54'	29(7)
C31-C32-C33-C28	2.3(9)	C52'-O4'-C55'-C54'	21(7)
C33-C28-C29-C30	-0.1(6)	C52'-C53'-C54'-C55'	-15(7)
C34-P2-C24-C19	-77.4(3)	C53'-C54'-C55'-O4'	-3(8)
C34-P2-C24-C23	101.9(3)	C55'-O4'-C52'-C53'	-32(6)

3.3 Monoalkylated Fe(II)-Xantphos (2-Me)

CRYSTAL STRUCTURE REPORT

C_{60} H_{75} Br Fe O_6 P_2

or

(Xantphos)FeBrMe · 5THF

Report prepared for:

M. C. Aguilera, Prof. M. Neidig

July 01, 2020

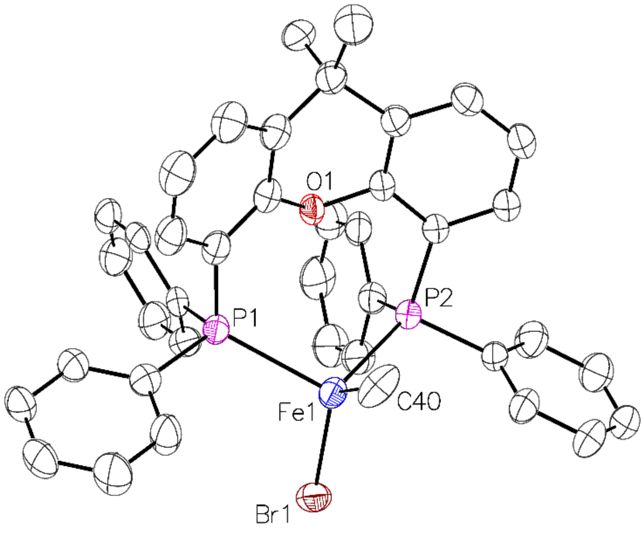

William W. Brennessel

X-ray Crystallographic Facility

Department of Chemistry, University of Rochester

120 Trustee Road

Rochester, NY 14627

Data collection

A crystal (0.185 x 0.071 x 0.066 mm^3) was placed onto a thin glass optical fiber or a nylon loop and mounted on a Rigaku XtaLab Synergy-S Dualflex diffractometer equipped with a HyPix-6000HE HPC area detector for data collection at 100.00(10) K. A preliminary set of cell constants and an orientation matrix were calculated from a small sampling of reflections.[1] A short pre-experiment was run, from which an optimal data collection strategy was determined. The full data collection was carried out using a PhotonJet (Cu) X-ray source with frame times of 1.24 and 4.96 seconds and a detector distance of 31.2 mm. Series of frames were collected in 0.50º steps in ω at different 2θ, κ, and ϕ settings. After the intensity data were corrected for absorption, the final cell constants were calculated from the xyz centroids of 21622 strong reflections from the actual data collection after integration.[1] See Table 1 for additional crystal and refinement information.

Structure solution and refinement

The structure was solved using[2] and refined using SHELXL.[3] The space group *P*-1 was determined based on intensity statistics. Most or all non-hydrogen atoms were assigned from the solution. Full-matrix least squares / difference Fourier cycles were performed which located any remaining non-hydrogen atoms. All non-hydrogen atoms were refined with anisotropic displacement parameters. All hydrogen atoms were placed in ideal positions and refined as riding atoms with relative isotropic displacement parameters. The final full matrix least squares refinement converged to $R1 = 0.0553$ (F^2, $I > 2\sigma(I)$) and $wR2 = 0.1554$ (F^2, all data).

Structure description

The structure is the one suggested. The asymmetric unit contains one iron molecule and five cocrystallized THF solvent molecules in general positions. The bromido and methyl ligands are modeled as disordered with each other (0.66:0.34). When the disorder ratios of the two sites are modeled independently, they confirm one bromido and one methyl ligand. Anisotropic displacement parameters for the two element types (i.e., Br and C) of the terminal ligand disorder were constrained to be equivalent at each site. No other constraints or restraints were employed in this disorder model because of the asymmetry of the Xantphos ligand. Three phenyl groups are modeled as disordered over two positions each: C1-C6, 0.60:0.40, C7-C12, 0.55:0.45, C28-C33, 0.52:0.48. Four of the five cocrystallized THF solvent molecules were modeled as disordered over two positions each: O3/C45-C48, 0.57:0.43, O4/C49-C52, 0.69:0.31, O5/C53-C56, 0.57:0.43, O6/C57-C60, 0.58:0.42. Although *R*1 (strong data) decreased by nearly 1% by application of the SQUEEZE routine of program Platon,[4] it was felt best to retain the solvent molecules, since they could be given reasonable disorder models.

Structure manipulation and figure generation were performed using Olex2.[5] Unless noted otherwise all structural diagrams containing anisotropic displacement ellipsoids are drawn at the 50 % probability level.

Data collection, structure solution, and structure refinement were conducted at the X-ray Crystallographic Facility,

B04 Hutchison Hall, Department of Chemistry, University of Rochester. The instrument was purchased with funding from NSF MRI program grant CHE-1725028. All publications arising from this report MUST either 1) include William W. Brennessel as a coauthor or 2) acknowledge William W. Brennessel and the X-ray Crystallographic Facility of the Department of Chemistry at the University of Rochester.

---

[1] *CrysAlisPro*, version 171.40.81a; Rigaku Corporation: Oxford, UK, 2020.

[2] Sheldrick, G. M. *SHELXT*, version 2018/2; *Acta. Crystallogr.* **2015**, *A71*, 3-8.

[3] Sheldrick, G. M. *SHELXL*, version 2018/3; *Acta. Crystallogr.* **2015**, *C71*, 3-8.

[4] Spek, A. L. *PLATON*, version 250420; *Acta. Crystallogr.* **2015**, *C71*, 9-18.

[5] Dolomanov, O. V.; Bourhis, L. J.; Gildea, R. J.; Howard, J. A. K.; Puschmann, H. *Olex2*, version 1.2-ac3; *J. Appl. Cryst.* **2009**, *42*, 339-341.

Some equations of interest:

$$R_{int} = \Sigma\, |F_o^2 - \langle F_o^2 \rangle| \,/\, \Sigma |F_o^2|$$

$$R1 = \Sigma ||F_o| - |F_c|| \,/\, \Sigma |F_o|$$

$$wR2 = [\Sigma[w(F_o^2 - F_c^2)^2] \,/\, \Sigma\, [w(F_o^2)^2]]^{1/2}$$

where $w = 1 / [\sigma^2 (F_o^2) + (aP)^2 + bP]$ and

$$P = 1/3 \max (0, F_o^2) + 2/3\, F_c^2$$

$$\mathrm{GOF} = S = [\Sigma[w(F_o^2 - F_c^2)^2] \,/\, (m-n)]^{1/2}$$

where m = number of reflections and n = number of parameters

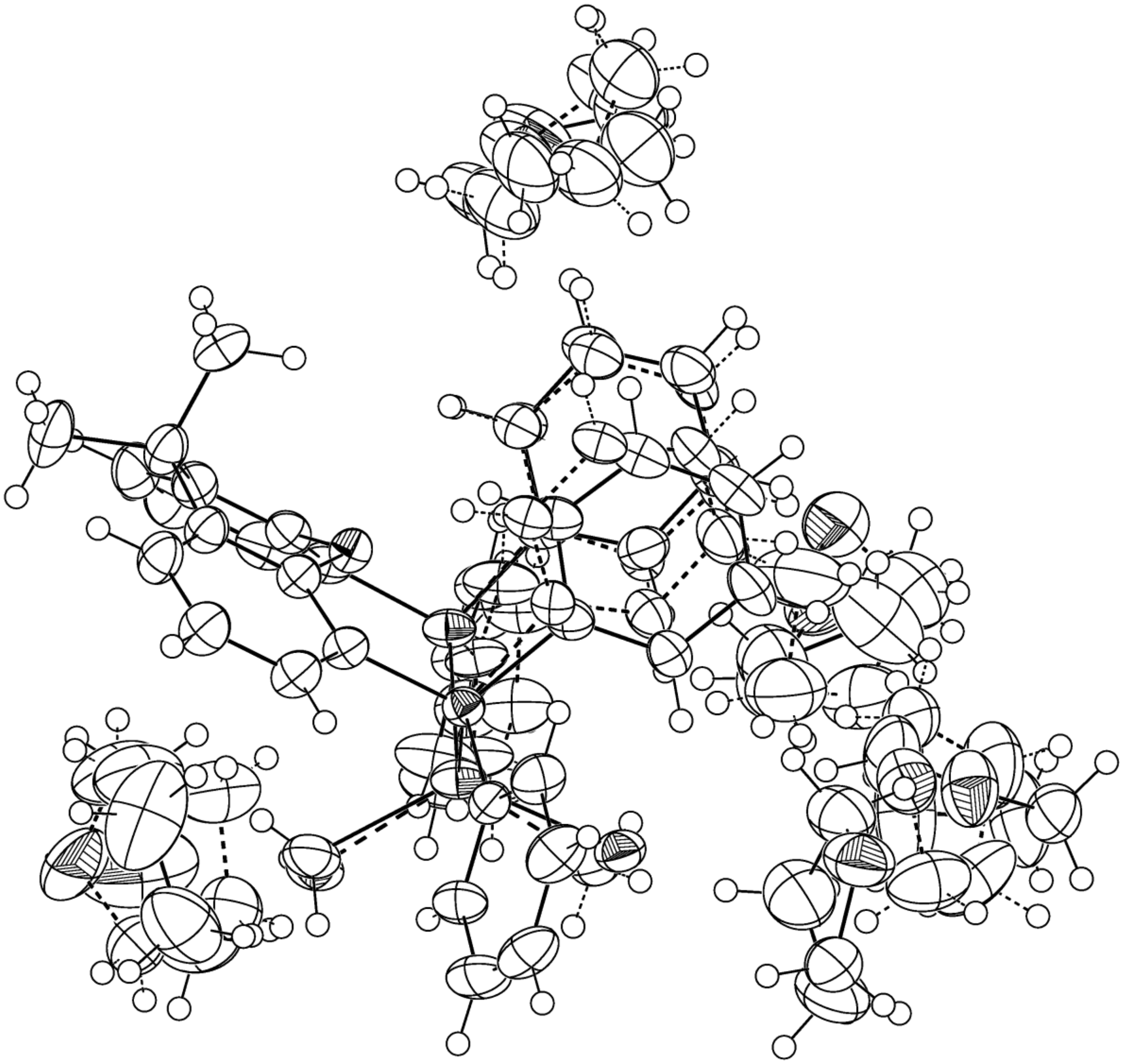

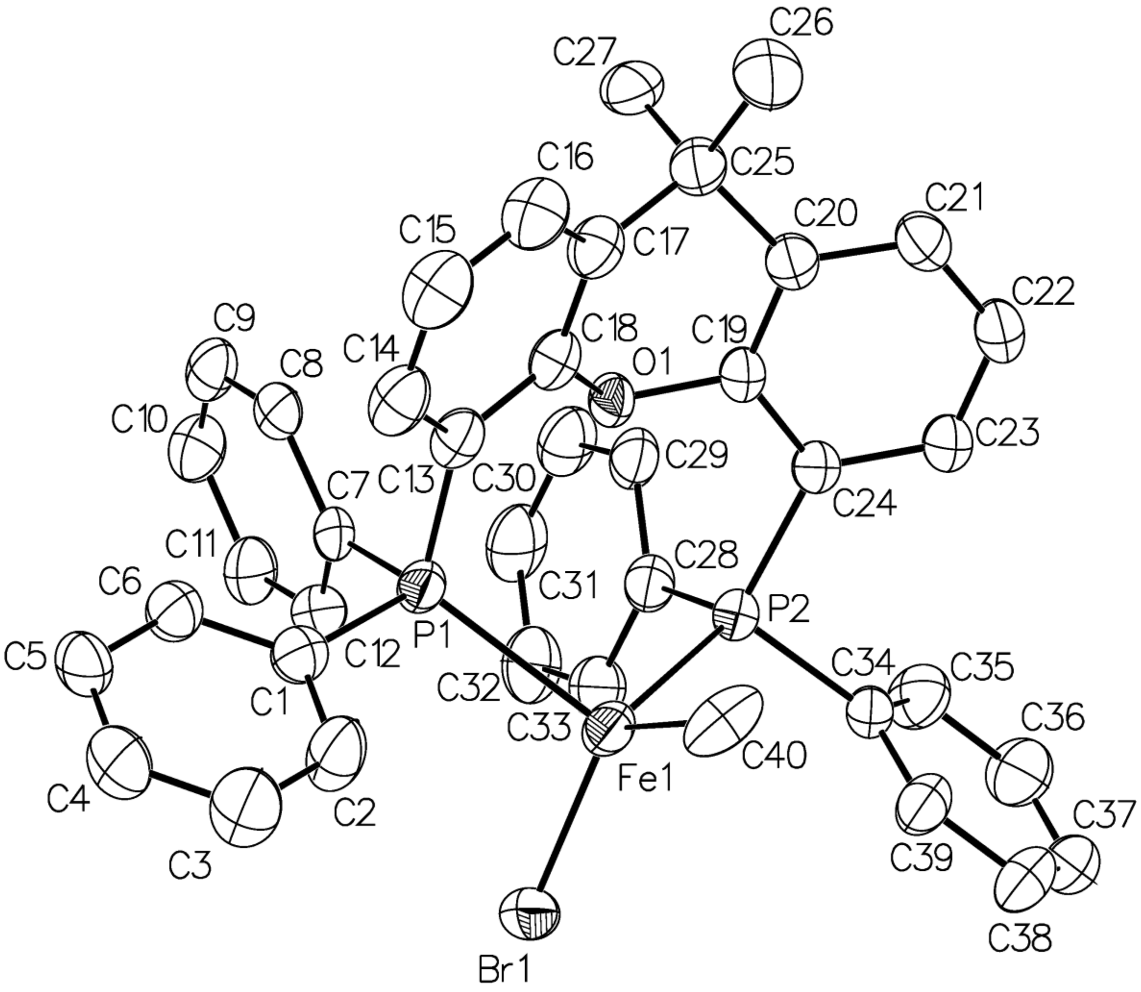
C26
C27
C25
C16
C21
C20
C15
C17
C22
C18
C19
C9
C14
O1
C8
C10
C23
C29
C30
C13
C7
C24
C11
C28
C31
C6
P2
C5
P1
C34
C35
C12
C32
C1
C36
C33
C40
Fe1
C4
C2
C37
C39
C3
C38
Br1

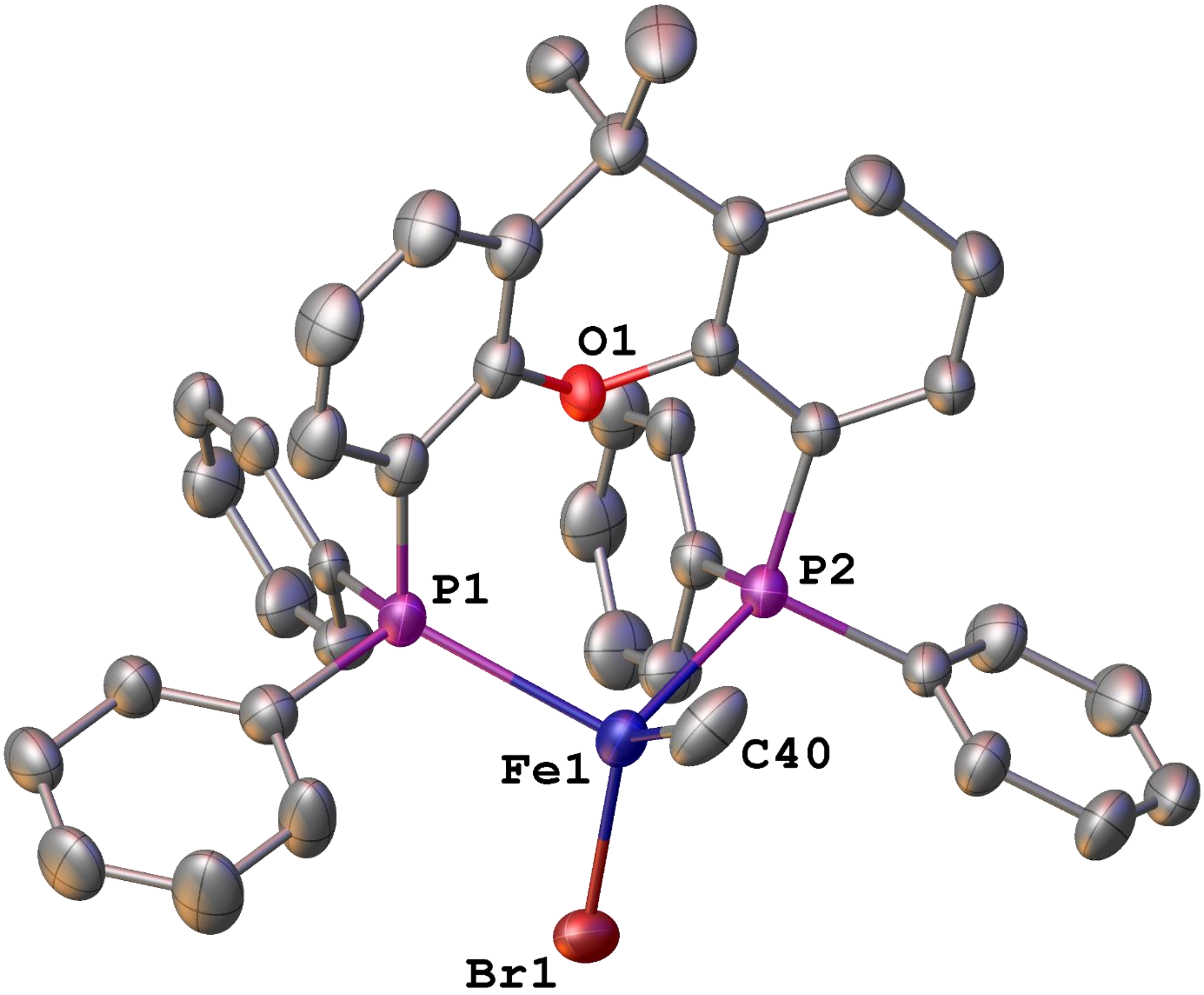
O1
P1
P2
C40
Fe1
Br1

Table 1. Crystal data and structure refinement for neimca08.

Identification code	neimca08	
Empirical formula	C60 H75 Br Fe O6 P2	
Formula weight	1089.90	
Temperature	100.00(10) K	
Wavelength	1.54184 Å	
Crystal system	triclinic	
Space group	*P*-1	
Unit cell dimensions	*a* = 12.3557(2) Å	α = 100.1054(10)°
	b = 12.99425(18) Å	β = 106.3890(12)°
	c = 18.40115(17) Å	γ = 96.0899(13)°
Volume	2752.22(7) $Å^3$	
Z	2	
Density (calculated)	1.315 Mg/m^3	
Absorption coefficient	3.984 mm^{-1}	
F(000)	1148	
Crystal color, morphology	colourless, needle	
Crystal size	0.185 x 0.071 x 0.066 mm^3	
Theta range for data collection	2.563 to 77.851°	
Index ranges	$-15 \le h \le 15$, $-16 \le k \le 15$, $-22 \le l \le 23$	
Reflections collected	46955	
Independent reflections	11567 [*R*(int) = 0.0640]	
Observed reflections	9751	
Completeness to theta = 74.504°	99.8%	
Absorption correction	Multi-scan	
Max. and min. transmission	1.00000 and 0.58587	
Refinement method	Full-matrix least-squares on F^2	
Data / restraints / parameters	11567 / 975 / 991	
Goodness-of-fit on F^2	1.083	
Final *R* indices [*I*>2sigma(*I*)]	*R*1 = 0.0553, *wR*2 = 0.1494	
R indices (all data)	*R*1 = 0.0639, *wR*2 = 0.1554	
Largest diff. peak and hole	0.513 and -0.618 e.$Å^{-3}$	

Table 2. Atomic coordinates (x 10^4) and equivalent isotropic displacement parameters ($Å^2$x 10^3) for neimca08. U_{eq} is defined as one third of the trace of the orthogonalized U_{ij} tensor.

	x	y	z	U_{eq}
Br1	6691(1)	8687(1)	3611(1)	46(1)
C40	9300(20)	8535(15)	2680(14)	57(1)
Br1'	9241(4)	8693(2)	2658(2)	57(1)
C40'	6718(10)	8904(10)	3360(8)	46(1)
Fe1	7874(1)	7810(1)	3002(1)	38(1)
P1	6587(1)	6586(1)	1821(1)	35(1)
P2	8679(1)	6623(1)	3844(1)	29(1)
O1	8248(1)	5274(1)	2338(1)	31(1)
C1	5744(9)	7255(10)	1117(5)	40(1)
C2	6125(12)	8314(10)	1171(7)	65(3)
C3	5503(13)	8843(8)	640(7)	74(3)
C4	4507(11)	8314(7)	70(6)	57(2)
C5	4140(9)	7265(8)	15(6)	46(2)
C6	4755(11)	6739(10)	547(7)	45(2)
C7	5557(4)	5573(7)	1960(6)	37(3)
C8	5237(12)	4556(10)	1475(7)	34(2)
C9	4459(13)	3780(11)	1566(8)	39(2)
C10	3960(12)	3974(10)	2145(7)	50(3)
C11	4250(7)	4966(9)	2638(7)	55(2)
C12	5044(7)	5743(8)	2547(7)	47(2)
C1'	5601(12)	7256(14)	1190(7)	41(2)
C2'	5801(15)	8344(14)	1278(9)	60(4)
C3'	5073(16)	8873(11)	809(10)	66(4)
C4'	4129(16)	8298(12)	234(10)	63(4)
C5'	3904(17)	7221(13)	143(11)	63(4)
C6'	4628(17)	6697(16)	613(11)	57(4)
C7'	5532(4)	5564(6)	1912(3)	33(2)
C8'	5457(10)	4495(8)	1649(7)	36(2)
C9'	4606(10)	3805(10)	1759(7)	44(2)
C10'	3842(9)	4187(8)	2110(5)	40(2)
C11'	3932(8)	5255(6)	2381(5)	47(2)

C12'	4788(7)	5973(6)	2290(5)	41(2)
C13	7318(2)	5857(2)	1217(2)	37(1)
C14	7179(3)	5879(2)	440(2)	46(1)
C15	7812(3)	5341(3)	44(2)	53(1)
C16	8607(3)	4772(3)	408(2)	50(1)
C17	8772(2)	4720(2)	1185(2)	40(1)
C18	8123(2)	5278(2)	1564(1)	34(1)
C19	9359(2)	5268(2)	2783(1)	32(1)
C20	10062(2)	4692(2)	2467(2)	37(1)
C21	11155(2)	4705(2)	2950(2)	44(1)
C22	11511(2)	5272(3)	3710(2)	46(1)
C23	10795(2)	5843(2)	4008(2)	40(1)
C24	9689(2)	5849(2)	3544(1)	32(1)
C25	9567(2)	4059(2)	1628(2)	41(1)
C26	10513(3)	3807(4)	1270(2)	62(1)
C27	8843(3)	3013(2)	1627(2)	45(1)
C28	7611(5)	5740(6)	4066(3)	36(2)
C29	7525(7)	4635(7)	3893(4)	40(2)
C30	6691(8)	4008(7)	4076(4)	48(2)
C31	5958(6)	4446(7)	4425(5)	53(2)
C32	6037(5)	5526(6)	4594(5)	55(2)
C33	6855(5)	6184(6)	4419(5)	43(2)
C28'	7694(5)	5563(6)	3967(2)	32(2)
C29'	7918(7)	4543(6)	3958(4)	35(2)
C30'	7127(7)	3791(6)	4056(4)	40(2)
C31'	6126(7)	4042(7)	4165(4)	44(2)
C32'	5902(6)	5054(7)	4172(5)	52(2)
C33'	6690(6)	5840(6)	4068(5)	41(2)
C34	9511(2)	7341(2)	4828(1)	33(1)
C35	9625(3)	6888(3)	5468(2)	47(1)
C36	10258(3)	7459(3)	6197(2)	56(1)
C37	10769(3)	8484(3)	6299(2)	55(1)
C38	10676(3)	8941(3)	5667(2)	57(1)
C39	10040(3)	8374(2)	4932(2)	44(1)
O2	7907(3)	7658(2)	7291(2)	78(1)
C41	8519(4)	8705(3)	7507(3)	79(1)

C42	7902(5)	9285(4)	6911(4)	105(2)
C43	7427(5)	8419(4)	6186(4)	94(2)
C44	7223(4)	7459(4)	6499(3)	78(1)
O3	4934(10)	1239(15)	1523(8)	85(2)
C45	4336(9)	972(13)	2046(7)	92(3)
C46	5130(8)	1056(11)	2823(6)	104(3)
C47	6312(7)	1098(9)	2691(7)	88(2)
C48	6125(11)	1624(15)	2006(8)	95(3)
O4	4184(4)	8025(4)	5109(3)	78(1)
C49	4262(8)	7811(8)	4364(6)	92(2)
C50	3739(8)	8654(12)	3990(6)	149(5)
C51	2767(7)	8794(9)	4330(6)	110(3)
C52	3193(5)	8484(5)	5087(4)	66(2)
O5	7977(14)	9655(10)	-420(6)	169(5)
C53	8109(13)	10297(9)	321(7)	109(3)
C54	8871(10)	9858(9)	846(5)	109(3)
C55	8785(12)	8707(11)	462(6)	134(5)
C56	8020(20)	8591(10)	-303(9)	112(4)
O6	1027(8)	7474(7)	747(5)	128(3)
C57	724(9)	7276(13)	1292(6)	122(4)
C58	1773(11)	7274(14)	2015(8)	139(4)
C59	2679(10)	7683(14)	1690(11)	119(5)
C60	1995(9)	8141(9)	1039(7)	115(3)
O3'	5210(14)	1365(19)	1592(9)	80(3)
C45'	4312(12)	740(15)	1762(9)	84(4)
C46'	4837(9)	339(10)	2455(7)	81(3)
C47'	5841(13)	1192(11)	2903(7)	90(3)
C48'	6182(15)	1650(20)	2294(10)	91(3)
O4'	4792(9)	7863(7)	4993(6)	72(2)
C49'	3928(13)	7461(13)	4291(11)	78(4)
C50'	3018(12)	8160(14)	4297(13)	106(4)
C51'	3727(17)	9240(12)	4684(9)	104(5)
C52'	4816(15)	8963(10)	5185(8)	91(5)
O5'	8597(13)	9544(10)	-510(7)	119(4)
C53'	8729(15)	10275(11)	221(8)	102(4)
C54'	7998(14)	9883(10)	584(8)	92(3)

C55'	7661(13)	8691(9)	208(7)	97(3)
C56'	8170(30)	8538(12)	-396(12)	110(4)
O6'	1417(7)	6405(7)	1300(6)	102(3)
C57'	2430(13)	6721(10)	1824(12)	142(7)
C58'	2824(13)	7911(14)	1950(20)	127(6)
C59'	1682(12)	8238(9)	1728(10)	106(4)
C60'	961(13)	7157(11)	1662(12)	112(4)

Table 3.   Bond lengths [Å] and angles [°] for neimca08.

Br(1)-Fe(1)	2.3492(7)	C(7)-C(8)	1.405(11)
C(40)-H(40A)	0.9600	C(7)-C(12)	1.397(10)
C(40)-H(40B)	0.9600	C(8)-H(8)	0.9300
C(40)-H(40C)	0.9600	C(8)-C(9)	1.383(11)
C(40)-Fe(1)	2.18(2)	C(9)-H(9)	0.9300
Br(1')-Fe(1)	2.241(4)	C(9)-C(10)	1.373(12)
C(40')-H(40D)	0.9600	C(10)-H(10)	0.9300
C(40')-H(40E)	0.9600	C(10)-C(11)	1.387(11)
C(40')-H(40F)	0.9600	C(11)-H(11)	0.9300
C(40')-Fe(1)	2.264(11)	C(11)-C(12)	1.397(10)
Fe(1)-P(1)	2.4657(7)	C(12)-H(12)	0.9300
Fe(1)-P(2)	2.4615(7)	C(1')-C(2')	1.382(11)
P(1)-C(1)	1.828(5)	C(1')-C(6')	1.387(12)
P(1)-C(7)	1.833(6)	C(2')-H(2')	0.9300
P(1)-C(1')	1.836(6)	C(2')-C(3')	1.388(12)
P(1)-C(7')	1.823(5)	C(3')-H(3')	0.9300
P(1)-C(13)	1.826(3)	C(3')-C(4')	1.371(12)
P(2)-C(24)	1.828(3)	C(4')-H(4')	0.9300
P(2)-C(28)	1.836(6)	C(4')-C(5')	1.369(12)
P(2)-C(28')	1.827(6)	C(5')-H(5')	0.9300
P(2)-C(34)	1.832(3)	C(5')-C(6')	1.382(12)
O(1)-C(18)	1.390(3)	C(6')-H(6')	0.9300
O(1)-C(19)	1.388(3)	C(7')-C(8')	1.372(9)
C(1)-C(2)	1.383(9)	C(7')-C(12')	1.396(8)
C(1)-C(6)	1.378(9)	C(8')-H(8')	0.9300
C(2)-H(2)	0.9300	C(8')-C(9')	1.392(9)
C(2)-C(3)	1.393(9)	C(9')-H(9')	0.9300
C(3)-H(3)	0.9300	C(9')-C(10')	1.372(10)
C(3)-C(4)	1.388(9)	C(10')-H(10')	0.9300
C(4)-H(4)	0.9300	C(10')-C(11')	1.371(9)
C(4)-C(5)	1.367(9)	C(11')-H(11')	0.9300
C(5)-H(5)	0.9300	C(11')-C(12')	1.401(7)
C(5)-C(6)	1.388(9)	C(12')-H(12')	0.9300
C(6)-H(6)	0.9300	C(13)-C(14)	1.396(4)

C(13)-C(18)	1.390(4)	C(32)-C(33)	1.397(8)
C(14)-H(14)	0.9300	C(33)-H(33)	0.9300
C(14)-C(15)	1.375(5)	C(28')-C(29')	1.381(9)
C(15)-H(15)	0.9300	C(28')-C(33')	1.381(8)
C(15)-C(16)	1.385(5)	C(29')-H(29')	0.9300
C(16)-H(16)	0.9300	C(29')-C(30')	1.377(9)
C(16)-C(17)	1.399(4)	C(30')-H(30')	0.9300
C(17)-C(18)	1.385(4)	C(30')-C(31')	1.371(9)
C(17)-C(25)	1.530(4)	C(31')-H(31')	0.9300
C(19)-C(20)	1.387(4)	C(31')-C(32')	1.371(9)
C(19)-C(24)	1.391(3)	C(32')-H(32')	0.9300
C(20)-C(21)	1.388(4)	C(32')-C(33')	1.411(8)
C(20)-C(25)	1.532(4)	C(33')-H(33')	0.9300
C(21)-H(21)	0.9300	C(34)-C(35)	1.386(4)
C(21)-C(22)	1.384(4)	C(34)-C(39)	1.386(4)
C(22)-H(22)	0.9300	C(35)-H(35)	0.9300
C(22)-C(23)	1.381(4)	C(35)-C(36)	1.377(4)
C(23)-H(23)	0.9300	C(36)-H(36)	0.9300
C(23)-C(24)	1.394(4)	C(36)-C(37)	1.370(5)
C(25)-C(26)	1.532(4)	C(37)-H(37)	0.9300
C(25)-C(27)	1.546(4)	C(37)-C(38)	1.378(5)
C(26)-H(26A)	0.9600	C(38)-H(38)	0.9300
C(26)-H(26B)	0.9600	C(38)-C(39)	1.385(4)
C(26)-H(26C)	0.9600	C(39)-H(39)	0.9300
C(27)-H(27A)	0.9600	O(2)-C(41)	1.415(5)
C(27)-H(27B)	0.9600	O(2)-C(44)	1.426(6)
C(27)-H(27C)	0.9600	C(41)-H(41A)	0.9700
C(28)-C(29)	1.402(9)	C(41)-H(41B)	0.9700
C(28)-C(33)	1.397(9)	C(41)-C(42)	1.514(8)
C(29)-H(29)	0.9300	C(42)-H(42A)	0.9700
C(29)-C(30)	1.398(10)	C(42)-H(42B)	0.9700
C(30)-H(30)	0.9300	C(42)-C(43)	1.511(9)
C(30)-C(31)	1.366(10)	C(43)-H(43A)	0.9700
C(31)-H(31)	0.9300	C(43)-H(43B)	0.9700
C(31)-C(32)	1.370(9)	C(43)-C(44)	1.489(6)
C(32)-H(32)	0.9300	C(44)-H(44A)	0.9700

C(44)-H(44B)	0.9700	C(55)-H(55B)	0.9700
O(3)-C(45)	1.433(11)	C(55)-C(56)	1.430(17)
O(3)-C(48)	1.468(10)	C(56)-H(56A)	0.9700
C(45)-H(45A)	0.9700	C(56)-H(56B)	0.9700
C(45)-H(45B)	0.9700	O(6)-C(57)	1.222(11)
C(45)-C(46)	1.466(11)	O(6)-C(60)	1.313(11)
C(46)-H(46A)	0.9700	C(57)-H(57A)	0.9700
C(46)-H(46B)	0.9700	C(57)-H(57B)	0.9700
C(46)-C(47)	1.545(12)	C(57)-C(58)	1.575(13)
C(47)-H(47A)	0.9700	C(58)-H(58A)	0.9700
C(47)-H(47B)	0.9700	C(58)-H(58B)	0.9700
C(47)-C(48)	1.511(12)	C(58)-C(59)	1.501(14)
C(48)-H(48A)	0.9700	C(59)-H(59A)	0.9700
C(48)-H(48B)	0.9700	C(59)-H(59B)	0.9700
O(4)-C(49)	1.383(9)	C(59)-C(60)	1.513(14)
O(4)-C(52)	1.410(7)	C(60)-H(60A)	0.9700
C(49)-H(49A)	0.9700	C(60)-H(60B)	0.9700
C(49)-H(49B)	0.9700	O(3')-C(45')	1.443(12)
C(49)-C(50)	1.502(11)	O(3')-C(48')	1.454(12)
C(50)-H(50A)	0.9700	C(45')-H(45C)	0.9700
C(50)-H(50B)	0.9700	C(45')-H(45D)	0.9700
C(50)-C(51)	1.515(11)	C(45')-C(46')	1.470(13)
C(51)-H(51A)	0.9700	C(46')-H(46C)	0.9700
C(51)-H(51B)	0.9700	C(46')-H(46D)	0.9700
C(51)-C(52)	1.485(10)	C(46')-C(47')	1.503(14)
C(52)-H(52A)	0.9700	C(47')-H(47C)	0.9700
C(52)-H(52B)	0.9700	C(47')-H(47D)	0.9700
O(5)-C(53)	1.425(12)	C(47')-C(48')	1.492(13)
O(5)-C(56)	1.440(12)	C(48')-H(48C)	0.9700
C(53)-H(53A)	0.9700	C(48')-H(48D)	0.9700
C(53)-H(53B)	0.9700	O(4')-C(49')	1.395(13)
C(53)-C(54)	1.394(13)	O(4')-C(52')	1.406(12)
C(54)-H(54A)	0.9700	C(49')-H(49C)	0.9700
C(54)-H(54B)	0.9700	C(49')-H(49D)	0.9700
C(54)-C(55)	1.516(14)	C(49')-C(50')	1.520(15)
C(55)-H(55A)	0.9700	C(50')-H(50C)	0.9700

C(50')-H(50D)	0.9700	Fe(1)-C(40)-H(40A)	109.5
C(50')-C(51')	1.510(18)	Fe(1)-C(40)-H(40B)	109.5
C(51')-H(51C)	0.9700	Fe(1)-C(40)-H(40C)	109.5
C(51')-H(51D)	0.9700	H(40D)-C(40')-H(40E)	109.5
C(51')-C(52')	1.521(15)	H(40D)-C(40')-H(40F)	109.5
C(52')-H(52C)	0.9700	H(40E)-C(40')-H(40F)	109.5
C(52')-H(52D)	0.9700	Fe(1)-C(40')-H(40D)	109.5
O(5')-C(53')	1.460(11)	Fe(1)-C(40')-H(40E)	109.5
O(5')-C(56')	1.431(14)	Fe(1)-C(40')-H(40F)	109.5
C(53')-H(53C)	0.9700	Br(1)-Fe(1)-P(1)	106.26(3)
C(53')-H(53D)	0.9700	Br(1)-Fe(1)-P(2)	105.21(3)
C(53')-C(54')	1.374(13)	C(40)-Fe(1)-Br(1)	127.1(6)
C(54')-H(54C)	0.9700	C(40)-Fe(1)-P(1)	106.7(6)
C(54')-H(54D)	0.9700	C(40)-Fe(1)-P(2)	105.7(6)
C(54')-C(55')	1.542(15)	Br(1')-Fe(1)-C(40')	111.0(3)
C(55')-H(55C)	0.9700	Br(1')-Fe(1)-P(1)	107.29(11)
C(55')-H(55D)	0.9700	Br(1')-Fe(1)-P(2)	110.83(10)
C(55')-C(56')	1.416(17)	C(40')-Fe(1)-P(1)	103.2(3)
C(56')-H(56C)	0.9700	C(40')-Fe(1)-P(2)	119.5(3)
C(56')-H(56D)	0.9700	P(2)-Fe(1)-P(1)	103.74(3)
O(6')-C(57')	1.318(12)	C(1)-P(1)-Fe(1)	113.7(4)
O(6')-C(60')	1.347(13)	C(1)-P(1)-C(7)	105.7(4)
C(57')-H(57C)	0.9700	C(7)-P(1)-Fe(1)	116.7(3)
C(57')-H(57D)	0.9700	C(1')-P(1)-Fe(1)	112.9(6)
C(57')-C(58')	1.528(15)	C(7')-P(1)-Fe(1)	119.24(18)
C(58')-H(58C)	0.9700	C(7')-P(1)-C(1')	98.2(6)
C(58')-H(58D)	0.9700	C(7')-P(1)-C(13)	103.7(3)
C(58')-C(59')	1.484(15)	C(13)-P(1)-Fe(1)	114.45(9)
C(59')-H(59C)	0.9700	C(13)-P(1)-C(1)	99.5(2)
C(59')-H(59D)	0.9700	C(13)-P(1)-C(7)	105.0(4)
C(59')-C(60')	1.546(13)	C(13)-P(1)-C(1')	106.4(3)
C(60')-H(60C)	0.9700	C(24)-P(2)-Fe(1)	116.10(8)
C(60')-H(60D)	0.9700	C(24)-P(2)-C(28)	109.1(3)
H(40A)-C(40)-H(40B)	109.5	C(24)-P(2)-C(34)	102.70(11)
H(40A)-C(40)-H(40C)	109.5	C(28)-P(2)-Fe(1)	114.57(17)
H(40B)-C(40)-H(40C)	109.5	C(28')-P(2)-Fe(1)	118.1(2)

C(28')-P(2)-C(24)	99.8(3)	C(10)-C(11)-C(12)	120.1(10)
C(28')-P(2)-C(34)	105.57(13)	C(12)-C(11)-H(11)	119.9
C(34)-P(2)-Fe(1)	112.68(9)	C(7)-C(12)-C(11)	122.5(8)
C(34)-P(2)-C(28)	99.91(18)	C(7)-C(12)-H(12)	118.8
C(19)-O(1)-C(18)	114.41(19)	C(11)-C(12)-H(12)	118.8
C(2)-C(1)-P(1)	117.9(8)	C(2')-C(1')-P(1)	120.8(12)
C(6)-C(1)-P(1)	122.2(9)	C(2')-C(1')-C(6')	117.5(10)
C(6)-C(1)-C(2)	119.9(7)	C(6')-C(1')-P(1)	121.7(12)
C(1)-C(2)-H(2)	120.4	C(1')-C(2')-H(2')	118.9
C(1)-C(2)-C(3)	119.2(8)	C(1')-C(2')-C(3')	122.2(11)
C(3)-C(2)-H(2)	120.4	C(3')-C(2')-H(2')	118.9
C(2)-C(3)-H(3)	119.9	C(2')-C(3')-H(3')	120.5
C(4)-C(3)-C(2)	120.2(8)	C(4')-C(3')-C(2')	119.0(12)
C(4)-C(3)-H(3)	119.9	C(4')-C(3')-H(3')	120.5
C(3)-C(4)-H(4)	119.8	C(3')-C(4')-H(4')	120.1
C(5)-C(4)-C(3)	120.3(7)	C(5')-C(4')-C(3')	119.8(12)
C(5)-C(4)-H(4)	119.8	C(5')-C(4')-H(4')	120.1
C(4)-C(5)-H(5)	120.3	C(4')-C(5')-H(5')	119.5
C(4)-C(5)-C(6)	119.5(9)	C(4')-C(5')-C(6')	121.0(13)
C(6)-C(5)-H(5)	120.3	C(6')-C(5')-H(5')	119.5
C(1)-C(6)-C(5)	120.9(9)	C(1')-C(6')-H(6')	119.8
C(1)-C(6)-H(6)	119.6	C(5')-C(6')-C(1')	120.5(13)
C(5)-C(6)-H(6)	119.6	C(5')-C(6')-H(6')	119.8
C(8)-C(7)-P(1)	121.5(9)	C(8')-C(7')-P(1)	125.0(7)
C(12)-C(7)-P(1)	123.3(8)	C(8')-C(7')-C(12')	121.9(6)
C(12)-C(7)-C(8)	115.3(8)	C(12')-C(7')-P(1)	113.1(5)
C(7)-C(8)-H(8)	118.7	C(7')-C(8')-H(8')	120.7
C(9)-C(8)-C(7)	122.5(11)	C(7')-C(8')-C(9')	118.7(9)
C(9)-C(8)-H(8)	118.7	C(9')-C(8')-H(8')	120.7
C(8)-C(9)-H(9)	119.6	C(8')-C(9')-H(9')	119.7
C(10)-C(9)-C(8)	120.9(12)	C(10')-C(9')-C(8')	120.7(10)
C(10)-C(9)-H(9)	119.6	C(10')-C(9')-H(9')	119.7
C(9)-C(10)-H(10)	120.7	C(9')-C(10')-H(10')	119.8
C(9)-C(10)-C(11)	118.7(12)	C(11')-C(10')-C(9')	120.4(9)
C(11)-C(10)-H(10)	120.7	C(11')-C(10')-H(10')	119.8
C(10)-C(11)-H(11)	119.9	C(10')-C(11')-H(11')	119.7

C(10')-C(11')-C(12')	120.6(7)	C(22)-C(23)-C(24)	120.0(3)
C(12')-C(11')-H(11')	119.7	C(24)-C(23)-H(23)	120.0
C(7')-C(12')-C(11')	117.8(6)	C(19)-C(24)-P(2)	117.89(18)
C(7')-C(12')-H(12')	121.1	C(19)-C(24)-C(23)	117.3(2)
C(11')-C(12')-H(12')	121.1	C(23)-C(24)-P(2)	124.7(2)
C(14)-C(13)-P(1)	125.1(2)	C(17)-C(25)-C(20)	106.6(2)
C(18)-C(13)-P(1)	117.77(19)	C(17)-C(25)-C(26)	112.4(3)
C(18)-C(13)-C(14)	117.0(3)	C(17)-C(25)-C(27)	108.4(2)
C(13)-C(14)-H(14)	119.7	C(20)-C(25)-C(27)	108.3(2)
C(15)-C(14)-C(13)	120.7(3)	C(26)-C(25)-C(20)	111.6(2)
C(15)-C(14)-H(14)	119.7	C(26)-C(25)-C(27)	109.4(3)
C(14)-C(15)-H(15)	119.6	C(25)-C(26)-H(26A)	109.5
C(14)-C(15)-C(16)	120.8(3)	C(25)-C(26)-H(26B)	109.5
C(16)-C(15)-H(15)	119.6	C(25)-C(26)-H(26C)	109.5
C(15)-C(16)-H(16)	119.7	H(26A)-C(26)-H(26B)	109.5
C(15)-C(16)-C(17)	120.7(3)	H(26A)-C(26)-H(26C)	109.5
C(17)-C(16)-H(16)	119.7	H(26B)-C(26)-H(26C)	109.5
C(16)-C(17)-C(25)	125.1(3)	C(25)-C(27)-H(27A)	109.5
C(18)-C(17)-C(16)	116.7(3)	C(25)-C(27)-H(27B)	109.5
C(18)-C(17)-C(25)	118.1(2)	C(25)-C(27)-H(27C)	109.5
O(1)-C(18)-C(13)	115.8(2)	H(27A)-C(27)-H(27B)	109.5
C(17)-C(18)-O(1)	120.1(2)	H(27A)-C(27)-H(27C)	109.5
C(17)-C(18)-C(13)	124.1(2)	H(27B)-C(27)-H(27C)	109.5
O(1)-C(19)-C(24)	116.1(2)	C(29)-C(28)-P(2)	122.3(6)
C(20)-C(19)-O(1)	120.0(2)	C(33)-C(28)-P(2)	119.0(6)
C(20)-C(19)-C(24)	123.9(2)	C(33)-C(28)-C(29)	118.7(6)
C(19)-C(20)-C(21)	116.9(2)	C(28)-C(29)-H(29)	120.3
C(19)-C(20)-C(25)	118.1(2)	C(30)-C(29)-C(28)	119.4(7)
C(21)-C(20)-C(25)	125.0(2)	C(30)-C(29)-H(29)	120.3
C(20)-C(21)-H(21)	119.6	C(29)-C(30)-H(30)	119.2
C(22)-C(21)-C(20)	120.7(3)	C(31)-C(30)-C(29)	121.7(7)
C(22)-C(21)-H(21)	119.6	C(31)-C(30)-H(30)	119.2
C(21)-C(22)-H(22)	119.4	C(30)-C(31)-H(31)	120.5
C(23)-C(22)-C(21)	121.1(3)	C(30)-C(31)-C(32)	119.1(6)
C(23)-C(22)-H(22)	119.4	C(32)-C(31)-H(31)	120.5
C(22)-C(23)-H(23)	120.0	C(31)-C(32)-H(32)	119.4

C(31)-C(32)-C(33)	121.3(7)	C(37)-C(38)-C(39)	120.0(3)
C(33)-C(32)-H(32)	119.4	C(39)-C(38)-H(38)	120.0
C(28)-C(33)-H(33)	120.1	C(34)-C(39)-H(39)	119.9
C(32)-C(33)-C(28)	119.8(6)	C(38)-C(39)-C(34)	120.1(3)
C(32)-C(33)-H(33)	120.1	C(38)-C(39)-H(39)	119.9
C(29')-C(28')-P(2)	123.2(6)	C(41)-O(2)-C(44)	109.0(4)
C(29')-C(28')-C(33')	121.1(6)	O(2)-C(41)-H(41A)	110.5
C(33')-C(28')-P(2)	115.7(6)	O(2)-C(41)-H(41B)	110.5
C(28')-C(29')-H(29')	120.3	O(2)-C(41)-C(42)	106.0(4)
C(30')-C(29')-C(28')	119.4(7)	H(41A)-C(41)-H(41B)	108.7
C(30')-C(29')-H(29')	120.3	C(42)-C(41)-H(41A)	110.5
C(29')-C(30')-H(30')	119.5	C(42)-C(41)-H(41B)	110.5
C(31')-C(30')-C(29')	121.1(7)	C(41)-C(42)-H(42A)	111.2
C(31')-C(30')-H(30')	119.5	C(41)-C(42)-H(42B)	111.2
C(30')-C(31')-H(31')	120.2	H(42A)-C(42)-H(42B)	109.1
C(30')-C(31')-C(32')	119.6(7)	C(43)-C(42)-C(41)	102.9(4)
C(32')-C(31')-H(31')	120.2	C(43)-C(42)-H(42A)	111.2
C(31')-C(32')-H(32')	119.6	C(43)-C(42)-H(42B)	111.2
C(31')-C(32')-C(33')	120.7(6)	C(42)-C(43)-H(43A)	111.2
C(33')-C(32')-H(32')	119.6	C(42)-C(43)-H(43B)	111.2
C(28')-C(33')-C(32')	118.1(6)	H(43A)-C(43)-H(43B)	109.1
C(28')-C(33')-H(33')	120.9	C(44)-C(43)-C(42)	102.8(5)
C(32')-C(33')-H(33')	120.9	C(44)-C(43)-H(43A)	111.2
C(35)-C(34)-P(2)	122.4(2)	C(44)-C(43)-H(43B)	111.2
C(39)-C(34)-P(2)	118.5(2)	O(2)-C(44)-C(43)	108.7(4)
C(39)-C(34)-C(35)	119.1(3)	O(2)-C(44)-H(44A)	109.9
C(34)-C(35)-H(35)	119.8	O(2)-C(44)-H(44B)	109.9
C(36)-C(35)-C(34)	120.3(3)	C(43)-C(44)-H(44A)	109.9
C(36)-C(35)-H(35)	119.8	C(43)-C(44)-H(44B)	109.9
C(35)-C(36)-H(36)	119.8	H(44A)-C(44)-H(44B)	108.3
C(37)-C(36)-C(35)	120.4(3)	C(45)-O(3)-C(48)	105.4(10)
C(37)-C(36)-H(36)	119.8	O(3)-C(45)-H(45A)	109.4
C(36)-C(37)-H(37)	120.0	O(3)-C(45)-H(45B)	109.4
C(36)-C(37)-C(38)	120.0(3)	O(3)-C(45)-C(46)	111.2(9)
C(38)-C(37)-H(37)	120.0	H(45A)-C(45)-H(45B)	108.0
C(37)-C(38)-H(38)	120.0	C(46)-C(45)-H(45A)	109.4

C(46)-C(45)-H(45B)	109.4	C(52)-C(51)-H(51A)	111.3
C(45)-C(46)-H(46A)	111.0	C(52)-C(51)-H(51B)	111.3
C(45)-C(46)-H(46B)	111.0	O(4)-C(52)-C(51)	109.2(5)
C(45)-C(46)-C(47)	103.7(8)	O(4)-C(52)-H(52A)	109.8
H(46A)-C(46)-H(46B)	109.0	O(4)-C(52)-H(52B)	109.8
C(47)-C(46)-H(46A)	111.0	C(51)-C(52)-H(52A)	109.8
C(47)-C(46)-H(46B)	111.0	C(51)-C(52)-H(52B)	109.8
C(46)-C(47)-H(47A)	111.6	H(52A)-C(52)-H(52B)	108.3
C(46)-C(47)-H(47B)	111.6	C(53)-O(5)-C(56)	105.8(10)
H(47A)-C(47)-H(47B)	109.4	O(5)-C(53)-H(53A)	110.8
C(48)-C(47)-C(46)	101.0(9)	O(5)-C(53)-H(53B)	110.8
C(48)-C(47)-H(47A)	111.6	H(53A)-C(53)-H(53B)	108.8
C(48)-C(47)-H(47B)	111.6	C(54)-C(53)-O(5)	104.9(10)
O(3)-C(48)-C(47)	106.4(8)	C(54)-C(53)-H(53A)	110.8
O(3)-C(48)-H(48A)	110.5	C(54)-C(53)-H(53B)	110.8
O(3)-C(48)-H(48B)	110.5	C(53)-C(54)-H(54A)	110.4
C(47)-C(48)-H(48A)	110.5	C(53)-C(54)-H(54B)	110.4
C(47)-C(48)-H(48B)	110.5	C(53)-C(54)-C(55)	106.8(8)
H(48A)-C(48)-H(48B)	108.6	H(54A)-C(54)-H(54B)	108.6
C(49)-O(4)-C(52)	107.9(5)	C(55)-C(54)-H(54A)	110.4
O(4)-C(49)-H(49A)	110.7	C(55)-C(54)-H(54B)	110.4
O(4)-C(49)-H(49B)	110.7	C(54)-C(55)-H(55A)	110.8
O(4)-C(49)-C(50)	105.2(7)	C(54)-C(55)-H(55B)	110.8
H(49A)-C(49)-H(49B)	108.8	H(55A)-C(55)-H(55B)	108.9
C(50)-C(49)-H(49A)	110.7	C(56)-C(55)-C(54)	104.7(8)
C(50)-C(49)-H(49B)	110.7	C(56)-C(55)-H(55A)	110.8
C(49)-C(50)-H(50A)	111.3	C(56)-C(55)-H(55B)	110.8
C(49)-C(50)-H(50B)	111.3	O(5)-C(56)-H(56A)	110.7
C(49)-C(50)-C(51)	102.2(7)	O(5)-C(56)-H(56B)	110.7
H(50A)-C(50)-H(50B)	109.2	C(55)-C(56)-O(5)	105.2(10)
C(51)-C(50)-H(50A)	111.3	C(55)-C(56)-H(56A)	110.7
C(51)-C(50)-H(50B)	111.3	C(55)-C(56)-H(56B)	110.7
C(50)-C(51)-H(51A)	111.3	H(56A)-C(56)-H(56B)	108.8
C(50)-C(51)-H(51B)	111.3	C(57)-O(6)-C(60)	107.2(9)
H(51A)-C(51)-H(51B)	109.2	O(6)-C(57)-H(57A)	109.2
C(52)-C(51)-C(50)	102.4(5)	O(6)-C(57)-H(57B)	109.2

O(6)-C(57)-C(58)	111.9(9)	C(46')-C(47')-H(47D)	110.9
H(57A)-C(57)-H(57B)	107.9	H(47C)-C(47')-H(47D)	108.9
C(58)-C(57)-H(57A)	109.2	C(48')-C(47')-C(46')	104.3(10)
C(58)-C(57)-H(57B)	109.2	C(48')-C(47')-H(47C)	110.9
C(57)-C(58)-H(58A)	112.5	C(48')-C(47')-H(47D)	110.9
C(57)-C(58)-H(58B)	112.5	O(3')-C(48')-C(47')	107.5(10)
H(58A)-C(58)-H(58B)	110.0	O(3')-C(48')-H(48C)	110.2
C(59)-C(58)-C(57)	96.2(8)	O(3')-C(48')-H(48D)	110.2
C(59)-C(58)-H(58A)	112.5	C(47')-C(48')-H(48C)	110.2
C(59)-C(58)-H(58B)	112.5	C(47')-C(48')-H(48D)	110.2
C(58)-C(59)-H(59A)	111.4	H(48C)-C(48')-H(48D)	108.5
C(58)-C(59)-H(59B)	111.4	C(49')-O(4')-C(52')	107.7(10)
C(58)-C(59)-C(60)	102.0(9)	O(4')-C(49')-H(49C)	110.7
H(59A)-C(59)-H(59B)	109.2	O(4')-C(49')-H(49D)	110.7
C(60)-C(59)-H(59A)	111.4	O(4')-C(49')-C(50')	105.1(12)
C(60)-C(59)-H(59B)	111.4	H(49C)-C(49')-H(49D)	108.8
O(6)-C(60)-C(59)	103.9(9)	C(50')-C(49')-H(49C)	110.7
O(6)-C(60)-H(60A)	111.0	C(50')-C(49')-H(49D)	110.7
O(6)-C(60)-H(60B)	111.0	C(49')-C(50')-H(50C)	111.3
C(59)-C(60)-H(60A)	111.0	C(49')-C(50')-H(50D)	111.3
C(59)-C(60)-H(60B)	111.0	H(50C)-C(50')-H(50D)	109.2
H(60A)-C(60)-H(60B)	109.0	C(51')-C(50')-C(49')	102.3(11)
C(45')-O(3')-C(48')	107.1(11)	C(51')-C(50')-H(50C)	111.3
O(3')-C(45')-H(45C)	110.1	C(51')-C(50')-H(50D)	111.3
O(3')-C(45')-H(45D)	110.1	C(50')-C(51')-H(51C)	111.4
O(3')-C(45')-C(46')	107.9(11)	C(50')-C(51')-H(51D)	111.4
H(45C)-C(45')-H(45D)	108.4	C(50')-C(51')-C(52')	102.1(10)
C(46')-C(45')-H(45C)	110.1	H(51C)-C(51')-H(51D)	109.2
C(46')-C(45')-H(45D)	110.1	C(52')-C(51')-H(51C)	111.4
C(45')-C(46')-H(46C)	111.2	C(52')-C(51')-H(51D)	111.4
C(45')-C(46')-H(46D)	111.2	O(4')-C(52')-C(51')	109.2(10)
C(45')-C(46')-C(47')	103.1(9)	O(4')-C(52')-H(52C)	109.8
H(46C)-C(46')-H(46D)	109.1	O(4')-C(52')-H(52D)	109.8
C(47')-C(46')-H(46C)	111.2	C(51')-C(52')-H(52C)	109.8
C(47')-C(46')-H(46D)	111.2	C(51')-C(52')-H(52D)	109.8
C(46')-C(47')-H(47C)	110.9	H(52C)-C(52')-H(52D)	108.3

C(56')-O(5')-C(53')	104.0(10)	C(57')-O(6')-C(60')	92.0(12)
O(5')-C(53')-H(53C)	109.6	O(6')-C(57')-H(57C)	109.3
O(5')-C(53')-H(53D)	109.6	O(6')-C(57')-H(57D)	109.3
H(53C)-C(53')-H(53D)	108.2	O(6')-C(57')-C(58')	111.7(13)
C(54')-C(53')-O(5')	110.1(10)	H(57C)-C(57')-H(57D)	107.9
C(54')-C(53')-H(53C)	109.6	C(58')-C(57')-H(57C)	109.3
C(54')-C(53')-H(53D)	109.6	C(58')-C(57')-H(57D)	109.3
C(53')-C(54')-H(54C)	110.7	C(57')-C(58')-H(58C)	112.1
C(53')-C(54')-H(54D)	110.7	C(57')-C(58')-H(58D)	112.1
C(53')-C(54')-C(55')	105.1(9)	H(58C)-C(58')-H(58D)	109.8
H(54C)-C(54')-H(54D)	108.8	C(59')-C(58')-C(57')	98.3(10)
C(55')-C(54')-H(54C)	110.7	C(59')-C(58')-H(58C)	112.1
C(55')-C(54')-H(54D)	110.7	C(59')-C(58')-H(58D)	112.1
C(54')-C(55')-H(55C)	110.7	C(58')-C(59')-H(59C)	112.3
C(54')-C(55')-H(55D)	110.7	C(58')-C(59')-H(59D)	112.3
H(55C)-C(55')-H(55D)	108.8	C(58')-C(59')-C(60')	97.5(10)
C(56')-C(55')-C(54')	105.4(10)	H(59C)-C(59')-H(59D)	109.9
C(56')-C(55')-H(55C)	110.7	C(60')-C(59')-H(59C)	112.3
C(56')-C(55')-H(55D)	110.7	C(60')-C(59')-H(59D)	112.3
O(5')-C(56')-H(56C)	109.7	O(6')-C(60')-C(59')	107.1(10)
O(5')-C(56')-H(56D)	109.7	O(6')-C(60')-H(60C)	110.3
C(55')-C(56')-O(5')	109.6(11)	O(6')-C(60')-H(60D)	110.3
C(55')-C(56')-H(56C)	109.7	C(59')-C(60')-H(60C)	110.3
C(55')-C(56')-H(56D)	109.7	C(59')-C(60')-H(60D)	110.3
H(56C)-C(56')-H(56D)	108.2	H(60C)-C(60')-H(60D)	108.6

Table 4. Anisotropic displacement parameters ($Å^2$x 10^3) for neimca08. The anisotropic displacement factor exponent takes the form: $-2\pi^2[h^2 a^{*2}U_{11} + ... + 2 h k a^* b^* U_{12}]$

	U_{11}	U_{22}	U_{33}	U_{23}	U_{13}	U_{12}
Br1	38(1)	32(1)	62(1)	0(1)	12(1)	7(1)
C40	74(1)	45(1)	43(1)	-3(1)	21(1)	-17(1)
Br1'	74(1)	45(1)	43(1)	-3(1)	21(1)	-17(1)
C40'	38(1)	32(1)	62(1)	0(1)	12(1)	7(1)
Fe1	40(1)	29(1)	34(1)	4(1)	-1(1)	3(1)
P1	34(1)	27(1)	34(1)	5(1)	-2(1)	4(1)
P2	26(1)	30(1)	28(1)	4(1)	4(1)	3(1)
O1	28(1)	37(1)	24(1)	5(1)	3(1)	7(1)
C1	41(3)	37(3)	40(3)	7(3)	6(2)	7(2)
C2	65(6)	39(3)	66(4)	19(3)	-20(4)	-2(4)
C3	79(7)	46(3)	80(5)	31(3)	-10(5)	4(4)
C4	63(5)	54(3)	53(4)	28(3)	1(3)	19(3)
C5	45(4)	53(3)	38(3)	13(2)	4(3)	18(3)
C6	45(3)	37(3)	46(3)	10(3)	0(3)	8(3)
C7	28(5)	33(4)	37(4)	1(4)	-8(4)	8(4)
C8	38(6)	31(3)	29(4)	14(3)	-3(4)	9(3)
C9	40(5)	35(3)	32(5)	11(3)	-3(4)	1(3)
C10	37(5)	46(5)	59(4)	6(4)	8(4)	-2(4)
C11	28(4)	57(5)	68(6)	-4(4)	9(4)	0(3)
C12	34(4)	37(4)	60(5)	-6(3)	7(4)	7(3)
C1'	50(5)	25(4)	35(4)	5(4)	-6(3)	11(4)
C2'	71(8)	31(4)	52(5)	5(4)	-18(5)	6(5)
C3'	75(8)	37(4)	69(7)	15(4)	-7(6)	12(5)
C4'	61(7)	51(4)	63(7)	17(5)	-9(5)	17(5)
C5'	57(7)	51(4)	58(7)	10(5)	-17(6)	9(5)
C6'	54(6)	37(5)	56(6)	9(4)	-21(5)	2(5)
C7'	27(4)	33(3)	32(4)	5(3)	3(3)	3(3)
C8'	27(4)	33(3)	48(5)	14(3)	5(4)	9(2)
C9'	37(4)	37(3)	53(6)	13(4)	6(4)	5(3)
C10'	27(3)	42(4)	49(3)	16(3)	10(2)	1(3)
C11'	41(4)	50(3)	54(4)	8(3)	25(3)	1(3)

C12'	39(4)	35(3)	50(4)	7(3)	20(3)	2(2)
C13	40(1)	33(1)	31(1)	8(1)	3(1)	-1(1)
C14	54(2)	44(2)	33(1)	13(1)	2(1)	-2(1)
C15	64(2)	63(2)	31(1)	15(1)	13(1)	0(2)
C16	53(2)	62(2)	33(1)	8(1)	16(1)	3(2)
C17	38(1)	45(2)	31(1)	4(1)	10(1)	1(1)
C18	34(1)	37(1)	26(1)	6(1)	5(1)	-1(1)
C19	27(1)	36(1)	29(1)	6(1)	3(1)	6(1)
C20	32(1)	41(1)	35(1)	4(1)	8(1)	8(1)
C21	34(1)	51(2)	43(2)	4(1)	10(1)	14(1)
C22	32(1)	55(2)	43(2)	4(1)	1(1)	14(1)
C23	34(1)	43(2)	34(1)	2(1)	1(1)	10(1)
C24	31(1)	33(1)	29(1)	4(1)	3(1)	7(1)
C25	37(1)	51(2)	32(1)	-1(1)	10(1)	10(1)
C26	52(2)	91(3)	43(2)	-3(2)	22(2)	21(2)
C27	54(2)	41(2)	35(1)	-1(1)	7(1)	14(1)
C28	31(3)	34(3)	38(3)	6(2)	3(2)	3(2)
C29	35(4)	37(3)	37(3)	7(2)	-3(3)	2(3)
C30	44(5)	39(3)	53(3)	14(3)	4(4)	-5(3)
C31	40(3)	51(4)	63(4)	17(3)	11(3)	-10(3)
C32	32(3)	58(4)	71(5)	12(3)	18(3)	-7(3)
C33	32(3)	41(3)	54(4)	5(3)	15(3)	1(2)
C28'	28(3)	37(3)	28(3)	7(2)	4(2)	5(2)
C29'	30(4)	35(3)	32(3)	8(2)	0(3)	2(3)
C30'	37(4)	34(3)	40(3)	9(2)	1(3)	-2(3)
C31'	37(4)	46(4)	47(4)	17(3)	12(3)	-5(3)
C32'	34(3)	55(4)	69(4)	19(3)	18(3)	4(3)
C33'	33(3)	42(4)	50(4)	16(3)	13(3)	6(3)
C34	26(1)	36(1)	31(1)	0(1)	5(1)	6(1)
C35	54(2)	49(2)	33(1)	6(1)	8(1)	7(1)
C36	65(2)	65(2)	32(1)	4(1)	6(1)	18(2)
C37	50(2)	57(2)	41(2)	-10(1)	-3(1)	18(2)
C38	52(2)	44(2)	55(2)	-7(1)	-2(2)	-3(1)
C39	41(2)	38(1)	40(1)	1(1)	2(1)	-1(1)
O2	82(2)	51(2)	96(2)	15(2)	24(2)	1(1)
C41	67(3)	54(2)	119(4)	3(2)	43(3)	8(2)

C42	83(3)	55(3)	190(7)	40(3)	52(4)	13(2)
C43	87(3)	76(3)	134(5)	43(3)	39(3)	28(3)
C44	64(2)	59(2)	108(4)	23(2)	21(2)	12(2)
O3	46(5)	60(5)	135(5)	35(4)	1(3)	-3(4)
C45	56(4)	87(7)	124(7)	30(7)	9(4)	18(4)
C46	54(4)	119(6)	115(5)	6(5)	7(4)	0(5)
C47	46(4)	97(5)	113(6)	21(5)	15(4)	1(4)
C48	51(4)	82(5)	135(7)	26(6)	7(5)	-10(4)
O4	69(3)	95(3)	94(3)	44(3)	38(2)	37(2)
C49	67(5)	118(6)	97(4)	15(4)	37(4)	26(4)
C50	101(6)	287(13)	127(7)	128(8)	66(5)	98(8)
C51	82(5)	157(8)	139(6)	94(6)	57(4)	59(5)
C52	65(3)	58(3)	86(4)	20(3)	34(3)	21(3)
O5	225(12)	126(6)	111(5)	20(5)	-15(6)	21(7)
C53	117(7)	83(5)	108(6)	-1(5)	20(6)	5(6)
C54	95(6)	130(6)	78(5)	17(5)	9(5)	-25(6)
C55	161(11)	185(10)	104(7)	81(6)	53(6)	114(9)
C56	150(8)	81(5)	86(5)	3(4)	21(5)	6(5)
O6	144(6)	117(6)	109(5)	-29(4)	60(5)	-3(5)
C57	93(6)	152(8)	80(6)	-33(6)	20(5)	-38(6)
C58	117(8)	146(9)	141(8)	49(7)	25(6)	-29(7)
C59	93(6)	95(8)	186(13)	37(9)	63(6)	20(5)
C60	124(8)	110(8)	129(9)	20(6)	66(6)	25(5)
O3'	62(6)	60(5)	110(5)	28(4)	13(4)	-1(5)
C45'	64(5)	73(7)	106(7)	35(6)	11(5)	-6(4)
C46'	64(5)	87(7)	88(6)	26(5)	12(5)	11(5)
C47'	65(6)	87(6)	104(6)	14(5)	14(5)	-2(6)
C48'	57(5)	90(6)	115(8)	31(7)	8(5)	-2(4)
O4'	66(5)	74(4)	82(5)	25(4)	29(4)	14(4)
C49'	46(7)	79(8)	100(7)	-3(6)	22(5)	10(6)
C50'	76(7)	124(10)	144(9)	49(8)	51(7)	47(6)
C51'	165(15)	91(9)	62(8)	8(7)	38(9)	60(9)
C52'	136(12)	65(7)	63(8)	12(6)	17(7)	13(8)
O5'	176(11)	104(5)	87(5)	-16(4)	80(6)	12(6)
C53'	118(9)	91(6)	87(6)	-29(5)	63(6)	-26(6)
C54'	105(6)	86(6)	83(6)	0(5)	48(5)	-11(6)

C55'	136(9)	69(5)	74(6)	12(5)	17(6)	9(6)
C56'	158(9)	77(5)	77(6)	-6(5)	23(6)	22(6)
O6'	89(5)	76(5)	144(8)	5(5)	53(5)	12(4)
C57'	95(8)	75(7)	214(19)	-5(10)	12(9)	-9(6)
C58'	98(6)	75(7)	195(15)	-6(9)	47(8)	8(6)
C59'	122(9)	72(6)	119(11)	11(7)	33(9)	17(6)
C60'	84(6)	118(6)	124(9)	16(7)	30(7)	2(6)

Table 5. Hydrogen coordinates (x 10^4) and isotropic displacement parameters (Å^2 x 10^3) for neimca08.

	x	y	z	U(eq)
H40A	9009	8727	2189	86
H40B	9813	8040	2641	86
H40C	9696	9158	3068	86
H40D	6790	8946	3898	69
H40E	5939	8633	3051	69
H40F	6935	9598	3281	69
H2	6788	8668	1557	78
H3	5756	9553	669	88
H4	4088	8675	-277	69
H5	3483	6907	-375	55
H6	4497	6030	517	55
H8	5559	4399	1077	41
H9	4273	3117	1232	46
H10	3438	3450	2204	60
H11	3914	5115	3029	66
H12	5240	6398	2891	56
H2'	6446	8735	1664	72
H3'	5222	9607	885	79
H4'	3644	8638	-94	76
H5'	3254	6835	-240	75
H6'	4462	5965	542	69
H8'	5966	4238	1402	43
H9'	4553	3078	1593	52
H10'	3261	3719	2165	47
H11'	3420	5505	2628	56
H12'	4858	6698	2476	49
H14	6653	6262	188	56
H15	7705	5359	-474	64
H16	9036	4420	134	60
H21	11654	4328	2761	52

H22	12246	5269	4025	55
H23	11050	6224	4519	48
H26A	10175	3409	746	93
H26B	10993	3399	1568	93
H26C	10963	4456	1269	93
H27A	8271	3169	1873	68
H27B	9329	2590	1906	68
H27C	8477	2630	1102	68
H29	8019	4321	3658	47
H30	6634	3275	3957	58
H31	5413	4017	4547	64
H32	5537	5827	4829	65
H33	6896	6916	4538	52
H29'	8596	4364	3887	42
H30'	7274	3103	4047	48
H31'	5602	3528	4233	52
H32'	5223	5225	4247	62
H33'	6536	6525	4067	49
H35	9273	6195	5405	56
H36	10338	7146	6623	67
H37	11179	8872	6794	66
H38	11041	9631	5734	69
H39	9968	8688	4507	52
H41A	9303	8704	7506	94
H41B	8523	9042	8022	94
H42A	7293	9591	7060	126
H42B	8426	9841	6839	126
H43A	6721	8556	5850	113
H43B	7974	8346	5899	113
H44A	6421	7298	6458	93
H44B	7423	6856	6202	93
H45A	3921	253	1849	110
H45B	3785	1444	2076	110
H46A	4956	447	3033	124
H46B	5099	1695	3175	124
H47A	6913	1518	3139	106

H47B	6496	393	2567	106
H48A	6255	2388	2179	114
H48B	6644	1436	1714	114
H49A	3846	7112	4085	110
H49B	5054	7843	4375	110
H50A	3457	8422	3430	179
H50B	4282	9305	4127	179
H51A	2637	9523	4401	132
H51B	2064	8334	4004	132
H52A	3371	9104	5506	79
H52B	2609	7980	5152	79
H53A	8415	11027	349	131
H53B	7381	10276	426	131
H54A	8672	9906	1323	131
H54B	9645	10230	968	131
H55A	9528	8550	443	161
H55B	8485	8238	741	161
H56A	7264	8239	-343	134
H56B	8298	8181	-684	134
H57A	238	6587	1131	146
H57B	277	7800	1438	146
H58A	1763	7751	2481	167
H58B	1836	6569	2111	167
H59A	3041	7115	1495	143
H59B	3258	8223	2076	143
H60A	1865	8845	1236	139
H60B	2385	8180	653	139
H45C	3929	154	1326	101
H45D	3752	1173	1856	101
H46C	5084	-333	2317	97
H46D	4309	249	2751	97
H47C	5625	1728	3249	108
H47D	6462	898	3206	108
H48C	6387	2413	2460	110
H48D	6837	1363	2201	110
H49C	3621	6729	4254	94

H49D	4218	7500	3856	94
H50C	2546	8149	3775	127
H50D	2533	7941	4594	127
H51C	3352	9663	4997	125
H51D	3880	9620	4305	125
H52C	5477	9334	5098	110
H52D	4876	9180	5728	110
H53C	9512	10372	555	122
H53D	8565	10960	121	122
H54C	7328	10228	504	110
H54D	8376	9991	1137	110
H55C	7951	8269	583	117
H55D	6836	8493	2	117
H56C	7602	8139	-872	132
H56D	8786	8137	-264	132
H57C	2983	6339	1660	170
H57D	2397	6556	2312	170
H58C	3265	8227	2480	153
H58D	3260	8070	1604	153
H59C	1510	8465	1239	128
H59D	1597	8787	2129	128
H60C	986	7045	2173	134
H60D	171	7137	1365	134

Table 6. Torsion angles [°] for neimca08.

Fe1-P1-C1-C2	19.8(4)	P2-C34-C39-C38	-180.0(3)
Fe1-P1-C1-C6	-160.0(5)	O1-C19-C20-C21	-178.8(2)
Fe1-P1-C7-C8	-145.3(6)	O1-C19-C20-C25	-1.0(4)
Fe1-P1-C7-C12	34.2(6)	O1-C19-C24-P2	-4.5(3)
Fe1-P1-C1'-C2'	16.6(6)	O1-C19-C24-C23	179.0(2)
Fe1-P1-C1'-C6'	-163.0(7)	C1-P1-C7-C8	87.2(6)
Fe1-P1-C7'-C8'	-121.6(5)	C1-P1-C7-C12	-93.3(6)
Fe1-P1-C7'-C12'	57.7(5)	C1-P1-C13-C14	-0.3(5)
Fe1-P1-C13-C14	-121.9(2)	C1-P1-C13-C18	176.0(5)
Fe1-P1-C13-C18	54.4(2)	C1-C2-C3-C4	0.6(8)
Fe1-P2-C24-C19	-51.2(2)	C2-C1-C6-C5	0.8(8)
Fe1-P2-C24-C23	125.1(2)	C2-C3-C4-C5	-1.2(10)
Fe1-P2-C28-C29	122.4(3)	C3-C4-C5-C6	1.6(10)
Fe1-P2-C28-C33	-57.5(4)	C4-C5-C6-C1	-1.4(10)
Fe1-P2-C28'-C29'	137.2(3)	C6-C1-C2-C3	-0.5(6)
Fe1-P2-C28'-C33'	-42.4(4)	C7-P1-C1-C2	149.0(6)
Fe1-P2-C34-C35	151.7(2)	C7-P1-C1-C6	-30.8(7)
Fe1-P2-C34-C39	-28.1(2)	C7-P1-C13-C14	108.9(3)
P1-C1-C2-C3	179.7(4)	C7-P1-C13-C18	-74.8(3)
P1-C1-C6-C5	-179.4(5)	C7-C8-C9-C10	0.0(9)
P1-C7-C8-C9	-179.8(4)	C8-C7-C12-C11	-1.4(7)
P1-C7-C12-C11	179.1(4)	C8-C9-C10-C11	0.0(12)
P1-C1'-C2'-C3'	180.0(4)	C9-C10-C11-C12	-0.7(11)
P1-C1'-C6'-C5'	-179.6(6)	C10-C11-C12-C7	1.5(10)
P1-C7'-C8'-C9'	179.9(4)	C12-C7-C8-C9	0.6(6)
P1-C7'-C12'-C11'	179.1(4)	C1'-P1-C7'-C8'	116.3(6)
P1-C13-C14-C15	176.8(2)	C1'-P1-C7'-C12'	-64.4(6)
P1-C13-C18-O1	3.0(3)	C1'-P1-C13-C14	3.5(7)
P1-C13-C18-C17	-177.5(2)	C1'-P1-C13-C18	179.8(7)
P2-C28-C29-C30	-179.9(3)	C1'-C2'-C3'-C4'	-0.8(9)
P2-C28-C33-C32	-179.9(4)	C2'-C1'-C6'-C5'	0.8(9)
P2-C28'-C29'-C30'	-180.0(4)	C2'-C3'-C4'-C5'	1.7(12)
P2-C28'-C33'-C32'	-179.5(4)	C3'-C4'-C5'-C6'	-1.4(13)
P2-C34-C35-C36	-179.8(3)	C4'-C5'-C6'-C1'	0.2(13)

C6'-C1'-C2'-C3'	-0.5(6)	C19-O1-C18-C13	-144.6(2)
C7'-P1-C1'-C2'	143.2(6)	C19-O1-C18-C17	35.9(3)
C7'-P1-C1'-C6'	-36.3(7)	C19-C20-C21-C22	-0.1(4)
C7'-P1-C13-C14	106.5(3)	C19-C20-C25-C17	36.3(3)
C7'-P1-C13-C18	-77.2(2)	C19-C20-C25-C26	159.4(3)
C7'-C8'-C9'-C10'	1.2(8)	C19-C20-C25-C27	-80.2(3)
C8'-C7'-C12'-C11'	-1.5(6)	C20-C19-C24-P2	176.5(2)
C8'-C9'-C10'-C11'	-2.2(11)	C20-C19-C24-C23	0.0(4)
C9'-C10'-C11'-C12'	1.3(10)	C20-C21-C22-C23	-0.2(5)
C10'-C11'-C12'-C7'	0.5(8)	C21-C20-C25-C17	-146.2(3)
C12'-C7'-C8'-C9'	0.7(5)	C21-C20-C25-C26	-23.1(4)
C13-P1-C1-C2	-102.3(5)	C21-C20-C25-C27	97.4(3)
C13-P1-C1-C6	77.8(5)	C21-C22-C23-C24	0.5(5)
C13-P1-C7-C8	-17.4(5)	C22-C23-C24-P2	-176.6(2)
C13-P1-C7-C12	162.1(6)	C22-C23-C24-C19	-0.3(4)
C13-P1-C1'-C2'	-109.7(6)	C24-P2-C28-C29	-9.6(3)
C13-P1-C1'-C6'	70.7(7)	C24-P2-C28-C33	170.5(3)
C13-P1-C7'-C8'	7.1(5)	C24-P2-C28'-C29'	10.5(3)
C13-P1-C7'-C12'	-173.6(5)	C24-P2-C28'-C33'	-169.2(4)
C13-C14-C15-C16	-0.5(5)	C24-P2-C34-C35	-82.7(3)
C14-C13-C18-O1	179.6(2)	C24-P2-C34-C39	97.5(2)
C14-C13-C18-C17	-0.9(4)	C24-C19-C20-C21	0.2(4)
C14-C15-C16-C17	0.9(5)	C24-C19-C20-C25	177.9(3)
C15-C16-C17-C18	-1.3(5)	C25-C17-C18-O1	3.4(4)
C15-C16-C17-C25	175.8(3)	C25-C17-C18-C13	-176.0(3)
C16-C17-C18-O1	-179.3(2)	C25-C20-C21-C22	-177.6(3)
C16-C17-C18-C13	1.3(4)	C28-P2-C24-C19	80.1(2)
C16-C17-C25-C20	145.4(3)	C28-P2-C24-C23	-103.7(3)
C16-C17-C25-C26	22.8(4)	C28-P2-C34-C35	29.6(4)
C16-C17-C25-C27	-98.2(3)	C28-P2-C34-C39	-150.2(3)
C18-O1-C19-C20	-37.1(3)	C28-C29-C30-C31	-0.4(8)
C18-O1-C19-C24	143.8(2)	C29-C28-C33-C32	0.2(6)
C18-C13-C14-C15	0.5(4)	C29-C30-C31-C32	0.6(9)
C18-C17-C25-C20	-37.5(3)	C30-C31-C32-C33	-0.4(9)
C18-C17-C25-C26	-160.1(3)	C31-C32-C33-C28	0.0(9)
C18-C17-C25-C27	78.9(3)	C33-C28-C29-C30	0.0(5)

C28'-P2-C24-C19	76.9(2)	C52-O4-C49-C50	31.3(10)
C28'-P2-C24-C23	-106.9(3)	O5-C53-C54-C55	-24.4(17)
C28'-P2-C34-C35	21.4(4)	C53-O5-C56-C55	-33(2)
C28'-P2-C34-C39	-158.4(3)	C53-C54-C55-C56	4.4(18)
C28'-C29'-C30'-C31'	-0.3(8)	C54-C55-C56-O5	17.2(19)
C29'-C28'-C33'-C32'	0.9(7)	C56-O5-C53-C54	35.9(19)
C29'-C30'-C31'-C32'	0.4(10)	O6-C57-C58-C59	7.5(18)
C30'-C31'-C32'-C33'	0.1(10)	C57-O6-C60-C59	44.9(15)
C31'-C32'-C33'-C28'	-0.8(9)	C57-C58-C59-C60	17.4(16)
C33'-C28'-C29'-C30'	-0.4(5)	C58-C59-C60-O6	-37.9(15)
C34-P2-C24-C19	-174.6(2)	C60-O6-C57-C58	-33.6(17)
C34-P2-C24-C23	1.7(3)	O3'-C45'-C46'-C47'	31(2)
C34-P2-C28-C29	-116.9(3)	C45'-O3'-C48'-C47'	-1(3)
C34-P2-C28-C33	63.2(4)	C45'-C46'-C47'-C48'	-30.3(19)
C34-P2-C28'-C29'	-95.8(3)	C46'-C47'-C48'-O3'	20(2)
C34-P2-C28'-C33'	84.6(4)	C48'-O3'-C45'-C46'	-19(3)
C34-C35-C36-C37	0.8(5)	O4'-C49'-C50'-C51'	37(2)
C35-C34-C39-C38	0.2(4)	C49'-O4'-C52'-C51'	15(2)
C35-C36-C37-C38	-1.6(5)	C49'-C50'-C51'-C52'	-27(2)
C36-C37-C38-C39	1.8(5)	C50'-C51'-C52'-O4'	9.3(19)
C37-C38-C39-C34	-1.1(5)	C52'-O4'-C49'-C50'	-33(2)
C39-C34-C35-C36	-0.1(5)	O5'-C53'-C54'-C55'	19(2)
O2-C41-C42-C43	-31.2(5)	C53'-O5'-C56'-C55'	21(3)
C41-O2-C44-C43	1.4(5)	C53'-C54'-C55'-C56'	-5(2)
C41-C42-C43-C44	30.8(5)	C54'-C55'-C56'-O5'	-10(3)
C42-C43-C44-O2	-20.8(5)	C56'-O5'-C53'-C54'	-25(2)
C44-O2-C41-C42	18.8(5)	O6'-C57'-C58'-C59'	27(3)
O3-C45-C46-C47	-15.4(18)	C57'-O6'-C60'-C59'	56.6(15)
C45-O3-C48-C47	27(2)	C57'-C58'-C59'-C60'	8(2)
C45-C46-C47-C48	30.0(14)	C58'-C59'-C60'-O6'	-42(2)
C46-C47-C48-O3	-35.1(16)	C60'-O6'-C57'-C58'	-52(2)
C48-O3-C45-C46	-7(2)		
O4-C49-C50-C51	-36.8(12)		
C49-O4-C52-C51	-13.1(9)		
C49-C50-C51-C52	27.5(12)		
C50-C51-C52-O4	-10.3(11)		

3.4 Bisalkylated Fe(II)-Xantphos (3-CH_2SiMe_3)

CRYSTAL STRUCTURE REPORT

C_{47} H_{54} Fe O P_2 Si_2

or

(Xantphos)Fe(CH_2SiMe_3)$_2$

Report prepared for:

M. Gimeno, Prof. M. Neidig

August 22, 2022

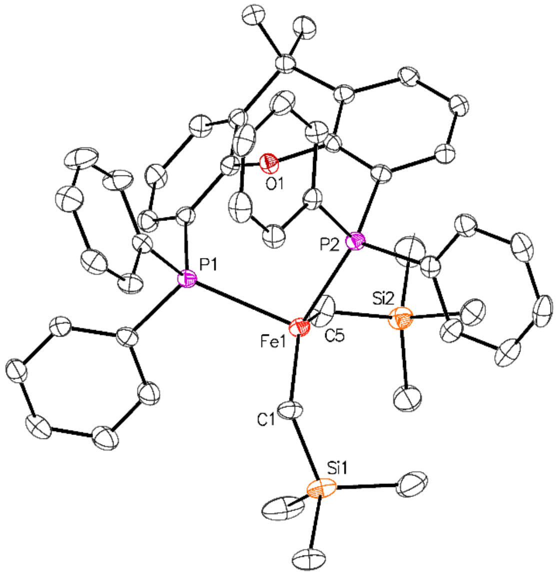

William W. Brennessel

X-ray Crystallographic Facility

Department of Chemistry, University of Rochester

120 Trustee Road

Rochester, NY 14627

Data collection

A crystal (0.214 x 0.162 x 0.033 mm^3) was placed onto a thin glass optical fiber or a nylon loop and mounted on a Rigaku XtaLAB Synergy-S Dualflex diffractometer equipped with a HyPix-6000HE HPC area detector for data collection at 99.99(10) K. A preliminary set of cell constants and an orientation matrix were calculated from a small sampling of reflections.[1] A short pre-experiment was run, from which an optimal data collection strategy was determined. The full data collection was carried out using a PhotonJet (Cu) X-ray source with frame times of 0.47 and 1.89 seconds and a detector distance of 34.0 mm. Series of frames were collected in 0.50º steps in ω at different 2θ, κ, and ϕ settings. After the intensity data were corrected for absorption, the final cell constants were calculated from the xyz centroids of 38964 strong reflections from the actual data collection after integration.[1] See Table 1 for additional crystal and refinement information.

Structure solution and refinement

The structure was solved using SHELXT[2] and refined using SHELXL.[3] The space group $P2_1/n$ was determined based on systematic absences. Most or all non-hydrogen atoms were assigned from the solution. Full-matrix least squares / difference Fourier cycles were performed which located any remaining non-hydrogen atoms. All non-hydrogen atoms were refined with anisotropic displacement parameters. All hydrogen atoms were placed in ideal positions and refined as riding atoms with relative isotropic displacement parameters. The final full matrix least squares refinement converged to $R1 = 0.0358$ (F^2, $I > 2\sigma(I)$) and $wR2 = 0.0970$ (F^2, all data).

Structure description

The structure is the one suggested. The asymmetric unit contains one molecule in a general position.

Structure manipulation and figure generation were performed using Olex2.[4] Unless noted otherwise all structural diagrams containing anisotropic displacement ellipsoids are drawn at the 50 % probability level.

Data collection, structure solution, and structure refinement were conducted at the X-ray Crystallographic Facility, B04 Hutchison Hall, Department of Chemistry, University of Rochester. The instrument was purchased with funding from NSF MRI program grant CHE-1725028. All publications arising from this report MUST either 1) include William W. Brennessel as a coauthor or 2) acknowledge William W. Brennessel and the X-ray Crystallographic Facility of the Department of Chemistry at the University of Rochester.

[1] *CrysAlisPro*, version 171.42.64a; Rigaku Corporation: Oxford, UK, 2022.

[2] Sheldrick, G. M. *SHELXT*, version 2018/2; *Acta. Crystallogr.* **2015**, *A71*, 3-8.

[3] Sheldrick, G. M. *SHELXL*, version 2019/2; *Acta. Crystallogr.* **2015**, *C71*, 3-8.

[4] Dolomanov, O. V.; Bourhis, L. J.; Gildea, R. J.; Howard, J. A. K.; Puschmann, H. *Olex2*, version 1.5; *J. Appl. Cryst.* **2009**, *42*, 339-341.

Some equations of interest:

$$R_{int} = \Sigma |F_o^2 - \langle F_o^2 \rangle| / \Sigma|F_o^2|$$

$$R1 = \Sigma||F_o|-|F_c|| / \Sigma|F_o|$$

$$wR2 = [\Sigma[w(F_o^2-F_c^2)^2] / \Sigma [w(F_o^2)^2]]^{1/2}$$

where $w = 1 / [\sigma^2 (F_o^2) + (aP)^2 + bP]$ and

$$P = 1/3 \max (0, F_o^2) + 2/3 F_c^2$$

$$GOF = S = [\Sigma[w(F_o^2-F_c^2)^2] / (m-n)]^{1/2}$$

where m = number of reflections and n = number of parameters

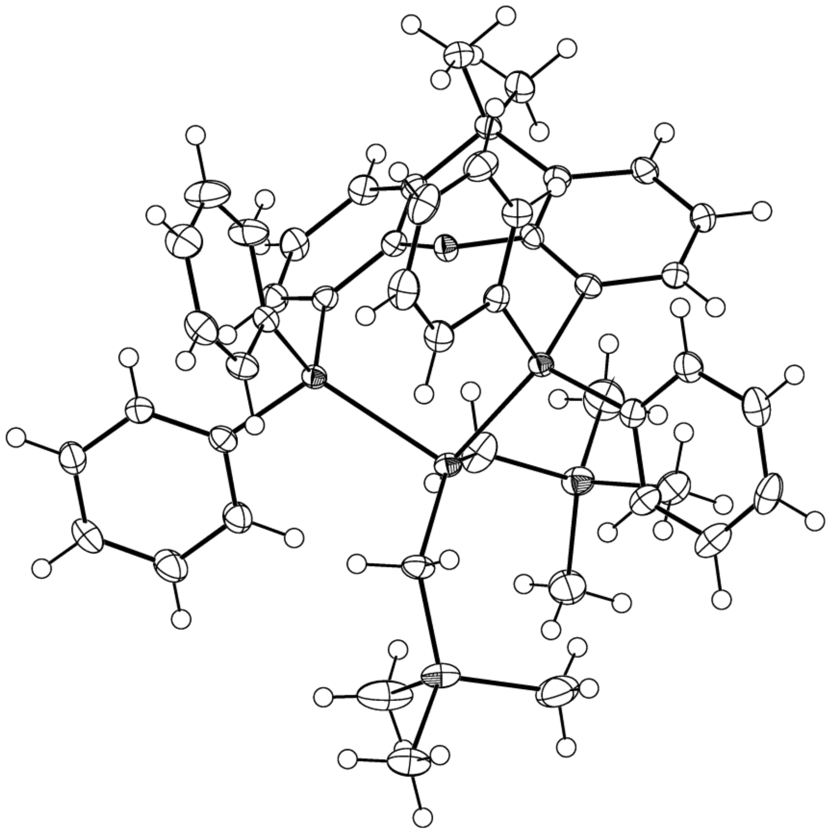

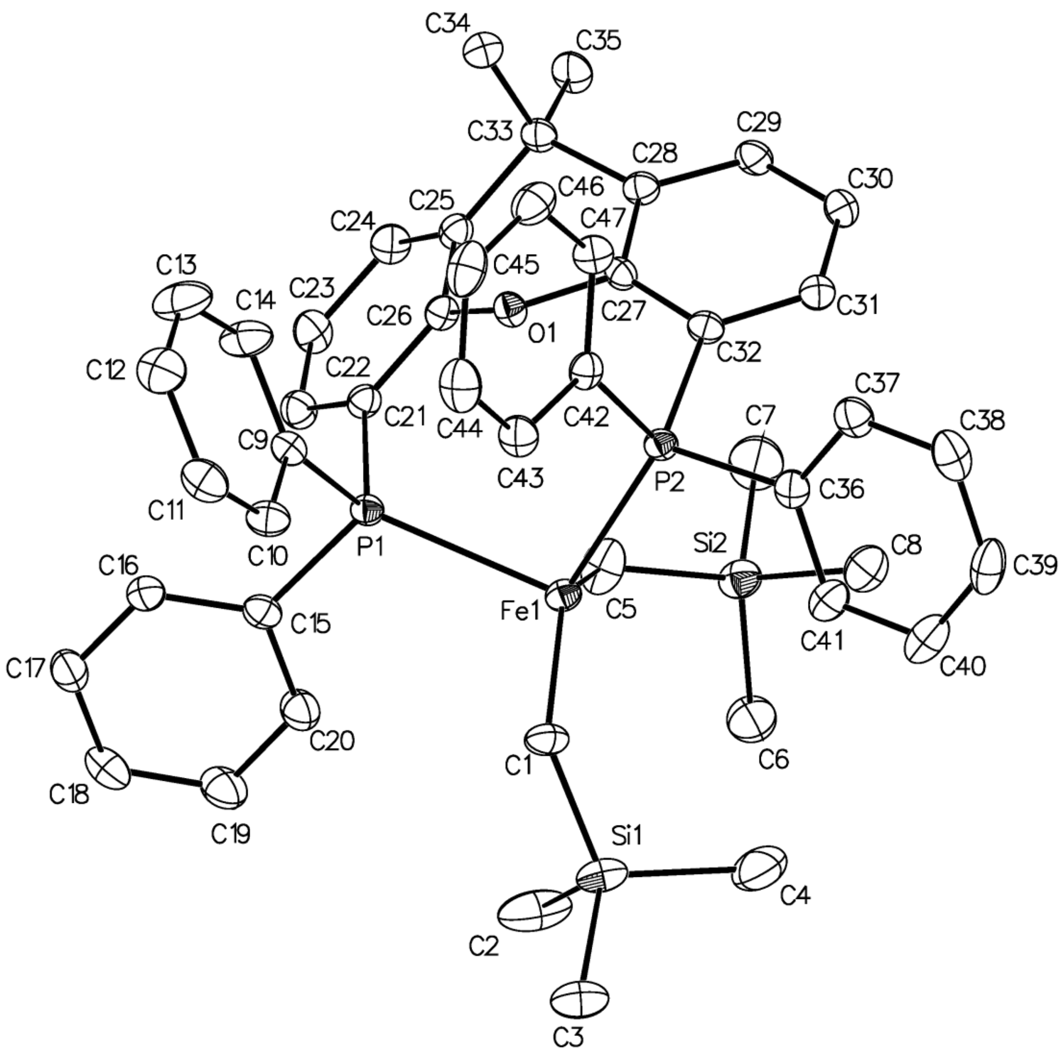
C34
C35
C33
C28
C29
C30
C46
C25
C24
C47
C13
C45
C14
C23
C26
O1
C27
C31
C32
C12
C22
C37
C7
C21
C44
C42
C38
C9
C43
P2
C36
C11
P1
C10
Si2
C8
C39
C16
Fe1
C5
C15
C41
C40
C17
C20
C6
C1
C18
C19
Si1
C4
C2
C3

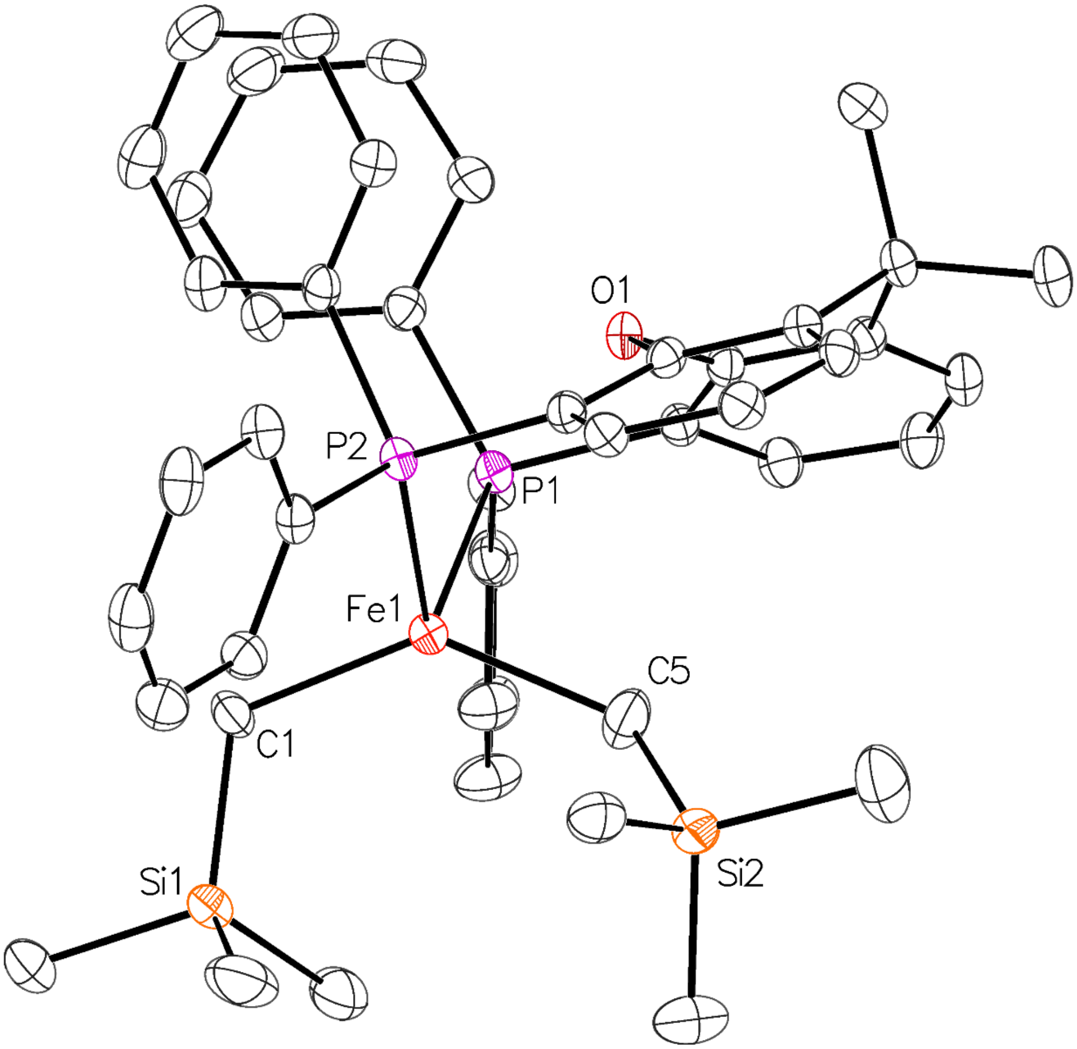
O1
P2
P1
Fe1
C5
C1
Si1
Si2

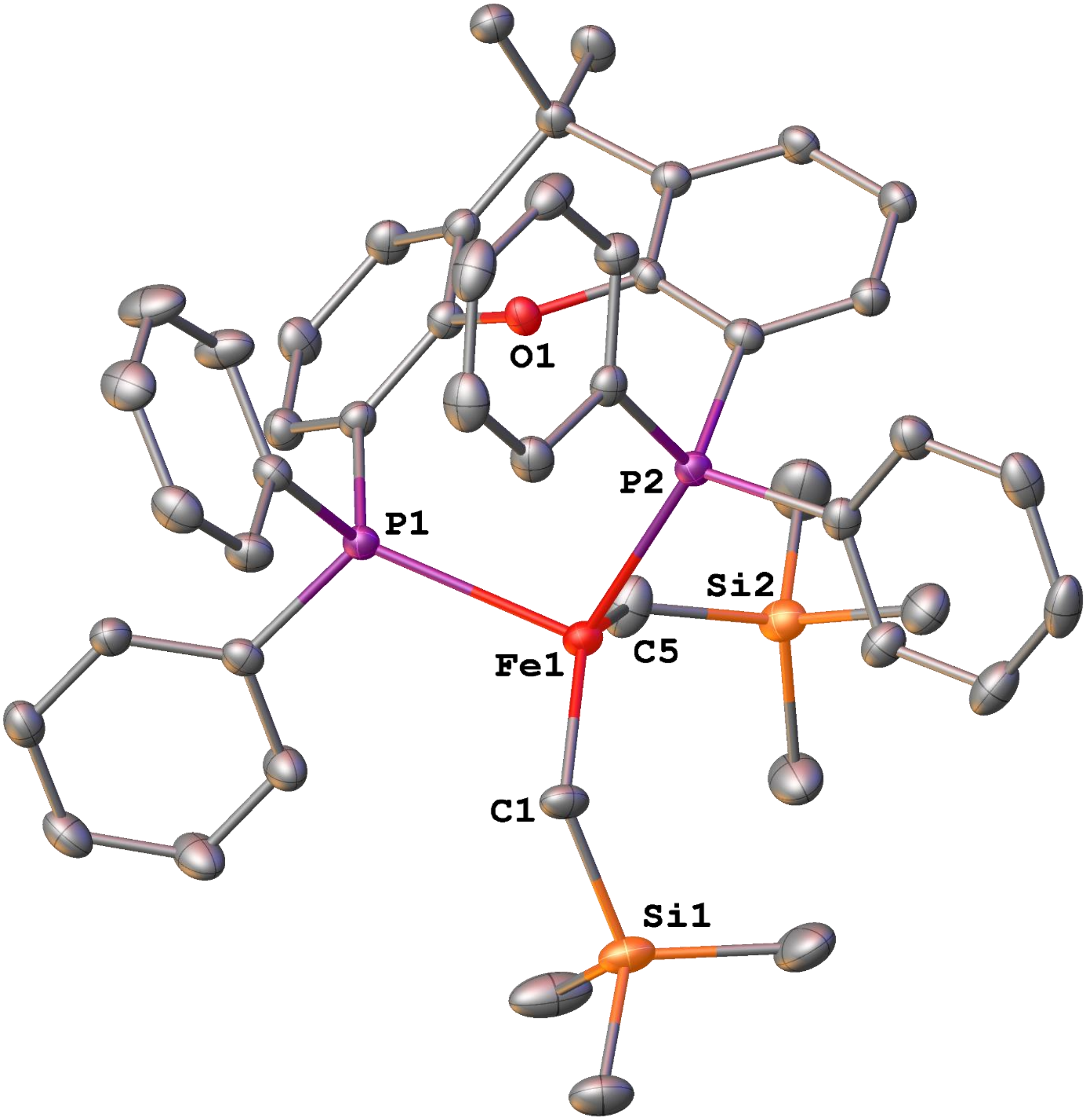
O1
P2
P1
Si2
C5
Fe1
C1
Si1

Table 1. Crystal data and structure refinement for neimg03.

Identification code	neimg03	
Empirical formula	C47 H54 Fe O P2 Si2	
Formula weight	808.87	
Temperature	99.99(10) K	
Wavelength	1.54184 Å	
Crystal system	monoclinic	
Space group	$P2_1/n$	
Unit cell dimensions	*a* = 12.56580(10) Å	α = 90°
	b = 19.11120(10) Å	β = 105.9190(10)°
	c = 19.21520(10) Å	γ = 90°
Volume	4437.52(5) $Å^3$	
Z	4	
Density (calculated)	1.211 Mg/m^3	
Absorption coefficient	4.170 mm^{-1}	
F(000)	1712	
Crystal color, morphology	yellow, plate	
Crystal size	0.214 x 0.162 x 0.033 mm^3	
Theta range for data collection	3.327 to 80.305°	
Index ranges	$-15 \leq h \leq 16$, $-19 \leq k \leq 23$, $-24 \leq l \leq 24$	
Reflections collected	75912	
Independent reflections	9558 [*R*(int) = 0.0469]	
Observed reflections	8861	
Completeness to theta = 74.504°	100.0%	
Absorption correction	Multi-scan	
Max. and min. transmission	1.00000 and 0.62830	
Refinement method	Full-matrix least-squares on F^2	
Data / restraints / parameters	9558 / 0 / 486	
Goodness-of-fit on F^2	1.084	
Final *R* indices [*I*>2sigma(*I*)]	*R*1 = 0.0358, *wR*2 = 0.0953	
R indices (all data)	*R*1 = 0.0385, *wR*2 = 0.0970	
Largest diff. peak and hole	0.730 and -0.408 e.$Å^{-3}$	

Table 2. Atomic coordinates (x 10^4) and equivalent isotropic displacement parameters ($Å^2$x 10^3) for neimg03. U_{eq} is defined as one third of the trace of the orthogonalized U_{ij} tensor.

	x	y	z	U_{eq}
Fe1	4539(1)	6345(1)	2254(1)	16(1)
P1	5832(1)	5387(1)	2087(1)	15(1)
P2	5743(1)	7385(1)	2256(1)	16(1)
Si1	1813(1)	6306(1)	1381(1)	25(1)
Si2	4182(1)	6612(1)	3997(1)	23(1)
O1	7360(1)	6387(1)	2976(1)	16(1)
C1	3247(1)	6354(1)	1302(1)	22(1)
C2	1614(2)	5504(1)	1888(1)	46(1)
C3	724(2)	6302(1)	484(1)	34(1)
C4	1477(2)	7071(1)	1901(1)	44(1)
C5	4664(2)	6112(1)	3326(1)	31(1)
C6	2814(2)	6272(1)	4067(1)	38(1)
C7	5205(2)	6540(1)	4918(1)	45(1)
C8	3989(2)	7572(1)	3785(1)	30(1)
C9	6641(1)	5396(1)	1427(1)	18(1)
C10	6056(1)	5458(1)	698(1)	22(1)
C11	6598(2)	5433(1)	161(1)	27(1)
C12	7740(2)	5349(1)	346(1)	31(1)
C13	8326(2)	5292(1)	1065(1)	38(1)
C14	7782(1)	5317(1)	1607(1)	29(1)
C15	5123(1)	4545(1)	1873(1)	19(1)
C16	5590(1)	3980(1)	1602(1)	21(1)
C17	5043(2)	3343(1)	1470(1)	24(1)
C18	4014(2)	3264(1)	1597(1)	27(1)
C19	3534(2)	3827(1)	1856(1)	32(1)
C20	4089(2)	4461(1)	1995(1)	26(1)
C21	6835(1)	5197(1)	2958(1)	16(1)
C22	6893(1)	4558(1)	3319(1)	21(1)
C23	7569(2)	4486(1)	4024(1)	23(1)
C24	8202(1)	5044(1)	4372(1)	21(1)
C25	8182(1)	5686(1)	4024(1)	18(1)

C26	7474(1)	5746(1)	3328(1)	16(1)
C27	7414(1)	6962(1)	3426(1)	15(1)
C28	8144(1)	6958(1)	4118(1)	17(1)
C29	8144(1)	7548(1)	4548(1)	19(1)
C30	7466(1)	8117(1)	4285(1)	20(1)
C31	6754(1)	8104(1)	3588(1)	19(1)
C32	6710(1)	7519(1)	3148(1)	17(1)
C33	8887(1)	6320(1)	4337(1)	18(1)
C34	9851(1)	6371(1)	3984(1)	22(1)
C35	9366(2)	6270(1)	5157(1)	25(1)
C36	5013(1)	8221(1)	2063(1)	19(1)
C37	5554(2)	8847(1)	2007(1)	22(1)
C38	4962(2)	9466(1)	1845(1)	28(1)
C39	3819(2)	9465(1)	1730(1)	31(1)
C40	3272(2)	8846(1)	1780(1)	31(1)
C41	3867(1)	8224(1)	1946(1)	23(1)
C42	6656(1)	7413(1)	1660(1)	17(1)
C43	6230(2)	7174(1)	951(1)	23(1)
C44	6878(2)	7187(1)	473(1)	30(1)
C45	7963(2)	7423(1)	699(1)	30(1)
C46	8393(2)	7659(1)	1403(1)	27(1)
C47	7740(1)	7656(1)	1882(1)	22(1)

Table 3. Bond lengths [Å] and angles [°] for neimg03.

Fe(1)-P(1)	2.5246(5)	C(6)-H(6B)	0.9800
Fe(1)-P(2)	2.4981(5)	C(6)-H(6C)	0.9800
Fe(1)-C(1)	2.0867(17)	C(7)-H(7A)	0.9800
Fe(1)-C(5)	2.0708(18)	C(7)-H(7B)	0.9800
P(1)-C(9)	1.8305(16)	C(7)-H(7C)	0.9800
P(1)-C(15)	1.8314(17)	C(8)-H(8A)	0.9800
P(1)-C(21)	1.8340(16)	C(8)-H(8B)	0.9800
P(2)-C(32)	1.8264(16)	C(8)-H(8C)	0.9800
P(2)-C(36)	1.8282(17)	C(9)-C(10)	1.397(2)
P(2)-C(42)	1.8312(16)	C(9)-C(14)	1.388(2)
Si(1)-C(1)	1.8528(18)	C(10)-H(10)	0.9500
Si(1)-C(2)	1.868(2)	C(10)-C(11)	1.385(2)
Si(1)-C(3)	1.884(2)	C(11)-H(11)	0.9500
Si(1)-C(4)	1.884(2)	C(11)-C(12)	1.390(3)
Si(2)-C(5)	1.8371(19)	C(12)-H(12)	0.9500
Si(2)-C(6)	1.878(2)	C(12)-C(13)	1.379(3)
Si(2)-C(7)	1.885(2)	C(13)-H(13)	0.9500
Si(2)-C(8)	1.880(2)	C(13)-C(14)	1.396(3)
O(1)-C(26)	1.3875(19)	C(14)-H(14)	0.9500
O(1)-C(27)	1.3883(19)	C(15)-C(16)	1.395(2)
C(1)-H(1A)	0.9900	C(15)-C(20)	1.392(2)
C(1)-H(1B)	0.9900	C(16)-H(16)	0.9500
C(2)-H(2A)	0.9800	C(16)-C(17)	1.386(3)
C(2)-H(2B)	0.9800	C(17)-H(17)	0.9500
C(2)-H(2C)	0.9800	C(17)-C(18)	1.389(3)
C(3)-H(3A)	0.9800	C(18)-H(18)	0.9500
C(3)-H(3B)	0.9800	C(18)-C(19)	1.390(3)
C(3)-H(3C)	0.9800	C(19)-H(19)	0.9500
C(4)-H(4A)	0.9800	C(19)-C(20)	1.386(3)
C(4)-H(4B)	0.9800	C(20)-H(20)	0.9500
C(4)-H(4C)	0.9800	C(21)-C(22)	1.397(2)
C(5)-H(5A)	0.9900	C(21)-C(26)	1.392(2)
C(5)-H(5B)	0.9900	C(22)-H(22)	0.9500
C(6)-H(6A)	0.9800	C(22)-C(23)	1.394(2)

C(23)-H(23)	0.9500	C(42)-C(47)	1.392(2)
C(23)-C(24)	1.387(2)	C(43)-H(43)	0.9500
C(24)-H(24)	0.9500	C(43)-C(44)	1.384(3)
C(24)-C(25)	1.395(2)	C(44)-H(44)	0.9500
C(25)-C(26)	1.393(2)	C(44)-C(45)	1.388(3)
C(25)-C(33)	1.523(2)	C(45)-H(45)	0.9500
C(27)-C(28)	1.394(2)	C(45)-C(46)	1.387(3)
C(27)-C(32)	1.392(2)	C(46)-H(46)	0.9500
C(28)-C(29)	1.396(2)	C(46)-C(47)	1.390(2)
C(28)-C(33)	1.524(2)	C(47)-H(47)	0.9500
C(29)-H(29)	0.9500	P(2)-Fe(1)-P(1)	99.628(15)
C(29)-C(30)	1.389(2)	C(1)-Fe(1)-P(1)	105.56(5)
C(30)-H(30)	0.9500	C(1)-Fe(1)-P(2)	108.64(5)
C(30)-C(31)	1.392(2)	C(5)-Fe(1)-P(1)	95.42(6)
C(31)-H(31)	0.9500	C(5)-Fe(1)-P(2)	106.78(7)
C(31)-C(32)	1.395(2)	C(5)-Fe(1)-C(1)	134.62(8)
C(33)-C(34)	1.545(2)	C(9)-P(1)-Fe(1)	125.48(5)
C(33)-C(35)	1.527(2)	C(9)-P(1)-C(15)	100.35(7)
C(34)-H(34A)	0.9800	C(9)-P(1)-C(21)	105.11(7)
C(34)-H(34B)	0.9800	C(15)-P(1)-Fe(1)	112.07(5)
C(34)-H(34C)	0.9800	C(15)-P(1)-C(21)	101.64(7)
C(35)-H(35A)	0.9800	C(21)-P(1)-Fe(1)	109.44(5)
C(35)-H(35B)	0.9800	C(32)-P(2)-Fe(1)	111.08(5)
C(35)-H(35C)	0.9800	C(32)-P(2)-C(36)	103.69(7)
C(36)-C(37)	1.395(2)	C(32)-P(2)-C(42)	102.31(7)
C(36)-C(41)	1.395(2)	C(36)-P(2)-Fe(1)	115.16(6)
C(37)-H(37)	0.9500	C(36)-P(2)-C(42)	102.32(7)
C(37)-C(38)	1.387(3)	C(42)-P(2)-Fe(1)	120.30(5)
C(38)-H(38)	0.9500	C(1)-Si(1)-C(2)	110.87(9)
C(38)-C(39)	1.392(3)	C(1)-Si(1)-C(3)	113.79(9)
C(39)-H(39)	0.9500	C(1)-Si(1)-C(4)	111.66(9)
C(39)-C(40)	1.384(3)	C(2)-Si(1)-C(3)	107.85(10)
C(40)-H(40)	0.9500	C(2)-Si(1)-C(4)	106.21(13)
C(40)-C(41)	1.393(3)	C(3)-Si(1)-C(4)	106.05(10)
C(41)-H(41)	0.9500	C(5)-Si(2)-C(6)	110.74(10)
C(42)-C(43)	1.396(2)	C(5)-Si(2)-C(7)	110.44(10)

C(5)-Si(2)-C(8)	113.83(9)	Si(2)-C(6)-H(6B)	109.5
C(6)-Si(2)-C(7)	108.04(12)	Si(2)-C(6)-H(6C)	109.5
C(6)-Si(2)-C(8)	106.69(10)	H(6A)-C(6)-H(6B)	109.5
C(8)-Si(2)-C(7)	106.83(11)	H(6A)-C(6)-H(6C)	109.5
C(26)-O(1)-C(27)	114.54(12)	H(6B)-C(6)-H(6C)	109.5
Fe(1)-C(1)-H(1A)	107.8	Si(2)-C(7)-H(7A)	109.5
Fe(1)-C(1)-H(1B)	107.8	Si(2)-C(7)-H(7B)	109.5
Si(1)-C(1)-Fe(1)	117.90(9)	Si(2)-C(7)-H(7C)	109.5
Si(1)-C(1)-H(1A)	107.8	H(7A)-C(7)-H(7B)	109.5
Si(1)-C(1)-H(1B)	107.8	H(7A)-C(7)-H(7C)	109.5
H(1A)-C(1)-H(1B)	107.2	H(7B)-C(7)-H(7C)	109.5
Si(1)-C(2)-H(2A)	109.5	Si(2)-C(8)-H(8A)	109.5
Si(1)-C(2)-H(2B)	109.5	Si(2)-C(8)-H(8B)	109.5
Si(1)-C(2)-H(2C)	109.5	Si(2)-C(8)-H(8C)	109.5
H(2A)-C(2)-H(2B)	109.5	H(8A)-C(8)-H(8B)	109.5
H(2A)-C(2)-H(2C)	109.5	H(8A)-C(8)-H(8C)	109.5
H(2B)-C(2)-H(2C)	109.5	H(8B)-C(8)-H(8C)	109.5
Si(1)-C(3)-H(3A)	109.5	C(10)-C(9)-P(1)	117.12(12)
Si(1)-C(3)-H(3B)	109.5	C(14)-C(9)-P(1)	123.93(13)
Si(1)-C(3)-H(3C)	109.5	C(14)-C(9)-C(10)	118.90(15)
H(3A)-C(3)-H(3B)	109.5	C(9)-C(10)-H(10)	119.6
H(3A)-C(3)-H(3C)	109.5	C(11)-C(10)-C(9)	120.83(16)
H(3B)-C(3)-H(3C)	109.5	C(11)-C(10)-H(10)	119.6
Si(1)-C(4)-H(4A)	109.5	C(10)-C(11)-H(11)	120.0
Si(1)-C(4)-H(4B)	109.5	C(10)-C(11)-C(12)	119.92(16)
Si(1)-C(4)-H(4C)	109.5	C(12)-C(11)-H(11)	120.0
H(4A)-C(4)-H(4B)	109.5	C(11)-C(12)-H(12)	120.2
H(4A)-C(4)-H(4C)	109.5	C(13)-C(12)-C(11)	119.65(17)
H(4B)-C(4)-H(4C)	109.5	C(13)-C(12)-H(12)	120.2
Fe(1)-C(5)-H(5A)	104.9	C(12)-C(13)-H(13)	119.7
Fe(1)-C(5)-H(5B)	104.9	C(12)-C(13)-C(14)	120.60(18)
Si(2)-C(5)-Fe(1)	129.56(11)	C(14)-C(13)-H(13)	119.7
Si(2)-C(5)-H(5A)	104.9	C(9)-C(14)-C(13)	120.09(17)
Si(2)-C(5)-H(5B)	104.9	C(9)-C(14)-H(14)	120.0
H(5A)-C(5)-H(5B)	105.8	C(13)-C(14)-H(14)	120.0
Si(2)-C(6)-H(6A)	109.5	C(16)-C(15)-P(1)	122.41(13)

C(20)-C(15)-P(1)	118.83(13)	O(1)-C(27)-C(32)	116.68(13)
C(20)-C(15)-C(16)	118.75(16)	C(32)-C(27)-C(28)	123.54(15)
C(15)-C(16)-H(16)	119.7	C(27)-C(28)-C(29)	116.97(15)
C(17)-C(16)-C(15)	120.65(16)	C(27)-C(28)-C(33)	117.44(14)
C(17)-C(16)-H(16)	119.7	C(29)-C(28)-C(33)	125.58(14)
C(16)-C(17)-H(17)	119.9	C(28)-C(29)-H(29)	119.4
C(16)-C(17)-C(18)	120.11(17)	C(30)-C(29)-C(28)	121.10(15)
C(18)-C(17)-H(17)	119.9	C(30)-C(29)-H(29)	119.4
C(17)-C(18)-H(18)	120.2	C(29)-C(30)-H(30)	119.9
C(17)-C(18)-C(19)	119.67(17)	C(29)-C(30)-C(31)	120.30(15)
C(19)-C(18)-H(18)	120.2	C(31)-C(30)-H(30)	119.9
C(18)-C(19)-H(19)	120.0	C(30)-C(31)-H(31)	119.8
C(20)-C(19)-C(18)	119.99(17)	C(30)-C(31)-C(32)	120.34(15)
C(20)-C(19)-H(19)	120.0	C(32)-C(31)-H(31)	119.8
C(15)-C(20)-H(20)	119.6	C(27)-C(32)-P(2)	116.26(12)
C(19)-C(20)-C(15)	120.82(17)	C(27)-C(32)-C(31)	117.73(14)
C(19)-C(20)-H(20)	119.6	C(31)-C(32)-P(2)	125.87(12)
C(22)-C(21)-P(1)	123.72(12)	C(25)-C(33)-C(28)	106.35(13)
C(26)-C(21)-P(1)	118.24(12)	C(25)-C(33)-C(34)	108.52(13)
C(26)-C(21)-C(22)	117.46(14)	C(25)-C(33)-C(35)	112.32(14)
C(21)-C(22)-H(22)	119.9	C(28)-C(33)-C(34)	108.85(13)
C(23)-C(22)-C(21)	120.26(16)	C(28)-C(33)-C(35)	111.89(13)
C(23)-C(22)-H(22)	119.9	C(35)-C(33)-C(34)	108.80(14)
C(22)-C(23)-H(23)	119.7	C(33)-C(34)-H(34A)	109.5
C(24)-C(23)-C(22)	120.53(16)	C(33)-C(34)-H(34B)	109.5
C(24)-C(23)-H(23)	119.7	C(33)-C(34)-H(34C)	109.5
C(23)-C(24)-H(24)	119.6	H(34A)-C(34)-H(34B)	109.5
C(23)-C(24)-C(25)	120.88(15)	H(34A)-C(34)-H(34C)	109.5
C(25)-C(24)-H(24)	119.6	H(34B)-C(34)-H(34C)	109.5
C(24)-C(25)-C(33)	125.36(14)	C(33)-C(35)-H(35A)	109.5
C(26)-C(25)-C(24)	117.03(15)	C(33)-C(35)-H(35B)	109.5
C(26)-C(25)-C(33)	117.60(14)	C(33)-C(35)-H(35C)	109.5
O(1)-C(26)-C(21)	116.62(13)	H(35A)-C(35)-H(35B)	109.5
O(1)-C(26)-C(25)	119.58(14)	H(35A)-C(35)-H(35C)	109.5
C(21)-C(26)-C(25)	123.78(15)	H(35B)-C(35)-H(35C)	109.5
O(1)-C(27)-C(28)	119.78(14)	C(37)-C(36)-P(2)	122.44(13)

C(41)-C(36)-P(2)	118.35(13)	C(47)-C(42)-P(2)	123.15(12)
C(41)-C(36)-C(37)	119.19(16)	C(47)-C(42)-C(43)	119.27(15)
C(36)-C(37)-H(37)	119.8	C(42)-C(43)-H(43)	119.9
C(38)-C(37)-C(36)	120.41(17)	C(44)-C(43)-C(42)	120.24(17)
C(38)-C(37)-H(37)	119.8	C(44)-C(43)-H(43)	119.9
C(37)-C(38)-H(38)	120.0	C(43)-C(44)-H(44)	119.8
C(37)-C(38)-C(39)	120.01(18)	C(43)-C(44)-C(45)	120.31(17)
C(39)-C(38)-H(38)	120.0	C(45)-C(44)-H(44)	119.8
C(38)-C(39)-H(39)	120.0	C(44)-C(45)-H(45)	120.1
C(40)-C(39)-C(38)	120.08(17)	C(46)-C(45)-C(44)	119.75(17)
C(40)-C(39)-H(39)	120.0	C(46)-C(45)-H(45)	120.1
C(39)-C(40)-H(40)	120.0	C(45)-C(46)-H(46)	119.9
C(39)-C(40)-C(41)	119.95(18)	C(45)-C(46)-C(47)	120.12(17)
C(41)-C(40)-H(40)	120.0	C(47)-C(46)-H(46)	119.9
C(36)-C(41)-H(41)	119.8	C(42)-C(47)-H(47)	119.9
C(40)-C(41)-C(36)	120.36(18)	C(46)-C(47)-C(42)	120.30(16)
C(40)-C(41)-H(41)	119.8	C(46)-C(47)-H(47)	119.9
C(43)-C(42)-P(2)	117.57(13)		

Table 4. Anisotropic displacement parameters ($Å^2 x 10^3$) for neimg03. The anisotropic displacement factor exponent takes the form: $-2\pi^2[h^2 a^{*2}U_{11} + ... + 2 h k a^* b^* U_{12}]$

	U_{11}	U_{22}	U_{33}	U_{23}	U_{13}	U_{12}
Fe1	14(1)	19(1)	15(1)	2(1)	3(1)	1(1)
P1	15(1)	16(1)	14(1)	0(1)	2(1)	0(1)
P2	15(1)	16(1)	14(1)	1(1)	1(1)	1(1)
Si1	15(1)	35(1)	24(1)	7(1)	3(1)	0(1)
Si2	21(1)	31(1)	19(1)	0(1)	7(1)	0(1)
O1	19(1)	15(1)	12(1)	0(1)	2(1)	1(1)
C1	15(1)	24(1)	23(1)	4(1)	-1(1)	-1(1)
C2	23(1)	59(2)	52(1)	28(1)	4(1)	-4(1)
C3	21(1)	45(1)	31(1)	6(1)	-1(1)	-2(1)
C4	24(1)	63(2)	48(1)	-10(1)	12(1)	4(1)
C5	40(1)	34(1)	22(1)	7(1)	14(1)	12(1)
C6	32(1)	41(1)	47(1)	-9(1)	22(1)	-8(1)
C7	45(1)	52(1)	29(1)	-4(1)	-3(1)	10(1)
C8	29(1)	33(1)	31(1)	-1(1)	13(1)	3(1)
C9	18(1)	18(1)	16(1)	-1(1)	4(1)	-2(1)
C10	20(1)	27(1)	18(1)	1(1)	2(1)	-2(1)
C11	31(1)	30(1)	18(1)	-1(1)	5(1)	-6(1)
C12	31(1)	40(1)	26(1)	-3(1)	15(1)	-5(1)
C13	20(1)	64(2)	31(1)	-2(1)	9(1)	-2(1)
C14	18(1)	48(1)	20(1)	1(1)	3(1)	-1(1)
C15	18(1)	21(1)	15(1)	0(1)	1(1)	-1(1)
C16	18(1)	21(1)	23(1)	-1(1)	2(1)	1(1)
C17	28(1)	20(1)	23(1)	-3(1)	3(1)	0(1)
C18	32(1)	25(1)	24(1)	-4(1)	7(1)	-11(1)
C19	30(1)	34(1)	35(1)	-9(1)	16(1)	-12(1)
C20	26(1)	26(1)	28(1)	-7(1)	12(1)	-5(1)
C21	16(1)	18(1)	15(1)	1(1)	3(1)	2(1)
C22	22(1)	18(1)	20(1)	0(1)	4(1)	1(1)
C23	28(1)	17(1)	22(1)	4(1)	4(1)	2(1)
C24	23(1)	23(1)	15(1)	2(1)	1(1)	3(1)
C25	17(1)	20(1)	15(1)	-1(1)	4(1)	3(1)

C26	17(1)	16(1)	14(1)	2(1)	4(1)	2(1)
C27	17(1)	16(1)	13(1)	-2(1)	4(1)	-2(1)
C28	16(1)	20(1)	14(1)	0(1)	3(1)	0(1)
C29	19(1)	23(1)	14(1)	-3(1)	2(1)	-1(1)
C30	21(1)	21(1)	18(1)	-5(1)	5(1)	0(1)
C31	18(1)	19(1)	18(1)	-1(1)	4(1)	1(1)
C32	16(1)	18(1)	14(1)	0(1)	3(1)	-1(1)
C33	18(1)	19(1)	14(1)	-1(1)	0(1)	1(1)
C34	18(1)	24(1)	24(1)	-1(1)	3(1)	1(1)
C35	28(1)	26(1)	15(1)	-2(1)	-3(1)	4(1)
C36	22(1)	19(1)	14(1)	0(1)	3(1)	3(1)
C37	25(1)	22(1)	18(1)	0(1)	2(1)	1(1)
C38	41(1)	19(1)	19(1)	1(1)	2(1)	2(1)
C39	43(1)	26(1)	21(1)	2(1)	6(1)	18(1)
C40	28(1)	37(1)	29(1)	4(1)	11(1)	13(1)
C41	22(1)	26(1)	22(1)	3(1)	6(1)	5(1)
C42	20(1)	16(1)	16(1)	3(1)	4(1)	4(1)
C43	26(1)	24(1)	17(1)	0(1)	2(1)	3(1)
C44	43(1)	29(1)	19(1)	0(1)	10(1)	5(1)
C45	41(1)	26(1)	30(1)	6(1)	21(1)	8(1)
C46	23(1)	27(1)	34(1)	5(1)	13(1)	2(1)
C47	21(1)	23(1)	21(1)	1(1)	6(1)	0(1)

Table 5. Hydrogen coordinates (x 10^4) and isotropic displacement parameters ($Å^2$ x 10^3) for neimg03.

	x	y	z	U(eq)
H1A	3313	6788	1035	26
H1B	3358	5956	1000	26
H2A	1658	5088	1599	69
H2B	887	5522	1983	69
H2C	2194	5482	2348	69
H3A	785	6731	218	50
H3B	-11	6278	566	50
H3C	834	5894	202	50
H4A	2030	7104	2372	67
H4B	742	7006	1974	67
H4C	1485	7503	1628	67
H5A	4316	5646	3313	38
H5B	5464	6038	3552	38
H6A	2278	6282	3588	57
H6B	2546	6566	4401	57
H6C	2903	5790	4248	57
H7A	5265	6050	5074	67
H7B	4952	6824	5265	67
H7C	5931	6707	4892	67
H8A	4699	7779	3777	45
H8B	3715	7804	4157	45
H8C	3452	7634	3312	45
H10	5275	5518	569	27
H11	6190	5473	-333	32
H12	8115	5331	-21	37
H13	9107	5236	1191	45
H14	8193	5279	2101	35
H16	6288	4033	1507	25
H17	5374	2960	1292	29
H18	3639	2828	1508	33

H19	2826	3778	1938	38
H20	3760	4842	2176	31
H22	6470	4171	3083	25
H23	7597	4051	4267	27
H24	8654	4987	4853	26
H29	8616	7559	5027	23
H30	7489	8517	4582	24
H31	6294	8496	3412	22
H34A	10310	6778	4179	34
H34B	9552	6422	3459	34
H34C	10301	5945	4090	34
H35A	9827	5850	5276	37
H35B	8760	6243	5386	37
H35C	9816	6685	5333	37
H37	6333	8849	2081	27
H38	5336	9892	1813	33
H39	3414	9888	1617	37
H40	2492	8845	1701	37
H41	3490	7800	1980	28
H43	5493	7002	797	28
H44	6578	7033	-11	36
H45	8410	7424	372	36
H46	9134	7823	1558	32
H47	8036	7821	2362	26

Table 6. Torsion angles [°] for neimg03.

Fe1-P1-C9-C10	-58.45(15)	C9-P1-C15-C20	-150.91(14)
Fe1-P1-C9-C14	124.19(15)	C9-P1-C21-C22	-107.67(14)
Fe1-P1-C15-C16	165.42(12)	C9-P1-C21-C26	81.35(13)
Fe1-P1-C15-C20	-15.62(15)	C9-C10-C11-C12	-0.3(3)
Fe1-P1-C21-C22	115.20(13)	C10-C9-C14-C13	-0.8(3)
Fe1-P1-C21-C26	-55.78(13)	C10-C11-C12-C13	-0.1(3)
Fe1-P2-C32-C27	57.80(13)	C11-C12-C13-C14	0.1(4)
Fe1-P2-C32-C31	-117.75(14)	C12-C13-C14-C9	0.3(4)
Fe1-P2-C36-C37	-176.64(12)	C14-C9-C10-C11	0.8(3)
Fe1-P2-C36-C41	1.32(15)	C15-P1-C9-C10	68.37(14)
Fe1-P2-C42-C43	42.30(15)	C15-P1-C9-C14	-108.99(17)
Fe1-P2-C42-C47	-137.40(13)	C15-P1-C21-C22	-3.45(16)
P1-C9-C10-C11	-176.72(14)	C15-P1-C21-C26	-174.43(12)
P1-C9-C14-C13	176.56(17)	C15-C16-C17-C18	0.9(3)
P1-C15-C16-C17	177.72(13)	C16-C15-C20-C19	0.5(3)
P1-C15-C20-C19	-178.50(15)	C16-C17-C18-C19	0.1(3)
P1-C21-C22-C23	-170.90(13)	C17-C18-C19-C20	-0.8(3)
P1-C21-C26-O1	-5.04(19)	C18-C19-C20-C15	0.5(3)
P1-C21-C26-C25	173.34(12)	C20-C15-C16-C17	-1.2(2)
P2-C36-C37-C38	178.66(13)	C21-P1-C9-C10	173.54(13)
P2-C36-C41-C40	-178.46(14)	C21-P1-C9-C14	-3.82(18)
P2-C42-C43-C44	179.53(14)	C21-P1-C15-C16	-77.82(15)
P2-C42-C47-C46	179.56(14)	C21-P1-C15-C20	101.13(14)
O1-C27-C28-C29	-178.90(14)	C21-C22-C23-C24	-0.8(3)
O1-C27-C28-C33	2.3(2)	C22-C21-C26-O1	-176.59(14)
O1-C27-C32-P2	4.37(18)	C22-C21-C26-C25	1.8(2)
O1-C27-C32-C31	-179.71(14)	C22-C23-C24-C25	-0.5(3)
C2-Si1-C1-Fe1	-57.14(14)	C23-C24-C25-C26	2.2(2)
C3-Si1-C1-Fe1	-178.91(10)	C23-C24-C25-C33	-176.49(16)
C4-Si1-C1-Fe1	61.08(13)	C24-C25-C26-O1	175.37(14)
C6-Si2-C5-Fe1	97.89(17)	C24-C25-C26-C21	-3.0(2)
C7-Si2-C5-Fe1	-142.47(15)	C24-C25-C33-C28	-140.39(16)
C8-Si2-C5-Fe1	-22.31(19)	C24-C25-C33-C34	102.65(18)
C9-P1-C15-C16	30.13(15)	C24-C25-C33-C35	-17.7(2)

C26-O1-C27-C28	37.10(19)
C26-O1-C27-C32	-142.43(14)
C26-C21-C22-C23	0.1(2)
C26-C25-C33-C28	40.89(18)
C26-C25-C33-C34	-76.07(17)
C26-C25-C33-C35	163.60(15)
C27-O1-C26-C21	143.25(14)
C27-O1-C26-C25	-35.2(2)
C27-C28-C29-C30	-1.6(2)
C27-C28-C33-C25	-39.06(18)
C27-C28-C33-C34	77.68(17)
C27-C28-C33-C35	-162.05(15)
C28-C27-C32-P2	-175.14(12)
C28-C27-C32-C31	0.8(2)
C28-C29-C30-C31	1.2(3)
C29-C28-C33-C25	142.26(16)
C29-C28-C33-C34	-101.00(18)
C29-C28-C33-C35	19.3(2)
C29-C30-C31-C32	0.2(2)
C30-C31-C32-P2	174.30(13)
C30-C31-C32-C27	-1.2(2)
C32-P2-C36-C37	61.80(15)
C32-P2-C36-C41	-120.24(13)
C32-P2-C42-C43	165.94(13)
C32-P2-C42-C47	-13.76(16)
C32-C27-C28-C29	0.6(2)
C32-C27-C28-C33	-178.20(15)
C33-C25-C26-O1	-5.8(2)
C33-C25-C26-C21	175.87(14)
C33-C28-C29-C30	177.09(15)
C36-P2-C32-C27	-177.95(12)
C36-P2-C32-C31	6.51(16)
C36-P2-C42-C43	-86.87(14)
C36-P2-C42-C47	93.43(15)
C36-C37-C38-C39	-0.6(3)
C37-C36-C41-C40	-0.4(2)
C37-C38-C39-C40	0.2(3)
C38-C39-C40-C41	0.0(3)
C39-C40-C41-C36	0.1(3)
C41-C36-C37-C38	0.7(2)
C42-P2-C32-C27	-71.82(13)
C42-P2-C32-C31	112.64(15)
C42-P2-C36-C37	-44.33(15)
C42-P2-C36-C41	133.63(13)
C42-C43-C44-C45	1.4(3)
C43-C42-C47-C46	-0.1(3)
C43-C44-C45-C46	-1.1(3)
C44-C45-C46-C47	0.2(3)
C45-C46-C47-C42	0.4(3)
C47-C42-C43-C44	-0.7(3)

3.5 Fe(^{n}Bu)(κ^{2}P:(η^{6}-C_6H_5)-Xantphos (4)

CRYSTAL STRUCTURE REPORT

$C_{47} H_{49} Fe O_2 P_2$

or

(κ^{2}*P*:(*η*^{6}-*C_6H_5*)-Xantphos)Fe(C_4H_9) · THF

Report prepared for:

V. Fleischauer, Prof. M. Neidig

March 28, 2018

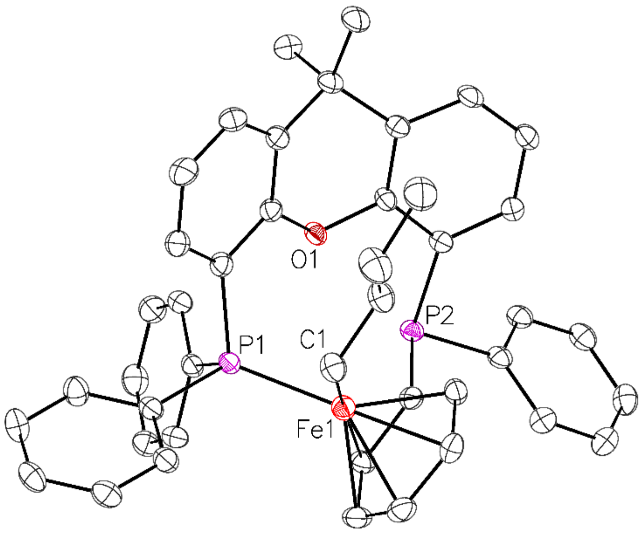

William W. Brennessel

X-ray Crystallographic Facility

Department of Chemistry, University of Rochester

120 Trustee Road

Rochester, NY 14627

Data collection

A crystal (0.152 x 0.055 x 0.041 mm^3) was placed onto a thin glass optical fiber or a nylon loop and mounted on a XtaLab Synergy-S Dualflex diffractometer equipped with a HyPix-6000HE HPC area detector for data collection at 100.01(10) K. A preliminary set of cell constants and an orientation matrix were calculated from a small sampling of reflections.[1] A short pre-experiment was run, from which an optimal data collection strategy was determined. The full data collection was carried out using a PhotonJet (Cu) X-ray Source with frame times of 0.40 and 1.58 seconds and a detector distance of 31.2 mm. Series of frames were collected in 0.50º steps in ω at different 2θ, κ, and ϕ settings. After the intensity data were corrected for absorption, the final cell constants were calculated from the xyz centroids of 36458 strong reflections from the actual data collection after integration.[1] See Table 1 for additional crystal and refinement information.

Structure solution and refinement

The structure was solved using ShelXT[2] and refined using ShelXL.[3] The space group *P*-1 was determined based on intensity statistics. Most or all non-hydrogen atoms were assigned from the solution. Full-matrix least squares / difference Fourier cycles were performed which located any remaining non-hydrogen atoms. All non-hydrogen atoms were refined with anisotropic displacement parameters. The hydrogen atoms on the iron-coordinating phenyl ring were found from the difference map and refined freely to confirm their presence. All other hydrogen atoms were placed in ideal positions and refined as riding atoms with relative isotropic displacement parameters. The final full matrix least squares refinement converged to $R1 = 0.0397$ (F^2, $I > 2\sigma(I)$) and $wR2 = 0.1118$ (F^2, all data).

Structure description

The structure is the one suggested. The asymmetric unit contains one iron complex and one cocrystallized THF solvent molecule in general positions.

Structure manipulation and figure generation were performed using Olex2.[4] Unless noted otherwise all structural diagrams containing thermal displacement ellipsoids are drawn at the 50 % probability level.

Data collection, structure solution, and structure refinement were conducted at the X-ray Crystallographic Facility, B04 Hutchison Hall, Department of Chemistry, University of Rochester. All publications arising from this report MUST either 1) include William W. Brennessel as a coauthor or 2) acknowledge William W. Brennessel and the X-ray Crystallographic Facility of the Department of Chemistry at the University of Rochester.

[1] *CrysAlisPro*, version 171.39.43c; Rigaku Corporation: Oxford, UK, 2018.

[2] Sheldrick, G. M. *SHELXT*, version 2018/2; *Acta. Crystallogr.* **2015**, *A71*, 3-8.

[3] Sheldrick, G. M. *SHELXL*, version 2018/3; *Acta. Crystallogr.* **2015**, *C71*, 3-8.

[4] Dolomanov, O. V.; Bourhis, L. J.; Gildea, R. J.; Howard, J. A. K.; Puschmann, H. *Olex2*, version 1.2-ac3; *J. Appl. Cryst.* **2009**, *42*, 339-341.

Some equations of interest:

$$R_{int} = \Sigma |F_o^2 - < F_o^2 >| / \Sigma|F_o^2|$$

$$R1 = \Sigma||F_o|-|F_c|| / \Sigma|F_o|$$

$$wR2 = [\Sigma[w(F_o^2-F_c^2)^2] / \Sigma [w(F_o^2)^2]]^{1/2}$$

where $w = 1 / [\sigma^2 (F_o^2) + (aP)^2 + bP]$ and

$$P = 1/3 \max (0, F_o^2) + 2/3 F_c^2$$

$$GOF = S = [\Sigma[w(F_o^2-F_c^2)^2] / (m-n)]^{1/2}$$

where m = number of reflections and n = number of parameters

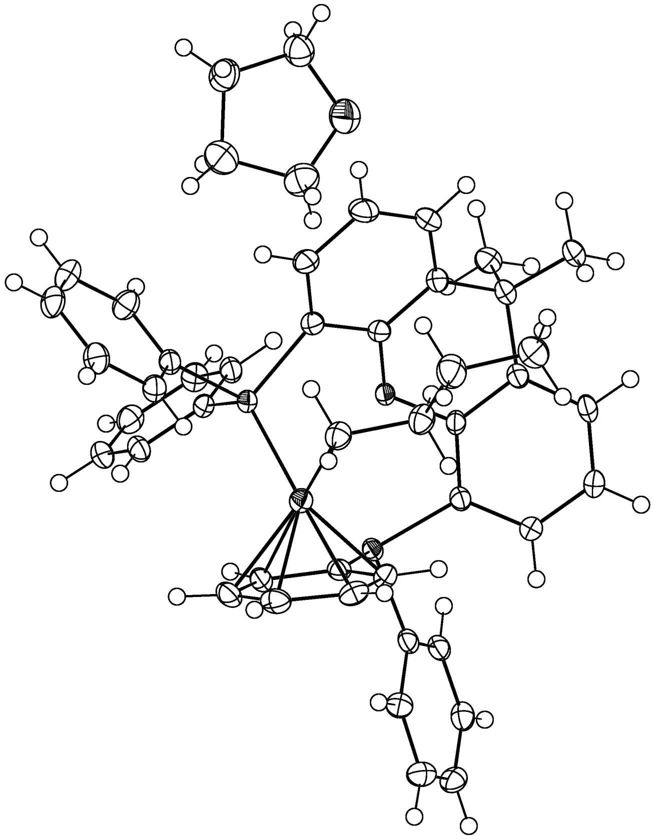

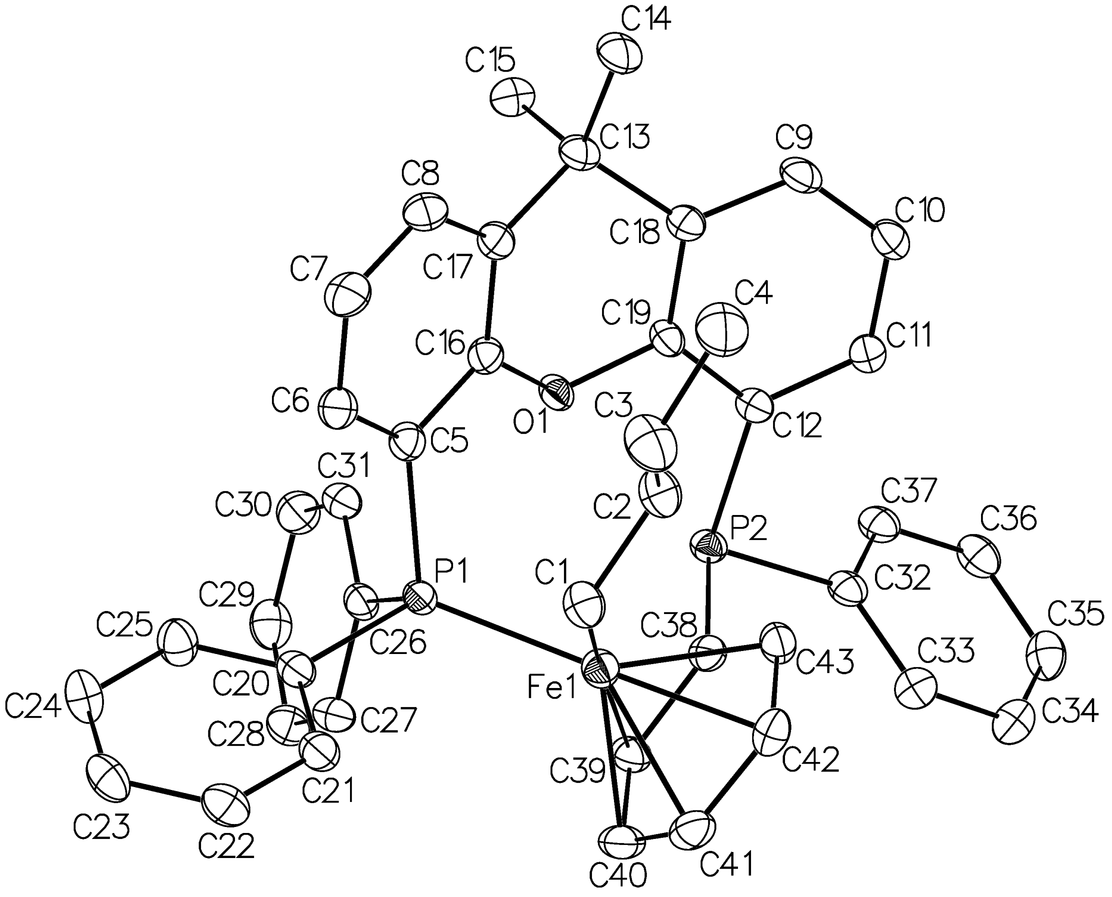
C14
C15
C13
C9
C8
C18
C10
C17
C7
C4
C19
C16
C11
C6
C3
O1
C12
C5
C31
C37
C30
C2
C36
P2
P1
C32
C1
C29
C35
C38
C26
C25
C43
C33
C20
Fe1
C24
C34
C28
C27
C42
C39
C21
C23
C22
C40
C41

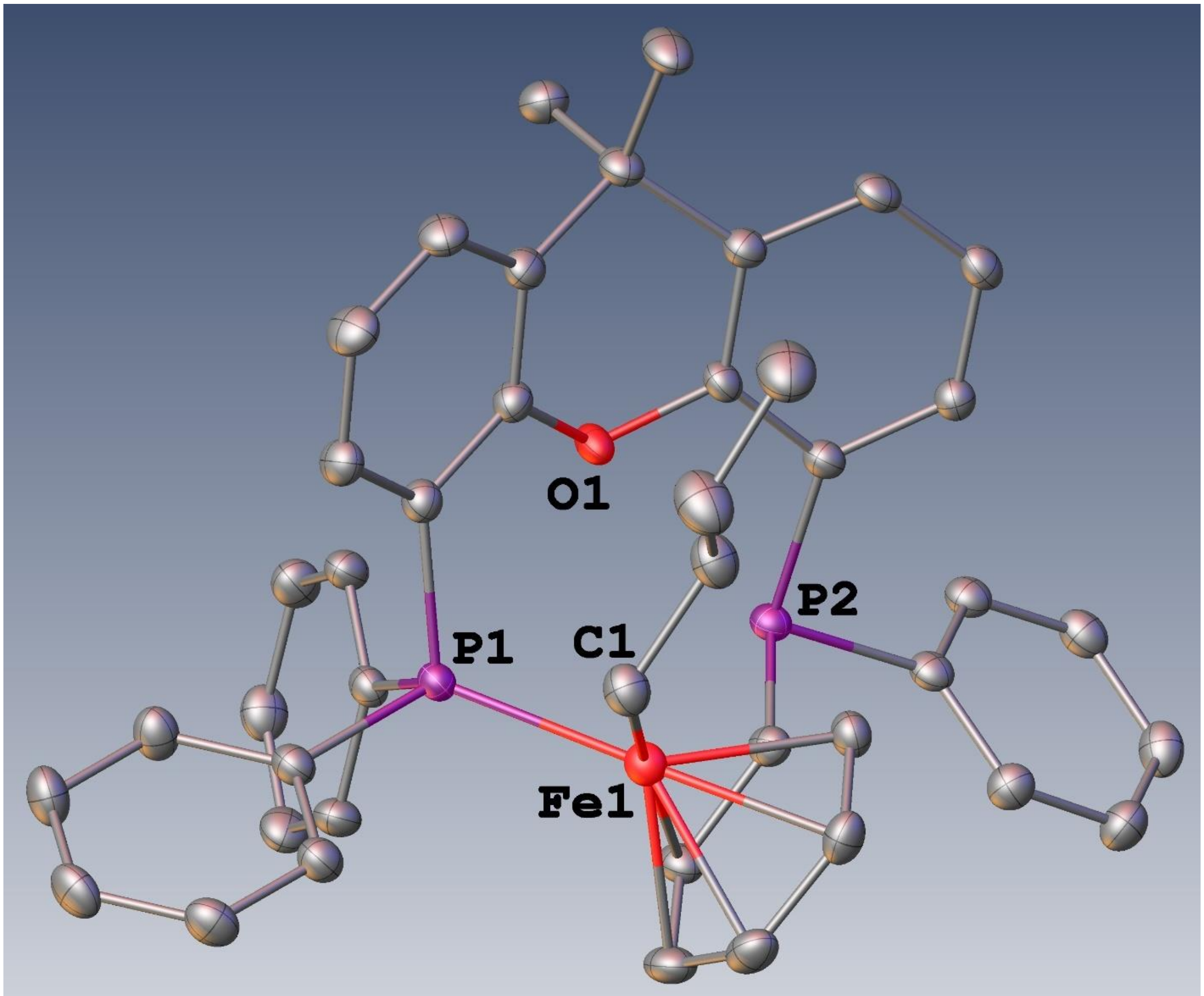
O1
P1
C1
P2
Fe1

Table 1. Crystal data and structure refinement for neivf21.

Identification code	neivf21	
Empirical formula	C47 H49 Fe O2 P2	
Formula weight	763.65	
Temperature	100.01(10) K	
Wavelength	1.54184 Å	
Crystal system	triclinic	
Space group	*P*-1	
Unit cell dimensions	*a* = 10.37360(10) Å	α = 76.1440(10)°
	b = 10.64430(10) Å	β = 80.7290(10)°
	c = 18.30550(10) Å	γ = 79.9080(10)°
Volume	1917.04(3) $Å^3$	
Z	2	
Density (calculated)	1.323 Mg/m^3	
Absorption coefficient	4.238 mm^{-1}	
F(000)	806	
Crystal color, morphology	orange, needle	
Crystal size	0.152 x 0.055 x 0.041 mm^3	
Theta range for data collection	2.506 to 78.029°	
Index ranges	$-11 \le h \le 12$, $-13 \le k \le 13$, $-23 \le l \le 23$	
Reflections collected	69036	
Independent reflections	8093 [*R*(int) = 0.0464]	
Observed reflections	7769	
Completeness to theta = 74.504°	99.9%	
Absorption correction	Multi-scan	
Max. and min. transmission	1.00000 and 0.76829	
Refinement method	Full-matrix least-squares on F^2	
Data / restraints / parameters	8093 / 0 / 492	
Goodness-of-fit on F^2	1.135	
Final *R* indices [*I*>2sigma(*I*)]	*R*1 = 0.0397, *wR*2 = 0.1108	
R indices (all data)	*R*1 = 0.0411, *wR*2 = 0.1118	
Largest diff. peak and hole	0.809 and -0.631 e.$Å^{-3}$	

Table 2. Atomic coordinates (x 10^4) and equivalent isotropic displacement parameters ($Å^2$x 10^3) for neivf21. U_{eq} is defined as one third of the trace of the orthogonalized U_{ij} tensor.

	x	y	z	U_{eq}
Fe1	2304(1)	7706(1)	1674(1)	19(1)
P1	2975(1)	5734(1)	2339(1)	16(1)
P2	619(1)	8607(1)	3401(1)	16(1)
O1	3284(1)	7200(1)	3458(1)	18(1)
C1	4176(2)	7767(2)	1106(1)	24(1)
C2	4922(2)	8673(2)	1361(1)	26(1)
C3	6316(2)	8745(2)	949(2)	38(1)
C4	7073(2)	9599(2)	1226(2)	40(1)
C5	4491(2)	5729(2)	2731(1)	19(1)
C6	5714(2)	5112(2)	2450(1)	22(1)
C7	6877(2)	5333(2)	2647(1)	27(1)
C8	6846(2)	6157(2)	3132(1)	24(1)
C9	4273(2)	9985(2)	3969(1)	21(1)
C10	3118(2)	10858(2)	3900(1)	21(1)
C11	2018(2)	10500(2)	3696(1)	19(1)
C12	2062(2)	9262(2)	3552(1)	18(1)
C13	5543(2)	7667(2)	3986(1)	21(1)
C14	6808(2)	8250(2)	3925(1)	29(1)
C15	5259(2)	6838(2)	4795(1)	26(1)
C16	4505(2)	6568(2)	3211(1)	18(1)
C17	5653(2)	6790(2)	3428(1)	20(1)
C18	4362(2)	8732(2)	3837(1)	19(1)
C19	3259(2)	8417(2)	3613(1)	17(1)
C20	3461(2)	4450(2)	1794(1)	19(1)
C21	3548(2)	4770(2)	1007(1)	22(1)
C22	3926(2)	3800(2)	593(1)	26(1)
C23	4205(2)	2504(2)	964(1)	27(1)
C24	4118(2)	2175(2)	1751(1)	28(1)
C25	3751(2)	3142(2)	2162(1)	25(1)
C26	1880(2)	4908(2)	3128(1)	18(1)
C27	781(2)	4503(2)	2944(1)	21(1)

C28	-91(2)	3863(2)	3510(1)	25(1)
C29	102(2)	3638(2)	4264(1)	26(1)
C30	1181(2)	4047(2)	4451(1)	25(1)
C31	2071(2)	4666(2)	3887(1)	21(1)
C32	-689(2)	10001(2)	3470(1)	18(1)
C33	-1328(2)	10732(2)	2856(1)	22(1)
C34	-2394(2)	11691(2)	2963(1)	25(1)
C35	-2825(2)	11955(2)	3675(1)	25(1)
C36	-2195(2)	11234(2)	4291(1)	23(1)
C37	-1153(2)	10253(2)	4189(1)	20(1)
C38	859(2)	8690(2)	2383(1)	18(1)
C39	268(2)	7794(2)	2123(1)	21(1)
C40	405(2)	7760(2)	1348(1)	25(1)
C41	1123(2)	8656(2)	817(1)	26(1)
C42	1686(2)	9579(2)	1062(1)	24(1)
C43	1578(2)	9585(2)	1840(1)	21(1)
O2	9510(2)	4610(2)	1212(1)	48(1)
C44	8455(3)	4798(2)	774(1)	38(1)
C45	8191(3)	3468(3)	752(2)	48(1)
C46	9571(3)	2666(2)	788(1)	37(1)
C47	10164(2)	3284(2)	1300(1)	32(1)

Table 3. Bond lengths [Å] and angles [°] for neivf21.

Fe(1)-P(1)	2.2164(5)	C(8)-H(8)	0.9300
Fe(1)-C(1)	2.053(2)	C(8)-C(17)	1.394(3)
Fe(1)-C(38)	2.1217(18)	C(9)-H(9)	0.9300
Fe(1)-C(39)	2.134(2)	C(9)-C(10)	1.385(3)
Fe(1)-C(40)	2.136(2)	C(9)-C(18)	1.397(3)
Fe(1)-C(41)	2.100(2)	C(10)-H(10)	0.9300
Fe(1)-C(42)	2.0859(19)	C(10)-C(11)	1.394(3)
Fe(1)-C(43)	2.0853(19)	C(11)-H(11)	0.9300
P(1)-C(5)	1.8299(19)	C(11)-C(12)	1.396(2)
P(1)-C(20)	1.8350(19)	C(12)-C(19)	1.403(3)
P(1)-C(26)	1.8331(19)	C(13)-C(14)	1.525(3)
P(2)-C(12)	1.8435(18)	C(13)-C(15)	1.544(3)
P(2)-C(32)	1.8405(19)	C(13)-C(17)	1.520(3)
P(2)-C(38)	1.8225(19)	C(13)-C(18)	1.527(3)
O(1)-C(16)	1.387(2)	C(14)-H(14A)	0.9600
O(1)-C(19)	1.385(2)	C(14)-H(14B)	0.9600
C(1)-H(1A)	0.9700	C(14)-H(14C)	0.9600
C(1)-H(1B)	0.9700	C(15)-H(15A)	0.9600
C(1)-C(2)	1.530(3)	C(15)-H(15B)	0.9600
C(2)-H(2A)	0.9700	C(15)-H(15C)	0.9600
C(2)-H(2B)	0.9700	C(16)-C(17)	1.388(3)
C(2)-C(3)	1.526(3)	C(18)-C(19)	1.392(3)
C(3)-H(3A)	0.9700	C(20)-C(21)	1.389(3)
C(3)-H(3B)	0.9700	C(20)-C(25)	1.397(3)
C(3)-C(4)	1.517(3)	C(21)-H(21)	0.9300
C(4)-H(4A)	0.9600	C(21)-C(22)	1.390(3)
C(4)-H(4B)	0.9600	C(22)-H(22)	0.9300
C(4)-H(4C)	0.9600	C(22)-C(23)	1.387(3)
C(5)-C(6)	1.401(3)	C(23)-H(23)	0.9300
C(5)-C(16)	1.398(3)	C(23)-C(24)	1.391(3)
C(6)-H(6)	0.9300	C(24)-H(24)	0.9300
C(6)-C(7)	1.386(3)	C(24)-C(25)	1.383(3)
C(7)-H(7)	0.9300	C(25)-H(25)	0.9300
C(7)-C(8)	1.383(3)	C(26)-C(27)	1.402(3)

C(26)-C(31)	1.392(3)	C(44)-C(45)	1.499(4)
C(27)-H(27)	0.9300	C(45)-H(45A)	0.9700
C(27)-C(28)	1.390(3)	C(45)-H(45B)	0.9700
C(28)-H(28)	0.9300	C(45)-C(46)	1.535(4)
C(28)-C(29)	1.385(3)	C(46)-H(46A)	0.9700
C(29)-H(29)	0.9300	C(46)-H(46B)	0.9700
C(29)-C(30)	1.387(3)	C(46)-C(47)	1.521(3)
C(30)-H(30)	0.9300	C(47)-H(47A)	0.9700
C(30)-C(31)	1.390(3)	C(47)-H(47B)	0.9700
C(31)-H(31)	0.9300	C(1)-Fe(1)-P(1)	89.37(6)
C(32)-C(33)	1.396(3)	C(1)-Fe(1)-C(38)	142.06(8)
C(32)-C(37)	1.399(3)	C(1)-Fe(1)-C(39)	172.24(8)
C(33)-H(33)	0.9300	C(1)-Fe(1)-C(40)	134.69(8)
C(33)-C(34)	1.391(3)	C(1)-Fe(1)-C(41)	102.24(8)
C(34)-H(34)	0.9300	C(1)-Fe(1)-C(42)	90.52(8)
C(34)-C(35)	1.386(3)	C(1)-Fe(1)-C(43)	106.95(8)
C(35)-H(35)	0.9300	C(38)-Fe(1)-P(1)	108.29(5)
C(35)-C(36)	1.391(3)	C(38)-Fe(1)-C(39)	39.11(7)
C(36)-H(36)	0.9300	C(38)-Fe(1)-C(40)	71.20(7)
C(36)-C(37)	1.388(3)	C(39)-Fe(1)-P(1)	97.10(6)
C(37)-H(37)	0.9300	C(39)-Fe(1)-C(40)	38.62(7)
C(38)-C(39)	1.424(3)	C(40)-Fe(1)-P(1)	110.05(6)
C(38)-C(43)	1.418(3)	C(41)-Fe(1)-P(1)	141.56(6)
C(39)-H(39)	0.92(3)	C(41)-Fe(1)-C(38)	84.73(7)
C(39)-C(40)	1.412(3)	C(41)-Fe(1)-C(39)	70.01(8)
C(40)-H(40)	0.94(3)	C(41)-Fe(1)-C(40)	38.82(8)
C(40)-C(41)	1.408(3)	C(42)-Fe(1)-P(1)	178.87(6)
C(41)-H(41)	0.99(3)	C(42)-Fe(1)-C(38)	71.18(7)
C(41)-C(42)	1.416(3)	C(42)-Fe(1)-C(39)	83.11(8)
C(42)-H(42)	0.91(3)	C(42)-Fe(1)-C(40)	70.79(8)
C(42)-C(43)	1.411(3)	C(42)-Fe(1)-C(41)	39.55(8)
C(43)-H(43)	0.97(2)	C(43)-Fe(1)-P(1)	139.49(6)
O(2)-C(44)	1.418(3)	C(43)-Fe(1)-C(38)	39.37(7)
O(2)-C(47)	1.439(3)	C(43)-Fe(1)-C(39)	70.70(7)
C(44)-H(44A)	0.9700	C(43)-Fe(1)-C(40)	84.71(8)
C(44)-H(44B)	0.9700	C(43)-Fe(1)-C(41)	71.97(8)

C(43)-Fe(1)-C(42)	39.55(8)	C(16)-C(5)-P(1)	119.81(14)
C(5)-P(1)-Fe(1)	110.61(6)	C(16)-C(5)-C(6)	117.09(17)
C(5)-P(1)-C(20)	102.38(8)	C(5)-C(6)-H(6)	119.6
C(5)-P(1)-C(26)	105.67(8)	C(7)-C(6)-C(5)	120.73(18)
C(20)-P(1)-Fe(1)	115.35(6)	C(7)-C(6)-H(6)	119.6
C(26)-P(1)-Fe(1)	120.80(6)	C(6)-C(7)-H(7)	119.9
C(26)-P(1)-C(20)	99.96(8)	C(8)-C(7)-C(6)	120.29(18)
C(32)-P(2)-C(12)	101.01(8)	C(8)-C(7)-H(7)	119.9
C(38)-P(2)-C(12)	103.27(8)	C(7)-C(8)-H(8)	119.5
C(38)-P(2)-C(32)	101.13(8)	C(7)-C(8)-C(17)	121.09(18)
C(19)-O(1)-C(16)	116.86(14)	C(17)-C(8)-H(8)	119.5
Fe(1)-C(1)-H(1A)	109.1	C(10)-C(9)-H(9)	119.6
Fe(1)-C(1)-H(1B)	109.1	C(10)-C(9)-C(18)	120.84(17)
H(1A)-C(1)-H(1B)	107.8	C(18)-C(9)-H(9)	119.6
C(2)-C(1)-Fe(1)	112.46(13)	C(9)-C(10)-H(10)	119.8
C(2)-C(1)-H(1A)	109.1	C(9)-C(10)-C(11)	120.48(17)
C(2)-C(1)-H(1B)	109.1	C(11)-C(10)-H(10)	119.8
C(1)-C(2)-H(2A)	108.8	C(10)-C(11)-H(11)	119.6
C(1)-C(2)-H(2B)	108.8	C(10)-C(11)-C(12)	120.74(17)
H(2A)-C(2)-H(2B)	107.7	C(12)-C(11)-H(11)	119.6
C(3)-C(2)-C(1)	113.91(17)	C(11)-C(12)-P(2)	124.45(14)
C(3)-C(2)-H(2A)	108.8	C(11)-C(12)-C(19)	117.02(16)
C(3)-C(2)-H(2B)	108.8	C(19)-C(12)-P(2)	118.12(13)
C(2)-C(3)-H(3A)	108.8	C(14)-C(13)-C(15)	109.13(17)
C(2)-C(3)-H(3B)	108.8	C(14)-C(13)-C(18)	111.53(16)
H(3A)-C(3)-H(3B)	107.7	C(17)-C(13)-C(14)	111.83(16)
C(4)-C(3)-C(2)	113.7(2)	C(17)-C(13)-C(15)	108.13(15)
C(4)-C(3)-H(3A)	108.8	C(17)-C(13)-C(18)	107.98(15)
C(4)-C(3)-H(3B)	108.8	C(18)-C(13)-C(15)	108.12(16)
C(3)-C(4)-H(4A)	109.5	C(13)-C(14)-H(14A)	109.5
C(3)-C(4)-H(4B)	109.5	C(13)-C(14)-H(14B)	109.5
C(3)-C(4)-H(4C)	109.5	C(13)-C(14)-H(14C)	109.5
H(4A)-C(4)-H(4B)	109.5	H(14A)-C(14)-H(14B)	109.5
H(4A)-C(4)-H(4C)	109.5	H(14A)-C(14)-H(14C)	109.5
H(4B)-C(4)-H(4C)	109.5	H(14B)-C(14)-H(14C)	109.5
C(6)-C(5)-P(1)	121.82(14)	C(13)-C(15)-H(15A)	109.5

C(13)-C(15)-H(15B)	109.5	C(31)-C(26)-P(1)	124.30(14)
C(13)-C(15)-H(15C)	109.5	C(31)-C(26)-C(27)	118.68(17)
H(15A)-C(15)-H(15B)	109.5	C(26)-C(27)-H(27)	119.8
H(15A)-C(15)-H(15C)	109.5	C(28)-C(27)-C(26)	120.45(17)
H(15B)-C(15)-H(15C)	109.5	C(28)-C(27)-H(27)	119.8
O(1)-C(16)-C(5)	115.78(16)	C(27)-C(28)-H(28)	119.8
O(1)-C(16)-C(17)	120.79(16)	C(29)-C(28)-C(27)	120.37(18)
C(17)-C(16)-C(5)	123.42(17)	C(29)-C(28)-H(28)	119.8
C(8)-C(17)-C(13)	123.86(17)	C(28)-C(29)-H(29)	120.3
C(16)-C(17)-C(8)	117.36(17)	C(28)-C(29)-C(30)	119.48(18)
C(16)-C(17)-C(13)	118.75(17)	C(30)-C(29)-H(29)	120.3
C(9)-C(18)-C(13)	123.83(16)	C(29)-C(30)-H(30)	119.8
C(19)-C(18)-C(9)	117.32(17)	C(29)-C(30)-C(31)	120.49(18)
C(19)-C(18)-C(13)	118.64(16)	C(31)-C(30)-H(30)	119.8
O(1)-C(19)-C(12)	115.94(15)	C(26)-C(31)-H(31)	119.7
O(1)-C(19)-C(18)	120.51(16)	C(30)-C(31)-C(26)	120.52(18)
C(18)-C(19)-C(12)	123.52(16)	C(30)-C(31)-H(31)	119.7
C(21)-C(20)-P(1)	120.27(14)	C(33)-C(32)-P(2)	123.29(14)
C(21)-C(20)-C(25)	119.17(17)	C(33)-C(32)-C(37)	118.40(17)
C(25)-C(20)-P(1)	120.56(14)	C(37)-C(32)-P(2)	117.99(14)
C(20)-C(21)-H(21)	119.8	C(32)-C(33)-H(33)	119.8
C(20)-C(21)-C(22)	120.36(18)	C(34)-C(33)-C(32)	120.31(18)
C(22)-C(21)-H(21)	119.8	C(34)-C(33)-H(33)	119.8
C(21)-C(22)-H(22)	120.0	C(33)-C(34)-H(34)	119.6
C(23)-C(22)-C(21)	120.07(19)	C(35)-C(34)-C(33)	120.78(19)
C(23)-C(22)-H(22)	120.0	C(35)-C(34)-H(34)	119.6
C(22)-C(23)-H(23)	120.0	C(34)-C(35)-H(35)	120.3
C(22)-C(23)-C(24)	119.97(18)	C(34)-C(35)-C(36)	119.43(18)
C(24)-C(23)-H(23)	120.0	C(36)-C(35)-H(35)	120.3
C(23)-C(24)-H(24)	120.1	C(35)-C(36)-H(36)	120.1
C(25)-C(24)-C(23)	119.87(19)	C(37)-C(36)-C(35)	119.87(18)
C(25)-C(24)-H(24)	120.1	C(37)-C(36)-H(36)	120.1
C(20)-C(25)-H(25)	119.7	C(32)-C(37)-H(37)	119.4
C(24)-C(25)-C(20)	120.56(19)	C(36)-C(37)-C(32)	121.17(18)
C(24)-C(25)-H(25)	119.7	C(36)-C(37)-H(37)	119.4
C(27)-C(26)-P(1)	117.02(14)	P(2)-C(38)-Fe(1)	132.59(10)

C(39)-C(38)-Fe(1)	70.92(11)	C(38)-C(43)-Fe(1)	71.70(10)
C(39)-C(38)-P(2)	117.02(14)	C(38)-C(43)-H(43)	120.0(14)
C(43)-C(38)-Fe(1)	68.93(10)	C(42)-C(43)-Fe(1)	70.25(11)
C(43)-C(38)-P(2)	124.56(14)	C(42)-C(43)-C(38)	119.91(17)
C(43)-C(38)-C(39)	118.42(17)	C(42)-C(43)-H(43)	120.1(14)
Fe(1)-C(39)-H(39)	127.9(17)	C(44)-O(2)-C(47)	110.02(18)
C(38)-C(39)-Fe(1)	69.97(11)	O(2)-C(44)-H(44A)	110.3
C(38)-C(39)-H(39)	119.1(16)	O(2)-C(44)-H(44B)	110.3
C(40)-C(39)-Fe(1)	70.75(11)	O(2)-C(44)-C(45)	107.0(2)
C(40)-C(39)-C(38)	121.81(18)	H(44A)-C(44)-H(44B)	108.6
C(40)-C(39)-H(39)	118.8(16)	C(45)-C(44)-H(44A)	110.3
Fe(1)-C(40)-H(40)	128.8(15)	C(45)-C(44)-H(44B)	110.3
C(39)-C(40)-Fe(1)	70.64(11)	C(44)-C(45)-H(45A)	111.4
C(39)-C(40)-H(40)	119.1(15)	C(44)-C(45)-H(45B)	111.4
C(41)-C(40)-Fe(1)	69.21(12)	C(44)-C(45)-C(46)	101.9(2)
C(41)-C(40)-C(39)	118.96(18)	H(45A)-C(45)-H(45B)	109.3
C(41)-C(40)-H(40)	121.8(15)	C(46)-C(45)-H(45A)	111.4
Fe(1)-C(41)-H(41)	129.4(15)	C(46)-C(45)-H(45B)	111.4
C(40)-C(41)-Fe(1)	71.98(11)	C(45)-C(46)-H(46A)	111.3
C(40)-C(41)-H(41)	120.4(15)	C(45)-C(46)-H(46B)	111.3
C(40)-C(41)-C(42)	120.01(18)	H(46A)-C(46)-H(46B)	109.2
C(42)-C(41)-Fe(1)	69.71(11)	C(47)-C(46)-C(45)	102.28(19)
C(42)-C(41)-H(41)	119.6(15)	C(47)-C(46)-H(46A)	111.3
Fe(1)-C(42)-H(42)	125.7(17)	C(47)-C(46)-H(46B)	111.3
C(41)-C(42)-Fe(1)	70.75(11)	O(2)-C(47)-C(46)	106.07(18)
C(41)-C(42)-H(42)	121.2(17)	O(2)-C(47)-H(47A)	110.5
C(43)-C(42)-Fe(1)	70.20(11)	O(2)-C(47)-H(47B)	110.5
C(43)-C(42)-C(41)	120.83(18)	C(46)-C(47)-H(47A)	110.5
C(43)-C(42)-H(42)	117.5(17)	C(46)-C(47)-H(47B)	110.5
Fe(1)-C(43)-H(43)	130.3(14)	H(47A)-C(47)-H(47B)	108.7

Table 4. Anisotropic displacement parameters ($Å^2$x 10^3) for neivf21. The anisotropic displacement factor exponent takes the form: $-2\pi^2[h^2 a^{*2}U_{11} + ... + 2 h k a^* b^* U_{12}]$

	U_{11}	U_{22}	U_{33}	U_{23}	U_{13}	U_{12}
Fe1	19(1)	19(1)	21(1)	-6(1)	-2(1)	-3(1)
P1	16(1)	15(1)	18(1)	-5(1)	-3(1)	-3(1)
P2	14(1)	18(1)	19(1)	-5(1)	-3(1)	-4(1)
O1	14(1)	16(1)	27(1)	-9(1)	-3(1)	-2(1)
C1	24(1)	23(1)	26(1)	-7(1)	1(1)	-4(1)
C2	28(1)	23(1)	27(1)	-6(1)	2(1)	-6(1)
C3	27(1)	36(1)	51(1)	-16(1)	3(1)	-9(1)
C4	28(1)	36(1)	59(2)	-11(1)	-7(1)	-7(1)
C5	17(1)	17(1)	22(1)	-4(1)	-2(1)	-2(1)
C6	21(1)	22(1)	25(1)	-9(1)	-4(1)	1(1)
C7	17(1)	30(1)	32(1)	-10(1)	-3(1)	2(1)
C8	16(1)	27(1)	30(1)	-7(1)	-6(1)	-2(1)
C9	21(1)	22(1)	24(1)	-6(1)	-5(1)	-9(1)
C10	24(1)	18(1)	23(1)	-6(1)	-3(1)	-7(1)
C11	19(1)	17(1)	20(1)	-4(1)	-3(1)	-3(1)
C12	18(1)	19(1)	18(1)	-6(1)	-3(1)	-4(1)
C13	17(1)	22(1)	28(1)	-7(1)	-6(1)	-5(1)
C14	19(1)	27(1)	48(1)	-13(1)	-9(1)	-5(1)
C15	26(1)	26(1)	26(1)	-6(1)	-9(1)	-2(1)
C16	16(1)	17(1)	21(1)	-4(1)	-3(1)	-2(1)
C17	18(1)	18(1)	23(1)	-3(1)	-4(1)	-5(1)
C18	18(1)	19(1)	21(1)	-4(1)	-4(1)	-5(1)
C19	18(1)	15(1)	21(1)	-5(1)	-2(1)	-5(1)
C20	19(1)	20(1)	22(1)	-7(1)	-3(1)	-4(1)
C21	24(1)	21(1)	23(1)	-6(1)	-4(1)	-5(1)
C22	30(1)	30(1)	22(1)	-10(1)	-2(1)	-7(1)
C23	29(1)	26(1)	30(1)	-16(1)	2(1)	-5(1)
C24	34(1)	19(1)	32(1)	-9(1)	1(1)	-4(1)
C25	32(1)	21(1)	22(1)	-6(1)	-1(1)	-4(1)
C26	18(1)	16(1)	21(1)	-7(1)	-3(1)	-2(1)
C27	22(1)	23(1)	22(1)	-6(1)	-7(1)	-5(1)

C28	20(1)	23(1)	33(1)	-5(1)	-5(1)	-8(1)
C29	24(1)	22(1)	29(1)	-3(1)	2(1)	-6(1)
C30	31(1)	24(1)	21(1)	-3(1)	-5(1)	-6(1)
C31	24(1)	20(1)	22(1)	-4(1)	-7(1)	-6(1)
C32	15(1)	20(1)	22(1)	-6(1)	-2(1)	-6(1)
C33	20(1)	23(1)	21(1)	-5(1)	-2(1)	-2(1)
C34	24(1)	24(1)	26(1)	-2(1)	-5(1)	0(1)
C35	19(1)	21(1)	32(1)	-7(1)	2(1)	-2(1)
C36	22(1)	24(1)	25(1)	-9(1)	1(1)	-7(1)
C37	17(1)	22(1)	23(1)	-7(1)	-3(1)	-6(1)
C38	15(1)	20(1)	20(1)	-7(1)	-3(1)	0(1)
C39	17(1)	22(1)	26(1)	-9(1)	-4(1)	-3(1)
C40	22(1)	30(1)	28(1)	-12(1)	-10(1)	-1(1)
C41	28(1)	30(1)	20(1)	-7(1)	-8(1)	3(1)
C42	26(1)	19(1)	21(1)	-1(1)	-2(1)	1(1)
C43	22(1)	17(1)	24(1)	-6(1)	-3(1)	-2(1)
O2	47(1)	44(1)	63(1)	-28(1)	-21(1)	2(1)
C44	38(1)	37(1)	38(1)	-12(1)	-2(1)	-1(1)
C45	54(2)	44(1)	52(2)	-12(1)	-28(1)	-2(1)
C46	50(2)	30(1)	33(1)	-10(1)	-15(1)	-1(1)
C47	35(1)	34(1)	29(1)	-7(1)	-7(1)	-7(1)

Table 5. Hydrogen coordinates (x 10^4) and isotropic displacement parameters (Å^2 x 10^3) for neivf21.

	x	y	z	U(eq)
H1A	4665	6891	1190	29
H1B	4122	8064	566	29
H2A	4424	9545	1280	31
H2B	4971	8372	1901	31
H3A	6803	7869	1011	45
H3B	6265	9083	411	45
H4A	7969	9522	988	60
H4B	7061	9322	1766	60
H4C	6668	10493	1101	60
H6	5745	4549	2128	27
H7	7683	4925	2452	32
H8	7633	6292	3263	29
H9	5000	10237	4106	25
H10	3075	11689	3990	25
H11	1245	11093	3656	22
H14A	6707	8785	4291	44
H14B	6985	8774	3424	44
H14C	7530	7558	4022	44
H15A	5957	6118	4885	38
H15B	4437	6509	4845	38
H15C	5207	7369	5158	38
H21	3353	5637	757	27
H22	3992	4021	66	32
H23	4449	1855	686	32
H24	4307	1306	2000	33
H25	3695	2920	2688	30
H27	634	4663	2439	26
H28	-809	3584	3381	30
H29	-487	3218	4642	31
H30	1310	3905	4957	30

H31	2800	4921	4018	25
H33	-1038	10577	2374	26
H34	-2824	12160	2552	30
H35	-3528	12609	3740	29
H36	-2472	11409	4770	27
H37	-757	9754	4607	24
H39	-140(30)	7160(30)	2472(15)	31(7)
H40	70(20)	7110(20)	1205(14)	25(6)
H41	1260(30)	8630(20)	271(15)	30(6)
H42	2220(30)	10110(30)	735(16)	35(7)
H43	2000(20)	10200(20)	2003(13)	22(6)
H44A	8695	5288	264	46
H44B	7676	5281	1002	46
H45A	7849	3479	287	57
H45B	7575	3133	1183	57
H46A	9519	1749	1007	44
H46B	10078	2752	289	44
H47A	10017	2817	1824	39
H47B	11107	3267	1151	39

Table 6. Torsion angles [°] for neivf21.

Fe1-P1-C5-C6	-108.15(15)	C5-P1-C26-C31	14.99(18)
Fe1-P1-C5-C16	58.52(16)	C5-C6-C7-C8	0.7(3)
Fe1-P1-C20-C21	7.76(18)	C5-C16-C17-C8	1.5(3)
Fe1-P1-C20-C25	-172.33(14)	C5-C16-C17-C13	-176.76(17)
Fe1-P1-C26-C27	68.49(15)	C6-C5-C16-O1	177.76(16)
Fe1-P1-C26-C31	-111.37(15)	C6-C5-C16-C17	-1.4(3)
Fe1-C1-C2-C3	-179.79(16)	C6-C7-C8-C17	-0.5(3)
Fe1-C38-C39-C40	-50.27(17)	C7-C8-C17-C13	177.64(18)
Fe1-C38-C43-C42	53.06(16)	C7-C8-C17-C16	-0.6(3)
Fe1-C39-C40-C41	-51.35(17)	C9-C10-C11-C12	-0.5(3)
Fe1-C40-C41-C42	-52.48(17)	C9-C18-C19-O1	178.79(16)
Fe1-C41-C42-C43	-51.21(17)	C9-C18-C19-C12	-3.3(3)
Fe1-C42-C43-C38	-53.73(16)	C10-C9-C18-C13	-172.94(18)
P1-C5-C6-C7	167.20(16)	C10-C9-C18-C19	1.8(3)
P1-C5-C16-O1	10.5(2)	C10-C11-C12-P2	171.72(14)
P1-C5-C16-C17	-168.64(15)	C10-C11-C12-C19	-0.8(3)
P1-C20-C21-C22	179.53(15)	C11-C12-C19-O1	-179.20(15)
P1-C20-C25-C24	-179.97(16)	C11-C12-C19-C18	2.8(3)
P1-C26-C27-C28	179.61(15)	C12-P2-C32-C33	112.41(16)
P1-C26-C31-C30	179.22(15)	C12-P2-C32-C37	-74.25(15)
P2-C12-C19-O1	7.8(2)	C12-P2-C38-Fe1	66.06(14)
P2-C12-C19-C18	-170.24(15)	C12-P2-C38-C39	154.37(14)
P2-C32-C33-C34	173.70(15)	C12-P2-C38-C43	-26.24(18)
P2-C32-C37-C36	-175.81(14)	C13-C18-C19-O1	-6.2(3)
P2-C38-C39-Fe1	-128.86(12)	C13-C18-C19-C12	171.76(17)
P2-C38-C39-C40	-179.13(15)	C14-C13-C17-C8	24.8(3)
P2-C38-C43-Fe1	127.97(14)	C14-C13-C17-C16	-156.99(18)
P2-C38-C43-C42	-178.97(14)	C14-C13-C18-C9	-27.1(3)
O1-C16-C17-C8	-177.53(17)	C14-C13-C18-C19	158.20(18)
O1-C16-C17-C13	4.2(3)	C15-C13-C17-C8	-95.3(2)
C1-C2-C3-C4	-177.5(2)	C15-C13-C17-C16	82.8(2)
C5-P1-C20-C21	-112.43(16)	C15-C13-C18-C9	92.9(2)
C5-P1-C20-C25	67.48(18)	C15-C13-C18-C19	-81.8(2)
C5-P1-C26-C27	-165.15(14)	C16-O1-C19-C12	154.47(16)

C16-O1-C19-C18	-27.4(2)	C33-C34-C35-C36	-1.2(3)
C16-C5-C6-C7	0.2(3)	C34-C35-C36-C37	-0.5(3)
C17-C13-C18-C9	-150.37(18)	C35-C36-C37-C32	2.2(3)
C17-C13-C18-C19	35.0(2)	C37-C32-C33-C34	0.4(3)
C18-C9-C10-C11	0.0(3)	C38-P2-C12-C11	105.83(16)
C18-C13-C17-C8	147.90(18)	C38-P2-C12-C19	-81.72(16)
C18-C13-C17-C16	-33.9(2)	C38-P2-C32-C33	6.35(17)
C19-O1-C16-C5	-150.51(16)	C38-P2-C32-C37	179.68(14)
C19-O1-C16-C17	28.6(2)	C38-C39-C40-Fe1	49.94(17)
C20-P1-C5-C6	15.29(18)	C38-C39-C40-C41	-1.4(3)
C20-P1-C5-C16	-178.04(15)	C39-C38-C43-Fe1	-52.64(15)
C20-P1-C26-C27	-59.17(16)	C39-C38-C43-C42	0.4(3)
C20-P1-C26-C31	120.98(16)	C39-C40-C41-Fe1	52.02(17)
C20-C21-C22-C23	0.7(3)	C39-C40-C41-C42	-0.5(3)
C21-C20-C25-C24	-0.1(3)	C40-C41-C42-Fe1	53.52(17)
C21-C22-C23-C24	-0.6(3)	C40-C41-C42-C43	2.3(3)
C22-C23-C24-C25	0.2(3)	C41-C42-C43-Fe1	51.46(17)
C23-C24-C25-C20	0.2(3)	C41-C42-C43-C38	-2.3(3)
C25-C20-C21-C22	-0.4(3)	C43-C38-C39-Fe1	51.71(15)
C26-P1-C5-C6	119.50(16)	C43-C38-C39-C40	1.4(3)
C26-P1-C5-C16	-73.83(16)	O2-C44-C45-C46	-31.5(3)
C26-P1-C20-C21	138.95(16)	C44-O2-C47-C46	7.7(3)
C26-P1-C20-C25	-41.14(18)	C44-C45-C46-C47	34.8(3)
C26-C27-C28-C29	1.2(3)	C45-C46-C47-O2	-26.8(3)
C27-C26-C31-C30	-0.6(3)	C47-O2-C44-C45	15.4(3)
C27-C28-C29-C30	-0.6(3)		
C28-C29-C30-C31	-0.6(3)		
C29-C30-C31-C26	1.2(3)		
C31-C26-C27-C28	-0.5(3)		
C32-P2-C12-C11	1.48(18)		
C32-P2-C12-C19	173.93(14)		
C32-P2-C38-Fe1	170.33(12)		
C32-P2-C38-C39	-101.37(15)		
C32-P2-C38-C43	78.02(17)		
C32-C33-C34-C35	1.3(3)		
C33-C32-C37-C36	-2.1(3)		

3.5 Fe(0)-DPEphos (5-Et)

CRYSTAL STRUCTURE REPORT

$C_{38}H_{32}FeOP_2$

Report prepared for:

M. C. Aguilera, Prof. M. Neidig

June 22, 2022

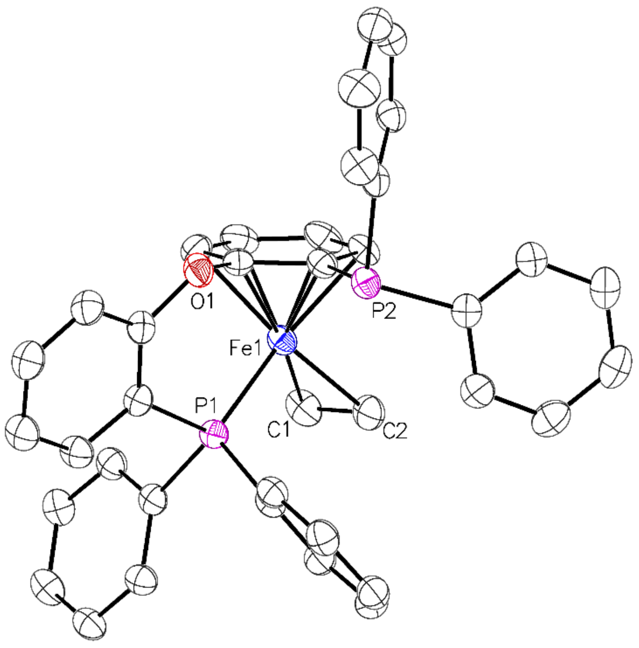

William W. Brennessel

X-ray Crystallographic Facility

Department of Chemistry, University of Rochester

120 Trustee Road

Rochester, NY 14627

Data collection

A crystal (0.123 x 0.042 x 0.031 mm^3) was placed onto a thin glass optical fiber or a nylon loop and mounted on a Rigaku XtaLAB Synergy-S Dualflex diffractometer equipped with a HyPix-6000HE HPC area detector for data collection at 100.00(10) K. A preliminary set of cell constants and an orientation matrix were calculated from a small sampling of reflections.[1] A short pre-experiment was run, from which an optimal data collection strategy was determined. The full data collection was carried out using a PhotonJet (Cu) X-ray source with frame times of 8.00 and 32.00 seconds and a detector distance of 34.0 mm. Series of frames were collected in 0.50º steps in ω at different 2θ, κ, and ϕ settings. After the intensity data were corrected for absorption, the final cell constants were calculated from the xyz centroids of 14362 strong reflections from the actual data collection after integration.[1] See Table 1 for additional crystal and refinement information.

Structure solution and refinement

The structure was solved using SHELXT[2] and refined using SHELXL.[3] The space group $P2_1/c$ was determined based on systematic absences. Most or all non-hydrogen atoms were assigned from the solution. Full-matrix least squares / difference Fourier cycles were performed which located any remaining non-hydrogen atoms. All non-hydrogen atoms were refined with anisotropic displacement parameters. The hydrogen atoms on the metal-coordinating carbon atoms were found from the difference Fourier map and refined freely to better approximate their true positions. All other hydrogen atoms were placed in ideal positions and refined as riding atoms with relative isotropic displacement parameters. The final full matrix least squares refinement converged to $R1 = 0.0413$ (F^2, $I > 2\sigma(I)$) and $wR2 = 0.1146$ (F^2, all data).

Structure description

The structure is the one suggested. The asymmetric unit contains one iron molecule in a general position.

Structure manipulation and figure generation were performed using Olex2.[4] Unless noted otherwise all structural diagrams containing anisotropic displacement ellipsoids are drawn at the 50 % probability level.

Data collection, structure solution, and structure refinement were conducted at the X-ray Crystallographic Facility, B04 Hutchison Hall, Department of Chemistry, University of Rochester. The instrument was purchased with funding from NSF MRI program grant CHE-1725028. All publications arising from this report MUST either 1) include William W. Brennessel as a coauthor or 2) acknowledge William W. Brennessel and the X-ray Crystallographic Facility of the Department of Chemistry at the University of Rochester.

[1] *CrysAlisPro*, version 171.42.57a; Rigaku Corporation: Oxford, UK, 2022.

[2] Sheldrick, G. M. *SHELXT*, version 2018/2; *Acta. Crystallogr.* **2015**, *A71*, 3-8.

[3] Sheldrick, G. M. *SHELXL*, version 2019/2; *Acta. Crystallogr.* **2015**, *C71*, 3-8.

[4] Dolomanov, O. V.; Bourhis, L. J.; Gildea, R. J.; Howard, J. A. K.; Puschmann, H. *Olex2*, version 1.5; *J. Appl. Cryst.* **2009**, *42*, 339-341.

Some equations of interest:

$$R_{int} = \Sigma |F_o^2 - \langle F_o^2 \rangle| / \Sigma|F_o^2|$$

$$R1 = \Sigma||F_o|-|F_c|| / \Sigma|F_o|$$

$$wR2 = [\Sigma[w(F_o^2-F_c^2)^2] / \Sigma [w(F_o^2)^2]]^{1/2}$$

where $w = 1 / [\sigma^2 (F_o^2) + (aP)^2 + bP]$ and

$$P = 1/3 \max (0, F_o^2) + 2/3\, F_c^2$$

$$\mathrm{GOF} = S = [\Sigma[w(F_o^2-F_c^2)^2] / (m-n)]^{1/2}$$

where m = number of reflections and n = number of parameters

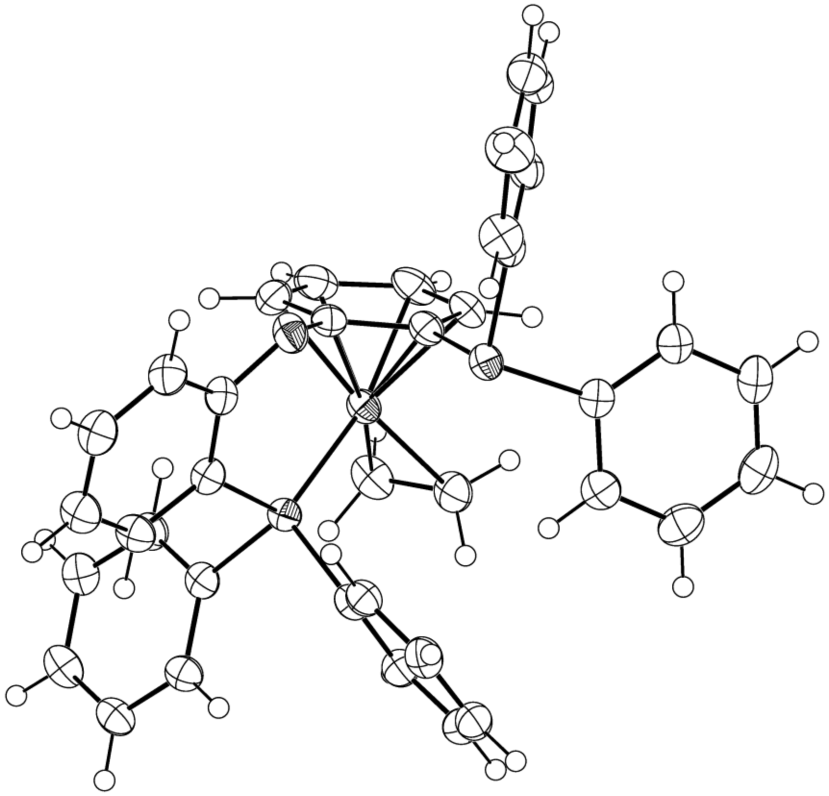

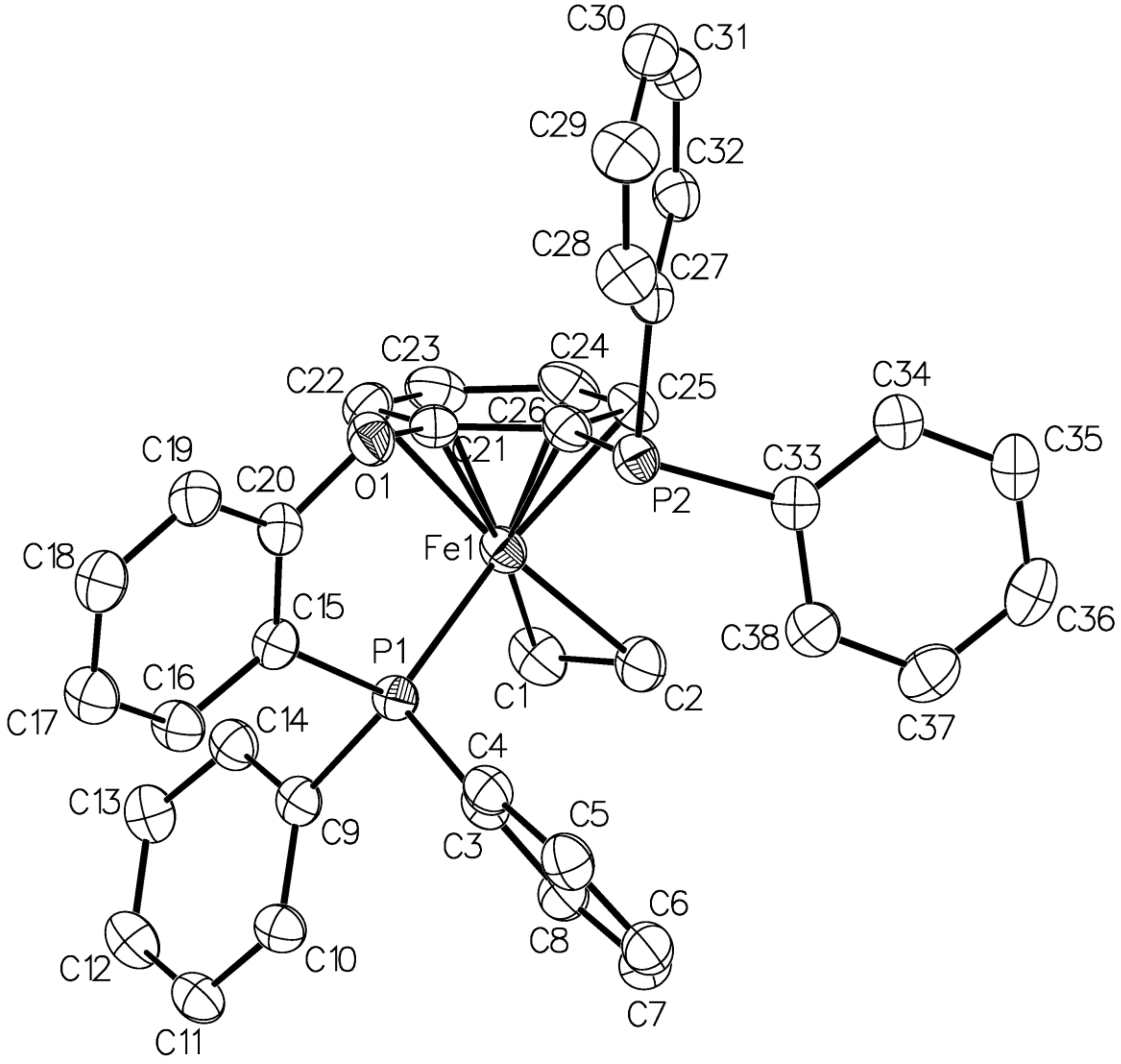
C30
C31
C29
C32
C28
C27
C23
C24
C22
C25
C26
C21
C34
C35
C19
C20
O1
P2
C33
Fe1
C18
C15
C38
C36
P1
C16
C1
C2
C37
C17
C14
C4
C13
C9
C5
C3
C6
C8
C12
C10
C7
C11

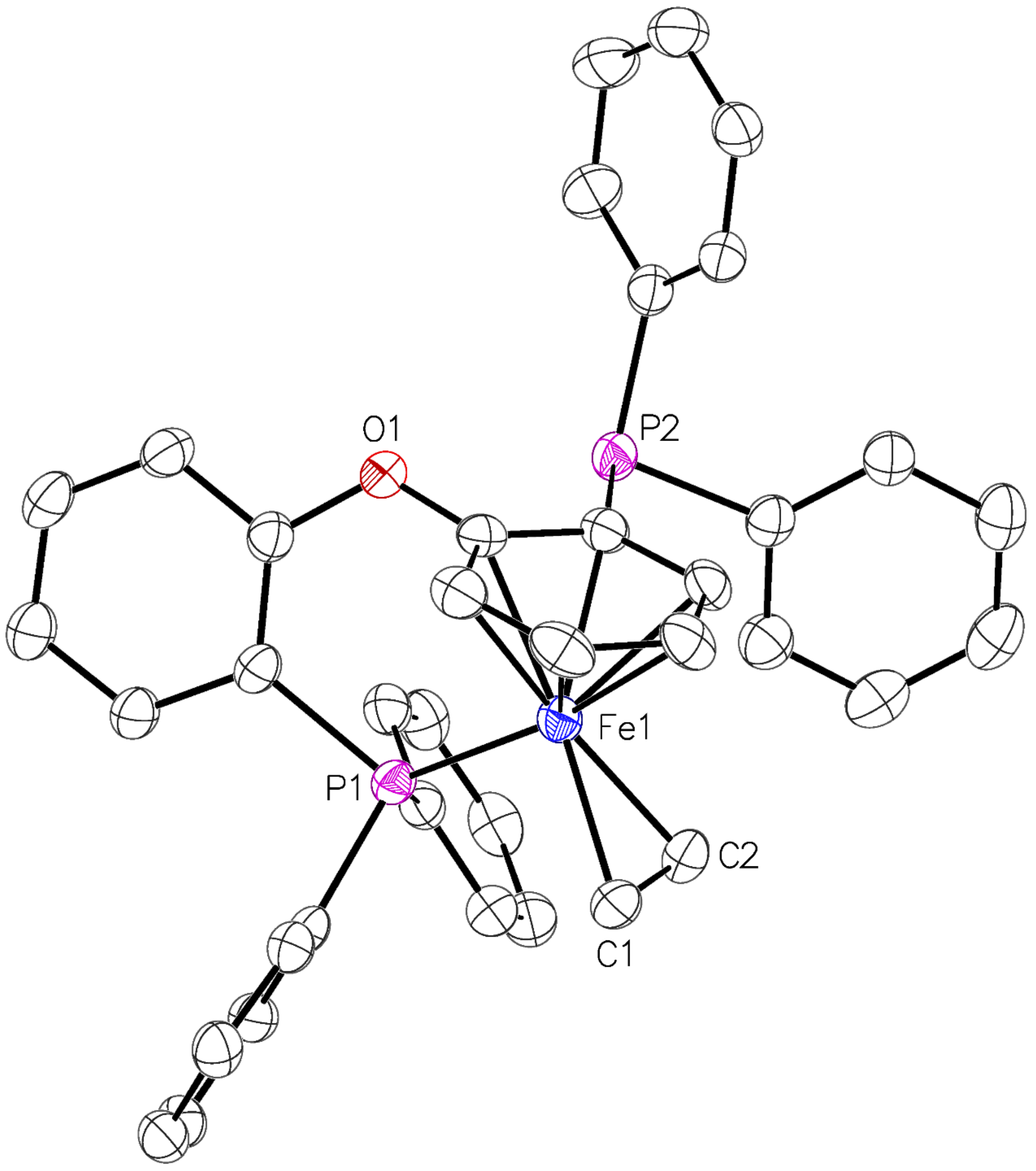
O1
P2
Fe1
P1
C2
C1

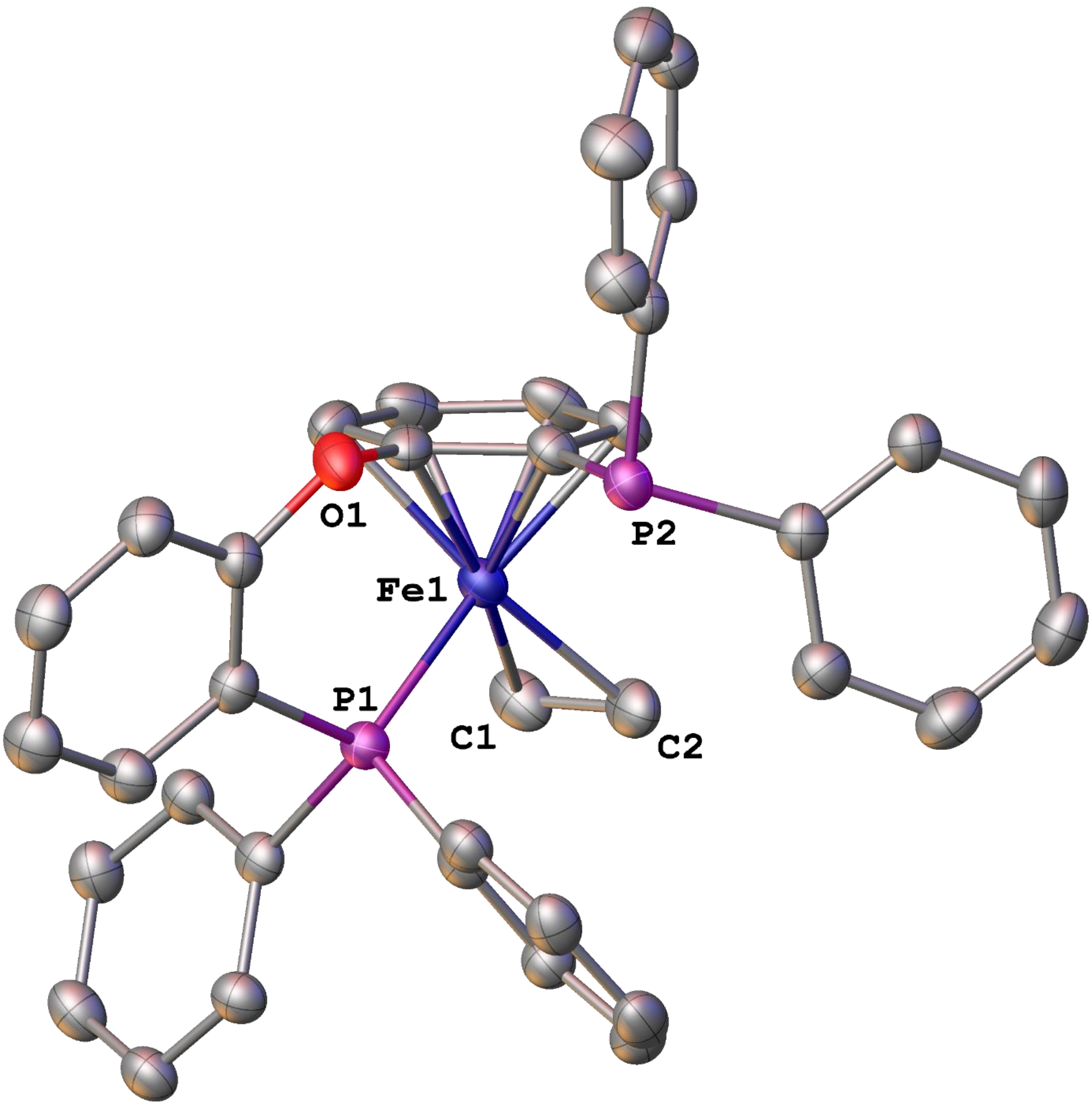
O1
P2
Fe1
P1
C1
C2

Table 1. Crystal data and structure refinement for neimca24.

Identification code	neimca24	
Empirical formula	C38 H32 Fe O P2	
Formula weight	622.42	
Temperature	100.00(10) K	
Wavelength	1.54184 Å	
Crystal system	monoclinic	
Space group	$P2_1/c$	
Unit cell dimensions	a = 13.1724(2) Å	α = 90°
	b = 14.6342(2) Å	β = 110.325(2)°
	c = 16.5868(2) Å	γ = 90°
Volume	2998.32(8) $Å^3$	
Z	4	
Density (calculated)	1.379 Mg/m^3	
Absorption coefficient	5.270 mm^{-1}	
$F(000)$	1296	
Crystal color, morphology	red-orange, needle	
Crystal size	0.123 x 0.042 x 0.031 mm^3	
Theta range for data collection	3.578 to 80.174°	
Index ranges	$-16 \le h \le 16$, $-17 \le k \le 18$, $-21 \le l \le 19$	
Reflections collected	30749	
Independent reflections	6326 [R(int) = 0.0384]	
Observed reflections	5603	
Completeness to theta = 74.504°	99.1%	
Absorption correction	Multi-scan	
Max. and min. transmission	1.00000 and 0.73221	
Refinement method	Full-matrix least-squares on F^2	
Data / restraints / parameters	6326 / 0 / 411	
Goodness-of-fit on F^2	1.081	
Final R indices [I>2sigma(I)]	$R1$ = 0.0413, $wR2$ = 0.1111	
R indices (all data)	$R1$ = 0.0468, $wR2$ = 0.1146	
Largest diff. peak and hole	0.525 and -0.536 e.$Å^{-3}$	

Table 2. Atomic coordinates (x 10^4) and equivalent isotropic displacement parameters ($Å^2$x 10^3) for neimca24. U_{eq} is defined as one third of the trace of the orthogonalized U_{ij} tensor.

	x	y	z	U_{eq}
Fe1	3557(1)	6103(1)	5935(1)	25(1)
P1	4613(1)	7055(1)	6866(1)	23(1)
P2	1450(1)	6445(1)	6819(1)	26(1)
O1	3508(1)	5633(1)	7806(1)	32(1)
C1	4081(2)	6538(2)	4971(2)	36(1)
C2	3106(2)	6995(2)	4912(2)	35(1)
C3	4028(2)	8179(2)	6928(1)	27(1)
C4	3597(2)	8373(2)	7573(1)	29(1)
C5	3039(2)	9178(2)	7554(2)	34(1)
C6	2882(2)	9800(2)	6891(2)	36(1)
C7	3308(2)	9617(2)	6254(2)	35(1)
C8	3888(2)	8819(2)	6276(2)	31(1)
C9	5951(2)	7312(2)	6799(1)	26(1)
C10	6452(2)	8164(2)	6980(1)	29(1)
C11	7501(2)	8285(2)	6980(1)	32(1)
C12	8063(2)	7549(2)	6815(1)	34(1)
C13	7574(2)	6699(2)	6639(1)	32(1)
C14	6520(2)	6581(2)	6620(1)	29(1)
C15	5023(2)	6691(2)	7998(1)	26(1)
C16	5931(2)	7101(2)	8612(1)	31(1)
C17	6225(2)	6919(2)	9481(2)	35(1)
C18	5605(2)	6320(2)	9764(1)	35(1)
C19	4716(2)	5900(2)	9179(2)	33(1)
C20	4440(2)	6075(2)	8303(1)	28(1)
C21	3301(2)	5433(2)	6948(1)	27(1)
C22	4041(2)	4913(2)	6691(2)	33(1)
C23	3779(2)	4706(2)	5820(2)	37(1)
C24	2753(2)	4981(2)	5229(2)	35(1)
C25	2031(2)	5502(2)	5499(1)	29(1)
C26	2286(2)	5754(2)	6371(1)	26(1)
C27	643(2)	5528(2)	7056(1)	28(1)

C28	409(2)	5604(2)	7813(2)	38(1)
C29	-120(2)	4899(2)	8071(2)	44(1)
C30	-425(2)	4117(2)	7578(2)	39(1)
C31	-206(2)	4035(2)	6822(2)	33(1)
C32	331(2)	4731(2)	6568(1)	30(1)
C33	533(2)	6972(2)	5830(1)	28(1)
C34	-490(2)	6642(2)	5335(1)	31(1)
C35	-1115(2)	7106(2)	4598(2)	39(1)
C36	-735(2)	7902(2)	4352(2)	43(1)
C37	279(2)	8238(2)	4845(2)	42(1)
C38	905(2)	7781(2)	5577(2)	34(1)

Table 3.   Bond lengths [Å] and angles [°] for neimca24.

Fe(1)-P(1)	2.1825(6)	C(9)-C(14)	1.396(3)
Fe(1)-C(1)	2.049(2)	C(10)-H(10)	0.9500
Fe(1)-C(2)	2.057(2)	C(10)-C(11)	1.393(3)
Fe(1)-C(21)	2.073(2)	C(11)-H(11)	0.9500
Fe(1)-C(22)	2.112(2)	C(11)-C(12)	1.387(4)
Fe(1)-C(23)	2.085(3)	C(12)-H(12)	0.9500
Fe(1)-C(24)	2.081(2)	C(12)-C(13)	1.385(4)
Fe(1)-C(25)	2.081(2)	C(13)-H(13)	0.9500
Fe(1)-C(26)	2.105(2)	C(13)-C(14)	1.388(3)
P(1)-C(3)	1.835(2)	C(14)-H(14)	0.9500
P(1)-C(9)	1.843(2)	C(15)-C(16)	1.407(3)
P(1)-C(15)	1.843(2)	C(15)-C(20)	1.389(3)
P(2)-C(26)	1.832(2)	C(16)-H(16)	0.9500
P(2)-C(27)	1.837(2)	C(16)-C(17)	1.383(3)
P(2)-C(33)	1.836(2)	C(17)-H(17)	0.9500
O(1)-C(20)	1.379(3)	C(17)-C(18)	1.386(4)
O(1)-C(21)	1.383(3)	C(18)-H(18)	0.9500
C(1)-C(2)	1.419(4)	C(18)-C(19)	1.379(4)
C(1)-H(1A)	1.03(3)	C(19)-H(19)	0.9500
C(1)-H(1B)	1.01(3)	C(19)-C(20)	1.393(3)
C(2)-H(2A)	0.97(3)	C(21)-C(22)	1.414(3)
C(2)-H(2B)	1.04(3)	C(21)-C(26)	1.426(3)
C(3)-C(4)	1.403(3)	C(22)-C(23)	1.398(4)
C(3)-C(8)	1.394(3)	C(22)-H(22)	0.94(3)
C(4)-H(4)	0.9500	C(23)-C(24)	1.425(4)
C(4)-C(5)	1.382(3)	C(23)-H(23)	0.96(3)
C(5)-H(5)	0.9500	C(24)-C(25)	1.407(3)
C(5)-C(6)	1.387(4)	C(24)-H(24)	0.93(3)
C(6)-H(6)	0.9500	C(25)-C(26)	1.416(3)
C(6)-C(7)	1.385(4)	C(25)-H(25)	0.96(3)
C(7)-H(7)	0.9500	C(27)-C(28)	1.399(3)
C(7)-C(8)	1.389(3)	C(27)-C(32)	1.398(3)
C(8)-H(8)	0.9500	C(28)-H(28)	0.9500
C(9)-C(10)	1.393(3)	C(28)-C(29)	1.394(4)

C(29)-H(29)	0.9500	C(21)-Fe(1)-C(24)	83.58(10)
C(29)-C(30)	1.382(4)	C(21)-Fe(1)-C(25)	70.83(9)
C(30)-H(30)	0.9500	C(21)-Fe(1)-C(26)	39.91(8)
C(30)-C(31)	1.387(4)	C(22)-Fe(1)-P(1)	96.86(7)
C(31)-H(31)	0.9500	C(23)-Fe(1)-P(1)	127.99(8)
C(31)-C(32)	1.386(3)	C(23)-Fe(1)-C(22)	38.91(10)
C(32)-H(32)	0.9500	C(23)-Fe(1)-C(26)	86.94(10)
C(33)-C(34)	1.398(3)	C(24)-Fe(1)-P(1)	167.52(8)
C(33)-C(38)	1.400(3)	C(24)-Fe(1)-C(22)	70.90(11)
C(34)-H(34)	0.9500	C(24)-Fe(1)-C(23)	40.02(10)
C(34)-C(35)	1.391(3)	C(24)-Fe(1)-C(25)	39.52(9)
C(35)-H(35)	0.9500	C(24)-Fe(1)-C(26)	72.01(9)
C(35)-C(36)	1.384(4)	C(25)-Fe(1)-P(1)	144.78(7)
C(36)-H(36)	0.9500	C(25)-Fe(1)-C(22)	85.01(10)
C(36)-C(37)	1.391(4)	C(25)-Fe(1)-C(23)	72.68(10)
C(37)-H(37)	0.9500	C(25)-Fe(1)-C(26)	39.54(8)
C(37)-C(38)	1.381(4)	C(26)-Fe(1)-P(1)	107.41(6)
C(38)-H(38)	0.9500	C(26)-Fe(1)-C(22)	72.96(9)
C(1)-Fe(1)-P(1)	92.83(8)	C(3)-P(1)-Fe(1)	115.79(7)
C(1)-Fe(1)-C(2)	40.45(11)	C(3)-P(1)-C(9)	104.45(10)
C(1)-Fe(1)-C(21)	166.68(10)	C(3)-P(1)-C(15)	100.44(10)
C(1)-Fe(1)-C(22)	127.27(11)	C(9)-P(1)-Fe(1)	118.38(7)
C(1)-Fe(1)-C(23)	97.89(11)	C(15)-P(1)-Fe(1)	115.20(8)
C(1)-Fe(1)-C(24)	92.85(10)	C(15)-P(1)-C(9)	99.93(10)
C(1)-Fe(1)-C(25)	113.99(9)	C(26)-P(2)-C(27)	99.05(10)
C(1)-Fe(1)-C(26)	150.10(9)	C(26)-P(2)-C(33)	99.87(10)
C(2)-Fe(1)-P(1)	96.28(8)	C(33)-P(2)-C(27)	104.31(10)
C(2)-Fe(1)-C(21)	152.52(10)	C(20)-O(1)-C(21)	122.06(17)
C(2)-Fe(1)-C(22)	162.57(11)	Fe(1)-C(1)-H(1A)	108.6(17)
C(2)-Fe(1)-C(23)	123.79(11)	Fe(1)-C(1)-H(1B)	116.6(18)
C(2)-Fe(1)-C(24)	95.26(11)	C(2)-C(1)-Fe(1)	70.07(14)
C(2)-Fe(1)-C(25)	90.83(10)	C(2)-C(1)-H(1A)	118.0(17)
C(2)-Fe(1)-C(26)	113.68(10)	C(2)-C(1)-H(1B)	118.1(19)
C(21)-Fe(1)-P(1)	88.37(6)	H(1A)-C(1)-H(1B)	116(3)
C(21)-Fe(1)-C(22)	39.49(9)	Fe(1)-C(2)-H(2A)	111.3(18)
C(21)-Fe(1)-C(23)	71.18(10)	Fe(1)-C(2)-H(2B)	117.0(17)

C(1)-C(2)-Fe(1)	69.48(14)	C(14)-C(13)-H(13)	119.9
C(1)-C(2)-H(2A)	119.4(19)	C(9)-C(14)-H(14)	119.8
C(1)-C(2)-H(2B)	120.1(18)	C(13)-C(14)-C(9)	120.5(2)
H(2A)-C(2)-H(2B)	112(3)	C(13)-C(14)-H(14)	119.8
C(4)-C(3)-P(1)	120.93(18)	C(16)-C(15)-P(1)	119.26(17)
C(8)-C(3)-P(1)	120.21(17)	C(20)-C(15)-P(1)	123.74(17)
C(8)-C(3)-C(4)	118.4(2)	C(20)-C(15)-C(16)	116.83(19)
C(3)-C(4)-H(4)	119.8	C(15)-C(16)-H(16)	118.9
C(5)-C(4)-C(3)	120.5(2)	C(17)-C(16)-C(15)	122.1(2)
C(5)-C(4)-H(4)	119.8	C(17)-C(16)-H(16)	118.9
C(4)-C(5)-H(5)	119.7	C(16)-C(17)-H(17)	120.3
C(4)-C(5)-C(6)	120.6(2)	C(16)-C(17)-C(18)	119.4(2)
C(6)-C(5)-H(5)	119.7	C(18)-C(17)-H(17)	120.3
C(5)-C(6)-H(6)	120.3	C(17)-C(18)-H(18)	120.0
C(7)-C(6)-C(5)	119.3(2)	C(19)-C(18)-C(17)	120.0(2)
C(7)-C(6)-H(6)	120.3	C(19)-C(18)-H(18)	120.0
C(6)-C(7)-H(7)	119.8	C(18)-C(19)-H(19)	119.9
C(6)-C(7)-C(8)	120.5(2)	C(18)-C(19)-C(20)	120.1(2)
C(8)-C(7)-H(7)	119.8	C(20)-C(19)-H(19)	119.9
C(3)-C(8)-H(8)	119.7	O(1)-C(20)-C(15)	125.20(19)
C(7)-C(8)-C(3)	120.6(2)	O(1)-C(20)-C(19)	113.1(2)
C(7)-C(8)-H(8)	119.7	C(15)-C(20)-C(19)	121.5(2)
C(10)-C(9)-P(1)	124.13(17)	O(1)-C(21)-Fe(1)	134.79(16)
C(10)-C(9)-C(14)	118.8(2)	O(1)-C(21)-C(22)	120.9(2)
C(14)-C(9)-P(1)	116.84(17)	O(1)-C(21)-C(26)	115.15(19)
C(9)-C(10)-H(10)	119.7	C(22)-C(21)-Fe(1)	71.76(13)
C(11)-C(10)-C(9)	120.6(2)	C(22)-C(21)-C(26)	123.9(2)
C(11)-C(10)-H(10)	119.7	C(26)-C(21)-Fe(1)	71.27(12)
C(10)-C(11)-H(11)	120.0	Fe(1)-C(22)-H(22)	128.8(19)
C(12)-C(11)-C(10)	119.9(2)	C(21)-C(22)-Fe(1)	68.75(13)
C(12)-C(11)-H(11)	120.0	C(21)-C(22)-H(22)	120.5(18)
C(11)-C(12)-H(12)	120.1	C(23)-C(22)-Fe(1)	69.48(15)
C(13)-C(12)-C(11)	119.9(2)	C(23)-C(22)-C(21)	118.7(2)
C(13)-C(12)-H(12)	120.1	C(23)-C(22)-H(22)	120.4(18)
C(12)-C(13)-H(13)	119.9	Fe(1)-C(23)-H(23)	128(2)
C(12)-C(13)-C(14)	120.3(2)	C(22)-C(23)-Fe(1)	71.61(14)

C(22)-C(23)-C(24)	118.9(2)	C(30)-C(29)-C(28)	120.3(2)
C(22)-C(23)-H(23)	122.1(19)	C(30)-C(29)-H(29)	119.8
C(24)-C(23)-Fe(1)	69.84(14)	C(29)-C(30)-H(30)	120.1
C(24)-C(23)-H(23)	118.9(19)	C(29)-C(30)-C(31)	119.8(2)
Fe(1)-C(24)-H(24)	126.0(19)	C(31)-C(30)-H(30)	120.1
C(23)-C(24)-Fe(1)	70.14(14)	C(30)-C(31)-H(31)	120.0
C(23)-C(24)-H(24)	118.0(18)	C(32)-C(31)-C(30)	120.0(2)
C(25)-C(24)-Fe(1)	70.25(13)	C(32)-C(31)-H(31)	120.0
C(25)-C(24)-C(23)	121.3(2)	C(27)-C(32)-H(32)	119.5
C(25)-C(24)-H(24)	120.2(18)	C(31)-C(32)-C(27)	121.1(2)
Fe(1)-C(25)-H(25)	128.7(17)	C(31)-C(32)-H(32)	119.5
C(24)-C(25)-Fe(1)	70.22(13)	C(34)-C(33)-P(2)	125.56(18)
C(24)-C(25)-C(26)	121.3(2)	C(34)-C(33)-C(38)	118.8(2)
C(24)-C(25)-H(25)	118.6(16)	C(38)-C(33)-P(2)	115.63(17)
C(26)-C(25)-Fe(1)	71.14(12)	C(33)-C(34)-H(34)	119.9
C(26)-C(25)-H(25)	119.9(16)	C(35)-C(34)-C(33)	120.2(2)
P(2)-C(26)-Fe(1)	131.85(13)	C(35)-C(34)-H(34)	119.9
C(21)-C(26)-Fe(1)	68.83(12)	C(34)-C(35)-H(35)	119.8
C(21)-C(26)-P(2)	117.69(16)	C(36)-C(35)-C(34)	120.4(2)
C(25)-C(26)-Fe(1)	69.32(12)	C(36)-C(35)-H(35)	119.8
C(25)-C(26)-P(2)	126.54(17)	C(35)-C(36)-H(36)	120.1
C(25)-C(26)-C(21)	115.8(2)	C(35)-C(36)-C(37)	119.7(2)
C(28)-C(27)-P(2)	117.28(19)	C(37)-C(36)-H(36)	120.1
C(32)-C(27)-P(2)	124.21(17)	C(36)-C(37)-H(37)	119.9
C(32)-C(27)-C(28)	118.3(2)	C(38)-C(37)-C(36)	120.3(3)
C(27)-C(28)-H(28)	119.8	C(38)-C(37)-H(37)	119.9
C(29)-C(28)-C(27)	120.4(3)	C(33)-C(38)-H(38)	119.7
C(29)-C(28)-H(28)	119.8	C(37)-C(38)-C(33)	120.6(2)
C(28)-C(29)-H(29)	119.8	C(37)-C(38)-H(38)	119.7

Table 4. Anisotropic displacement parameters ($Å^2$x 10^3) for neimca24. The anisotropic displacement factor exponent takes the form: $-2\pi^2$[h^2 $a^{*2}U_{11}$ + ... + 2 h k a* b* U_{12}]

	U_{11}	U_{22}	U_{33}	U_{23}	U_{13}	U_{12}
Fe1	25(1)	28(1)	22(1)	-3(1)	8(1)	-3(1)
P1	26(1)	25(1)	19(1)	-1(1)	8(1)	-2(1)
P2	26(1)	30(1)	21(1)	-2(1)	8(1)	-1(1)
O1	27(1)	44(1)	25(1)	7(1)	7(1)	-4(1)
C1	36(1)	51(2)	23(1)	-4(1)	13(1)	-10(1)
C2	37(1)	42(1)	22(1)	3(1)	5(1)	-5(1)
C3	24(1)	30(1)	26(1)	-4(1)	8(1)	-4(1)
C4	28(1)	31(1)	26(1)	-3(1)	8(1)	-4(1)
C5	31(1)	37(1)	32(1)	-8(1)	9(1)	0(1)
C6	30(1)	30(1)	44(1)	-5(1)	8(1)	2(1)
C7	35(1)	31(1)	36(1)	3(1)	9(1)	-2(1)
C8	30(1)	33(1)	29(1)	0(1)	10(1)	-1(1)
C9	29(1)	31(1)	16(1)	-1(1)	7(1)	-1(1)
C10	31(1)	30(1)	26(1)	-2(1)	10(1)	-4(1)
C11	30(1)	36(1)	28(1)	-4(1)	8(1)	-7(1)
C12	29(1)	45(1)	28(1)	-6(1)	12(1)	-6(1)
C13	30(1)	38(1)	27(1)	-5(1)	10(1)	2(1)
C14	30(1)	32(1)	24(1)	-2(1)	8(1)	-2(1)
C15	29(1)	28(1)	21(1)	0(1)	9(1)	3(1)
C16	35(1)	34(1)	23(1)	-4(1)	10(1)	-5(1)
C17	39(1)	40(1)	24(1)	-5(1)	9(1)	-3(1)
C18	41(1)	43(1)	21(1)	2(1)	11(1)	4(1)
C19	34(1)	39(1)	27(1)	5(1)	13(1)	1(1)
C20	25(1)	32(1)	25(1)	2(1)	8(1)	2(1)
C21	26(1)	27(1)	30(1)	5(1)	10(1)	-4(1)
C22	29(1)	27(1)	44(1)	6(1)	12(1)	3(1)
C23	33(1)	28(1)	53(2)	-8(1)	20(1)	-4(1)
C24	34(1)	36(1)	37(1)	-17(1)	15(1)	-11(1)
C25	24(1)	33(1)	28(1)	-7(1)	8(1)	-7(1)
C26	24(1)	30(1)	27(1)	-1(1)	10(1)	-4(1)
C27	25(1)	35(1)	24(1)	1(1)	7(1)	2(1)

C28	41(1)	45(2)	32(1)	-3(1)	18(1)	0(1)
C29	48(2)	53(2)	39(1)	1(1)	25(1)	-5(1)
C30	33(1)	45(2)	43(1)	11(1)	18(1)	1(1)
C31	26(1)	35(1)	35(1)	2(1)	7(1)	-1(1)
C32	27(1)	36(1)	25(1)	1(1)	7(1)	0(1)
C33	28(1)	32(1)	24(1)	-2(1)	10(1)	3(1)
C34	30(1)	34(1)	29(1)	-1(1)	10(1)	2(1)
C35	33(1)	46(2)	33(1)	-1(1)	5(1)	8(1)
C36	48(1)	46(2)	32(1)	9(1)	10(1)	14(1)
C37	53(2)	38(1)	37(1)	7(1)	18(1)	3(1)
C38	36(1)	35(1)	32(1)	-1(1)	12(1)	0(1)

Table 5. Hydrogen coordinates (x 10^4) and isotropic displacement parameters ($Å^2$ x 10^3) for neimca24.

	x	y	z	U(eq)
H4	3690	7949	8027	35
H5	2760	9305	7999	41
H6	2486	10346	6874	43
H7	3204	10040	5798	42
H8	4192	8708	5843	37
H10	6075	8666	7105	35
H11	7830	8871	7092	38
H12	8782	7628	6823	40
H13	7961	6194	6530	38
H14	6185	5999	6486	35
H16	6355	7517	8423	37
H17	6846	7201	9880	42
H18	5794	6198	10360	42
H19	4290	5490	9373	40
H28	612	6141	8154	45
H29	-273	4955	8588	53
H30	-783	3637	7757	47
H31	-424	3502	6478	40
H32	489	4665	6054	36
H34	-759	6099	5502	37
H35	-1807	6875	4261	47
H36	-1164	8217	3848	51
H37	541	8784	4678	50
H38	1594	8017	5912	41
H2A	2430(30)	6810(20)	4470(20)	44(8)
H1A	4030(20)	6000(20)	4562(19)	41(8)
H2B	3120(30)	7690(20)	5050(20)	46(8)
H25	1380(20)	5721(19)	5074(17)	30(7)
H24	2610(20)	4880(20)	4646(19)	37(7)
H22	4730(20)	4760(20)	7088(19)	38(8)

H23	4280(30)	4400(20)	5600(20)	48(9)
H1B	4780(30)	6910(20)	5150(20)	50(9)

Table 6. Torsion angles [°] for neimca24.

Fe1-P1-C3-C4	98.80(17)	C3-C4-C5-C6	0.9(3)
Fe1-P1-C3-C8	-73.65(19)	C4-C3-C8-C7	-1.9(3)
Fe1-P1-C9-C10	144.69(16)	C4-C5-C6-C7	-1.2(4)
Fe1-P1-C9-C14	-40.57(18)	C5-C6-C7-C8	0.0(4)
Fe1-P1-C15-C16	161.34(16)	C6-C7-C8-C3	1.6(4)
Fe1-P1-C15-C20	-23.5(2)	C8-C3-C4-C5	0.7(3)
Fe1-C21-C22-C23	-50.1(2)	C9-P1-C3-C4	-129.16(18)
Fe1-C21-C26-P2	-127.21(16)	C9-P1-C3-C8	58.4(2)
Fe1-C21-C26-C25	52.58(18)	C9-P1-C15-C16	33.3(2)
Fe1-C22-C23-C24	-53.2(2)	C9-P1-C15-C20	-151.5(2)
Fe1-C23-C24-C25	-50.2(2)	C9-C10-C11-C12	-1.4(3)
Fe1-C24-C25-C26	-51.7(2)	C10-C9-C14-C13	1.1(3)
Fe1-C25-C26-P2	127.43(19)	C10-C11-C12-C13	1.0(3)
Fe1-C25-C26-C21	-52.34(18)	C11-C12-C13-C14	0.4(4)
P1-C3-C4-C5	-171.91(18)	C12-C13-C14-C9	-1.5(3)
P1-C3-C8-C7	170.75(18)	C14-C9-C10-C11	0.3(3)
P1-C9-C10-C11	174.95(17)	C15-P1-C3-C4	-25.95(19)
P1-C9-C14-C13	-173.93(17)	C15-P1-C3-C8	161.61(18)
P1-C15-C16-C17	174.2(2)	C15-P1-C9-C10	-89.4(2)
P1-C15-C20-O1	2.3(3)	C15-P1-C9-C14	85.30(17)
P1-C15-C20-C19	-172.68(18)	C15-C16-C17-C18	-0.6(4)
P2-C27-C28-C29	-174.6(2)	C16-C15-C20-O1	177.5(2)
P2-C27-C32-C31	174.88(17)	C16-C15-C20-C19	2.5(3)
P2-C33-C34-C35	179.64(19)	C16-C17-C18-C19	1.1(4)
P2-C33-C38-C37	-179.7(2)	C17-C18-C19-C20	0.2(4)
O1-C21-C22-Fe1	-131.9(2)	C18-C19-C20-O1	-177.6(2)
O1-C21-C22-C23	178.1(2)	C18-C19-C20-C15	-2.1(4)
O1-C21-C26-Fe1	131.50(19)	C20-O1-C21-Fe1	-38.2(3)
O1-C21-C26-P2	4.3(3)	C20-O1-C21-C22	56.4(3)
O1-C21-C26-C25	-175.91(19)	C20-O1-C21-C26	-126.2(2)
C3-P1-C9-C10	14.2(2)	C20-C15-C16-C17	-1.2(4)
C3-P1-C9-C14	-171.10(16)	C21-O1-C20-C15	28.7(3)
C3-P1-C15-C16	-73.5(2)	C21-O1-C20-C19	-155.9(2)
C3-P1-C15-C20	101.6(2)	C21-C22-C23-Fe1	49.7(2)

C21-C22-C23-C24	-3.5(4)	C35-C36-C37-C38	0.0(4)
C22-C21-C26-Fe1	-51.2(2)	C36-C37-C38-C33	0.3(4)
C22-C21-C26-P2	-178.41(18)	C38-C33-C34-C35	0.9(3)
C22-C21-C26-C25	1.4(3)		
C22-C23-C24-Fe1	54.1(2)		
C22-C23-C24-C25	3.8(4)		
C23-C24-C25-Fe1	50.2(2)		
C23-C24-C25-C26	-1.5(4)		
C24-C25-C26-Fe1	51.3(2)		
C24-C25-C26-P2	178.69(18)		
C24-C25-C26-C21	-1.1(3)		
C26-P2-C27-C28	141.57(19)		
C26-P2-C27-C32	-32.8(2)		
C26-P2-C33-C34	94.6(2)		
C26-P2-C33-C38	-86.68(19)		
C26-C21-C22-Fe1	51.0(2)		
C26-C21-C22-C23	0.9(4)		
C27-P2-C26-Fe1	-176.90(14)		
C27-P2-C26-C21	-91.35(18)		
C27-P2-C26-C25	88.9(2)		
C27-P2-C33-C34	-7.5(2)		
C27-P2-C33-C38	171.24(17)		
C27-C28-C29-C30	-0.3(4)		
C28-C27-C32-C31	0.5(3)		
C28-C29-C30-C31	-0.2(4)		
C29-C30-C31-C32	0.9(4)		
C30-C31-C32-C27	-1.1(3)		
C32-C27-C28-C29	0.2(4)		
C33-P2-C26-Fe1	76.73(16)		
C33-P2-C26-C21	162.29(18)		
C33-P2-C26-C25	-17.5(2)		
C33-P2-C27-C28	-115.73(19)		
C33-P2-C27-C32	69.9(2)		
C33-C34-C35-C36	-0.5(4)		
C34-C33-C38-C37	-0.8(4)		
C34-C35-C36-C37	0.1(4)		
